# Computer Vision Enables Monitoring and Kinetic Analysis of Structurally Diverse Carbon Monoxide Surrogates

**DOI:** 10.1002/anie.8888536

**Published:** 2026-05-21

**Authors:** Kristin Donnachie, Ciaran Griffin, Morven L. Gray, Timothy J. D. McCabe, Marc Reid

**Affiliations:** ^1^ Department of Pure and Applied Chemistry University of Strathclyde Glasgow UK

**Keywords:** carbon monoxide surrogates, carbonylation, computer vision, kinetics, reaction monitoring

## Abstract

Carbon monoxide (CO) surrogates are valuable synthetic reagents that circumvent the hazards of handling CO gas directly. However, owing to the structural diversity of available surrogates, their varied triggering conditions, and the requirement for sealed reactor systems, quantitative understanding of their CO release kinetics has remained elusive. Here, we report a non‐contact computer vision method for monitoring and comparing vessel‐specific CO release from 10 structurally diverse surrogates in two‐chamber COware reactors. By tracking the colorimetric response of a ruthenium‐based chemosensor, we establish a scoring system based on cumulative CO flux—from surrogate activation through gas‐phase mass transfer to sensor capture—benchmarked against a CO balloon reference. This approach reveals a fourfold range in surrogate reactivity scores and enables systematic investigation of how reaction parameters such as base strength, solvent polarity, and stirring rate modulate CO release kinetics. We demonstrate that these kinetic differences translate directly to carbonylation reaction outcomes: in Pd‐catalyzed Suzuki–Miyaura carbonylation, surrogate score correlates linearly with product conversion, consistent with an inverse dependence on CO concentration. The methods presented enable rational surrogate selection and open new possibilities for the quantitative design of synthetic methodologies dependent on controlled gas release.

## Graphical abstract

A non‐contact computer vision method enables monitoring and quantitative comparison of vessel‐specific CO release kinetics from structurally diverse surrogates in two‐chamber COware reactors, revealing that surrogate reactivity scores correlate with carbonylation reaction outcomes.

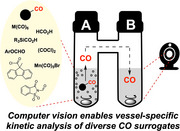

## 1 Introduction

Carbon monoxide (CO) is a ubiquitous C‐1 source in synthetic chemistry. Under transition metal‐mediated conditions, it is used to effect a wide range of chemical transformations, including aminocarbonylation, reductive carbonylation, Sonogashira carbonylation, hydroxycarbonylation, alkoxy‐carbonylation, and Suzuki carbonylation [1, 2]. However, due to the acute health risks associated with use and storage of CO gas [3], CO surrogates—bench‐stable molecules that are triggered to undergo controlled release of CO gas in situ—present a safe alternative to using CO balloons and cylinders. A suite of CO surrogates has been identified, from transition metal hexacarbonyls to organic molecules like formic acid and *N*‐formylsaccharin. Hand in hand with the structural diversity of available CO surrogates, the mechanisms of triggering CO release also range widely [1, 4, 5, 6, 7, 8, 9, 10, 11, 12, 13, 14].

**FIGURE 1:**
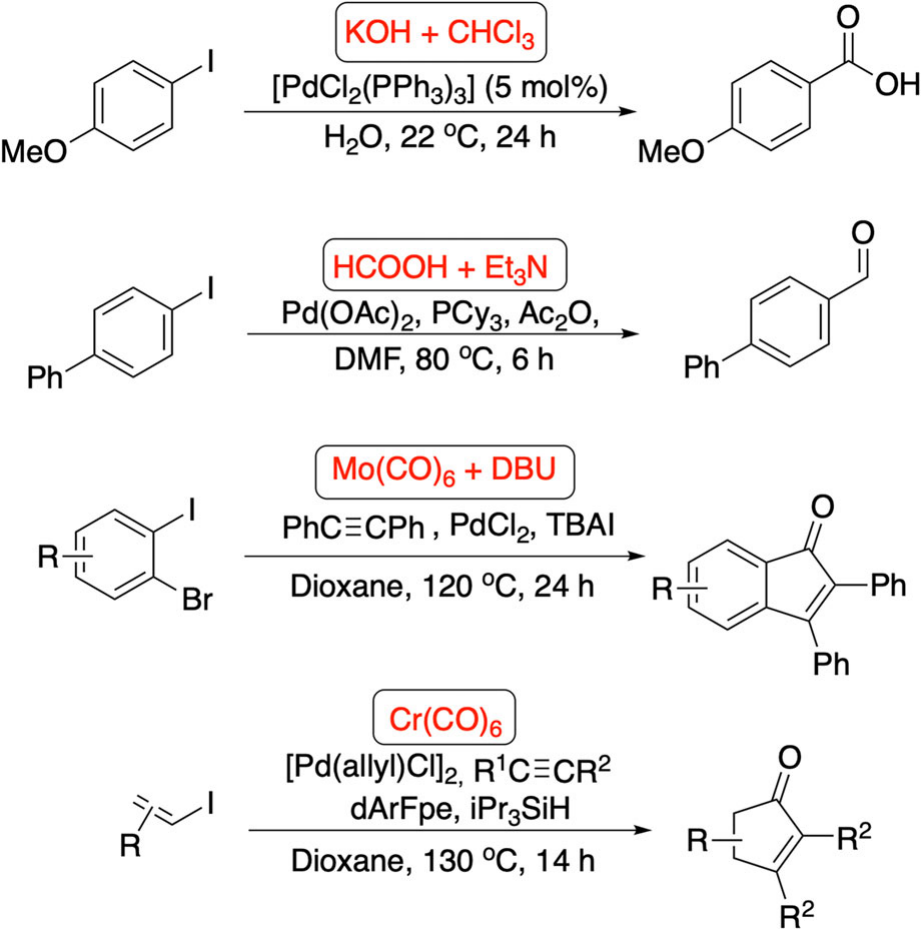
Examples of reported Pd‐catalyzed carbonylation reactions effected by a range of CO surrogates and gas release triggers (highlighted in boxes above the reaction arrows).

The applications of CO surrogates in carbonylation chemistry are extensive (Figure 1). An early example was reported by Alper and Grushin on carboxylation of aryl iodides using chloroform as the source of CO [15]. Further examples include formic acid as the CO source for Pd‐catalyzed reductive carbonylation of aryl iodides to form aromatic aldehydes [5, 6]. Group six hexacarbonyls have been widely utilized. Mo(CO)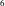 has been used as a CO source in the synthesis of indenones and as an ex situ source during aminocarbonylation reactions using aryl iodides and bromides [7, 8], while Cr(CO)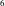 has been employed in Pauson–Khand reactions of vinyl iodides with alkynes (Figure 1) [11].

**FIGURE 2:**
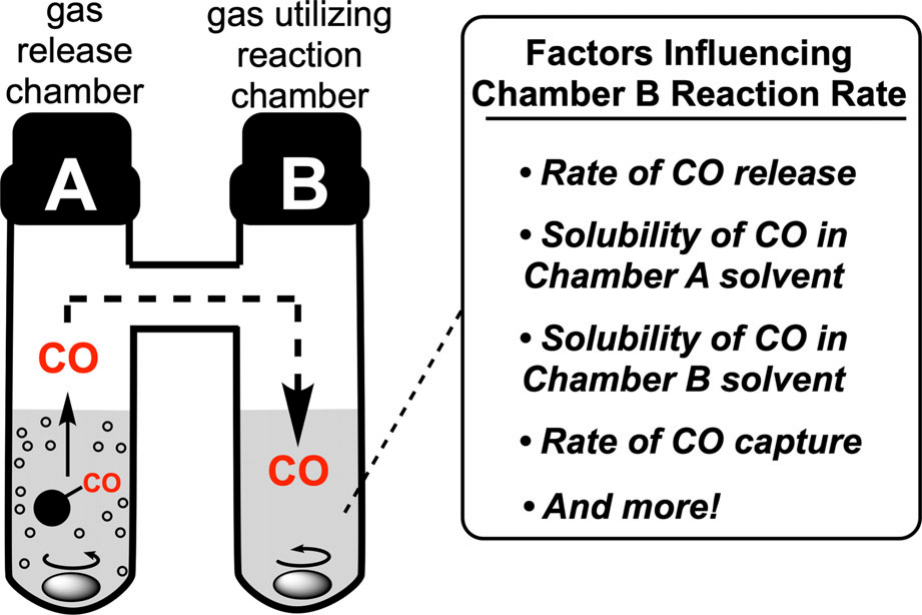
Simplified representation of two‐chamber reactor for clean release of CO from a surrogate, in Chamber A, and capture of the CO for another reaction, in Chamber B.

Beyond one‐pot uses of CO surrogates [15, 16, 17], two‐chamber methods have been developed, allowing CO to be generated in one chamber and fed to another, thus avoiding possible side reactions and other complications inherent in one‐pot methods. COware, developed and commercialized by Skrydstrup, represents the most widely known method for enabling separation of the CO release from surrogates and use of the released CO on accessible laboratory scales (Figure 2) [10, 12, 13, 18].

## 2 Barriers to Vessel‐Specific Kinetics of CO Surrogates

### 2.1 Precedented Efforts to Capture CO Surrogate Kinetics

Despite the success of two‐chamber systems for the development of carbonylation processes, access to vessel‐specific kinetic understanding of CO surrogates applied in such reactors remains a challenge. Lab‐based gas kinetics methods include gas uptake syringes [19], eudiometric or inverted burette‐based methods [20], and the use of pressure gauges [21]. However, these methods have significant limitations, including susceptibility to human error, a demand for some level of invasive interaction with the reactor, and incompatibility with certain reactor types [19].

Aligned with the focus of our study, several previous attempts have been made to measure gas evolution and reaction kinetics within two‐chamber systems and the associated challenges [19, 22, 23]. Gockel et al. focused on the release of CO from a chloroform and cesium hydroxide system with the aim of carrying out palladium‐catalyzed aminocarbonylation reactions [19]. During the exploratory stages of their investigation, they looked into the base used to trigger the release of CO from the surrogate. They carried out a gas evolution study using a glass vial, septum, needle, and plastic luer lock syringe and monitored the syringe for up to 24 h. A limitation of this method was its low sensitivity, requiring relatively high pressure to move the piston of the syringe to measure gas uptake volume. While the data provided by the team evidenced valuable temporal resolution to gain insight into the gas evolution kinetics, this method is limited by the need for human interaction to take a volume reading at each of the desired time points. Furthermore, with regard to capturing vessel‐specific kinetics, the syringe‐based gas uptake approach fails to capture the physically significant contributions to observed rates of CO consumption, including release from Chamber A, mass transfer across the chamber bridge, and dissolution of CO in the Chamber B solvent.

In 2023, Iqubal et al. measured the pressure within COware during the hydrogenation of a large scope of substrates, using an Al/$H_{2}O$/NaOH system as an $H_{2}$ surrogate. They acquired the data by using a small pressure gauge attached to one chamber [22]. Focus was placed on the final pressure of the system rather than the evolution of gas and increase in pressure over time. The method worked very well to determine the final pressure in the system. However, to be able to use the pressure gauge, adaptations to the glassware and equipment had to be made, leaving the system susceptible to gas leaks.

These intriguing and fleeting attempts to understand the bulk reaction kinetics of two‐chamber reaction kinetics involving gas release and capture inspired the focus of our study. We hypothesized that vessel‐specific kinetics of structurally diverse CO surrogates could be captured and compared non‐invasively using computer vision analysis of colorimetric CO‐responsive chemosensors.

In doing so, the choice of CO surrogate in reaction design could be placed under more quantified control. This motivated our investigation of camera‐enabled reaction monitoring to enable the recording of vessel‐specific kinetics of two‐chamber systems.

### 2.2 Computer Vision for Chemical Reaction Monitoring

**FIGURE 3:**
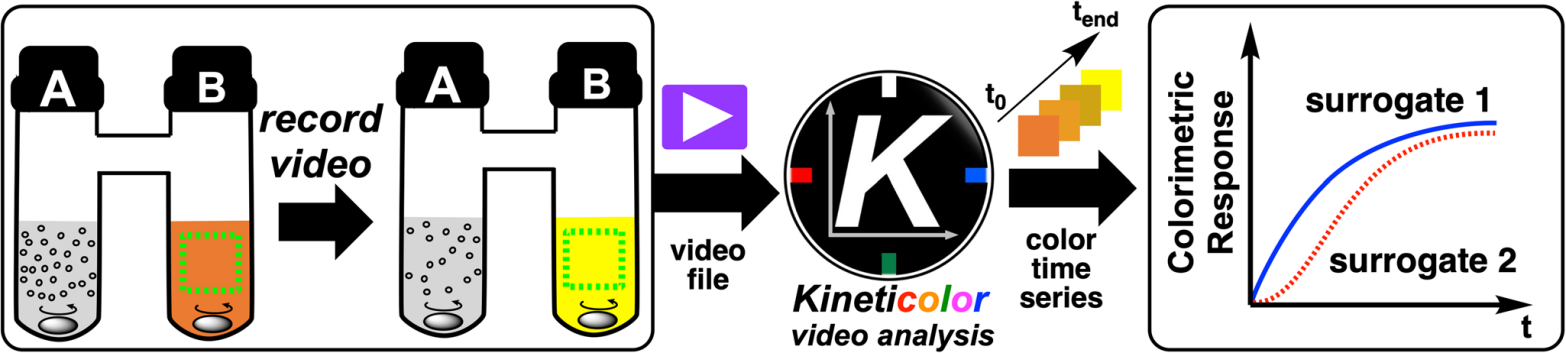
Simplified representation of a typical workflow of recording video data with which to generate colorimetric time series. In the present case, these data are being extracted from cameras focused on CO sequestration processes taking place in Chamber B. The green dashed region within Chamber B denotes the pixel‐defined region of interest from which colorimetric time series data are extracted.

Computer vision, while defined on more specific terms elsewhere [24], serves the chemist by enabling computers to extract information from digital images and videos. Of relevance to the present study, this methodology enables chemists to derive reaction kinetics from the visible bulk of a process from a video recording of that process. We, and others, have contributed to the chemically specific applications of this field over the past decade [24, 25, 26, 27, 28, 29, 30, 31]. Our contributions to computer vision for chemical reaction monitoring have focused on the development of *Kineticolor*, a computer vision software platform enabling chemists to extract color, spatial, and shape kinetic analyses from video footage recording the visible bulk of chemical reactions (Figure 3). The software has been applied to various process monitoring problems [32, 33, 34, 35, 36, 37], the most notable (in relation to this study) being a study on palladium catalyst degradation and productive catalyst lifetime [34]. This study employed computer vision reaction monitoring to assess diagnostic color differences in reaction mixtures whose bulk appearance differed under air, nitrogen, and argon atmospheres.

Across computing and printing, color can be encoded using various models [38]. For example, RGB (red, green, blue) and CMYK (cyan, magenta, yellow, and black) are two such models; t the former is more commonly applied in computer monitors while the latter is predominantly used in printing processes [39, 40]. In *Kineticolor*, color changes extracted from video footage of reactions are captured as RGB (red, green, blue) values, the native output format of digital camera sensors. Subsequently, these data can be converted into additional color spaces, including CIE $L^{*}a^{*}b^{*}$ and HSV (Hue, Saturation, and Value) [38]. CIE $L^{*}a^{*}b^{*}$ encodes color as lightness ($L^{*}$), a green–red axis ($a^{*}$), and a blue–yellow axis ($b^{*}$) within a perceptually uniform space, whereas HSV represents color through its dominant wavelength (hue), purity (saturation), and brightness (value). RGB is used as the acquisition format because it is generated directly by digital imaging sensors; CMYK is not applicable to camera‐based data capture. Among the RGB‐derived representations, hue was selected for this study because it isolates the dominant wavelength of color into a single metric that is robust to variations in lighting intensity (see “Choice of Hue” section below).

### 2.3 Study Aims

To understand how we might collect and compare the two‐chamber reaction kinetics of structurally diverse CO surrogates without interfering with the reaction vessel itself, we pursued three interconnected aims:

1. Develop a non‐contact monitoring method: Establish a computer vision‐based approach to track CO release and capture kinetics within COware reactors, avoiding the limitations of invasive measurement techniques such as gas uptake syringes and pressure gauges.
2. Enable quantitative comparison across surrogates: Create a scoring system that allows structurally diverse CO surrogates, regardless of their activation mechanism, to be placed on a common kinetic scale, benchmarked against a CO balloon as the reference standard.
3. Demonstrate practical relevance: Investigate whether the kinetic differences captured by our method translate to observable effects on carbonylation reaction outcomes, thereby validating the utility of surrogate scoring for reaction design.

### 2.4 Methodological Approach

Our proposed methodology combined three key components: (i) COware two‐chamber reactors, which physically separate the CO release process (Chamber A) from CO utilization (Chamber B); (ii) a colorimetric CO chemosensor placed in Chamber B, providing a visible readout of CO arrival and capture; and (iii) *Kineticolor* computer vision software, which extracts time‐resolved color data from video recordings of the sensor solution (Figure 3).

**FIGURE 4:**
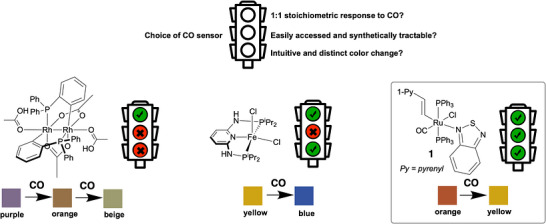
Choosing the optimal sensor for exploring a computer vision approach to monitoring CO uptake. Complex **1** was selected based on superior synthetic tractability, signal‐to‐noise ratio, and plateau definition compared to alternative sensors (see Section S2 for comparative data).

The foundation of this approach is the identification of a suitable CO chemosensor whose color change upon CO binding can be reliably tracked by video analysis. Several CO chemosensors have been reported in the literature, including ${\mathrm{Rh}}_{2}$(OAc)
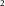
(HOAc)
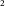
‐${\left({\mathrm{PPh}}_{2}-C_{6}H_{4}\right)}_{2}$ [41], [${\mathrm{FeCl}}_{2}$(PNP‐iPr)] [42], [Ru(CH = CHPyr‐1)Cl(CO)(BTD)${\left({\mathrm{PPh}}_{3}\right)}_{2}$] [43], and ${\mathrm{PdCl}}_{2}$ [44]. After extensive evaluation of these candidates (see Supporting Information, Section S2), complex **1**, the ruthenium‐based sensor [Ru(CH = CHPyr‐1)Cl(CO)(BTD)${\left({\mathrm{PPh}}_{3}\right)}_{2}$], was selected for this study (Figure 4).

Compared to other sensors explored, complex **1** offered the optimal balance of: (i) synthetic tractability, enabling preparation of sufficient quantities for a comprehensive surrogate study; (ii) sensitivity to CO at the concentrations relevant to COware experiments; and (iii) a color change profile most easily facilitating plateau analysis and other modeling efforts applied to extracting kinetic insights (vide infra). While complex **1** is not strictly CO‐specific, it provides a well‐defined, stoichiometric color response to CO gas via ligand substitution. This mechanism (one incoming CO molecule replacing one coordinated ligand) results in a color change (orange to yellow) that is intuitive as well as trackable.

### 2.5 Choice of Hue as the Key Colorimetric Metric

Color extracted from video footage can be encoded in multiple color spaces. In *Kineticolor*, raw pixel data are captured as RGB values and subsequently converted to other representations, including CIE L*a*b* and HSV [38, 45, 46, 47, 48]. For this study, we focused specifically on hue as the primary metric for tracking CO‐induced color changes.

**FIGURE 5:**
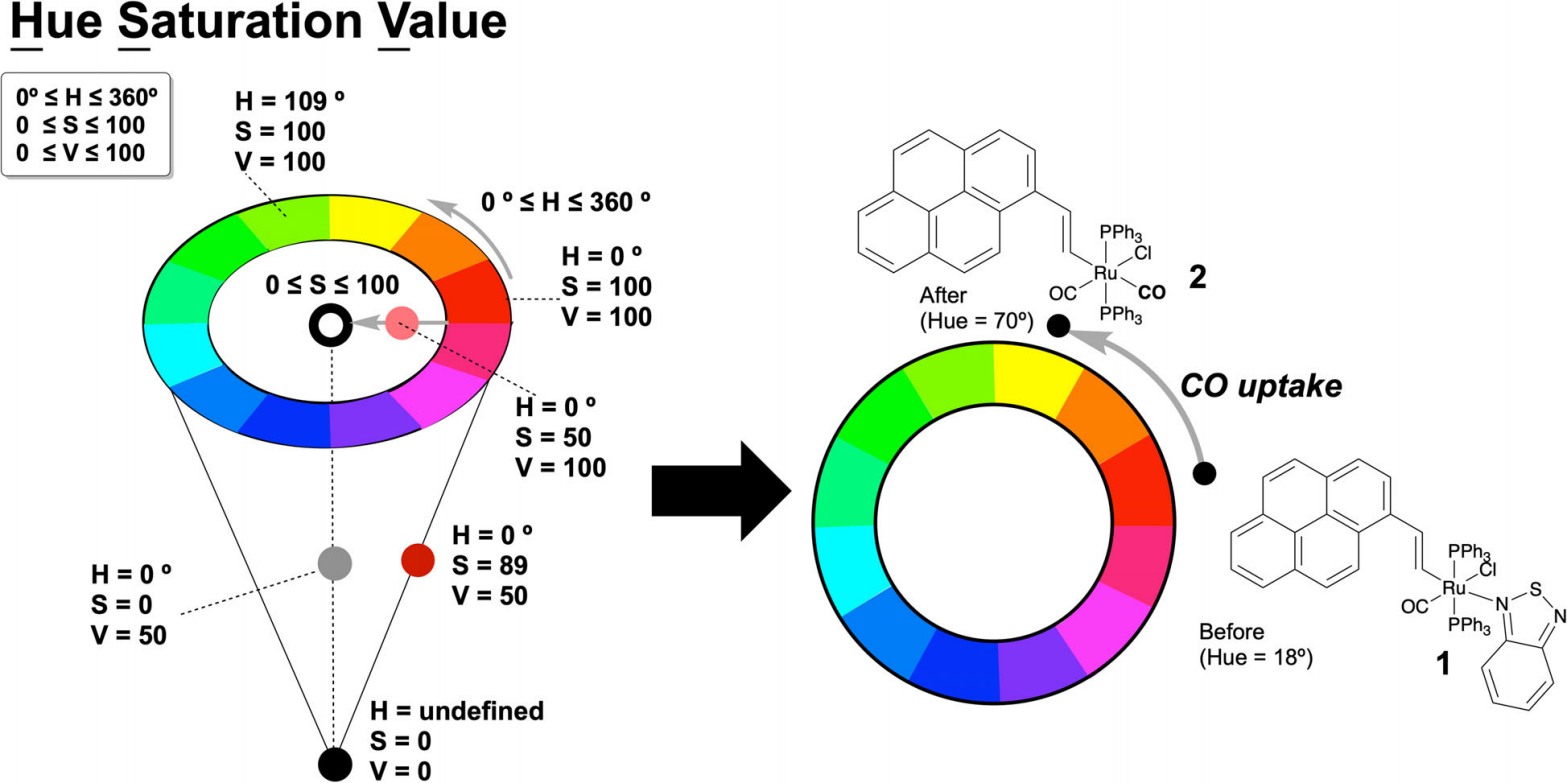
Left: Visual representation of hue in the broader context of the hue, saturation and value color space. Right: The significance of hue placed into the context of this study. Complex **1** has an orange hue of $18^{\circ }$. When the complex is exposed to CO gas it becomes yellow, increasing the hue to $70^{\circ }$ [38].

Hue represents the dominant wavelength of perceived color, expressed as an angular value between 0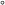 and 360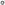 on a circular color wheel (Figure 5) [49]. This metric offers several advantages for reaction monitoring applications:

- **Lighting independence**. Unlike RGB values or color saturation, hue is relatively insensitive to variations in illumination intensity. Minor fluctuations in ambient lighting or camera exposure therefore have minimal impact on the extracted kinetic data.
- **Single‐value representation**. Hue condenses the color information into a single numerical descriptor, enabling straightforward plotting against time and facilitating curve fitting with standard kinetic models.
- **Intuitive mapping**. For the orange ($∼18^{\circ }$) to yellow ($∼70^{\circ }$) transition of complex **1**, the hue increase maps directly onto the visual perception of the color change, making the resulting kinetic traces immediately interpretable to the experimentalist.

Upon exposure to CO, ruthenium complex **1** undergoes substitution of its benzothiadiazole (BTD) ligand to form the CO‐bound adduct, complex **2** (Figure 5). This ligand exchange produces a pronounced color change from orange to yellow, corresponding to a hue shift from approximately $18^{\circ }$ to $70^{\circ }$. Critically, the orange‐to‐yellow transition avoids the problematic red/magenta region of the hue circle where values wrap from $360^{\circ }$ to $0^{\circ }$, which would complicate time‐series analysis.

By monitoring hue as a function of time in Chamber B, we obtain a colorimetric time series that encodes the cumulative kinetics of CO release from the surrogate in Chamber A, mass transfer across the chamber bridge, dissolution into the Chamber B solvent, and capture by the chemosensor. This composite signal, which we term the *vessel‐specific kinetics*, reflects the integrated behavior of the two‐chamber system under realistic operating conditions, precisely the information required to inform rational surrogate selection.

With this methodology established, we set out to characterize the CO release kinetics of 10 structurally diverse surrogates and to investigate how variations in reaction conditions affected their gas evolution kinetics across COware.

## 3 Results and Discussion

**FIGURE 6:**
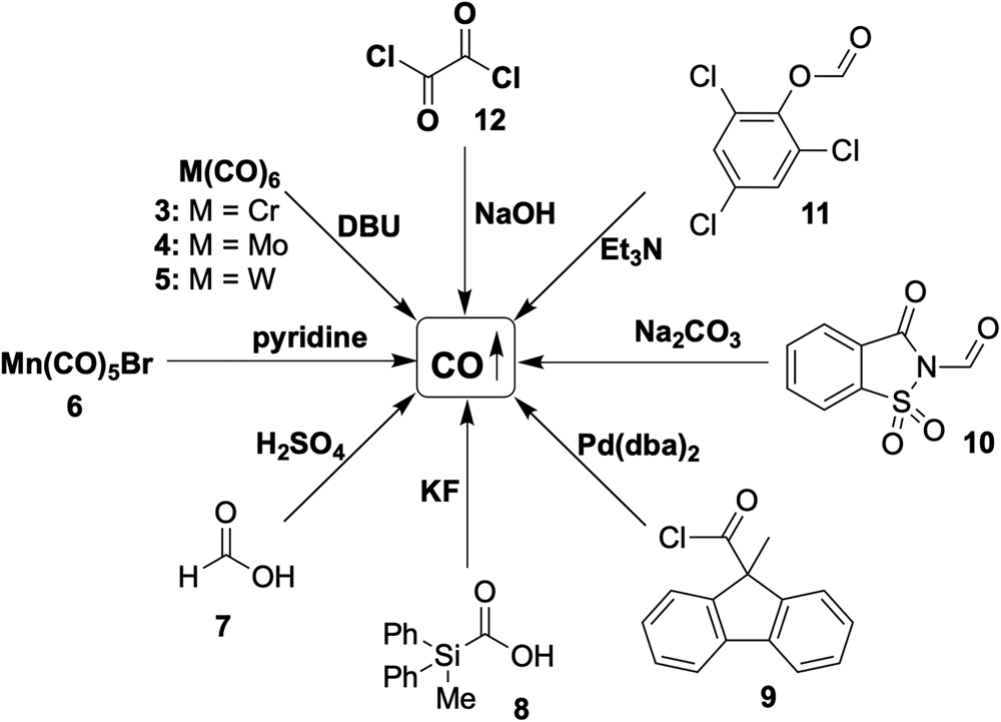
Selected CO surrogates used in this study, highlighting structural diversity and the differing mechanistic triggers required for CO release (e.g., nucleophilic, hydrolytic, acid‐promoted, metal‐catalyzed).

We selected 10 structurally diverse CO surrogates (compounds **3**–**12**) that employ different activation triggers for CO release (Figure 6). Of these, oxalyl chloride (**12**) was subsequently excluded from scored comparisons due to co‐release of HCl and ${\mathrm{CO}}_{2}$ (vide infra), giving nine surrogates with quantified surrogate scores. Using our proposed methodology combining COware, a CO chemosensor [43], and *Kineticolor*, we obtained previously inaccessible kinetic insights on surrogate reactivity for the first time.

### 3.1 Proof‐of‐Concept

**FIGURE 7:**
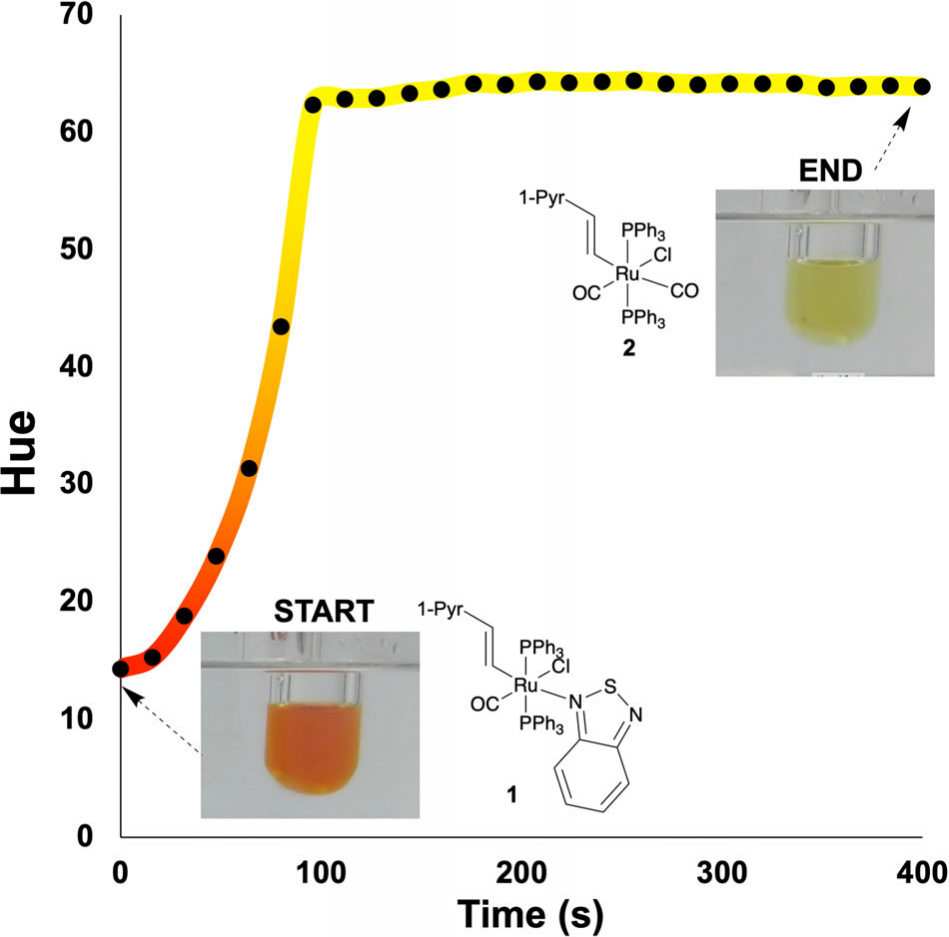
Structural and visual change seen when complex **1** is exposed to CO, in the context hue versus time, tracking the bulk color change of sensor‐containing Chamber B.

When exposed to CO the hue of complex **1** gradually increased as the color of the sensor changed from orange to yellow. This visual change is caused by the replacement of the benzothiadiazole (BTD) ligand with CO to form complex **2** (Figure 7) [43].

### 3.2 Exploring the Limits of Gas Pressure Measurements

We initially explored pressure‐based monitoring using commercial apparatus [50] but encountered multiple limitations: incompatibility with COware geometry necessitated alternative vessels (eliminating the vessel‐specific kinetics we sought to capture), gas leaks compromised measurement accuracy, detection sensitivity required a threefold scale increase, and volatile solvent evaporation (e.g., chloroform, bp 61 °C) introduced systematic artifacts. These challenges, detailed in Section S1.6, motivated our development of the non‐invasive computer vision approach described below.

### 3.3 Tracking Gas Evolution Using Computer Vision

The CO release kinetics from the selected CO surrogates (Figure 6) were studied using video footage capturing orange to yellow color change as sensor **1** reacted with CO to form **2**, analyzed using *Kineticolor* software. Each experiment targeted the same amount of surrogate to be used in Chamber A, on a scale, which released approximately a 25‐fold excess of CO (2.3 mL, 0.094 mmol) relative to the molar quantity of complex **1** (0.004 mmol) in Chamber B [5, 7, 10, 11, 12, 51, 52, 53, 54]. A 25‐fold molar excess of CO relative to complex **1** was chosen to ensure that the hue–time profiles reflect intrinsic CO release and mass‐transfer kinetics rather than stoichiometric depletion of the surrogate. Under these conditions, the sensor operates as a sub‐stoichiometric probe of the total CO available, and the surrogate score captures the kinetic character of CO delivery rather than its thermodynamic capacity. Users investigating different reaction stoichiometries may adjust this excess accordingly, and the scoring framework remains applicable provided the benchmark and surrogates employ the same molar ratio.

**FIGURE 8:**
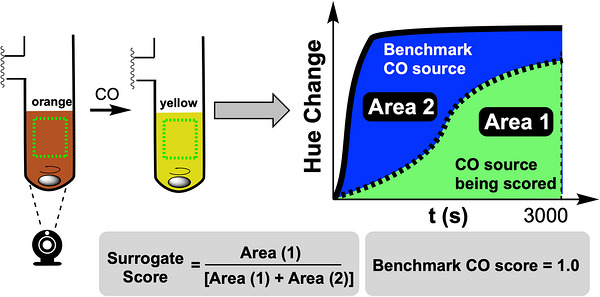
Graphical representation of how each surrogate score was calculated. The score captures the *cumulative flux* of CO from Chamber A to B, for a fixed quantity of sensor across a fixed monitoring period. This approach captures the impact of both observed rate and total volume of CO released in a single metric.

We set out to develop a methodology to quantitatively score each source of CO, based on the *cumulative flux* of CO gas migrating from Chamber A to B within a set time and fixed quantity of sensor. We define cumulative flux as the time‐integrated quantity of CO transferred from Chamber A to Chamber B over the monitoring period, as reflected by the area under the hue–time curve (Figure 8). This single metric captures both the rate of CO delivery and the total extent of CO transfer, providing a holistic measure of surrogate performance under standardized conditions. The comparative score of each surrogate was determined by normalizing the performance of each surrogate relative to a “benchmark” CO source. The benchmark, in this study, was chosen to be a balloon filled with CO. This scoring framework can be readily re‐referenced to alternative benchmarks, should an alternative to a CO balloon (e.g., most reactive surrogate) be desired. The CO balloon delivered gas at ambient pressure ( ~1 atm), consistent with standard laboratory practice for balloon‐mediated gas delivery [10]. No additional pressure measurement was employed for the balloon reference, as the intent was to benchmark surrogates against the most common laboratory‐scale CO delivery method under typical operating conditions.

**FIGURE 9:**
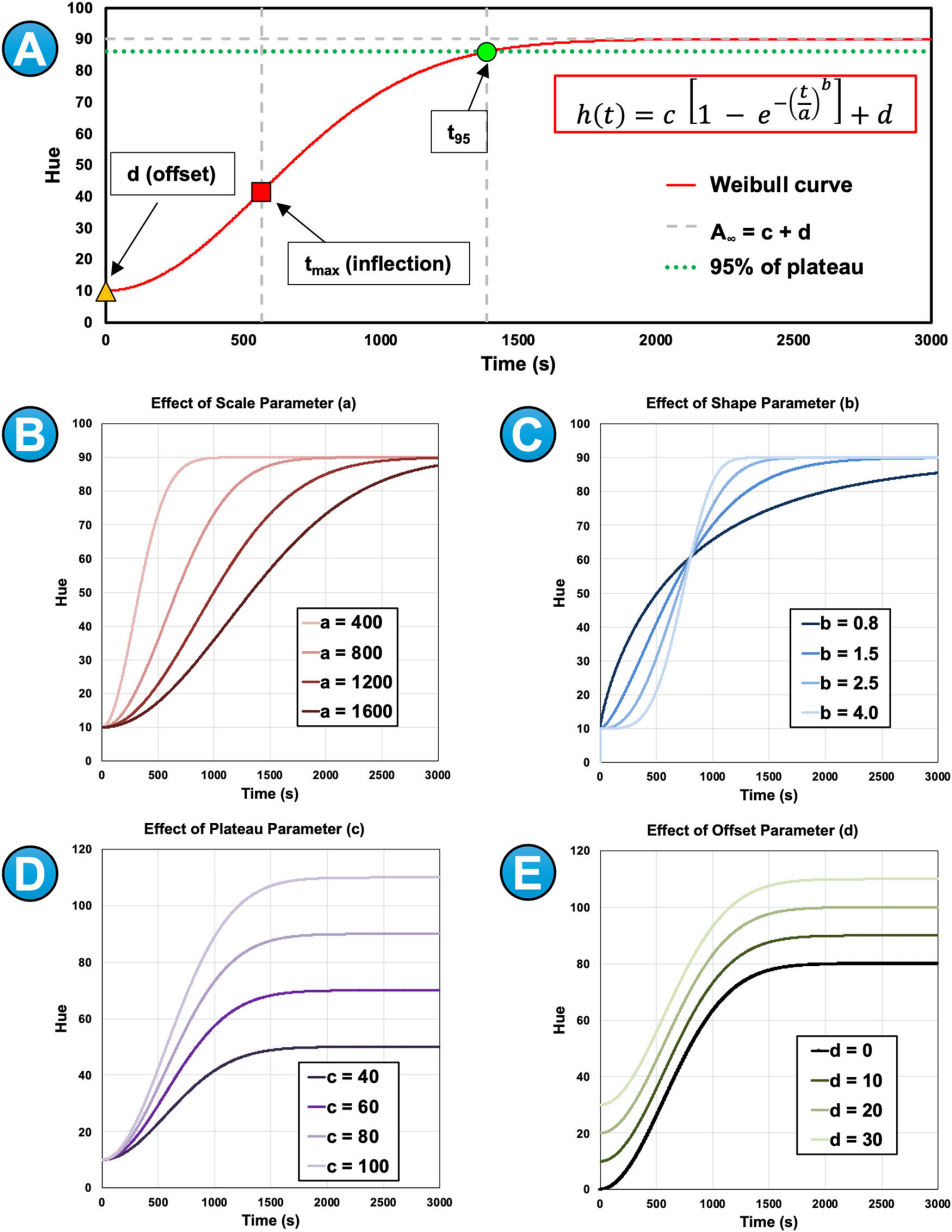
(A) Annotated Weibull curve. Equation used to calculate each curve, with the *t*_max_ and *t*_95_ highlighted. (B) Effect of Scale Parameter (a), this parameter controls the horizontal stretching of the curve fitted to the graph. Larger a value means the curve fitted is stretched more horizontally. (C) Effect of Shape Parameter (b), this parameter controls the graph steepness and sigmoid curve. A higher b value results in a steeper and more sigmodal curve. (D) Effect of Plateau Parameter, this parameter is the value of the max hue reached by the curve. (E) Effect of Offset Parameter, this controls the initial baseline of the Weibull curve fitted. See Section S5. Weibull parameters for each surrogate can be seen in Table S8.

To calculate the areas under the hue versus time profiles of each surrogate and thus calculate the surrogate scores, an appropriate exponential growth model was required. To this end, the Weibull distribution was then fitted to each hue profile to obtain a fitted growth curve (Figure 9). The Weibull distribution was selected following systematic comparison against alternative sigmoidal growth models, including Gompertz, Lay‐modified, Rogers‐modified, and Zwietering‐modified functions. Across the surrogate dataset, the Weibull model consistently outperformed all alternatives (e.g., Gompertz and its modified variants), exhibiting the lowest average sum of squares, root‐mean‐square error, and mean absolute error. These metrics confirmed superior goodness‐of‐fit for capturing the characteristic lag‐exponential‐plateau kinetics of CO uptake by the chemosensor (see Section S5.3 for full model details, and machine‐readable data providing error comparison across alternative growth model functions).

The area under the benchmark curve and the area under the surrogate curve were then used to determine the surrogate's score (Figure 8, above). A score equal to 1 indicates that the surrogate was identical to the benchmark, < 1 the surrogate was less reactive than the benchmark.

**FIGURE 10:**
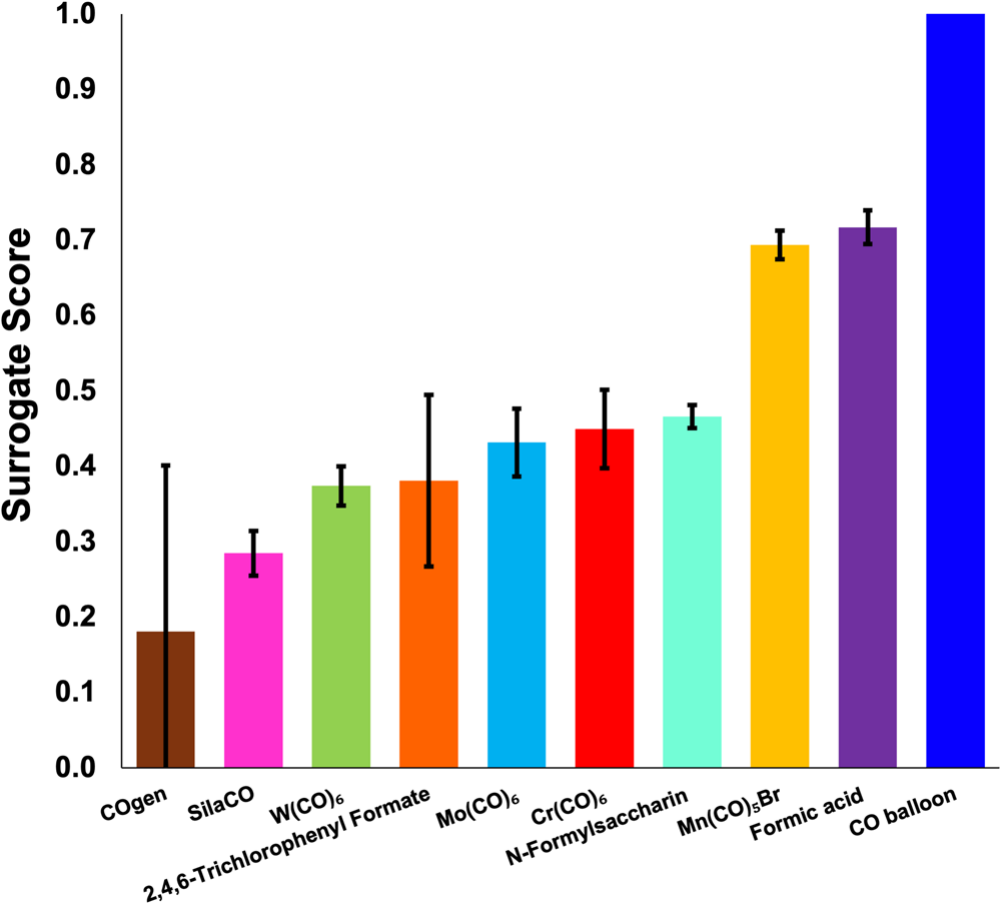
Surrogate scores compared to the benchmark CO balloon. High scores denote higher cumulative flux and faster uptake of CO released from the surrogate in Chamber A by sensor complex **1** in Chamber B. Error bars represent standard deviation from n=3 independent measurements. In all cases, the surrogate reaction in Chamber A was set to release a theoretical 25‐fold excess of CO relative to the molar quantity of complex **1** in Chamber B.

The relative range of CO surrogate scores is shown in Figure 10. The closer the surrogate score is to 1, the more akin its reactivity is to flooding the reactor with CO gas via a balloon. Formic acid was the most reactive surrogate studied, scoring 0.72, closely followed by Mn(CO)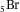 at 0.69. By contrast, COgen gave the lowest recorded score of 0.18, making it stand out as the slowest bleed of CO into Chamber B. As a collective, the group six hexacarbonyls performed similarly to one another. Their scores followed the group trend of Cr (0.45) > Mo (0.43) > W (0.37, consistent with increasing M─CO bond strength down the group, and with known mixed order kinetics of CO substitution at these hexacarbonyls [55].

Turning briefly to the practical choices enabled by this quantified approach to metricizing cumulative flux, consider the two highest scoring surrogates, formic acid and Mn(CO)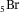. Each is structurally distinct, and each is triggered for CO release by two chemically different methods (addition of $H_{2}{\mathrm{SO}}_{4}$ and an amine base, respectively). With the quantified understanding of similar CO release capabilities of these two surrogates, one might employ these CO sources interchangeably in the design of carbonylation methods in two‐chamber COware. This rational approach could, for example, aid in reducing overall running costs of a reaction, Mn(CO)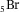 is currently priced at $78 per gram, whereas formic acid is only $0.48 per mL (Merck, Jan 2026).

Among all surrogates studied, oxalyl chloride (**12**) was identified as an informative outlier. Once triggered by the addition of aqueous sodium hydroxide, the recorded rate of color change (hue vs. time) in complex **1** was faster and exhibited a distinct start‐to‐end color profile compared to all other CO surrogates. A deeper investigation showed that the release of ${\mathrm{CO}}_{2}$ and HCl *as well as* CO served to complicate the observed sensor reactions in Chamber B. While no color change was observed when complex **1** was exposed to ${\mathrm{CO}}_{2}$ alone, it was found that the sensor changed color when exposed to gaseous HCl, and did so in a subtly different manner than when the sensor was exposed to CO (see Section S3.1 for studies confirming the presence of HCl). Consequently, oxalyl chloride was removed from the study, as the method was not suitable to monitor a surrogate which releases multiple reactive gases simultaneously.

### 3.4 Exploring Calibrations of Color and Concentration

**FIGURE 11:**
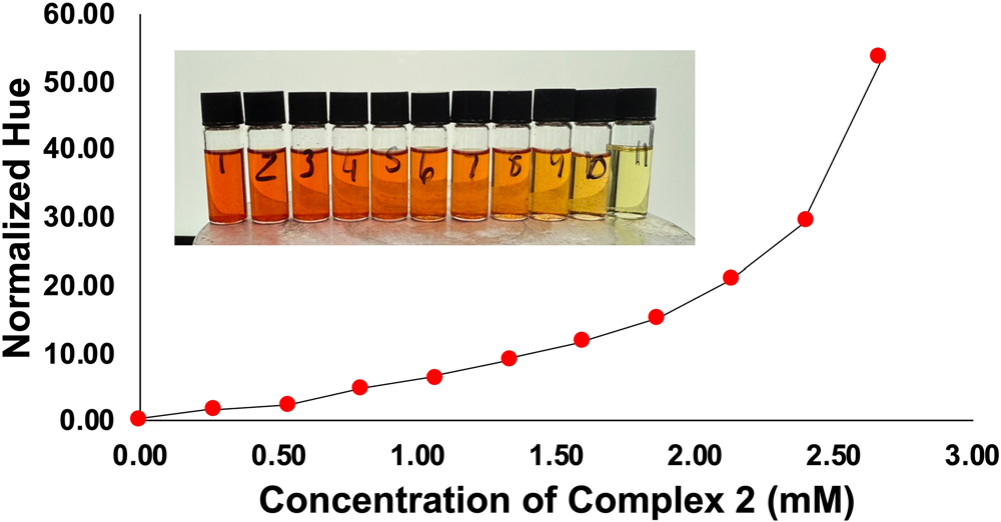
Eleven‐point calibration used to estimate concentration of complex **2** in the sensor chamber during each CO‐releasing reaction. For modeling purposes, hue values were normalized (zero set) by subtracting the value of hue in the first calibration sample from that of all subsequent calibration samples.

While color data derived from video analysis was a sufficient innovation around which to develop vessel‐specific kinetic understanding of CO surrogate performance, we aimed to investigate the possible calibration of color to ground truth concentration, to maximize the intuitive usefulness of our approach in mechanistic investigations. To this end, we measured the hue of a sample containing a known concentration of the [Ru] sensor (complex **1**) and CO‐ligated product complex **2**. An 11‐point calibration curve was created by varying the ratio of sensor complex **1** to CO‐bound complex **2** and de‐ligated by‐product BTD, ranging from 0.004 mmol of complex **1** in vial 1 to 0.004 mmol of complex **2** and BTD in vial 11 (*see Section* *S1.10* *and* *S4* *for full details*). An average hue was obtained from each calibration point by taking a 15‐s video of each solution in the same setup that was used previously within the surrogate investigation. The hue obtained was then plotted against the concentration of CO‐saturated complex **2** present in each sample. Inspired by Sun et al., a piecewise linear calibration curve was created (Figure 11) [56].

**FIGURE 12:**
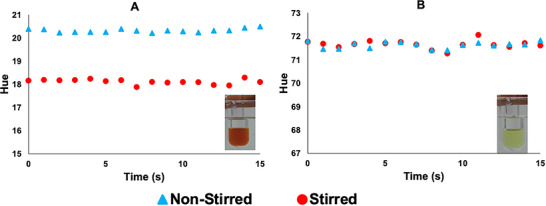
Left: Hue values obtained from *Kineticolor* where vial A contains 0.004 mmol of complex **1**. Right: Hue values obtained from *Kineticolor* where vial B contains 0.004 mmol of complex **2** and 0.004 mmol of BTD. Stirring rate = 400 RPM.

The hue–concentration relationship followed a nonlinear trend. Initial calibration points that contained a higher concentration of complex **1** were found to be more susceptible to variation compared to the later points in the series. The first attempt at video calibration was carried out without mechanical stirring. The observed variability of the early versus later calibration samples was ascribed to the lower solubility of complex **1** versus **2**. At higher concentrations of complex **1**, there remains undissolved solid that settles at the bottom of the COware when not stirred, whereas, when the solution is stirred, the undissolved solid is evenly distributed. This subtle difference in color arising from stirring versus not stirring results in the solution appearing more red with stirring, reducing the recorded hue value versus that observed with no stirring. The variation observed between stirred and non‐stirred reduced as the concentration of the more soluble complex **2** increased (Figure 12).

More generally, we recommend that calibration conditions match experimental conditions as closely as possible. Beyond the presence of undissolved solids discussed above, other factors, including stirring rate, vortex geometry, bubble entrainment, and spatial inhomogeneity of color within the solution, can all influence the perceived bulk color recorded by the camera, independent of chemical composition. Calibrating under matched conditions minimizes these systematic differences and improves the accuracy of concentration estimates derived from the hue–concentration relationship.

**FIGURE 13:**
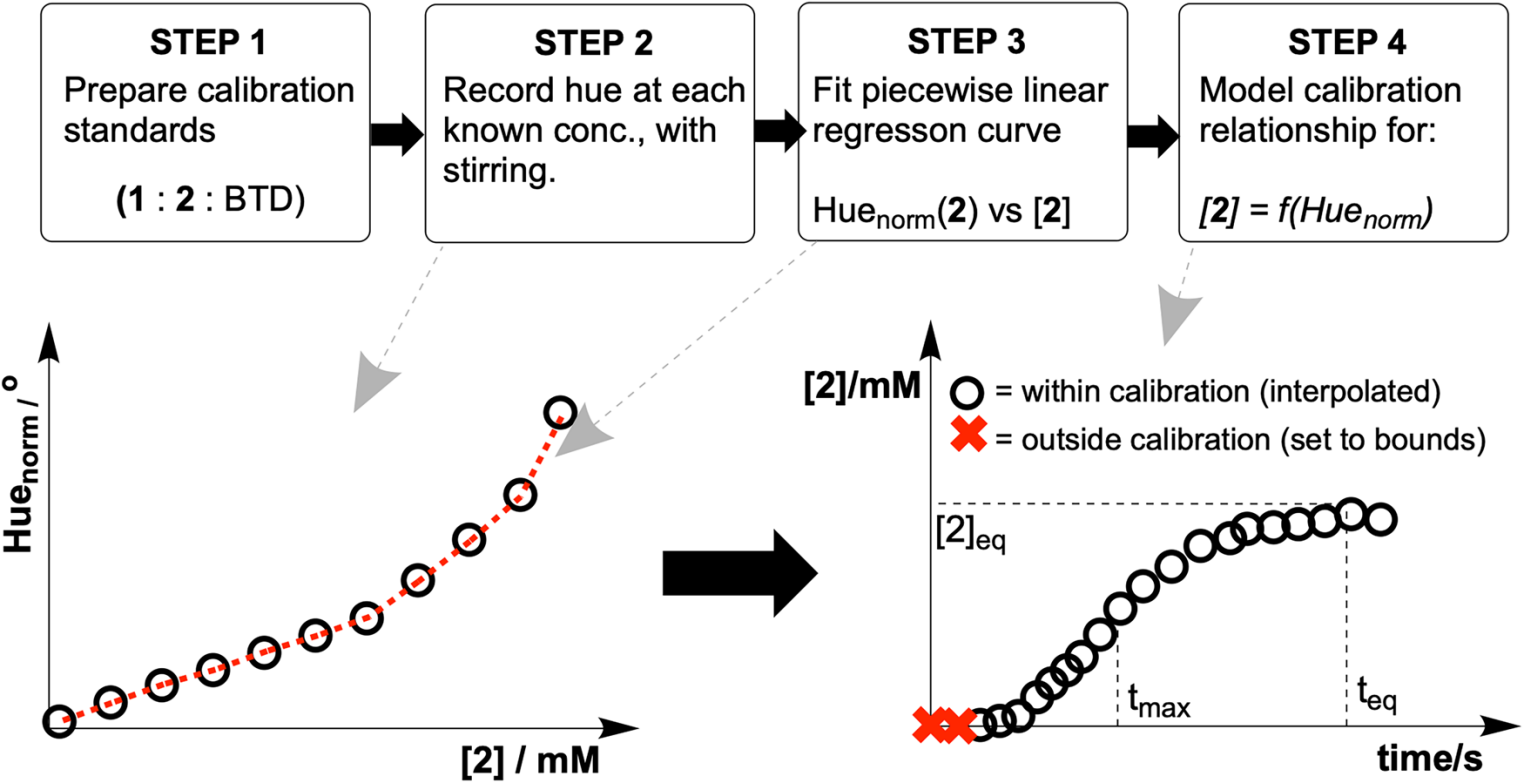
Simplified workflow to determine the concentration of complex **2** from hue data captured from videos of calibration samples. Python scripts and all raw data available in the Supporting Information.

A workflow was devised to determine the concentration of complex **2** in Chamber B over time using hue data from *Kineticolor* analysis (Figure 13). Using this method, kinetic information was derived from the videos, including the time at which complex **1** becomes fully saturated with CO or the surrogate stops producing CO (i.e., plateau time) and the approximate maximum conversion of complex **1** to **2** in the sealed Chamber B (Table 1).

**TABLE 1:** The suite of kinetic metrics enabling comparison of CO surrogates, determined by the hue versus concentration of complex **2** calibration workflow.

Surrogate	Surrogate score	Max rate (mM $s^{-1}$)	Time of max rate (s)	Plateau Time (s)	Max conc. at Plateau (mM)	Time to reach 50% of max conc. (s)
COgen	0.18	0.0029	1088	3120	2.50	1824
SilaCO	0.28	0.0023	1376	2768	2.66	1680
W(CO) 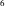	0.37	0.0030	400	2880	2.62	1088
2,4,6‐trichlorophenyl						
formate	0.38	0.0032	1264	2944	2.66	1472
Mo(CO) 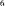	0.43	0.0029	400	2512	2.62	1072
Cr(CO) 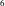	0.45	0.0027	416	2480	2.65	1056
N‐formylsaccharin	0.47	0.0034	448	2496	2.65	976
Mn(CO) 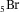	0.69	0.0072	560	1264	2.60	528
Formic acid	0.72	0.0041	784	1360	2.67	720

*Note*: Oxalyl chloride excluded due to HCl/${\mathrm{CO}}_{2}$ co‐release interfering with sensor response as previously discussed.

### 3.5 Impact of Reaction Conditions on CO Release

**FIGURE 14:**
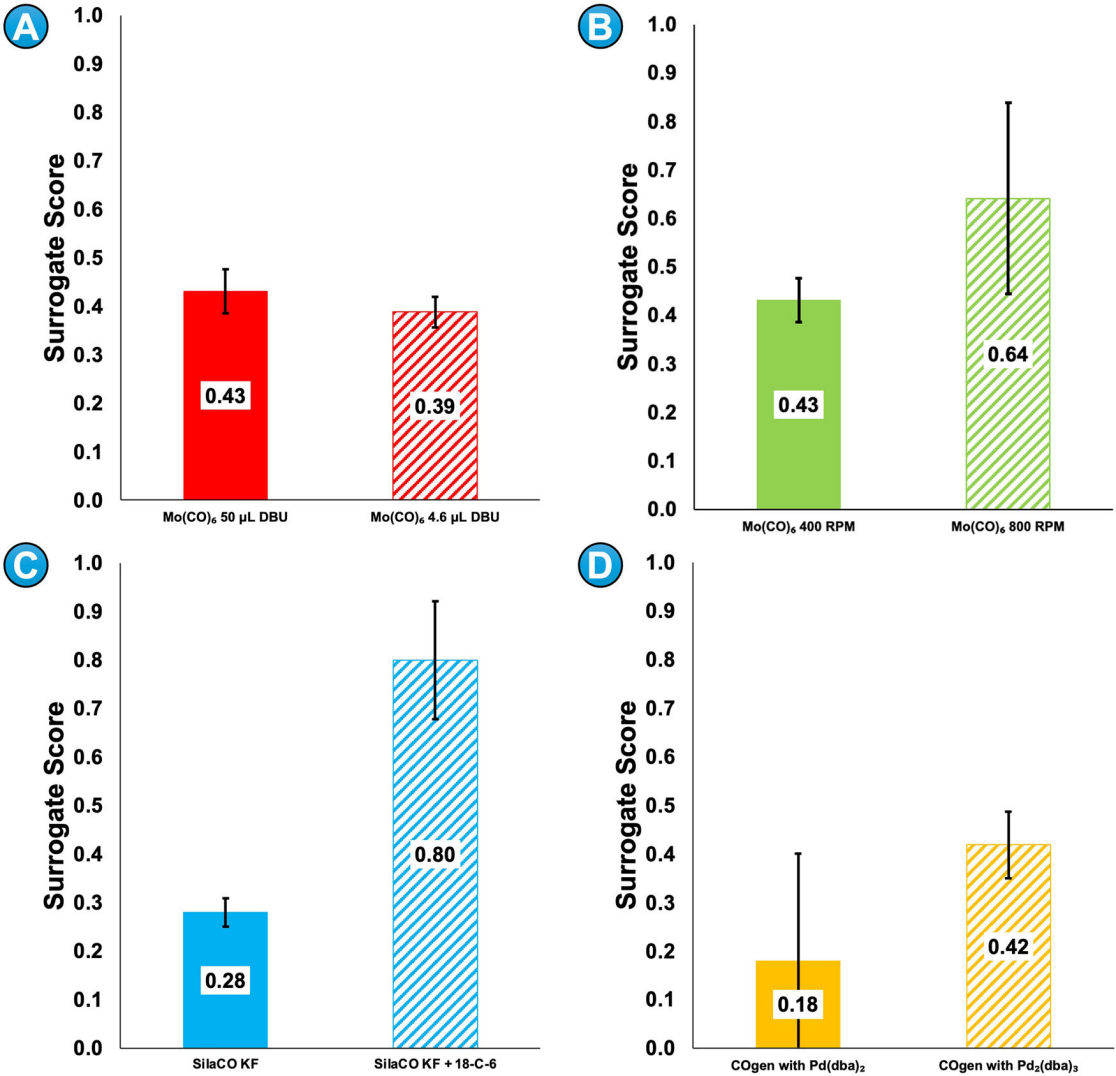
Bar charts of surrogate scores when reaction conditions for CO release are changed. Error bars represent standard deviation from n=3 independent measurements. (A) Solid red bar = original Mo(CO)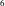 reaction conditions, striped red bar = 10‐fold decrease of DBU trigger added. (B) Solid green bar = original reaction conditions, striped green bar = increased stirring rate in surrogate chamber. (C) Solid blue bar = original SilaCO reaction conditions using KF alone as the CO release trigger, striped blue bar = using 18‐C‐6 as an additive alongside KF. (D) Solid yellow bar = COgen with original Pd source Pd(dba)_2_, striped yellow bar = COgen with a alternative Pd source Pd_2_(dba)_3_.

We next investigated A versus B comparisons of changes to CO release conditions, for four surrogates, beginning with Mo(CO)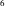, SilaCO, and COgen, with 2,4,6‐trichlorophenylformate examined in Section 3.6. Variations in reaction conditions in Chamber A included changes in the concentration of the trigger, stirring rate, catalyst used, reaction solvent, and the addition of additives alongside the trigger (Figure 14).

When examining Mo(CO)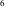, triggered by the addition of the amine base DBU, one might assume that decreasing the amount of DBU would correspondingly decrease the rate of CO uptake by the sensor in Chamber B. However, our *Kineticolor* analysis revealed only a modest but statistically significant reduction in surrogate score upon tenfold decrease in DBU concentration (0.44 ± 0.05 vs. 0.35 ± 0.03; p = 0.044 for a two‐tailed t‐test; Figure 14A). The practical magnitude of this difference (Δscore = 0.09) suggests that the CO release process is relatively insensitive to base stoichiometry under these conditions, consistent with the trigger concentration being above the saturation threshold for the rate‐determining decarbonylation step. DBU was selected as the sole base for this dose–response study in order to isolate the effect of trigger concentration on CO release from a single, well‐characterized base–surrogate combination. Systematic screening of alternative bases (e.g., DABCO, DMAP) represents a logical extension of this work, and the methodology reported herein is readily applicable to such investigations.

A factor that is commonly overlooked in early‐stage reaction development is the impact of mechanical stirring [57, 58, 59]. While the surrogate score increased from 0.43 at 400 RPM to 0.64 at 800 RPM (Figure 14B), inconsistencies between replicates were observed (*see Figure* *S74*). At 400 RPM, the replicates were almost identical with controlled stirring, but when the RPM was increased to 800, the stirring provided by the magnetic flea became erratic and irreproducible. This illustrates that, while increasing the stirring rate does enhance solution to gas phase transfer of CO from Chamber A to B, inconsistencies introduced can be detected using the computer vision approach.

**FIGURE 15:**
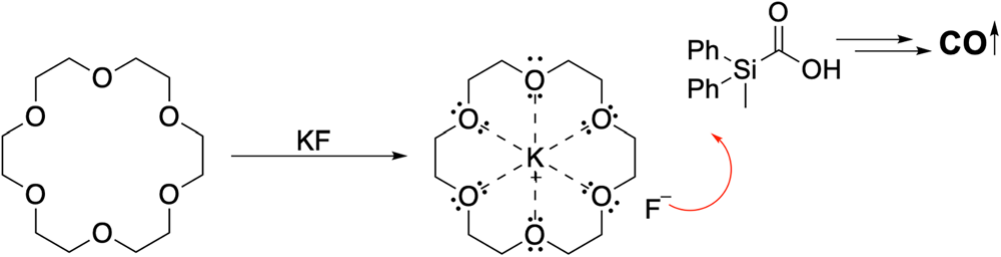
Representation of 18‐C‐6 being used to increase solubility of KF in dioxane to be used in the trigger of CO release from SilaCO.

The release of CO from SilaCO is triggered by potassium fluoride (KF) [12]. However, KF exhibits low solubility in dioxane, which made transferring the very small mass of KF required into the reaction chamber difficult. Consequently, only a small portion of the added KF dissolved in the CO surrogate chamber, resulting in sub‐optimal CO release. To enhance KF solubility, 18‐crown‐6 ether (18‐C‐6) was added [60, 61]. The resulting complex with KF, where $K^{+}$ resides within the ether, provides a naked $F^{-}$ nucleophile to initiate enhanced CO production from SilaCO (Figure 15) [60, 61]. Indeed, SilaCO's surrogate score increased from 0.28 with KF alone to 0.80 using the KF/18‐C‐6 combination (Figure 14C). This substantial improvement in score indicates that, when using the crown ether, SilaCO performs more comparably to the CO balloon benchmark than it does when triggered by KF alone. The study of alternative fluoride sources, such as tetrabutylammonium fluoride (TBAF), would mark an interesting extension of this surrogate comparison study, beyond the initial methodological scope.

An opportunity to increase CO release from COgen was identified by replacing the palladium(0) catalyst Pd(dba)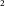 with ${\mathrm{Pd}}_{2}$(dba)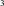. Using ${\mathrm{Pd}}_{2}$(dba)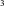 not only increased the CO release rate but also appeared to decrease the initial induction period in the rate of CO uptake in Chamber B. This increase in reaction rate is broadly consistent with the higher concentration of Pd(0) available per molecule of catalyst for the same molar quantity of each Pd source employed. However, more work is required to mechanistically understand this observation. COgen's score improved from 0.18 to 0.42 (Figure 14D). While COgen's performance remains less than half that of the balloon, this example demonstrates that reaction conditions could be further optimized to enhance surrogate performance, should one be bound to use a single CO surrogate, and require a faster release of CO to optimize the reaction taking place in Chamber B.

### 3.6 A Closer Look at Formate‐Derived CO Release

**FIGURE 16:**
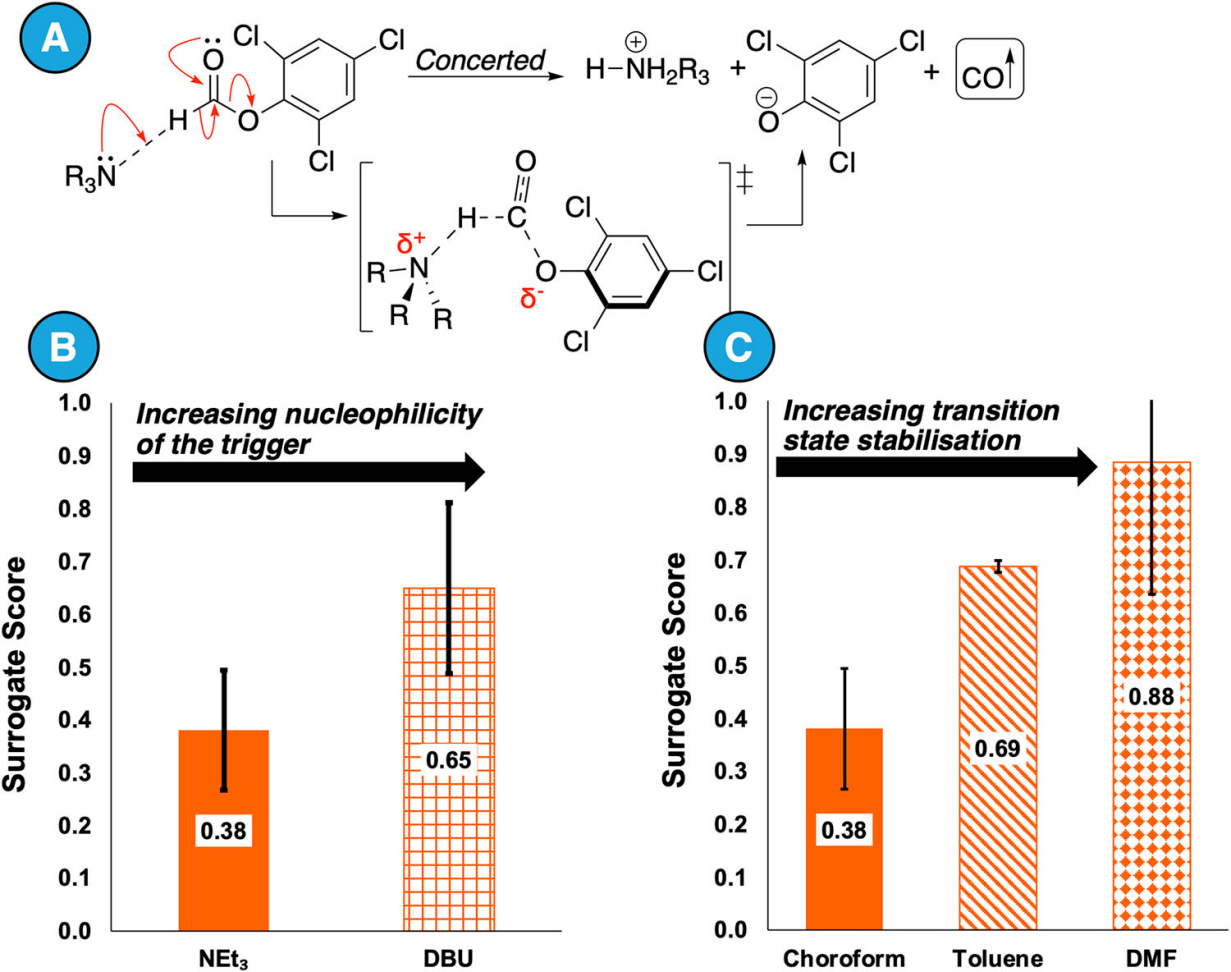
Optimization of CO release from 2,4,6‐trichlorophenyl formate. (A) Proposed E2 mechanism for base‐catalyzed CO release, showing the concerted transition state with developing partial charges. (B) Effect of base on surrogate score: substitution of ${\mathrm{NEt}}_{3}$ with the more basic and nucleophilic DBU increases the score from 0.38 to 0.65. (C) Effect of solvent on surrogate score: polar aprotic solvents (toluene, DMF) outperform the hydrogen bond donor chloroform, with DMF providing the highest score (0.88) due to enhanced stabilization of the charged transition state. Error bars represent standard deviation from n=3 independent measurements.

For the fourth A versus B comparison of changes to CO release conditions, we further examined CO release rates from 2,4,6‐trichlorophenyl formate, focusing on solvent and base selection (Figure 16). As guided by the evidenced mechanism of formate decay (Figure 16A), base selection proved an impactful parameter through which to tune CO surrogate reactivity (Figure 16B). Replacing triethylamine with the stronger base DBU increased the surrogate score from 0.38 to 0.65 (a 71% improvement). This enhancement reflects the greater basicity and nucleophilicity of DBU [62], which facilitates more efficient deprotonation during the rate‐determining step [63]. Together, these results demonstrate that systematic variation of solvent and base can substantially tune the CO release kinetics of formate‐based surrogates, providing chemists with practical levers for reaction optimization.

Switching from chloroform to aprotic solvents also substantially increased the surrogate score (Figure 16C) [51]. Chloroform, a hydrogen bond donor, can attenuate the nucleophilicity of the base through solvent–base interactions, thereby impeding attack on the formate carbonyl [64]. In aprotic solvents, this interaction is absent, resulting in faster CO release. The superior performance of DMF (0.88) over toluene (0.69) is consistent with stabilization of the developing charges at the E2 transition state by the polar aprotic environment (Figure 16A), in accord with mechanistic studies by Konishi et al. [63]. Overall, solvent selection enabled a near‐tripling of the surrogate score relative to chloroform (0.38).

The larger standard deviations observed for DMF and chloroform relative to toluene (Figure 16C) reflect distinct sources of replicate variability in each solvent. For chloroform (bp 61 °C), differential solvent evaporation between replicates under the sealed COware conditions introduces variability in both the effective concentration and the headspace composition. For DMF, the higher viscosity and surface tension lead to less reproducible vortex formation during stirring, which affects the rate of CO dissolution and consequently the hue–time profile. Toluene, with intermediate volatility and lower viscosity, provided the most reproducible conditions across replicates.

### 3.7 Migratory Insertion

**FIGURE 17:**
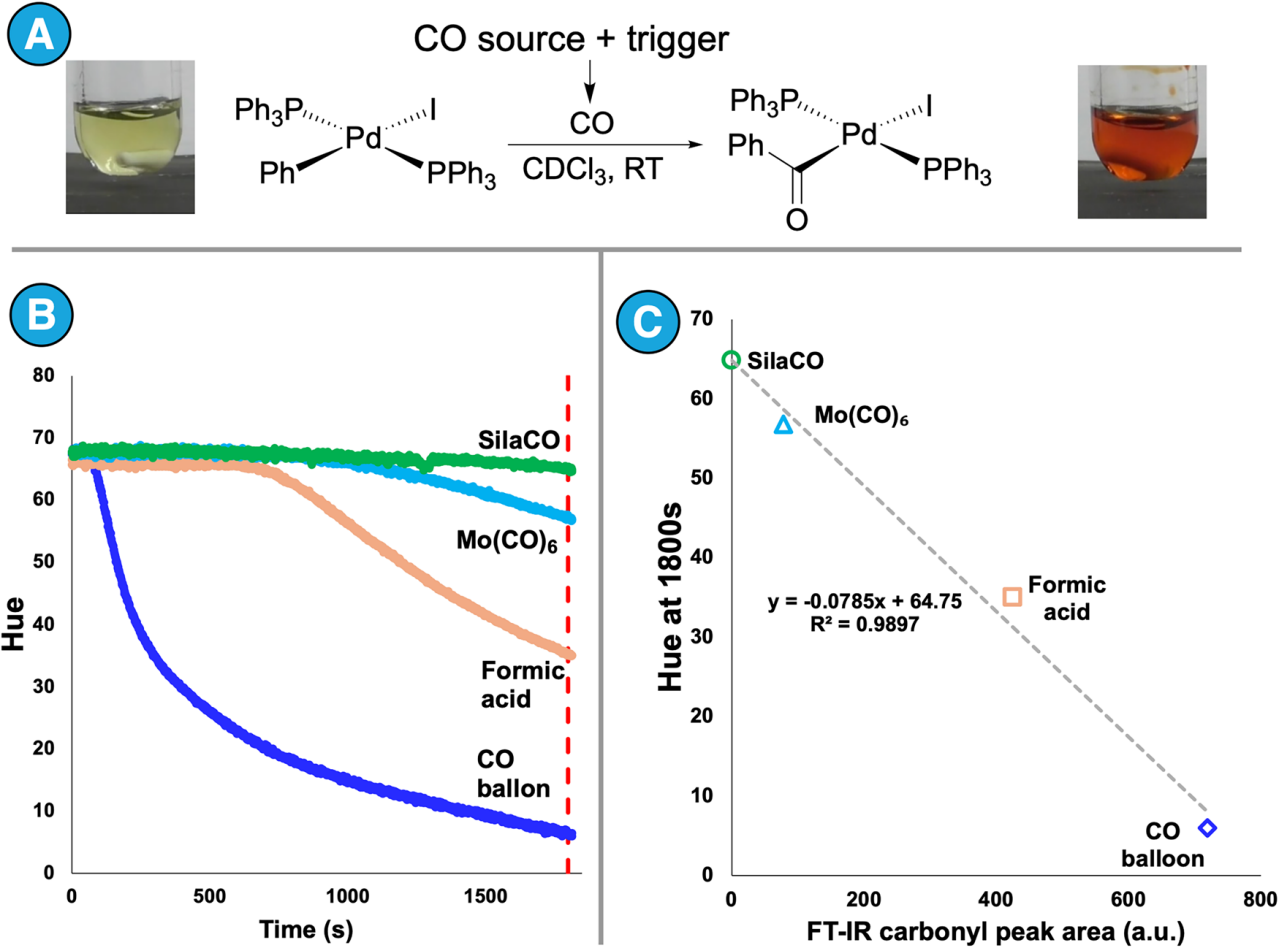
(A) Reaction scheme and associated color change for the CO insertion into [(${\mathrm{Ph}}_{3}P$)
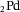
(Ph)(I)]. (B) Plot of Hue versus time obtained from *Kineticolor* analysis of the reaction. (C) Plot of FT‐IR carbonyl peak area versus the final hue value detected from the reaction bulk in Chamber B for migratory insertion using the selected CO sources.

We moved toward investigating the value of our quantitative understanding of CO surrogate reactivity in metal‐catalyzed carbonylation, beginning with an isolated study on migratory insertion (Figure 17). To do so, we selected three surrogates for study based on their position on our score of cumulative flux: formic acid (one of the more reactive surrogates), Mo(CO)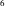 (which demonstrated intermediate reactivity and is commonly used in two‐chamber carbonylation chemistry), and SilaCO (one of the slower‐releasing surrogates).

As in previous experiments, we established a benchmark against which to evaluate each surrogate. The migratory insertion reaction was first conducted using the CO balloon method. When exposed to CO gas, the [(${\mathrm{Ph}}_{3}P$)
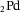
(Ph)(I)] solution transitioned from pale yellow (hue = 70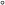) to deep red (hue = 6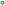) (Figure 17A). FT‐IR spectroscopy confirmed that this bulk color change was consistent with insertion of the CO into the Pd─$C_{\text{Ph}}$ bond and formation of a benzoyl palladium species [65]. Analysis of the crude reaction mixture from Chamber B showed the appearance of a characteristic Pd─C(O)R peak at 1660 ${\mathrm{cm}}^{-1}$.

*Kineticolor* analysis revealed distinct hue profiles for each chosen surrogate, with formic acid (the most reactive surrogate), reaching a final hue value closest to that of the CO balloon benchmark (Table 2). Mo(CO)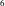 continued to demonstrate intermediate reactivity, consistent with our quantitative scoring of cumulative CO flux. Notably, within the 1800 s (30 min) reaction time, no carbonyl peak was detected in samples exposed to CO produced from SilaCO (Table 2 entry 4). From the concentration‐calibrated CO surrogate scoring metrics (Table 1, entry 2), it is worth noting that SilaCO exhibited the longest induction period (8 min) before reaching the point of maximum rate of CO uptake in Chamber B. This induction period is 27% of the 1800 s (30 min) reaction time used for the comparative migratory insertion reactions, and forms the likely explanation for the observed minimal change in hue and the absence of a detectable CO peak in the FT‐IR spectra for the SilaCO‐mediated reaction.

**TABLE 2:** Each of the CO sources used for the migratory insertion reaction, with the corresponding final hue value detected by *Kineticolor* and carbonyl peak area determined using FT‐IR.

CO source	${\mathrm{Hue}}_{final}$	C=O peak area (a.u.)
CO balloon	6.27	730.12
Formic acid	35.14	424.70
Mo(CO) 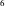	56.73	78.94
SilaCO	64.84	No peak detected

Figure 17C shows a linear relationship between the final hue value detected by *Kineticolor* and the carbonyl peak area detected by FT‐IR, consistent with the measured bulk color of the solution being representative of the major reaction of interest. These encouraging data set the stage for the final part of our study, applying our new reaction monitoring approach to a Pd‐catalyzed reaction using COware.

### 3.8 Carbonylative Suzuki–Miyaura Coupling

**FIGURE 18:**
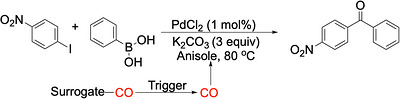
Reaction scheme for the Suzuki–Miyaura coupling of 1‐iodo‐4‐nitrobenzene and phenyl boronic acid to yield (4‐nitrophenyl)(phenyl)methanone. CO surrogates used were formic acid, SilaCO, Mo(CO)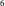, and 18‐C‐6 modified SilaCO.

To investigate the use of our new understanding of CO surrogate reactivity in a synthetically relevant application, we chose to focus on an example of an air‐tolerant carbonylative Suzuki–Miyaura coupling that was published by Ahlburg et al. [18]. Throughout their work, COgen (the least reactive surrogate used in our study) was used as the CO source. We investigated whether the surrogate score and its associated CO release kinetics impact the production of the target ketone, (4‐nitrophenyl)(phenyl)methanone (Figure 18).

In the CO‐releasing Chamber A, we employed formic acid, SilaCO, Mo(CO)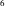 and the 18‐C‐6 modified SilaCO system (Figures 10 and 14). When using the different sources of CO in Chamber A, there was a detectable variation in the conversion of starting material, 1‐iodo‐4‐nitrobenzene, to the target ketone. Each surrogate was used on a scale that produced the same theoretical volume of gas as the original COgen experiments. The CO release rate from each surrogate had an impact on the conversion to the target ketone (Table 3). The highest HPLC yield was observed when using SilaCO as the source of CO, at an average of 61%. The lowest yield was seen using the more reactive SilaCO/KF/18‐C‐6 system at 32%.

**TABLE 3:** Chosen surrogates used in Chamber A, their scores, and the conversion of 1‐iodo‐4‐nitrobenzene to the target ketone as determined by HPLC.

Surrogate	Yield(%)^a^, ^b^	Surrogate score
SilaCO+KF+18‐C‐6	32	0.80
Formic acid	36	0.71
Mo(CO) 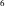	50	0.42
SilaCO	61	0.28

a Determined by HPLC, with acetophenone as the internal standard.

b Average conversion of three repeats, see Section S9 for full details.

**FIGURE 19:**
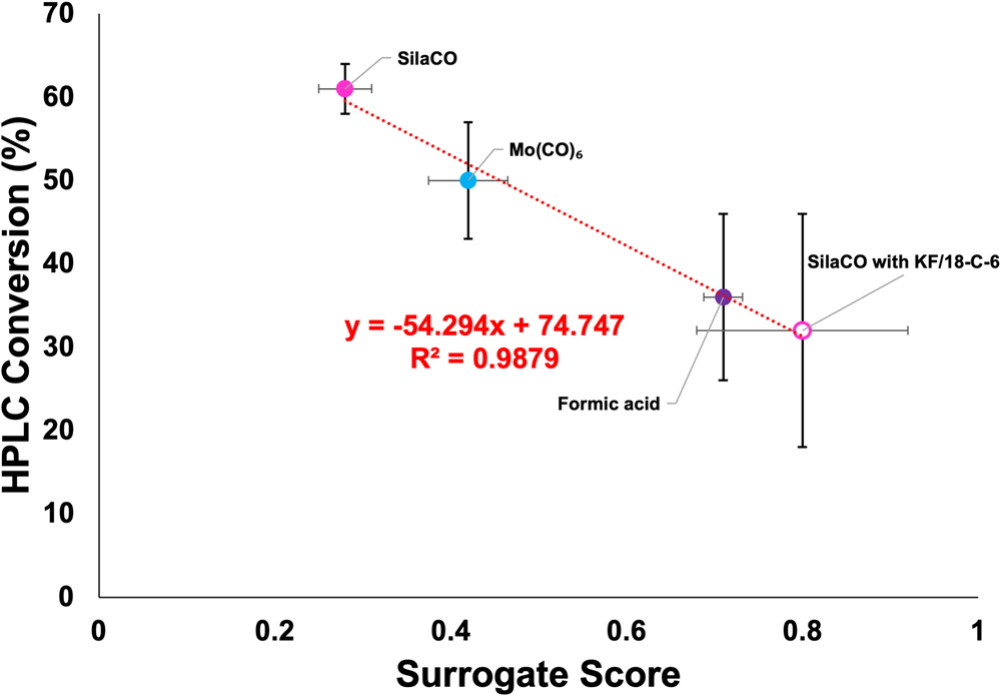
Reported HPLC conversion (%) to target ketone in Chamber B, where four different surrogate systems were used as a source of CO, plotted against the corresponding surrogate score for each surrogate system used in Chamber A. Error bars represent standard deviation from n=3 independent measurements.

Notably, when plotting the computer vision‐derived surrogate score of cumulative flux against the HPLC yield (%) of the target ketone, we observed a linear trend (Figure 19). The replicate variability (reflected in the error bars in Figure 19) is attributed to minor differences in COware sealing efficiency between assemblies, subtle variations in stirring reproducibility, and the inherent sensitivity of the Pd‐catalyzed coupling to local CO concentration gradients within Chamber B. Overall, these data were consistent with the carbonylative Suzuki–Miyaura coupling performing better when there is a slower release of CO from the surrogate. The trend is consistent with an inverse reaction order in [CO], and has been noted for Pd‐catalyzed carbonyl chemistry previously [66, 67]. This inverse dependence is consistent with competitive inhibition of the catalytic cycle by CO at high local concentrations, wherein rapid CO flux saturates palladium coordination sites, disfavoring transmetalation relative to unproductive pathways. Future kinetic modeling incorporating CO dissolution and mass transfer dynamics may further elucidate this selectivity‐rate trade‐off. Together, these data demonstrate that vessel‐specific quantitative understanding of CO surrogate reactivity could enable more judicious optimization of reactions conducted in COware.

### 3.9 Using Surrogate Scores to Guide Reaction Design

The surrogate scoring framework is not intended to rank CO surrogates as universally “better” or “worse,” but rather to enable rational matching of a CO source to the kinetic demands of a given transformation.

For reactions exhibiting an inverse dependence on CO concentration, where excess CO inhibits productive turnover by stabilizing off‐cycle metal carbonyl species or suppressing oxidative addition [68], a lower‐scoring surrogate that delivers CO gradually is preferred. This avoids mimicking the inhibitory effects of a pressurized CO atmosphere while maintaining sufficient carbonylation competency. The Suzuki–Miyaura results presented above (Figure 19) exemplify this regime.

Conversely, transformations requiring high and sustained CO availability, such as the Pauson–Khand reaction [69] or stoichiometric migratory insertion (Figure 17), benefit from higher‐scoring surrogates capable of rapid CO delivery to maintain the productive catalytic manifold.

**FIGURE 20:**
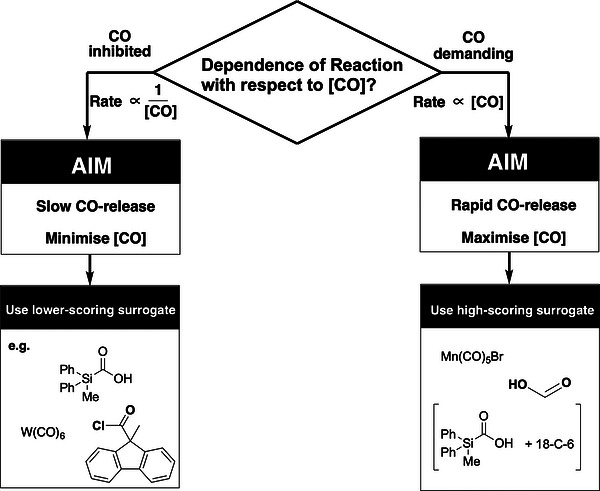
Decision chart illustrating when lower‐ versus higher‐scoring CO surrogates are expected to be optimal, based on the balance between CO inhibition and CO demand in the target reaction.

Figure 20 summarizes these considerations in a decision framework to guide practitioners toward appropriate surrogate choices based on mechanistic insight rather than trial and error.

## 4 Conclusion

We have developed a non‐contact computer vision method for monitoring vessel‐specific CO release from a range of structurally diverse CO surrogates for the first time. The combination of *Kineticolor* software, COware, and a CO sensor was used to place the chosen surrogates on a comparable scale to inform the chemist of which surrogate is best suited for their purpose. Additionally, it was possible to analyze how altering reaction conditions affected the CO production from each surrogate.

Furthermore, we have demonstrated that quantitative monitoring of CO surrogate reactivity enables predictive understanding of carbonylation outcomes in Chamber B. It was seen that a surrogate like SilaCO, which had a score of 0.28 (showing that it is unlike a CO balloon in its gas release), did not release enough CO to drive the migratory insertion reaction, resulting in no acyl product being detected by IR in the allotted time. Conversely, the more reactive CO surrogate, formic acid with a score of 0.72, was able to produce a detectable amount of the product similar to that produced when the insertion reaction was carried out using a CO balloon.

The results presented from the air‐tolerant Suzuki–Miyaura carbonylation reaction further demonstrate that the choice of surrogate can significantly impact the outcome of a reaction. SilaCO, out of the four surrogate systems chosen, was able to produce the highest yield of the target ketone product (61%). By contrast, when the CO release from this surrogate was increased using the KF/18‐C‐6 trigger system, the yield of the target ketone was decreased (32%). The findings from this investigation would suggest that this class of carbonylation reaction shows an inverse reaction order relative to the CO concentration; however, more work should be carried out to confirm this statement.

With regard to limitations, the present method is restricted to colorimetrically responsive systems and does not directly measure CO partial pressure or dissolved concentration. Future work could include extension of these methods to non‐visible gas‐phase kinetics using cameras that incorporate imaging sensors compatible with non‐visible signatures of reaction progress.

Overall this research highlights the value of using computer vision monitoring methods in synthetic chemistry to obtain non‐contact kinetic data, which has previously not been easily accessible.

## Conflicts of Interest

M.R. is the inventor of *Kineticolor* and ileading the software commercialization process. For information on licensing *Kineticolor* software, please contact the corresponding author and the University of Strathclyde technology transfer office: marc.reid.100@strath.ac.uk; info@kineticolor.org; iprmanager@strath.ac.uk.

## Supporting information

Beyond the summarized methods and characterization data in the main supporting information PDF, machine‐readable data for *Kineticolor* analysis, gas pressure measurements, HPLC, calibration data, and Python‐coded analysis scripts are available on figshare at: https://doi.org/10.6084/m9.figshare.31253947.

## Data Availability

The data that support the findings of this study are openly available in figshare at https://doi.org/10.6084/m9.figshare.31253947.v2, reference number DOI: 10.6084/m9.figshare.31253947.v2.
