# Supplementary material for: Computer Vision Enables Monitoring and Kinetic Analysis of Structurally Diverse Carbon Monoxide Surrogates
Source: Angew Chem Int Ed Engl. 2026 May 21;65(29):e8888536. doi: 10.1002/anie.8888536 (PMC13360326; doi:10.1002/anie.8888536)
Supplement: Supplementary file 1 — Beyond the summarized methods and characterization data in the main supporting information PDF, machine‐readable data for Kineticolor analysis, gas pressure measurements, HPLC, calibration data, and Python‐coded analysis scripts are available on figshare at: https://doi.org/10.6084/m9.figshare.31253947. [file ANIE-65-e8888536-s001.pdf]

Computer Vision Enables Monitoring and Kinetic Analysis of Structurally Diverse Carbon Monoxide Surrogates

Kristin Donnachie, Ciaran Griffin, Morven L. Gray, Timothy J.D. McCabe, and Marc Reid

Department of Pure and Applied Chemistry, University of Strathclyde, Glasgow G1 1XL, U.K.
marc.reid.100@strath.ac.uk

Brief guide to S.I. contents:

- **Section 1**: Full experimental procedures for sensor synthesis, COware operation, and video acquisition.
- **Section 2**: Evaluation and rejection of alternative CO chemosensors.
- **Section 3**: Raw Kineticolor outputs and model fitting used to derive surrogate scores.
- **Section 4**: Hue–concentration calibration methodology and limitations.
- **Section 5**: Effect of reaction variables on CO release kinetics.
- **Sections 6–10**: Full spectroscopic and analytical validation (NMR, FT-IR, UV-Vis, HPLC).

In addition to the summarized methods and data below, machine-readable data for *Kineticolor* analysis, HPLC, calibration data, and Python-coded analysis scripts are available on figshare at:

https://doi.org/10.6084/m9.figshare.31253947

Table of Contents

1. Experimental 5

1.1. Synthesis of $Rh_2(OAc)_2(HOAc)_2(PPh_2\text{-}C_6H_4)_2$ 5

1.2. Kineticolor software and data availability..... 6

1.3. CO Surrogate investigation using $Rh_2(OAc)_2(HOAc)_2(PPh_2\text{-}C_6H_4)_2$..... 6

1.3.1. General Procedure A 6

1.3.2. Formic acid as a CO surrogate using $Rh_2(OAc)_2(HOAc)_2(PPh_2\text{-}C_6H_4)_2$ as a sensor 7

1.3.3. Molybdenum hexacarbonyl ($Mo(CO)_6$) as a CO surrogate $Rh_2(OAc)_2(HOAc)_2(PPh_2\text{-}C_6H_4)_2$ as a sensor 8

1.4. Synthesis of $[Ru(CH{=}CHPyr\text{-}1)Cl(CO)(BTD)(PPh_3)_2]$ 8

1.5. CO Surrogate investigation using $[Ru(CH{=}CHPyr\text{-}1)Cl(CO)(BTD)(PPh_3)_2]$. 9

1.5.1. General Procedure B..... 9

1.5.2. Group 6 hexacarbonyls as a CO surrogate along with $[Ru(CH{=}CHPyr\text{-}1)Cl(CO)(BTD)(PPh_3)_2]$ as the CO chemosensor..... 9

1.5.3. Formic acid as a CO surrogate using $[Ru(CH{=}CHPyr\text{-}1)Cl(CO)(BTD)(PPh_3)_2]$ as the CO chemosensor..... 10

1.5.4. Bromopentacarbonyl manganese (I) as a CO surrogate using $[Ru(CH{=}CHPyr\text{-}1)Cl(CO)(BTD)(PPh_3)_2]$ as the CO chemosensor 10

1.5.5. *N*-Formylsaccharin as a CO surrogate using $[Ru(CH{=}CHPyr\text{-}1)Cl(CO)(BTD)(PPh_3)_2]$ as the CO chemosensor..... 10

1.5.7. Methyldiphenylsilacarboxylic acid (SilaCO) as a CO surrogate using $[Ru(CH{=}CHPyr\text{-}1)Cl(CO)(BTD)(PPh_3)_2]$ as the CO chemosensor 11

1.5.9. 2,4,6 trichlorophenyl formate as a CO surrogate using $[Ru(CH{=}CHPyr\text{-}1)Cl(CO)(BTD)(PPh_3)_2]$ as the CO chemosensor..... 12

1.5.10. $[Ru(CH{=}CHPyr\text{-}1)Cl(CO)(BTD)(PPh_3)_2]$ response to CO_2 gas 12

1.5.11. $[Ru(CH{=}CHPyr\text{-}1)Cl(CO)(BTD)(PPh_3)_2\ ]$ response to HCl gas..... 12

1.6. Gas Pressure Measurements 13

1.6.1. Gas pressure measurements of CO production from transition metal carbonyls 13

1.6.2. Gas pressure measurement of CO production from formic acid..... 13

1.6.3. Gas pressure measurement of CO production of CO from SilaCO..... 14

1.6.4. Gas pressure measurement of CO production from *N*-formylsaccharin 14

1.7. Variable Study 15

1.7.1. Stirring rate 16

1.7.2. Base Variation 16

1.7.3. SilaCO with 18-Crown-6 16

1.7.4. COgen with $Pd_2(dba)_3$ 16
1.8. Migratory Insertion 17
1.8.1. Synthesis of $Ph(I)Pd(PPh_3)_2$ 17
1.8.2. General Procedure C 18
1.8.3. Migratory insertion into $Ph(I)Pd(PPh_3)_2$ using CO balloon 18
1.8.4. Migratory insertion into $Ph(I)Pd(PPh_3)_2$ using Formic Acid 18
1.8.5. Migratory insertion into $Ph(I)Pd(PPh_3)_2$ using $Mo(CO)_6$ 19
1.8.6. Migratory insertion into $Ph(I)Pd(PPh_3)_2$ using SilaCO 19
1.9. Air-Tolerant Carbonylative Suzuki-Miyaura Coupling 19
1.9.1. Synthesis of (4-Nitrophenyl)(phenyl)methanone using SilaCO as the source of CO 19
1.9.2. Synthesis of (4-Nitrophenyl)(phenyl)methanone using $Mo(CO)_6$ as the source of CO 20
1.9.3. Synthesis of (4-Nitrophenyl)(phenyl)methanone using Formic acid as the source of CO 20
1.9.4. Synthesis of (4-Nitrophenyl)(phenyl)methanone using SilaCO and KF/18-c-6 trigger system as the source of CO 20
1.10. Video hue Calibration 21
1.10.1. Isolation of $[Ru(CH{=}CHPyr\text{-}1)Cl(CO)_2(PPh_3)_2]$ (Complex 2) 21
1.10.2. Collection of calibration data 21
2. Alternative CO Colorimetric sensors 22
2.1. $[FeCl_2(PNP\text{-}^iPr)]$ colorimetric sensor 22
2.2. $PdCl_2$ colorimetric sensor 23
2.3. CO surrogate investigation using $Rh_2(OAc)_2(HOAc)_2(PPh_2\text{-}C_6H_4)_2$ 24
3. CO surrogate investigation using $[Ru(CH{=}CHPyr\text{-}1)Cl(CO)(BTD)(PPh_3)_2]$. .. 26
3.1. [Ru] sensor response to other gases 26
3.2.1. Surrogate Score 29
3.2.2. Kineticolor data 29
3.2.3. Weibull curve fitting data 34
4. Calibration of color and concentration 50
4.1. Calibration details 50
4.2. Normalized Kineticolor data for calibration studies 56
5. Impact of reaction conditions on CO release 61
5.1. Surrogate Scores 61
5.2. Kineticolor data 62
5.3. Weibull Data 65

6. NMR 77
7. FTIR 86
8. UV-Vis 88
9. HPLC 89
10. References 103

1. Experimental

All reagents were used as supplied unless stated otherwise. Rhodium (II) acetate dimer was supplied by Fluorochem, the glacial acetic acid was supplied by Alfa Aesar. PPh_3 and anisole were supplied by Sigma-Aldrich.

Tris(triphenylphosphine)rhodium(I) carbonyl hydride was supplied by Sigma-Aldrich, 2,1,3-benothiadiazole, 1-ethynylpyrene, iodobenzene (98%), 1-iodo-4-nitrobenzene (99%) and phenylboronic acid (98+ %) were supplied by Thermoscientific, DCE was supplied by Fisher Scientific. Formic acid, 1,4-Dioxane, $Mn(CO)_5Br$ were all supplied by Alfa Aesar, N-Formylsaccharin was supplied by Apollo Science. Sulfuric acid, chloroform and silica 60A, 40-63 micron were supplied by Fisher Scientific, *N,N*-Dimethylformamide was supplied by Rathburn. Oxalyl chloride, silaCOgen, 18-Crown-6 ether, $Mo(CO)_6$, 1,8-Diazabicyclo[5.4.0]undec-7-ene, $W(CO)_6$ was supplied by Sigma-Aldrich, potassium fluoride and $Cr(CO)_6$ was supplied by Thermoscientific.

NMR spectra were obtained at ambient temperature using a Bruker 400 or 500 MHz spectrometer. IR spectra were acquired using an Agilent 5500 FT-IR spectrometer with diamond ATR.

Where replicate measurements are reported in this study, three fully independent experiments (i.e. n=3) were used to calculated mean and standard deviation values. No stock solutions were used. Fresh surrogate and sensor solutions were prepared for all repeat experiments.

1.1. Synthesis of $Rh_2(OAc)_2(HOAc)_2(PPh_2\text{-}C_6H_4)_2$

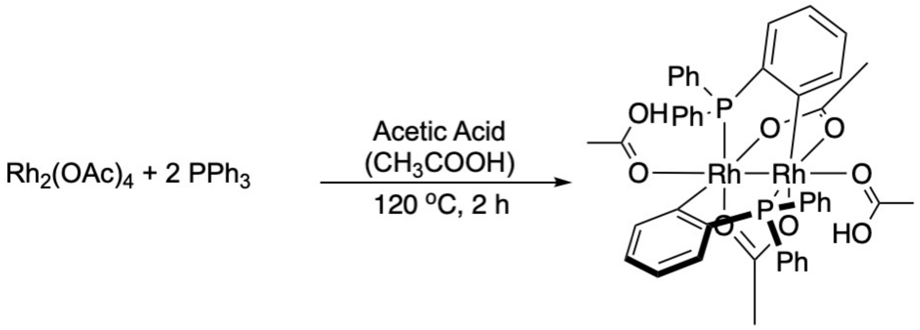

Figure S1: Synthesis of $Rh_2(OAc)_2(HOAc)_2(PPh_2\text{-}C_6H_4)_2$. [1]

Prior to the beginning of the synthesis, several reagent preparations were carried out. To degas the glacial acetic acid, argon was bubbled through the acid for 10 minutes. Furthermore, all triphenylphosphine used was recrystallized using hot ethanol to remove any potential triphenylphosphine oxide that may be present.

$Rh_2(OAc)_4$ (60 mg, 0.136 mmol, 1 equiv.) was dissolved in degassed glacial acetic acid (6 mL) in a Schleck tube fitted with a 1.5 x 8mm stirrer bar. Recrystallised triphenylphosphine (72 mg, 0.258 mmol, 2 equiv.) was added to the tube. This addition resulted in the original blue/green solution turning brown. Once the inert atmosphere was established, the solution was refluxed at 120 °C for 2 hours under an argon atmosphere. The reaction volume was then reduced by half under vacuum, and the resulting purple solid was filtered and dried under vacuum overnight (57.4 mg, 43.8%).

^{1}H NMR (500 MHz, $CDCl_3$, 298 K) δ(ppm): 7.5-6.4 (m, 28 H), 2.1 (s, 6 H), 1.1 (s, 6H)

^{31}P NMR (500 MHz, $CDCl_3$, 298 K) δ(ppm): 19.6

ATR-FTIR ($Rh_2(OAc)_2(HOAc)_2(PPh_2\text{-}C_6H_4)_2$) ν\cm^{-1}: 3057, 3031, 1675, 1583, 1563, 1483, 1434, 1390, 1296, 1093, 736, 689

1.2. Kineticolor software and data availability.

Kineticolor is a computer-vision software platform developed in our laboratory for the extraction of quantitative, time-resolved information from video recordings of visible chemical processes. In the present work, the software was used to analyze video footage of the sensor solution in Chamber B of COware reactors by defining a user-selected region of interest and extracting frame-by-frame color information as numerical time series. Raw pixel data are captured in RGB space and algorithmically converted into alternative color representations (including HSV and CIE L*a*b*), enabling metrics such as hue to be plotted directly as a function of time. The output of each analysis is a machine-readable spreadsheet file containing the full time series used for all subsequent data processing, modelling, and figure generation.
To ensure transparency and reproducibility independent of access to the software itself, all machine-readable Kineticolor output files underlying the figures and analyses reported in this study are provided as part of the Supporting Information in a publicly accessible figshare repository, organized according to figure and table numbering in the main text. These files enable full re-analysis, re-plotting, and independent interrogation of the data using standard data-analysis tools. At the time of publication, the Kineticolor software is not yet released openly to the academic community; this is declared as a competing interest in line with our previous publications.
To enquire about software licensing, please email marc.reid.100@strath.ac.uk, iprmanager@strath.ac.uk, and visit www.kineticolor.org.

1.3. CO Surrogate investigation using $Rh_2(OAc)_2(HOAc)_2(PPh_2\text{-}C_6H_4)_2$

1.3.1. General Procedure A

CO surrogate experiments were carried out in SyTrack COware gas reactor (20 mL) inside of a GODOX LSD80 LED mini light box. 3 LED panels were used within the lightbox, one on the left, one on the Right and one along the front (9300 Lux). Every reaction was filmed using a Panasonic HC-W580 camcorder at 780p at a 12x zoom. The recordings were filmed at 25 FPS and analyzed using the Kineticolor 'Standard' tab with a frame skip of 400 (i.e. analyzing every 400th frame). A frame-skip of 400 was selected to reduce data redundancy while preserving temporal resolution relative to gas-phase kinetics.

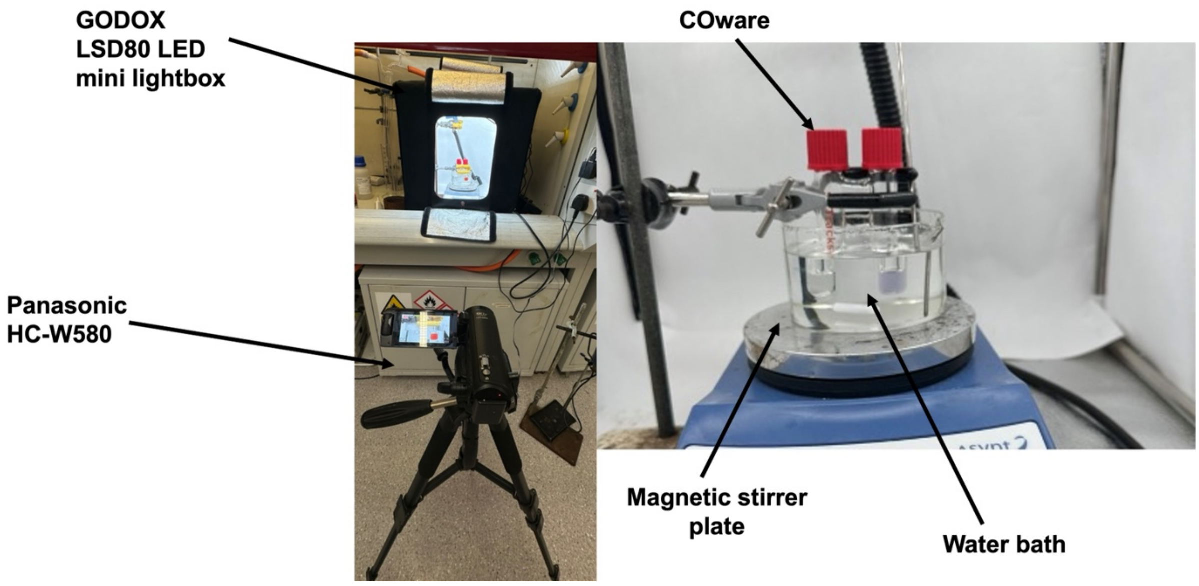

Figure S2: Standard equipment set up used during surrogate investigation.

Additional Note on Hardware and Recording Setup

For researchers adopting this technique, we recommend:

(i) a camera capable of manual white balance and exposure lock to prevent automatic adjustments during recording;

(ii) diffuse, consistent lighting to minimize shadows, reflections, and daylight influences;

(iii) a fixed camera mount to ensure the region of interest remains static throughout the experiment; and

(iv) a recording frame rate of at least 1 frame per second, which provides sufficient temporal resolution for the CO release timescales studied here (typically 30–50 min).

1.3.2. Formic acid as a CO surrogate using $Rh_2(OAc)_2(HOAc)_2(PPh_2\text{-}C_6H_4)_2$ as a sensor

Following details from **general procedure A.**

A 2.12 M stock solution of 98%+ formic acid in deionized water was made. In chamber A, 18 M sulfuric acid (1.5 mL) was added. Both chambers were mixed at 300 RPM using a magnetic stirrer bar (1.5 x 8 mm) in each chamber. Into chamber B of a COware reactor, $Rh_2(OAc)_2(HOAc)_2(PPh_2\text{-}C_6H_4)_2$ (2 mg, 0.002 mmol, 1 equiv.) was dissolved in DCE (1.5 mL) and fitted with a gas-tight seal. Both chambers were heated to 25 ºC in a water bath and allowed to equilibrate for approximately 5 minutes. Once the video recording was started, 48.8 μL, of a 2.12 M aqueous formic

acid solution was added into chamber A and then the chamber was fitted with a gas-tight seal. The reaction was recorded for approx. 50 minutes.

1.3.3. Molybdenum hexacarbonyl ($Mo(CO)_6$) as a CO surrogate $Rh_2(OAc)_2(HOAc)_2(PPh_2\text{-}C_6H_4)_2$ as a sensor

Following details from **general procedure A.**

Into chamber A, $Mo(CO)_6$ (13.6 mg, 0.052 mmol) was dissolved in 1,4-dioxane (1.5 mL). Into chamber B of a COware reactor, $Rh_2(OAc)_2(HOAc)_2(PPh_2\text{-}C_6H_4)_2$ (2 mg, 0.002 mmol, 1 equiv.) was dissolved in DCE (1.5 mL) and fitted with a gas-tight seal. Both chambers were mixed at 300 RPM using a magnetic stirrer bar (1.5 x 8 mm) in each chamber. Both chambers were heated to 25 ºC in a water bath and allowed to equilibrate for approximately 5 minutes. The video recording was then started and DBU, 50 μL, was added to chamber A, which was then quickly fitted with a gas-tight seal. The reaction was recorded for approx. 50 minutes.

1.3.4. Chromium hexacarbonyl ($Cr(CO)_6$) as a CO surrogate using $Rh_2(OAc)_2(HOAc)_2(PPh_2\text{-}C_6H_4)_2$ as a sensor

Following details from **general procedure A.**

Into chamber A, $Cr(CO)_6$ (13.6 mg, 0.052 mmol) was dissolved in 1,4-dioxane (1.5 mL). Into chamber B of a COware reactor, $Rh_2(OAc)_2(HOAc)_2(PPh_2\text{-}C_6H_4)_2$ (2 mg, 0.002 mmol, 1 equiv.) was dissolved in DCE (1.5 mL) and fitted with a gas-tight seal. Both chambers were mixed at 300 RPM using a magnetic stirrer bar (1.5 x 8 mm) in each chamber. Both chambers were heated to 25 ºC in a water bath and allowed to equilibrate for approximately 5 minutes. The video recording was then started and DBU, 50 μL, was added to chamber A, which was then quickly fitted with a gas-tight seal. The reaction was filmed for approx. 50 minutes.

1.4. Synthesis of $[Ru(CH{=}CHPyr\text{-}1)Cl(CO)(BTD)(PPh_3)_2]$

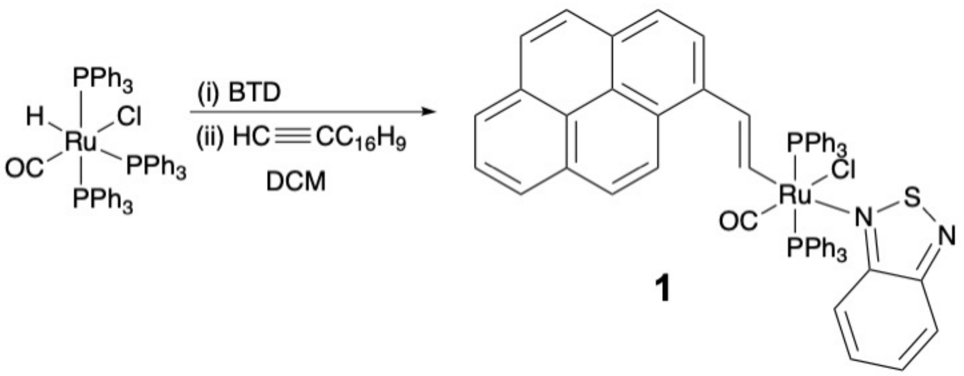

Figure S3: Synthesis of [Ru(CH=CHpyr-1)Cl(CO)(BTD)(PPh₃)₂.[2]

$[RuHCl(CO)(PPh_3)_3]$ (101.8 mg, 0.107 mmol) was dissolved into DCM (10 mL) in a Schleck tube, then 2,1,3-Benzothiadiazole (BTD) (25 mg, 0.184 mmol) was added to the solution. The resulting reaction mixture was stirred until the solution turned from

dark green to dark orange. Once this change had occurred, 1-ethynylpyrene (36.5 mg, 0.161 mmol) was added and the reaction mixture was stirred at RT for 1 hour. After this allotted time, mixing was stopped, and methanol (25 mL) was added to the reaction flask and left for 30 minutes. The reaction volume was then reduced under vacuum and cooled to –20 ^{o}C. Resulting in the formation of a red-orange precipitate which was then filtered and washed with ethanol (2 x 10 mL) and then dried under vacuum overnight. (182 mg, 80.6%)

^{1}H NMR (500 MHz, $CDCl_3$, 298 K) δ(ppm): 8.99 (d, 1H, RuCH=C), 7.98 (m, 2H, BTD), 7.93-7.54 (m, 1H, pyrenyl & BTD), 7.49-7.41 (m, 16H, PPh_3), 7.23-7.16 (m, 14H, PPh_3), 6.90 (d, 1H, RuC=CH)

^{31}P NMR (500 MHz, $CDCl_3$, 298 K) δ(ppm): 27.15

ATR-FTIR of [Ru(CH=CHPyr-1)Cl(CO)(BTD)$(PPh_3)_2$] in DCE ν/cm^{-1}: 1928, 1483, 1432, 1086, 832, 739, 693

1.5. CO Surrogate investigation using [Ru(CH=CHPyr-1)Cl(CO)(BTD)$(PPh_3)_2$]

1.5.1. General Procedure B

Each of the CO surrogate experiments were carried out in SyTrack COware gas reactor (20 mL) inside of a GODOX LSD80 LED mini light box. Within the box, three LED panels were used. One on the left side, right side and front of the box. These panels were all connected to the GODOX LSC3 power supply and set to full intensity (9300 lux). Each reaction was filmed using a Panasonic HC-W580 camcorder at 780p and x12 zoom. The recordings were analyzed using the Kineticolor to extract average pixel coloration within the selected region of interest, analyzing every 400th frame.

1.5.2. Group 6 hexacarbonyls as a CO surrogate along with [Ru(CH=CHPyr-1)Cl(CO)(BTD)$(PPh_3)_2$] as the CO chemosensor

Following details from **general procedure B.**

In chamber A, the chosen group six hexacarbonyl (Table S1) was dissolved in 1,4-dioxane (1.5 mL). In chamber B, [Ru(CH=CHPyr-1)Cl(CO)(BTD)$(PPh_3)_2$] (4 mg, 0.004 mmol) was dissolved in DCE. Both chambers were mixed at 400 RPM using a magnetic stirrer bar (1.5 x 8 mm) in each chamber. Both chambers were heated to 25 ºC in a water bath and allowed to equilibrate for approximately 5 minutes. The video recording was started and DBU, 50 μL, was added to chamber A and then the chamber was quickly fitted with a gas-tight seal. The reaction was recorded for approximately one hour.

Table S1: Reaction details for procedure 1.5.2.

$M(CO)_6$	No. of mmol required	Mass Required (mg)
Cr	0.047	10.4
Mo	0.047	12.5
W	0.047	16.6

1.5.3. Formic acid as a CO surrogate using [Ru(CH=CHPyr-1)Cl(CO)(BTD)(PPh_3)$_2$] as the CO chemosensor

Following details from **general procedure B.**

In chamber A, 18 M sulfuric acid (1.5 mL) was added. In chamber B, [Ru(CH=CHPyr-1)Cl(CO)(BTD)(PPh_3)$_2$] (4 mg, 0.004 mmol) was dissolved in DCE. Both chambers were mixed at 400 RPM using a magnetic stirrer bar (1.5 x 8 mm) in each chamber. Both chambers were heated to 25 ºC in a water bath and allowed to equilibrate for approximately 5 minutes. The video recording was started and 44.6 μL, of a 2.12 M aqueous formic acid solution was added to chamber A, and the chamber was quickly fitted with a tight gas seal. The reaction was recorded for approximately one hour.

1.5.4. Bromopentacarbonyl manganese (I) as a CO surrogate using [Ru(CH=CHPyr-1)Cl(CO)(BTD)(PPh_3)$_2$] as the CO chemosensor

Following details from **general procedure B.**

In chamber A, $Mn(CO)_5Br$ was dissolved in chloroform (1.5 mL). In chamber B, [Ru(CH=CHPyr-1)Cl(CO)(BTD)(PPh_3)$_2$] (4 mg, 0.004 mmol) was dissolved in DCE. Both chambers were mixed at 400 RPM using a magnetic stirrer bar (1.5 x 8 mm) in each chamber. Both chambers were heated to 25 ºC in a water bath and allowed to equilibrate for approximately 5 minutes. The video recording was started and pyridine, 42 μL, was added to chamber A and then the chamber was quickly fitted with a gas-tight seal. The reaction was recorded for approximately one hour.

1.5.5. *N*-Formylsaccharin as a CO surrogate using [Ru(CH=CHPyr-1)Cl(CO)(BTD)(PPh_3)$_2$] as the CO chemosensor

Following details from **general procedure B.**

In chamber A, Na_2CO_3 (22.1 mg, 0.208 mmol) was suspended in DMF (1 mL). In chamber B, Ru(CH=CHPyr-1)Cl(CO)(BTD)(PPh_3)$_2$ (4 mg, 0.004 mmol) was dissolved in DCE. Both chambers were mixed at 400 RPM using a magnetic stirrer bar (1.5 x 8 mm) in each chamber. Both chambers were heated to 25 ºC in a water bath and allowed to equilibrate for approximately 5 minutes. *N*-formylsaccharin (20 mg, 0.95 mmol) was dissolved into DMF (0.5 mL). The video recording was started

and *N*-formylsaccharin solution was added to chamber A. The reaction was recorded for approximately one hour.

1.5.6. Oxalyl chloride as a CO surrogate using [Ru(CH=CHPyr-1)Cl(CO)(BTD)(PPh_3)$_2$] as the CO chemosensor

Following details from **general procedure B.**

A 2M solution of oxalyl chloride in dichloromethane was made.

In chamber A, 1 M sodium hydroxide solution (1.5 mL) was added. In chamber B, [Ru(CH=CHPyr-1)Cl(CO)(BTD)(PPh_3)$_2$] (4 mg, 0.004 mmol) was dissolved in DCE. Both chambers were mixed at 400 RPM using a magnetic stirrer bar (1.5 x 8 mm) in each chamber. Both chambers were heated to 25 ºC in a water bath and allowed to equilibrate for approximately 5 minutes. The video recording was started and oxalyl chloride solution, 96.4 μL, was added to chamber A. The reaction was recorded for approximately one hour.

1.5.7. Methyldiphenylsilacarboxylic acid (SilaCO) as a CO surrogate using [Ru(CH=CHPyr-1)Cl(CO)(BTD)(PPh_3)$_2$] as the CO chemosensor

Following details from **general procedure B.**

In chamber A, SilaCO (22.9 mg, 0.95 mmol) was dissolved in 1,4-dioxane (1 mL). In chamber B of the 20 mL COware, [Ru(CH=CHPyr-1)Cl(CO)(BTD)(PPh_3)$_2$] (4 mg, 0.004 mmol) was dissolved in DCE. Both chambers were fitted with a gas-tight seal, set to stir using a magnetic stirrer bar (8 x 1.5 mm) at 400 RPM and placed into a 25 ºC water bath and allowed to equilibrate. The video recording was started and potassium fluoride (6 mg, 0.104 mmol) suspended in 1,4 dioxane (0.5 mL) was added to chamber A and then the chamber was quickly fitted with a gas-tight seal. The reaction was recorded for approximately one hour.

1.5.8. 9-methylfluorene-9-carbonyl chloride (COgen) as a CO surrogate using [Ru(CH=CHPyr-1)Cl(CO)(BTD)(PPh_3)$_2$] as the CO chemosensor

In chamber A, COgen (23 mg, 0.095 mmol), Tri-tert-butylphosphonium tetrafluoroborate (P(^{t}Bu)H_3BF_4) (1.38 mg, 0.0047 mmol) and Bis(dibenzylideneacetone)palladium(0) (Pd(dba)$_2$) (2.73 mg, 0.047 mmol) were dissolved in anisole (1.5 mL). In chamber B of the 20 mL COware, [Ru(CH=CHPyr-1)Cl(CO)(BTD)(PPh_3)$_2$] (4 mg, 0.004 mmol) was dissolved in DCE. Both chambers were fitted with a gas-tight seal, set to stir using a magnetic stirrer bar (8 x 1.5 mm) at 400 RPM and placed into a 25 ºC water bath and allowed to equilibrate. The video recording was started and *N,N*-Diisopropylethylamine (DIPEA) (33 μL, 0.19 mmol) was added to chamber A and then the chamber was quickly fitted with a gas-tight seal. The reaction was recorded for approximately one hour.

1.5.9. 2,4,6 trichlorophenyl formate as a CO surrogate using [Ru(CH=CHPyr-1)Cl(CO)(BTD)(PPh_3)$_2$] as the CO chemosensor

In chamber A, triethylamine (13.1 µL, 0.094 mmol) in chloroform (0.5 mL) was added. In chamber B of the 20 mL COware, [Ru(CH=CHPyr-1)Cl(CO)(BTD)(PPh_3)$_2$] (4 mg, 0.004 mmol) was dissolved in DCE and then fitted with a gas-tight seal. Both chambers were fitted with a gas-tight seal, set to stir using a magnetic stirrer bar (8 x 1.5 mm) at 400 RPM and placed into a 25 ºC water bath and allowed to equilibrate. The video recording was started and 2,4,6-trichlorophenyl formate (21.34 mg, 0.095 mmol) dissolved in chloroform (1 mL) was added to chamber A and then the chamber was quickly fitted with a gas-tight seal. The reaction was recorded for approximately one hour.

1.5.10. [Ru(CH=CHPyr-1)Cl(CO)(BTD)(PPh_3)$_2$] response to CO_2 gas

Following details from **general procedure B.**

In chamber A the COware, sodium bicarbonate (6.25 mg, 0.0325 mmol) and citric acid (16.25 mg, 0.19 mmol) were added. In chamber B, [Ru(CH=CHPyr-1)Cl(CO)(BTD)(PPh_3)$_2$] (4 mg, 0.004 mmol) was dissolved into DCE (1.5 mL). Both chambers were mixed at 400 RPM using a magnetic stirrer bar (1.5 x 8 mm) in each chamber. Both chambers were heated to 25 ºC in a water bath and allowed to equilibrate for approximately 5 minutes. The video recording was started, and water (2 mL) was added into chamber A and then it was quickly fitted with a gas-tight seal. The reaction was recorded for approximately 45 minutes.

1.5.11. [Ru(CH=CHPyr-1)Cl(CO)(BTD)(PPh_3)$_2$] response to HCl gas

Following details from **general procedure B.**

In chamber A of 20 mL COware, calcium chloride (500 mg, 0.003 mol) was added. In chamber B, [Ru(CH=CHPyr-1)Cl(CO)(BTD)(PPh_3)$_2$] (4 mg, 0.004 mmol) was dissolved into DCE (1.5 mL). Both chambers were mixed at 400 RPM using a magnetic stirrer bar (1.5 x 8 mm) in each chamber. Both chambers were heated to 25 ºC in a water bath and allowed to equilibrate for approximately 5 minutes. The recording was started and HCl (2 mL) was added into chamber A. The reaction was recorded for approximately 15 minutes.

1.6. Gas Pressure Measurements

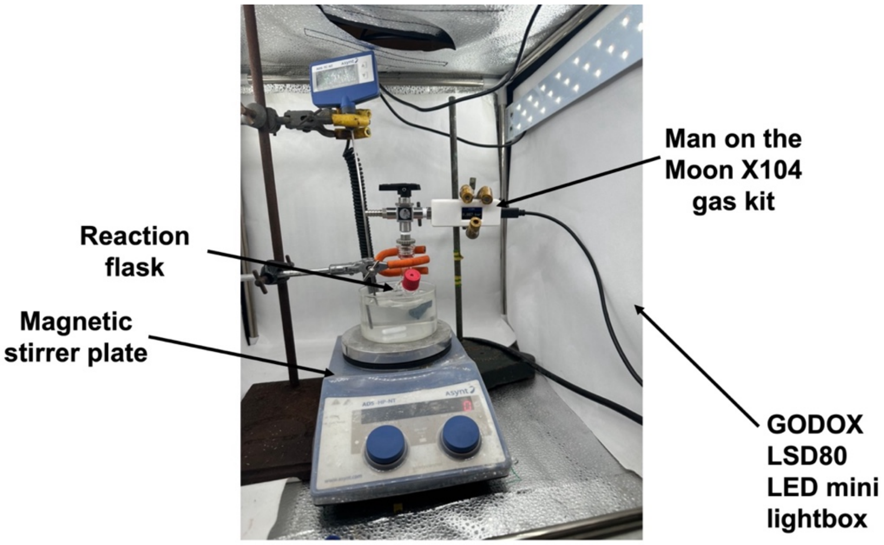

Figure S4: equipment setup used to gather gas presure measurements from surrogate.

1.6.1. Gas pressure measurements of CO production from transition metal carbonyls

Table S2: Reaction condition for section 1.6.1

Entry	CO Surrogate	Mass of Surrogate (mg)	No of mmol	Solvent	Trigger	Volume of Trigger (μL)
1	$Cr(CO)_6$	20.8	0.095	1,4 Dioxane	DBU	100
2	$Mo(CO)_6$	25	0.095	1,4 Dioxane	DBU	100
3	$W(CO)_6$	33.5	0.095	1,4 Dioxane	DBU	100
4	$Mn(CO)_5Br$	26	0.095	Chloroform	Pyridine	84

All gas pressure measurements were carried out using the X104 gas evolution kit supplied by Man on the Moon: https://manonthemoontech.com/kinetics-kits/x104-gas-evolution/.

Within the 15 mL round bottom flask supplied by the company, the chosen transition metal CO surrogate was dissolved into the solvent specified within Table S2 and added to the flask. The reaction vessel was set to stir with a magnetic stirrer bar (1.5 x 8 mm) at 400 RPM and then the round bottom was submerged into a water bath set to 25 °C and allowed to equilibrate. Within the X104_V2BT17 software, the recording was started, and the trigger was added to the round bottom. The reaction was allowed to proceed for approximately one hour before the recording was stopped.

1.6.2. Gas pressure measurement of CO production from formic acid

In the 15 mL round bottom supplied within the gas evolution kit, sulfuric acid was added (1.5 mL). The reaction vessel was set to stir with a small magnetic stirrer bar (1.5 x 8 mm) at 400 RPM and then the round bottom was submerged into a water bath set to 25 °C and allowed to equilibrate. Within the X104_V2BT17 software the recording was started and a 2.12 M aqueous formic acid solution, 89.2 μL, was

added to the round bottom. The reaction was allowed to proceed for approximately one hour.

1.6.3. Gas pressure measurement of CO production of CO from SilaCO

In the 15 mL round bottom supplied within the gas evolution kit, SilaCO (45.8 mg, 0.19 mmol, excess) was dissolved in 1,4 dioxane (1 mL). The reaction vessel was set to stir with a small magnetic stirrer bar (1.5 x 8 mm) at 400 RPM and then the round bottom was submerged into a water bath set to 25 ºC and allowed to equilibrate. Within the X104 V2BT17 software the recording was started, and then KF (12 mg, 0.21 mmol) suspended in 1,4 dioxane (0.5 mL) was added to the round bottom. The reaction was allowed to proceed for approximately one hour.

1.6.4. Gas pressure measurement of CO production from *N*-formylsaccharin

In the 15 mL round bottom supplied within the gas evolution kit, Na_2CO_3 (44.2 mg, 0.416 mmol) was suspended in DMF (1 mL). The reaction vessel was set to stir with a small magnetic stirrer bar (1.5 x 8 mm) at 400 RPM and then the round bottom was submerged into a water bath set to 25 ºC and allowed to equilibrate. Within the X104_V2BT17 software, the recording was started, and then *N*-Formylsaccharin (40 mg, 1.9 mmol) dissolved in DMF (0.5 mL) was added to the round bottom. The reaction was allowed to proceed for approximately one hour.

Table S3: Summary of conversion of surrogate to CO gas determined by the gas pressure measured using the Man on the Moon gas kit.

Surrogate	Conversion (%)
$Mn(CO)_5Br$	237.97
Formic acid	82.32
N-formylsaccharin	43.45
$Cr(CO)_6$	29.75
$Mo(CO)_6$	22.66
$W(CO)_6$	17.00
SilaCOgen	8.41

Apparent conversions exceeding 100% for $Mn(CO)_5Br$ arise from the stoichiometric assumption used in the calculation. The conversion is calculated based on the release of 2 equivalents of CO per molecule of $Mn(CO)_5Br$; however, each molecule bears 5 CO ligands, and under the reaction conditions employed, additional CO ligands beyond the assumed 2 equivalents may be released. Furthermore, evaporation of the volatile solvent ($CHCl_3$, bp 61 ºC) during the experiment may artificially concentrate the analyte in Chamber B, inflating the apparent conversion.

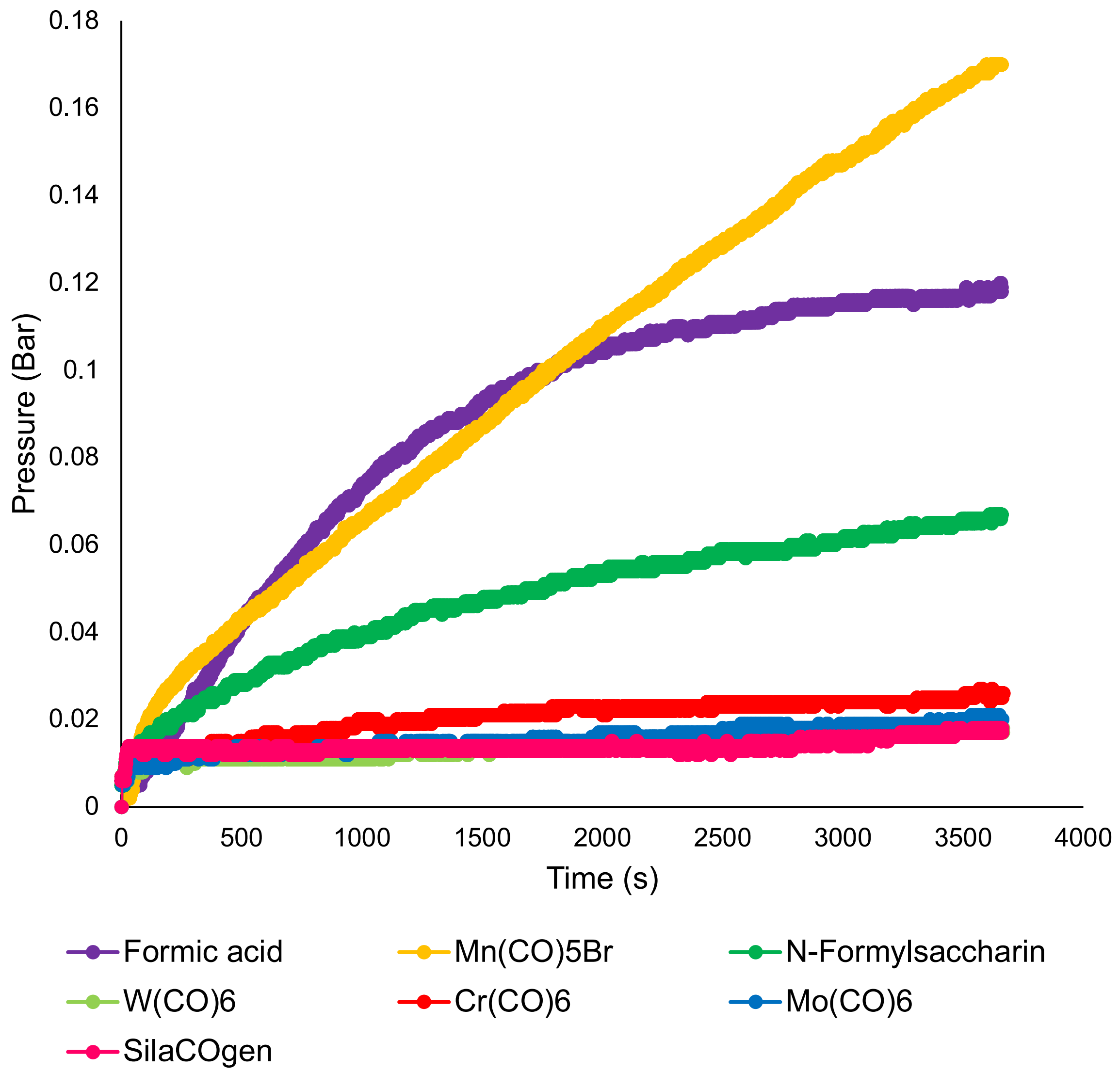

Figure S5: Pressure over time obtained from each surrogate.

1.7. Variable Study

Table S4: Reaction condition for section 1.7.1 and 1.7.2

Entry	No. of mmol of complex 1	Trigger	Volume of trigger (μL)	RPM
1	0.004	DBU	50	400
2	0.004	DBU	50	800
3	0.004	DBU	4.6	400
4	0.008	DBU	4.6	400
5	0.008	DBU	50	400

1.7.1. **Stirring rate**

Following details from **general procedure B.**

In chamber A, $Mo(CO)_6$ (12.5 mg, 0.047 mmol) dissolved in 1,4-dioxane (1.5 mL). In chamber B of COware, $[Ru(CH{=}CHPyr\text{-}1)Cl(CO)(BTD)(PPh_3)_2]$ (4 mg, 0.004 mmol) was dissolved in DCE. Both chambers were mixed at 800 RPM using a magnetic stirrer bar (1.5 x 8 mm) in each chamber. Both chambers were heated to 25 ºC in a water bath and allowed to equilibrate for approximately 5 minutes. The video recording was started and DBU, 50 μL, was added to chamber A and then the chamber was quickly fitted with a gas-tight seal. The reaction was recorded for approximately one hour.

1.7.2. **Base Variation**

Following details from **general procedure B.**

In chamber A, $Mo(CO)_6$ (12.5 mg, 0.047 mmol) dissolved in 1,4-dioxane (1.5 mL). In chamber B of COware, $[Ru(CH{=}CHPyr\text{-}1)Cl(CO)(BTD)(PPh_3)_2]$ (4 mg, 0.004 mmol) was dissolved in DCE. Both chambers were mixed at 400 RPM using a magnetic stirrer bar (1.5 x 8 mm) in each chamber. Both chambers were heated to 25 ºC in a water bath and allowed to equilibrate for approximately 5 minutes. The video recording was started and DBU, 4.6 μL, was added to chamber A and then the chamber was quickly fitted with a gas-tight seal. The reaction was recorded for approximately one hour.

1.7.3. **SilaCO with 18-Crown-6**

Following details from **general procedure B.**

In chamber A, SilaCO (22.9 mg, 0.95 mmol) was dissolved in 1,4-dioxane (1 mL). In chamber B of COware, $[Ru(CH{=}CHPyr\text{-}1)Cl(CO)(BTD)(PPh_3)_2]$ (4 mg, 0.004 mmol) was dissolved in DCE. Both chambers were mixed at 400 RPM using a magnetic stirrer bar (1.5 x 8 mm) in each chamber. Both chambers were heated to 25 ºC in a water bath and allowed to equilibrate for approximately 5 minutes. The video recording was started, KF (6 mg, 0.104 mmol) and 1,4,7,10,13,16-Hexaoxacyclooctadecane (18-crown-6) (13.7 mg, 0.052 mmol) were suspended in 1,4 dioxane (0.5 mL) and then added to chamber two. The reaction was recorded for approximately one hour.

1.7.4. **COgen with $Pd_2(dba)_3$**

In chamber A of the 20 mL COware, $[Ru(CH{=}CHPyr\text{-}1)Cl(CO)(BTD)(PPh_3)_2]$ (4 mg, 0.004 mmol) was dissolved in DCE. In chamber two, COgen (23 mg, 0.095 mmol), Tri-tert-butylphosphonium tetrafluoroborate ($P(^tBu)H_3BF_4$) (1.38 mg, 0.0047 mmol) and Tris(dibenzylideneacetone)dipalladium(0) ($Pd_2(dba)_3$) (4.34 mg, 0.0047 mmol) were dissolved in anisole (1.5 mL). Both chambers were fitted with a gas-tight seal, set to stir using a magnetic stirrer bar (8 x 1.5 mm) at 400 RPM and placed into a 25 ºC water bath and allowed to equilibrate. The video recording was started and *N,N*-

Diisopropylethylamine (DIPEA) (33 μL, 0.19 mmol) was added to chamber two and then the chamber was quickly fitted with a gas-tight seal. The reaction was recorded for approximately one hour.

1.7.5. **2,4,6-trichlorophenyl formate**

All reactions were carried out following **general procedure B** and **procedure 1.5.9.** See Table S5 for details.

Table S5: Reaction condition for section 1.7.5, with changes in reaction conditions highlighted.

Entry	Solvent	Base	Concentration of Base (mmol)
1	Toluene	NEt_3	0.095
2	DMF	NEt_3	0.095
3	Chloroform	DBU	0.095

1.8. Migratory Insertion

1.8.1. **Synthesis of Ph(I)Pd(PPh_3)$_2$**

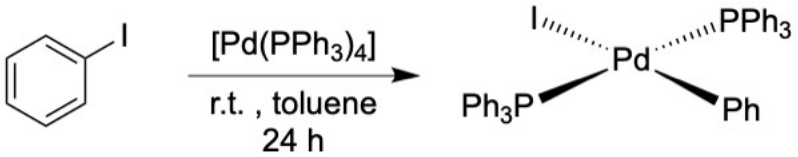

Figure S6: Synthesis of Ph(I)Pd(PPh_3)$_2$.[3]

Into an oven-dried Schlenk tube, iodobenzene (19 mL, 0.17 mmol) and Tetrakis(triphenylphosphine)palladium(0) (Pd(PPh_3)$_4$) (175 mg, 0.15 mmol) was dissolved into dry toluene. Reaction was left to stir (600 RPM) at RT for 24 h. The resulting solution was then cooled to 0 °C to force precipitation. Solid was then filtered and washed with toluene (3 x 1 mL) and pentane (3 x 1mL), then dried under vacuum overnight. Obtained as an off white free flowing powder (89 mg, 71%).

^{1}H NMR (500 MHz, $CDCl_3$,298 K) δ(ppm): 7.56-7.50 (m, 12H), 7.375-7.311 (t, 6H, 15 Hz), 7.29-7.23 (m, apparent 14H; expected 12H but integral is inclusive of residual $CHCl_3$) 6.63 (d, 2H, 7.2 Hz), 6.35 (t, 1H, 15 Hz), 6.24 (t, 2H, 15 Hz)

^{31}P NMR (500 MHz, $CDCl_3$,298 K) δ(ppm): 22.90

ATR-FTIR ν/cm^{-1}: 3064, 1568, 1488, 1474, 1441, 1191, 1103, 1058, 1031, 1022, 998,896, 745, 731, 693, 515, 496

1.8.2. **General Procedure C**

CO surrogate experiments were carried out in SyTrack COware gas reactor (20 mL) inside of a GODOX LSD80 LED mini light box. 3 LED panels were used within the lightbox, one on the left, one on the right and one along the front (9300 Lux). Every reaction was filmed using a Panasonic HC-W580 camcorder at 780p at a 12x zoom. Each recording was analyzed using the Kineticolor to extract average pixel coloration, analyzing every 25th frame. ^{1}H, ^{31}P NMR and an FTIR spectra of each sample were obtained.

1.8.3. **Migratory insertion into Ph(I)Pd(PPh$_3$)$_2$ using CO balloon**

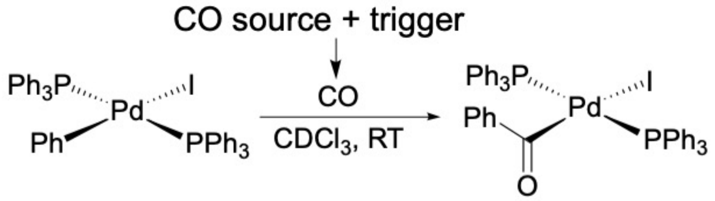

Figure S7: Synthesis of Ph(I)Pd(PPh$_3$)$_3$. [4]

Following details from **general procedure C.**

The process was carried out under an argon atmosphere using Schlenk techniques. Into chamber B, Ph(I)Pd(PPh$_3$)$_2$ (25.1 mg, 0.03 mmol) dissolved into $CDCl_3$ (0.75 mL). Both chambers were sealed with a gas-tight seal, and the reaction was set to stir (400 RPM) using a 1.5 x 8 mm magnetic stirrer. The video recording was started and into chamber A CO was added to the system using a balloon. The reaction was left at RT for 20 minutes, once the reaction was complete contents of chamber B was transferred to an NMR tube along with additional $CDCl_3$ (0.25 mL).

^{31}P NMR (500 MHz, $CDCl_3$, 298 K) δ(ppm): 18.30

ATR-FTIR in $CDCl_3$ ν/cm^{-1}: 3073, 1659, 1490, 1442, 1309, 1188, 1156, 1101, 1004, 915, 868, 732, 964, 629, 521, 494

1.8.4. **Migratory insertion into Ph(I)Pd(PPh$_3$)$_2$ using Formic Acid**

Following details from **general procedure C.**

Process was carried out under an argon atmosphere using Schleck techniques. Into chamber B, Ph(I)Pd(PPh$_3$)$_2$ (25.1 mg, 0.03 mmol) dissolved into $CDCl_3$ (0.75 mL). In chamber A, 18 M sulfuric acid (1.5 mL) was added. Both chambers were mixed at 400 RPM using a magnetic stirrer bar (1.5 x 8 mm) in each chamber. The video recording was started and, 44.6 μL, of a 2.12 M aqueous formic acid solution was added to chamber A and the chamber was quickly fitted with a gas-tight seal. The reaction was left at RT for 20 minutes, once the reaction was complete contents of chamber B was transferred to an NMR tube along with additional $CDCl_3$ (0.25 mL).

1.8.5. **Migratory insertion into Ph(I)Pd(PPh_3)$_2$ using Mo(CO)$_6$**

Following details from **general procedure C.**

Process was carried out under an argon atmosphere using Schleck techniques. Into chamber B, Ph(I)Pd(PPh_3)$_2$ (25.1 mg, 0.03 mmol) dissolved into $CDCl_3$ (0.75 mL). In chamber A, Mo(CO)$_6$(12.5 mg, 0.048 mmol) and 1,4 dioxane (1.5 mL) were added. Both chambers were mixed at 400 RPM using a magnetic stirrer bar (1.5 x 8 mm) in each chamber. The video recording was started and DBU (50 µL) was added to chamber A and the chamber was quickly fitted with a gas-tight seal. The reaction was left at RT for 20 minutes, once the reaction was complete contents of chamber B was transferred to an NMR tube along with additional $CDCl_3$ (0.25 mL).

1.8.6. **Migratory insertion into Ph(I)Pd(PPh_3)$_2$ using SilaCO**

Following details from **general procedure C.**

The process was carried out under an argon atmosphere using Schlenk techniques. Into chamber B, Ph(I)Pd(PPh_3)$_2$ (25.1 mg, 0.03 mmol) dissolved into $CDCl_3$ (0.75 mL). In chamber A, SilaCO (22.8 mg, 0.095 mmol) and 1,4 dioxane (1 mL) were added. Both chambers were mixed at 400 RPM using a magnetic stirrer bar (1.5 x 8 mm) in each chamber. The video recording was started and potassium fluoride (6 mg, 0.104 mmol) suspended in 1,4 dioxane (0.5 mL) was added to chamber two and the chamber was quickly fitted with a gas-tight seal. The reaction was left at RT for 20 minutes, once the reaction was complete contents of chamber B was transferred to an NMR tube along with additional $CDCl_3$ (0.25 mL).

1.9. Air-Tolerant Carbonylative Suzuki-Miyaura Coupling

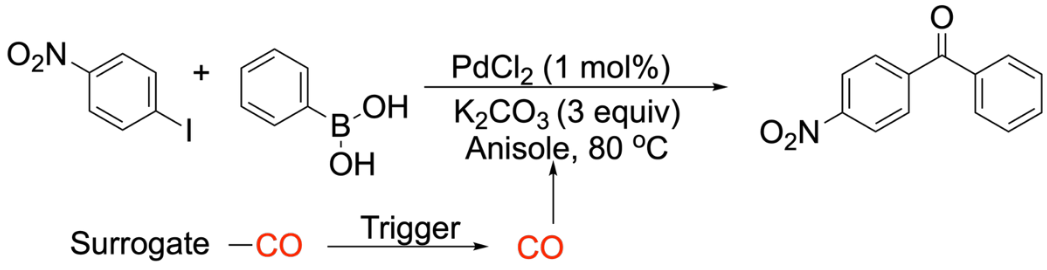

Figure S8:Synthesis of (4-Nitrophenyl)(phenyl)methanone.[3]

1.9.1. **Synthesis of (4-Nitrophenyl)(phenyl)methanone using SilaCO as the source of CO**

The reaction was carried out in a 20 mL SyTrack COware, and each chamber was fitted with a 1.5 x 8 mm magnetic stirrer bar. In chamber B, 1-iodo-4-nitrobenzene (124.5 mg, 0.5 mmol, 1 equiv.), phenylboronic acid (67.1 mg, 0.55 mmol), potassium carbonate (247.8 mg, 1.5 mmol) and $PdCl_2$ (0.89 mg, 0.005 mmol) in anisole (3 mL) was added. Into chamber A, silaCO (181 mg, 0.75 mmol) and KF (47.7 mg, 0.82 mmol) were added. Chambers were set to stir at 600 RPM and fitted with a gas-tight seal. 1,4 dioxane (3 mL) was added to chamber A. The reactor was then heated to

80 °C for 16 hours. The reaction was then cooled to RT before a sample of chamber B was taken for HPLC analysis see **section 9** for details.

^{1}H NMR (400 MHz, $CDCl_3$, 298 K) δ(ppm): 8.37 (d, 2H, J = 8.9 Hz), 7.96 (d, 2H, J = 8.8 Hz), 7.80 (d, 2H, 7.1 Hz), 7.71 - 7.65 (m, 1H), 7.58 - 7.52 (m, 2H)

^{13}C NMR (400 MHz, $CDCl_3$, 298 K) δ(ppm): 194.79, 161.55, 142.90, 133.47, 130.70, 130.10, 128.70, 123.55, 92.93, 77.35, 77.03, 76.71, 55.33

1.9.2. Synthesis of (4-Nitrophenyl)(phenyl)methanone using $Mo(CO)_6$ as the source of CO

The reaction was carried out in a 20 mL SyTrack COware, and each chamber was fitted with a 1.5 x 8 mm magnetic stirrer bar. In chamber B, 1-iodo-4-nitrobenzene (124.5 mg, 0.5 mmol, 1 equiv.), phenylboronic acid (67.1 mg, 0.55 mmol), potassium carbonate (247.8 mg, 1.5 mmol) and $PdCl_2$ (0.89 mg, 0.005 mmol) in anisole (3 mL) was added. In chamber A, $Mo(CO)_6$ (99 mg, 0.38 mmol) and 1,4 dioxane (3 mL) were added. Chambers were set to stir at 600 RPM and fitted with a gas-tight seal. To trigger the CO release, DBU (400 μL) was added to chamber A. The reactor was then heated to 80 °C for 16 hours. The reaction was then cooled to RT before a sample of chamber B was taken for HPLC analysis see **section 9** for details.

1.9.3. Synthesis of (4-Nitrophenyl)(phenyl)methanone using Formic acid as the source of CO

The reaction was carried out in a 20 mL SyTrack COware, and each chamber was fitted with a 1.5 x 8 mm magnetic stirrer bar. In chamber B, 1-iodo-4-nitrobenzene (124.5 mg, 0.5 mmol, 1 equiv.), phenylboronic acid (67.1 mg, 0.55 mmol), potassium carbonate (247.8 mg, 1.5 mmol) and $PdCl_2$ (0.89 mg, 0.005 mmol) in anisole (3 mL) was added. In chamber A, sulfuric acid (3 mL) was added. Chambers were set to stir at 600 RPM and fitted with a gas-tight seal. To trigger the CO release, 2.12 M formic acid solution (354 μL) was added to chamber A. The reactor was then heated to 80 °C for 16 hours. The reaction was then cooled to RT before a sample of chamber B was taken for HPLC analysis. See **section 9** for details.

1.9.4. Synthesis of (4-Nitrophenyl)(phenyl)methanone using SilaCO and KF/18-c-6 trigger system as the source of CO

The reaction was carried out in a 20 mL SyTrack COware, and each chamber was fitted with a 1.5 x 8 mm magnetic stirrer bar. In chamber B, 1-iodo-4-nitrobenzene (124.5 mg, 0.5 mmol, 1 equiv.), phenylboronic acid (67.1 mg, 0.55 mmol), potassium carbonate (247.8 mg, 1.5 mmol) and $PdCl_2$ (0.89 mg, 0.005 mmol) in anisole (3 mL) was added. Into chamber A, silaCO (181 mg, 0.75 mmol), KF (47.7 mg, 0.82 mmol) and 18-C-6 (108.5 mg, were added. Chambers were set to stir at 600 RPM and fitted with a gas-tight seal. 1,4 dioxane (3 mL) was added to chamber A. The reactor was then heated to 80 °C for 16 hours. The reaction was then cooled to RT before a sample of chamber B was taken for HPLC analysis see **section 9** for details.

1.10. Video hue Calibration

1.10.1. **Isolation of [Ru(CH=CHPyr-1)Cl(CO)$_2$(PPh$_3$)$_2$] (Complex 2)**

[Ru(CH=CHPyr-1)Cl(CO)(BTD)(PPh$_3$)$_2$ (91 mg) was dissolved into DCM (130 mL) in a large round bottom flask and set to stir. The solution was then exposed to CO gas from a balloon for 5 minutes. Once the solution changed from orange to yellow, ethanol was added (65 mL). The solid was precipitated by removing solvent under reduced pressure. The resulting yellow solid was filtered and washed using ethanol (4 x 30 mL) and dried using Schleck techniques.

^{13}C NMR (500 MHz, CDCl$_3$,298 K) δ(ppm): 163.76, 134.31, 134.27, 134.22, 134.02, 132.85, 132.66, 132.48, 131.77, 131.09, 130.16,128.77, 128.18, 128.14, 128.10, 127.62, 126.07, 125.84, 125.36, 125.02, 124.87, 124.43, 123.92, 123.59, 123.17, 77.27, 77.02, 76.76

^{1}H NMR (500 MHz, CDCl$_3$,298 K) δ(ppm): 8.03-7.96 (m, 3H), 7.91-7.75 (m, 6H), 7.70-7.64 (m, 11H), 7.5 (d, 1H, 8 Hz), 7.32-7.21 (m, 17H), 7.05 (d,1H, 17 Hz)

^{31}P NMR (500 MHz, CDCl$_3$,298 K) δ(ppm): 23.68

1.10.2. **Collection of calibration data**

A stock solution of 5.33 mM (0.008 mmol/1.5 mL) of complex 1, 2 and BTD in DCE were made. Following the details stated in Table S6, 11 calibration solutions were made.

Each of the calibration solutions placed into one chamber of the SyTrack COware with a 1.5x8 mm stirrer bar. The SyTrack COware gas reactor (20 mL) was placed in a water bath inside of a GODOX LSD80 LED mini light box. Within the box, three LED panels were used. One on the left side, right side and front of the box. These panels were all connected to the GODOX LSC3 power supply and set to full intensity (9300 lux). Each calibration solution was stirred at 400 RPM and filmed using a Panasonic HC-W580 camcorder at 780p and x12 zoom for 15 seconds. The recordings were analyzed using Kineticolor to extract average pixel coloration in the region of interest, analyzing every 25th frame.

Table S6: Concentrations of each component used to collect data for and create the video hue calibration curve.

Vial	Concentration of complex 1 (mM)	Volume of orange stock (mL)	Concentration of complex 2 (mM)	Volume of yellow stock (mL)	Concentration of BTD (mM)	Volume of BTD stock (mL)
**1**	2.7	0.75	0.0	0	0.0	0
**2**	2.4	0.675	0.3	0.075	0.3	0.075
**3**	2.1	0.6	0.5	0.15	0.5	0.15
**4**	1.9	0.525	0.8	0.225	0.8	0.225
**5**	1.6	0.45	1.1	0.3	1.1	0.3
**6**	1.3	0.375	1.3	0.375	1.3	0.375
**7**	1.1	0.3	1.6	0.45	1.6	0.45
**8**	0.8	0.225	1.9	0.525	1.9	0.525
**9**	0.5	0.15	2.1	0.6	2.1	0.6
**10**	0.3	0.075	2.4	0.675	2.4	0.675
**11**	0.0	0	2.7	0.75	2.7	0.75

2. Alternative CO Colorimetric sensors

The initial search for a suitable CO sensor highlighted several potential candidates. These included [$FeCl_2$(PNP-^{i}Pr)],[5] $Rh_2(OAc)_2(HOAc)_2(PPh_2\text{-}C_6H_4)_2$,[1] $PdCl_2$ [6] and [Ru(CH=CHPyr-1)Cl(CO)(BTD)($(PPh_3)_2$)].[2]

2.1. [$FeCl_2$(PNP-^{i}Pr)] colorimetric sensor

[$FeCl_2$(PNP-^{i}Pr)] was reported to undergo two different color changes depending on the on the isomer present. When the CO bonded in a cis fashion the complex would change from yellow to red. If it was the trans isomer, it would be yellow to blue (Figure S9).[5] Attempts to synthesis [$FeCl_2$(PNP-^{i}Pr)] were unsuccessful due to persistent oxidation of the phosphorous group. Alterations to the sensor were made to increase steric bulk around the phosphorus to hinder the oxidation but these were also unsuccessful, so this sensor was ruled out as a viable option for this study.

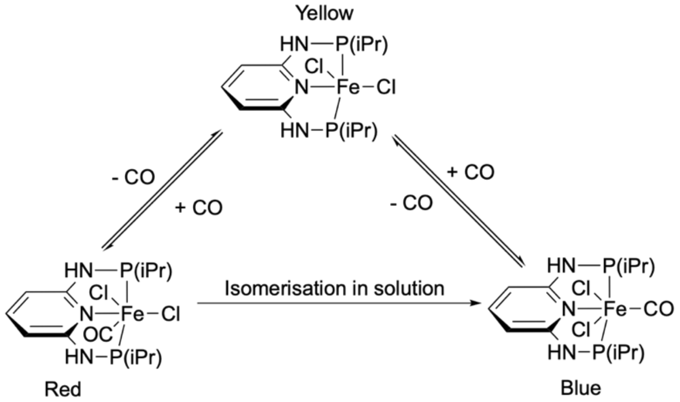

Figure S9: Iron based CO colorimetric sensor.[5]

2.2. $PdCl_2$ colorimetric sensor

$PdCl_2$ was evaluated as a potential CO chemosensor owing to its commercial availability and low cost, which would lower the barrier to adoption for researchers wishing to apply this computer vision methodology. When the solid was dissolved in water and exposed to CO gas from a balloon the solution changed from bright yellow to a dark brown as the Pd is reduced from Pd(II) to Pd(0).[6] The color change is depicted as ΔE, discussed in detail in other publications,[7–12] over time in Figure S10.

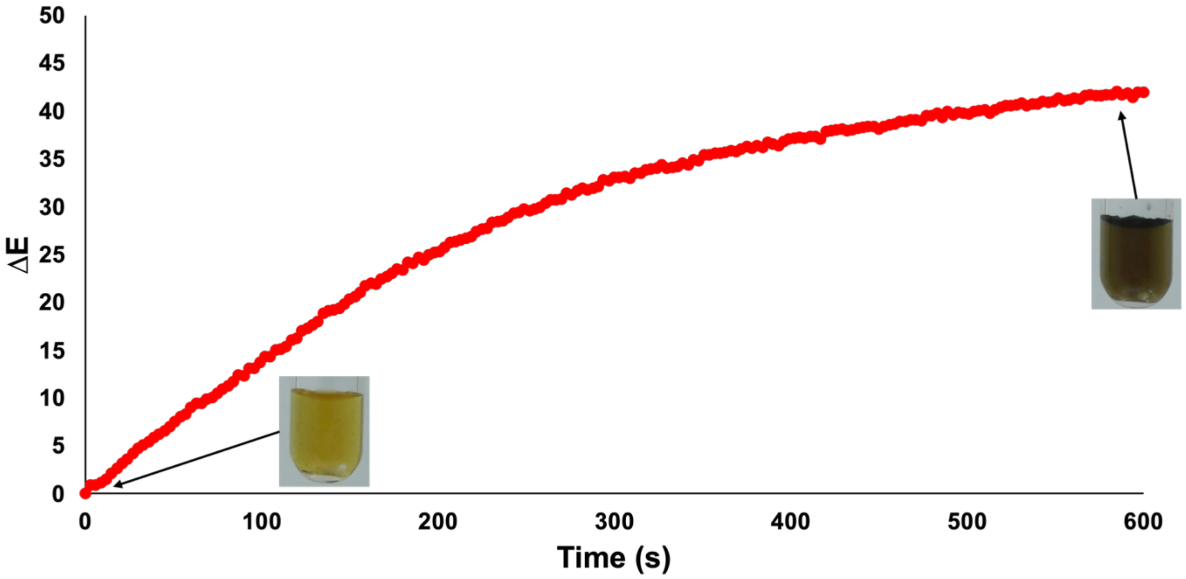

Figure S10: ΔE profile of the color change seen when $PdCl_2$ was exposed to $CO_{(g)}$.

Under the COware conditions employed in this study, $PdCl_2$ exhibited several limitations:

(i) the signal-to-noise ratio of the hue change was substantially lower than that of complex **1**, as the brown-to-black transition spans a narrow and poorly defined region of hue space;

(ii) precipitation of metallic Pd introduced spatial inhomogeneity in the Chamber B solution, complicating extraction of representative hue values from video footage; and

Nonetheless, $PdCl_2$ may offer a viable and more accessible alternative for researchers working with reactor systems that generate higher local CO concentrations (where the lower sensitivity may be less limiting), or for applications where a qualitative rather than quantitative assessment of CO arrival is sufficient. We encourage future investigation of $PdCl_2$ and other commercially available CO-responsive indicators as complementary sensors for the computer vision approach described herein.

2.3. CO surrogate investigation using $Rh_2(OAc)_2(HOAc)_2(PPh_2\text{-}C_6H_4)_2$

Surrogate investigation was started using a [Rh]-based sensor first reported by Wilson *et al.*[1] The sensor undergoes two color changes, purple to orange and then to yellow , when exposed to CO gas because of the displacement of two acetate ligands by CO.

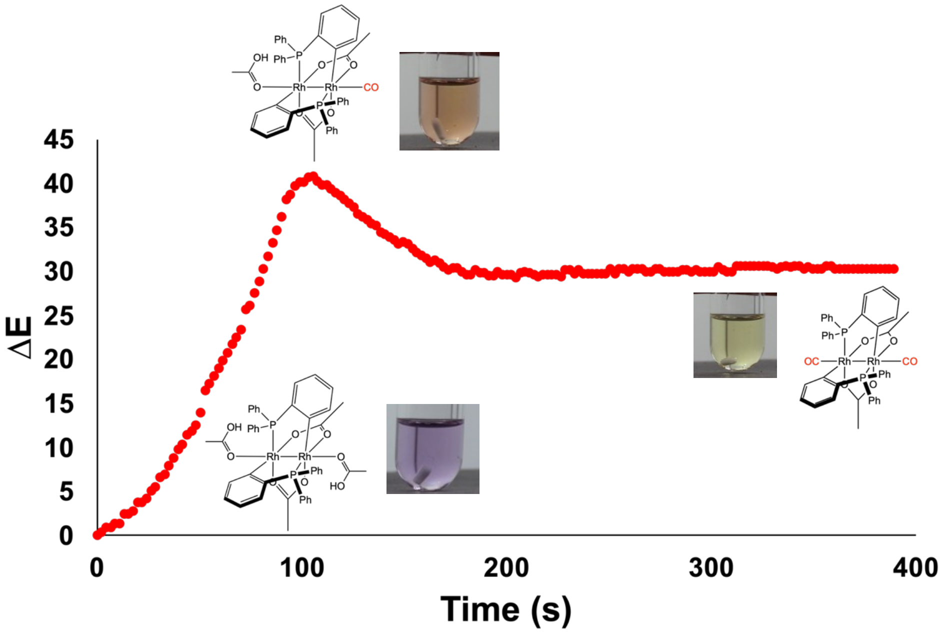

Figure S11: ΔE profile of the color change seen when $Rh_2(OAc)_2(HOAc)_2(PPh_2\text{-}C_6H_4)_2$ was exposed to $CO_{(g)}$

Using this senor the gas release from the following molecules was monitored; chromium hexacarbonyl ($Cr(CO)_6$), molybdenum hexacarbonyl ($Mo(CO)_6$), tungsten hexacarbonyl ($W(CO)_6$), manganese pentacarbonyl bromide ($Mn(CO)_5Br$), *N*-formylsaccharin, formic acid, and oxalyl chloride. Each surrogate was analyzed using Kineticolor to obtain a *ΔE* trace (Figure S11). While this sensor was informative about the CO release from each surrogate it was disregarded, due to several reasons including cost of materials, difficulties experienced during synthesis and complexity of scoring each surrogate. Attempts were made to create a score, however, due to the possibility of two-color changes, this proved to be difficult and less intuitive than the selected ruthenium sensor (Figure S12, Equation 1, Table S7).

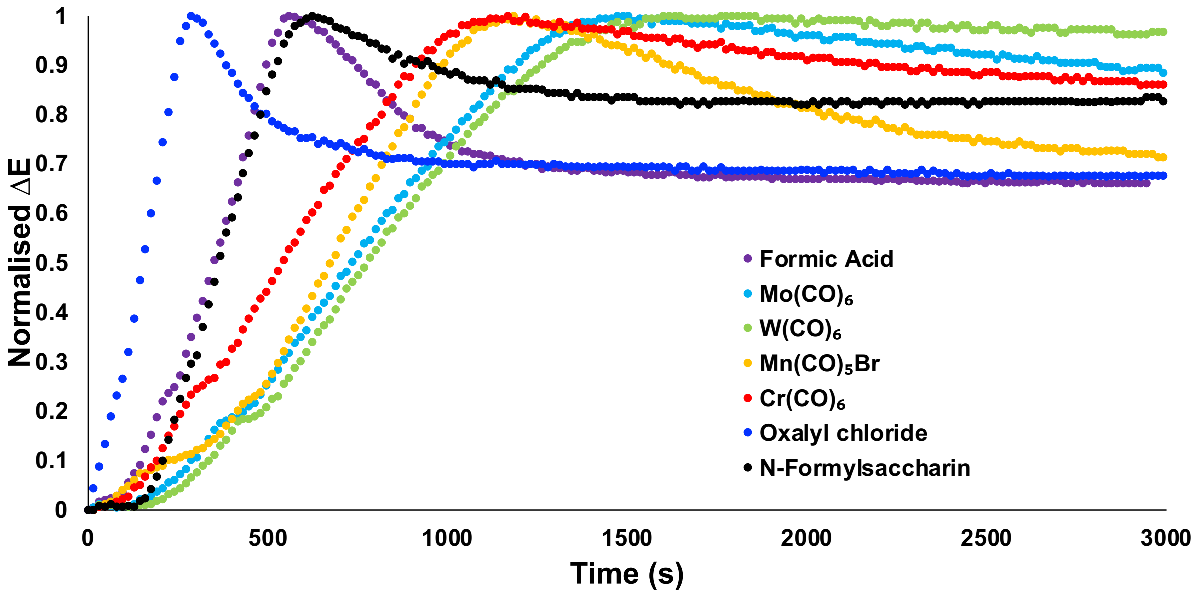

Figure S12: Normalized ΔE trace for the color change seen in the [Rh] based complex when exposed to CO from each surrogate

$$Surrogate\ Flux = \frac{t_p(CO)}{t_p\ (surrogate)} x \frac{\Delta E\ (CO)}{\Delta E\ (surrogate)}$$

Equation S1: Equation used to calculate the surrogate flux of each CO surrogate investigated. t_p (CO)= Time to reach max ΔE for CO balloon, t_p (surrogate)= time for the surrogate to reach max ΔE, ΔE(CO)= value of ΔE at end of set reaction time for CO balloon and ΔE(surrogate)= value of ΔE at end of set reaction time for the surrogate.

Table S7: Data obtained from Kineticolor and the score for each surrogate calculated using Equation 1.

CO Source	t_p (s)	*ΔE* (3000 s)	t_p (CO)/ t_p	*ΔE*(3000s, CO) / *ΔE*(3000s)	Score
**CO balloon**	122	0.64	1.00	1.00	1.00
**Oxalyl chloride**	288	0.7	0.42	0.91	0.39
**Formic acid**	560	0.65	0.22	0.98	0.21
***N*-formylsaccharin**	672	0.85	0.18	0.75	0.14
**$Mn(CO)_5Br$**	1184	0.73	0.10	0.88	0.09
**$Cr(CO)_6$**	1168	0.86	0.10	0.74	0.08
**$Mo(CO)_6$**	1472	0.88	0.08	0.73	0.06
**$W(CO)_6$**	1760	0.97	0.07	0.66	0.05

3. CO surrogate investigation using [Ru(CH=CHPyr-1)Cl(CO)(BTD)(PPh_3)$_2$].

3.1. [Ru] sensor response to other gases

Oxalyl chloride was excluded not due to poor performance, but because it releases multiple reactive gases (CO, CO_2, HCl) that independently modulate the sensor response. This violates the single-analyte assumption required for surrogate scoring.

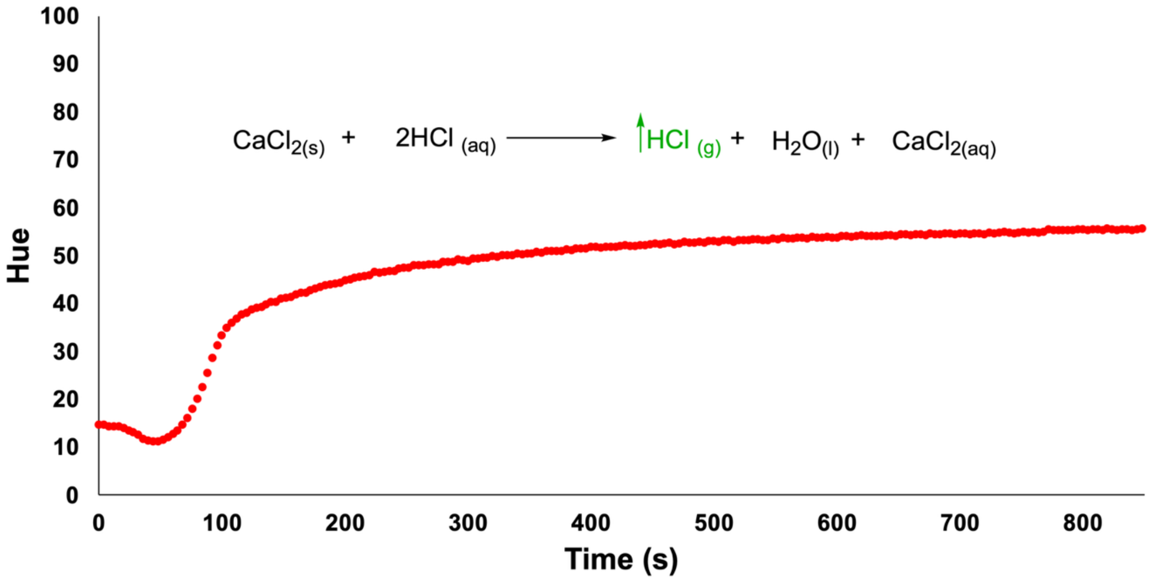

*Figure S13: Change in hue over time when complex **1** was exposed to $HCl_{(g)}$*

The presence of $HCl_{(g)}$ was further confirmed by the use of pH paper in the bridge of the COware. The oxalyl chloride experiment was run and a color change evident in the pH paper was monitored using Kineticolor. Over time ,the pH paper turned from yellow to red, confirming the presence of an acidic gaseous environment within the COware (Figure S14).

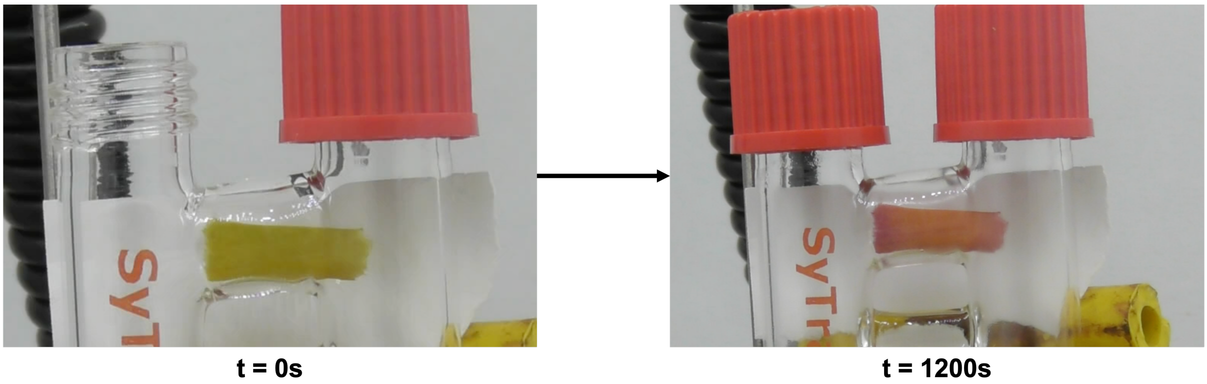

Figure S14: Left pH paper before gas release from oxalyl chloride was triggered. Right pH paper at the end of the reaction after being exposed to gas from oxalyl chloride.

To neutralize the HCl released from oxalyl chloride a higher concentration 2 M sodium hydroxide solution was used. This did not change the rate of change in the sensor or the change in the pH paper (Figure S15 and Figure S16).

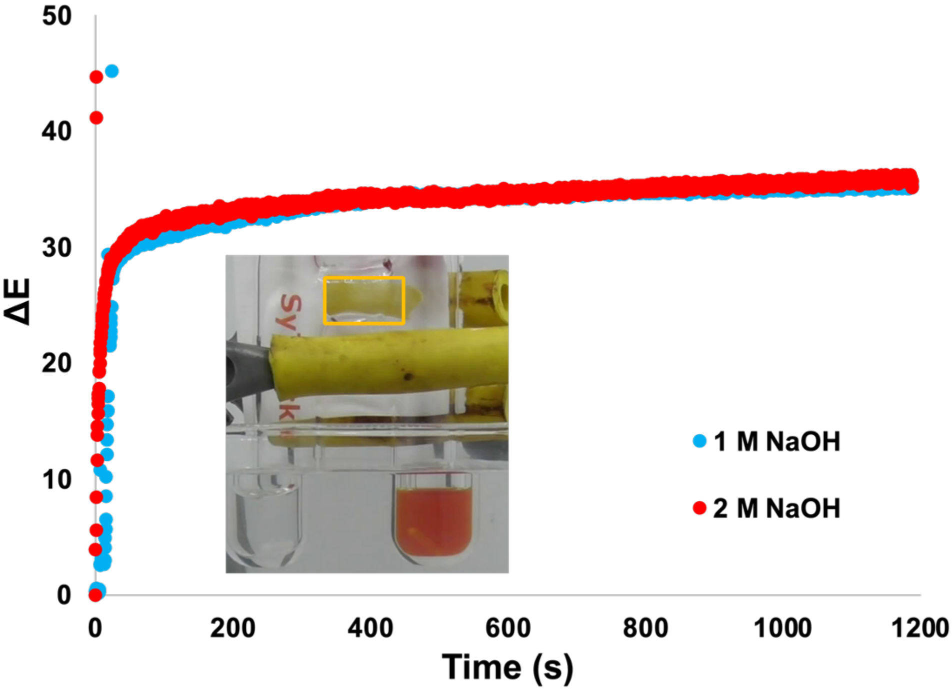

Figure S15: ΔE profile from analysis of pH paper during oxalyl chloride reaction using 1M sodium hydroxide (blue) and 2M sodium hydroxide (red).

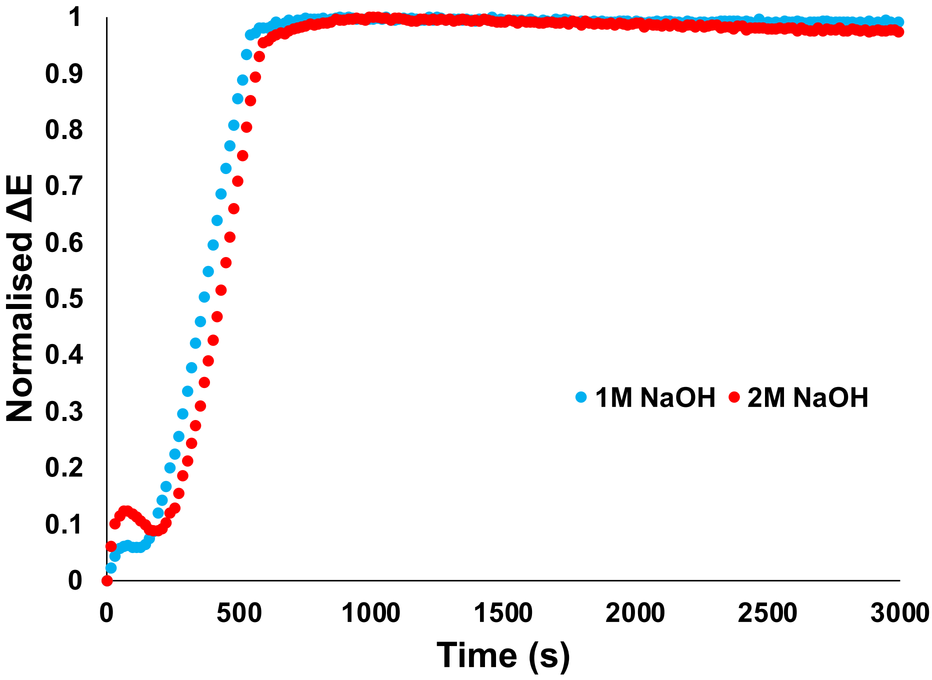

Figure S16: Normalised ΔE profile from analysis of Chamber B during oxalyl chloride reaction using 1M sodium hydroxide (blue) and 2M sodium hydroxide (red). Data was normalized by dividing every data point by the max ΔE to set the values between 0 and 1.

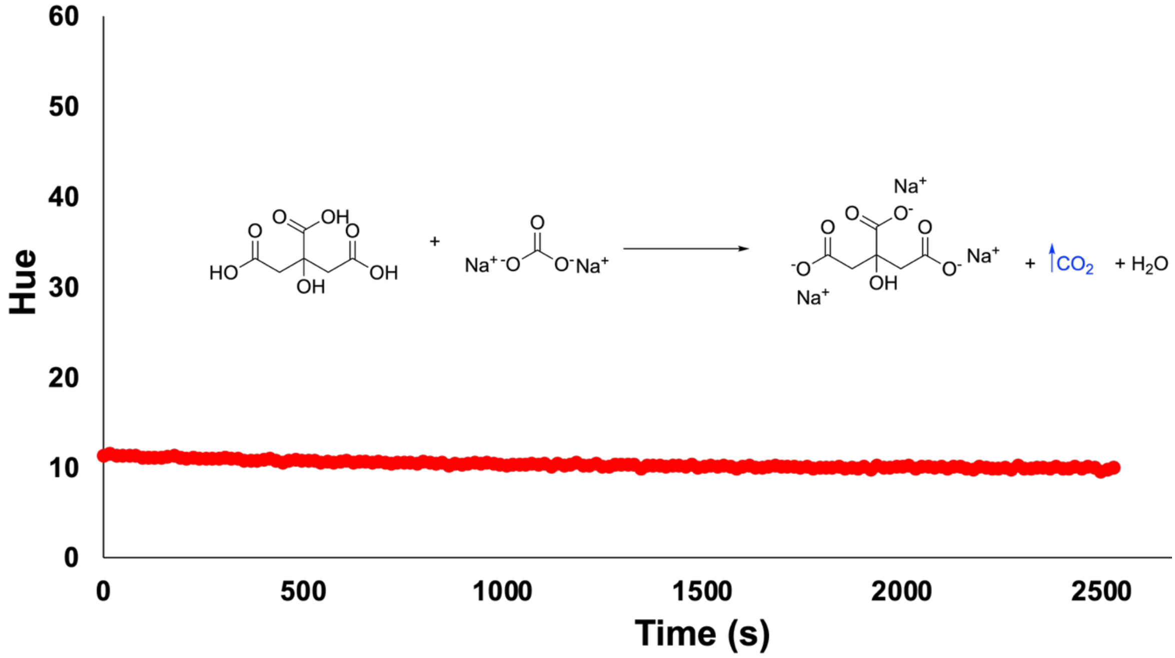

*Figure S17: Change in hue over time when complex **1** was exposed to $CO_{2(g)}$*

3.2.1. Surrogate Score

Table S8: Full details of calculated surrogate score. ***α*** *is defined as the speed component and is a measure of how quickly the surrogate reaches its maximum rate of hue change compared to the benchmark.* ***β*** *is the plateau time component which measures how quickly the surrogate reaches its plateau compared to the benchmark.* ***γ*** *is the magnitude component and measures the total hue change of the surrogate compared to the benchmark.*

Surrogate	File Name	Score A	Std Dev	α	β	γ
**$Mo(CO)_6$**	Color Analysis KD-009E-002	0.49	0.045	0.04	0.03	1.16
	Color Analysis KD-009E-003	0.43		0.04	0.03	1.08
	Color Analysis KD-009E-004	0.41		0.04	0.03	1.07
**$W(CO)_6$**	Color Analysis KD-010E-001	0.37	0.026	0.00	0.00	79.39
	Color Analysis KD-010E-003	0.42		0.03	0.02	1.81
	Color Analysis KD-010E-004	0.38		0.01	0.00	18.22
**$Cr(CO)_6$**	Color Analysis KD-011E-001	0.41	0.052	0.04	0.03	1.02
	Color Analysis KD-011E-002	0.45		0.04	0.03	1.07
	Color Analysis KD-011E-003	0.51		0.04	0.04	1.08
**Formic acid**	Color Analysis KD-012E-002	0.72	0.022	0.07	0.08	1.04
	Color Analysis KD-012E-003	0.73		0.07	0.07	1.09
	Color Analysis KD-012E-004	0.76		0.08	0.08	1.08
**$Mn(CO)_5Br$**	Color Analysis KD-013E-002	0.69	0.019	0.11	0.08	0.99
	Color Analysis KD-013E-003	0.71		0.10	0.08	0.97
	Color Analysis KD-013E-004	0.67		0.08	0.07	0.96
***N*-formylsaccharin**	Color Analysis KD-014E-001	0.47	0.015	0.04	0.03	1.20
	Color Analysis KD-014E-002	0.48		0.05	0.03	1.25
	Color Analysis KD-014E-003	0.50		0.05	0.03	1.20
**SilaCO**	Color Analysis KD-015E-001	0.30	0.030	0.00	0.00	3884.52
	Color Analysis KD-015E-002	0.34		0.03	0.03	1.21
	Color Analysis KD-015E-003	0.28		0.03	0.02	2.03
**COgen**	Color Analysis KD-030E-011	0.54	0.221	0.05	0.05	0.97
	Color Analysis KD-030E-012	0.14		0.03	0.02	0.69
	Color Analysis KD-030E-013	0.18		0.00	0.00	1278.66
**2,4,6 trichlorophenyl formate**	Color Analysis MG E003 006	0.38	0.114	0.04	0.04	1.08
	Color Analysis MG E003 007	0.38		0.04	0.04	1.02
	Color Analysis MG E003 008	0.58		0.05	0.05	1.06

3.2.2. Kineticolor data

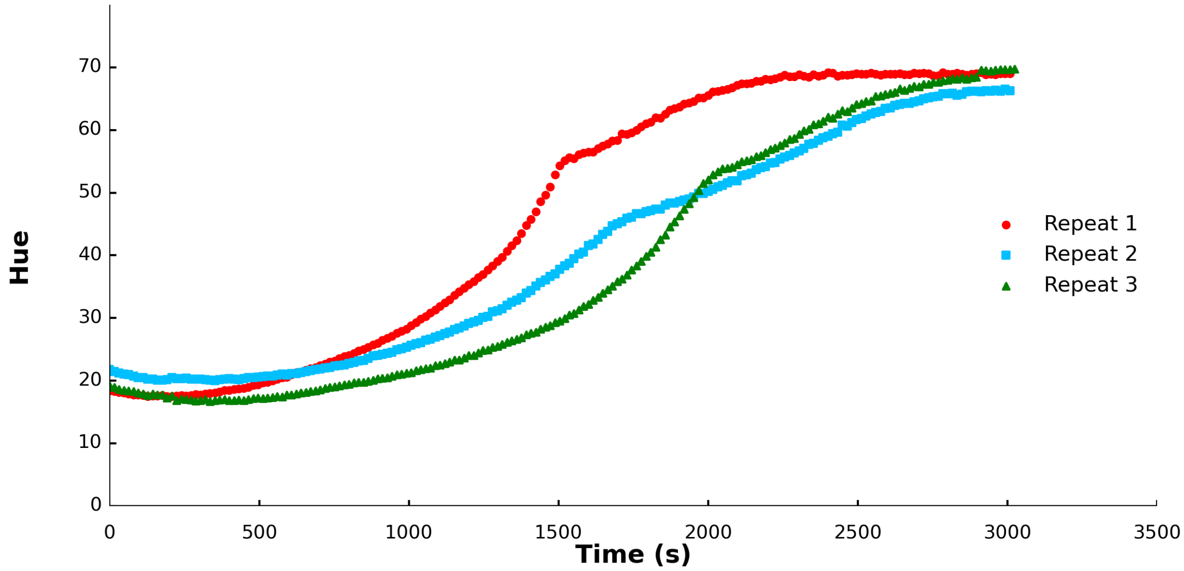

Figure S18: Three repeats of Kineticolor analysis of complex 1 when exposed to CO produced from 2,4,6 trichlorophenyl formate.

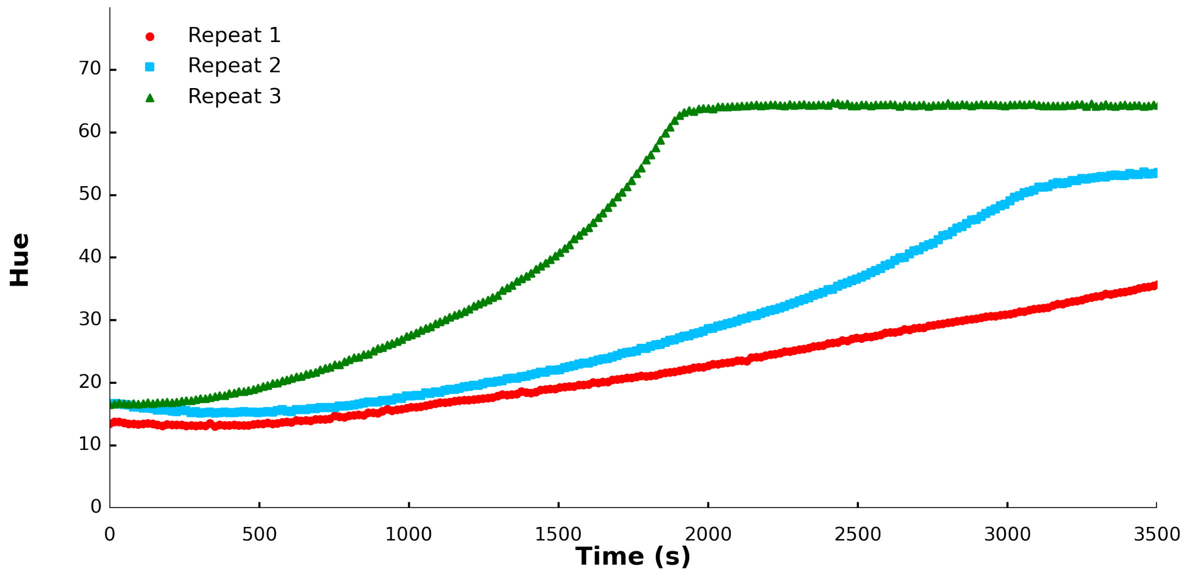

Figure S19: Three repeats of Kineticolor analysis of complex 1 when exposed to CO produced from COgen.

The triplicate hue–time profiles for 2,4,6-trichlorophenyl formate (Figure S18) and COgen (Figure S19) exhibit greater inter-replicate variability than the majority of surrogates studied. For COgen, this is attributed to the practical challenges of accurately transferring the very small quantities of Pd catalyst required on this scale, which introduces variability in the rate of Pd-catalysed CO release. For 2,4,6-trichlorophenyl formate, the variability likely reflects the sensitivity of the base-mediated elimination to minor differences in mixing efficiency upon injection of the triethylamine trigger. It is important to note that the Weibull curve-fitting and area-under-the-curve integration used to calculate surrogate scores mitigate the impact of point-to-point noise on the final scored metric, as evidenced by the interpretable standard deviations reported in Figure 10.

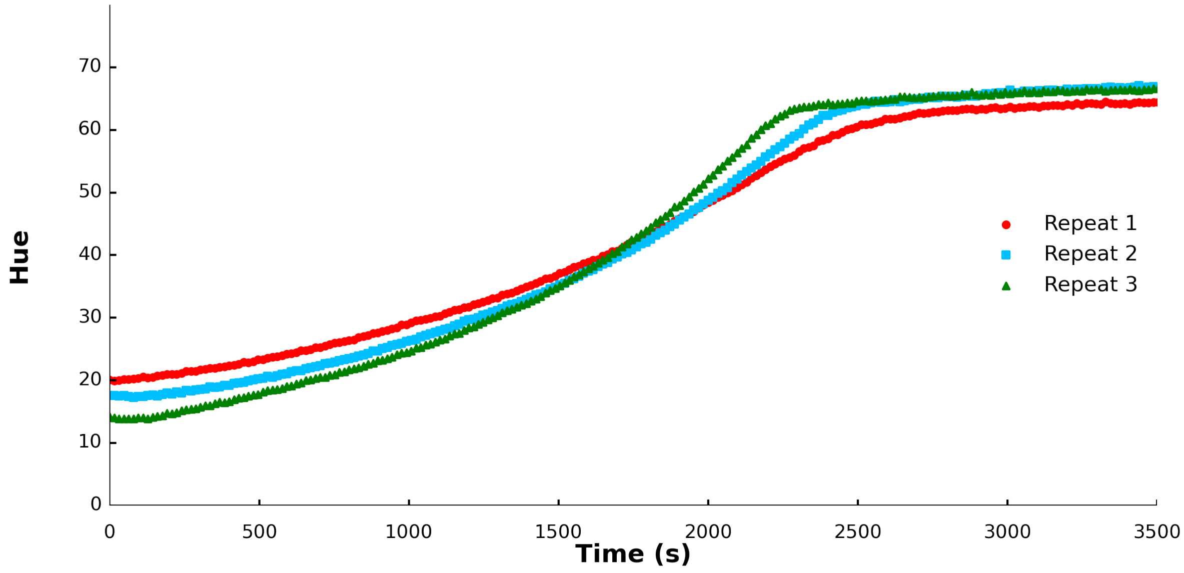

Figure S20: Three repeats of Kineticolor analysis of complex 1 when exposed to CO produced from $Cr(CO)_6$.

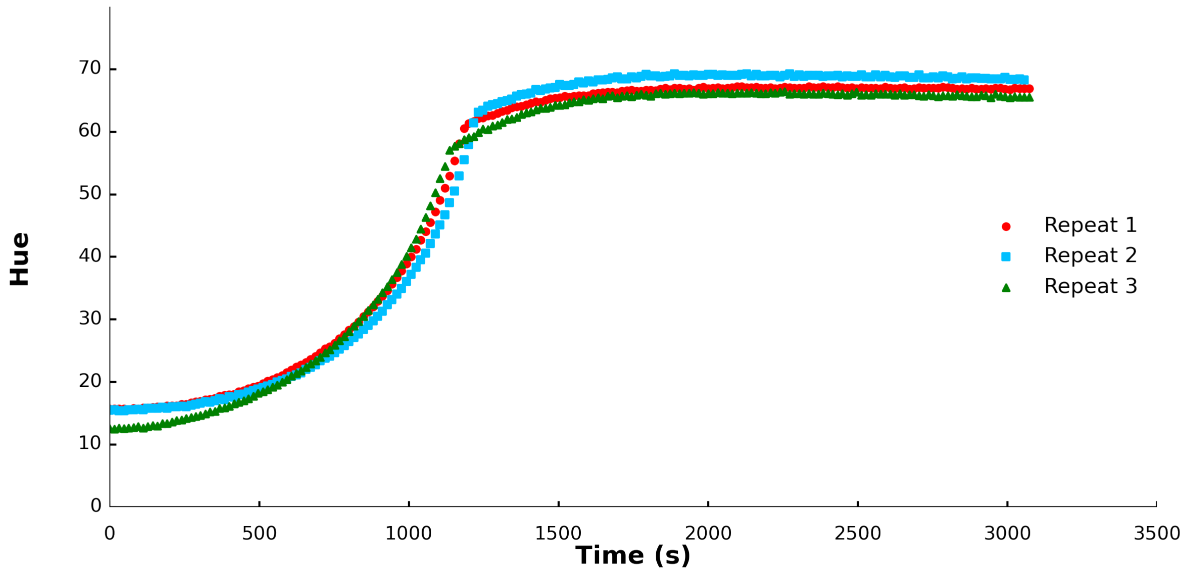

Figure S21: Three repeats of Kineticolor analysis of complex 1 when exposed to CO produced from formic acid.

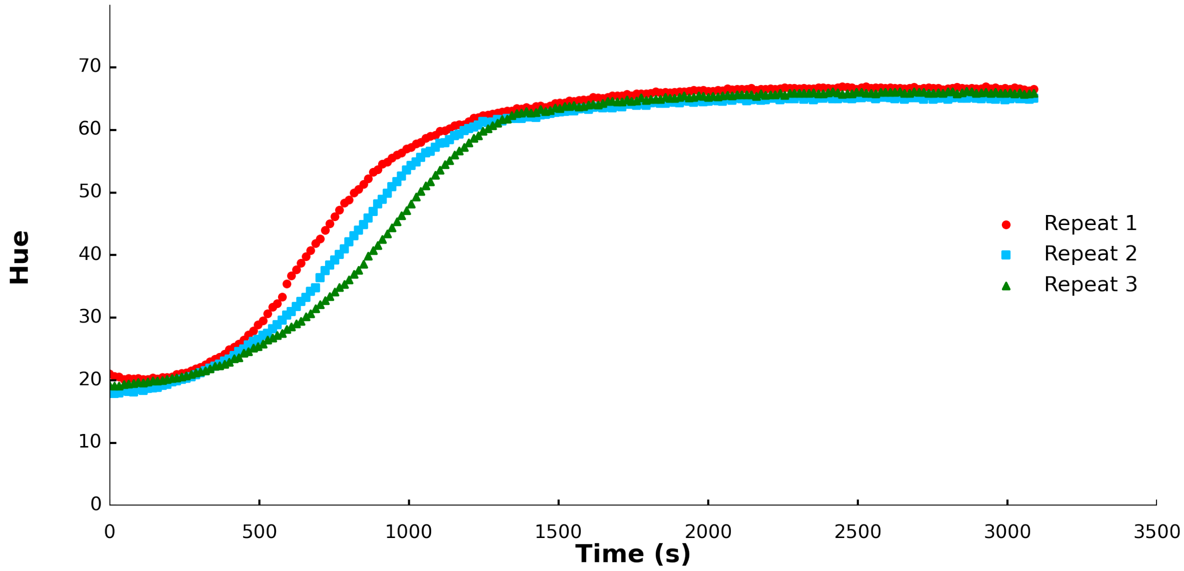

Figure S22: Three repeats of Kineticolor analysis of complex 1 when exposed to CO produced from $Mn(CO)_5Br$.

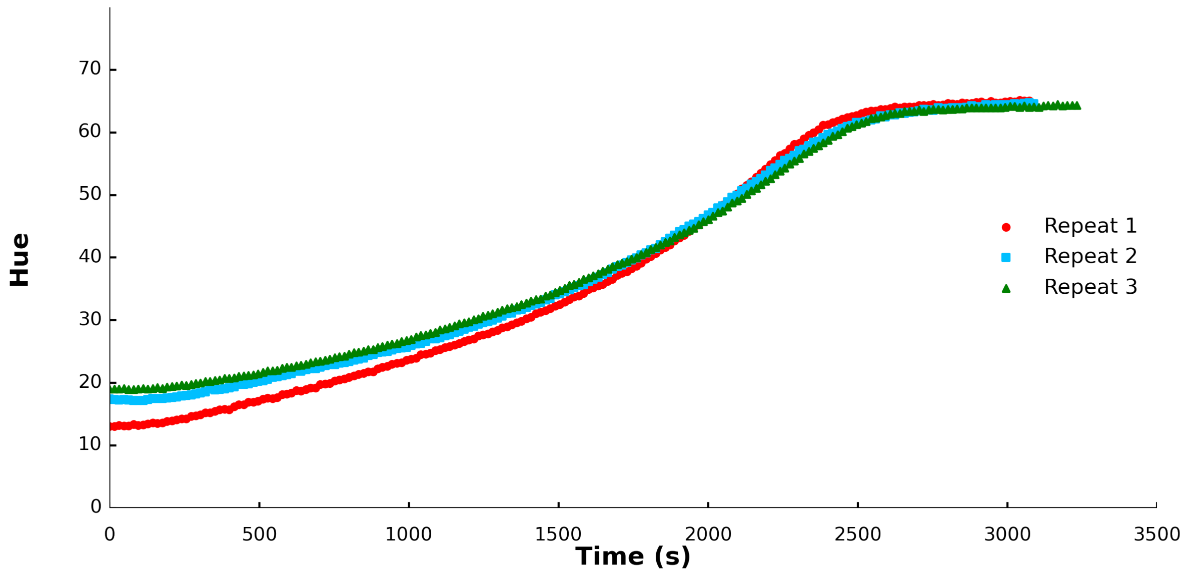

Figure S23: Three repeats of Kineticolor analysis of complex 1 when exposed to CO produced from $Mo(CO)_6$.

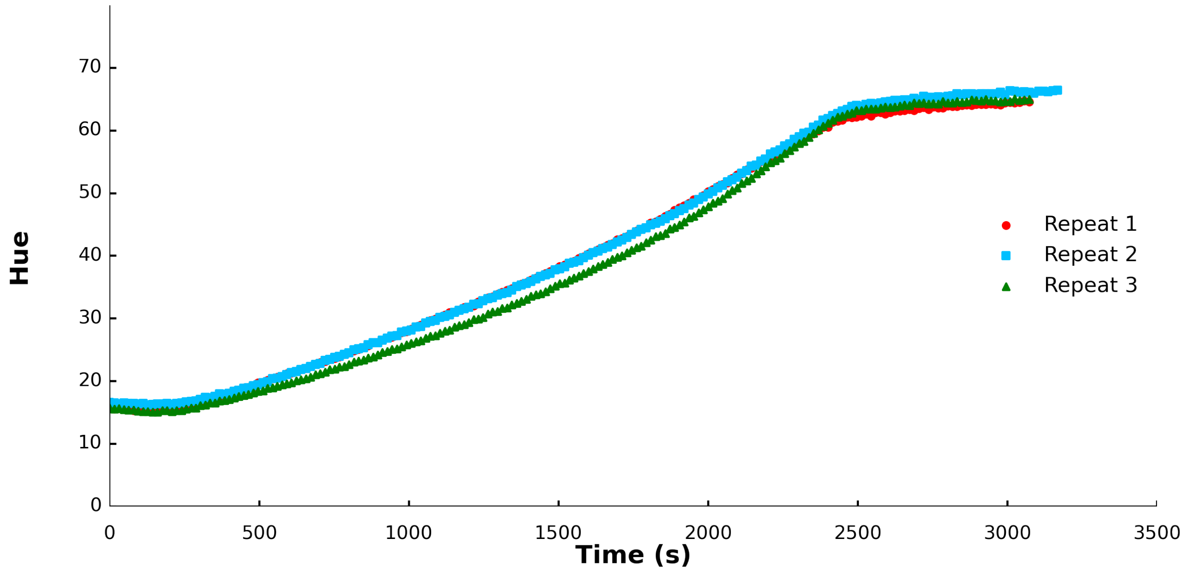

Figure S24: Three repeats of Kineticolor analysis of complex 1 when exposed to CO produced from N-formylsaccharin.

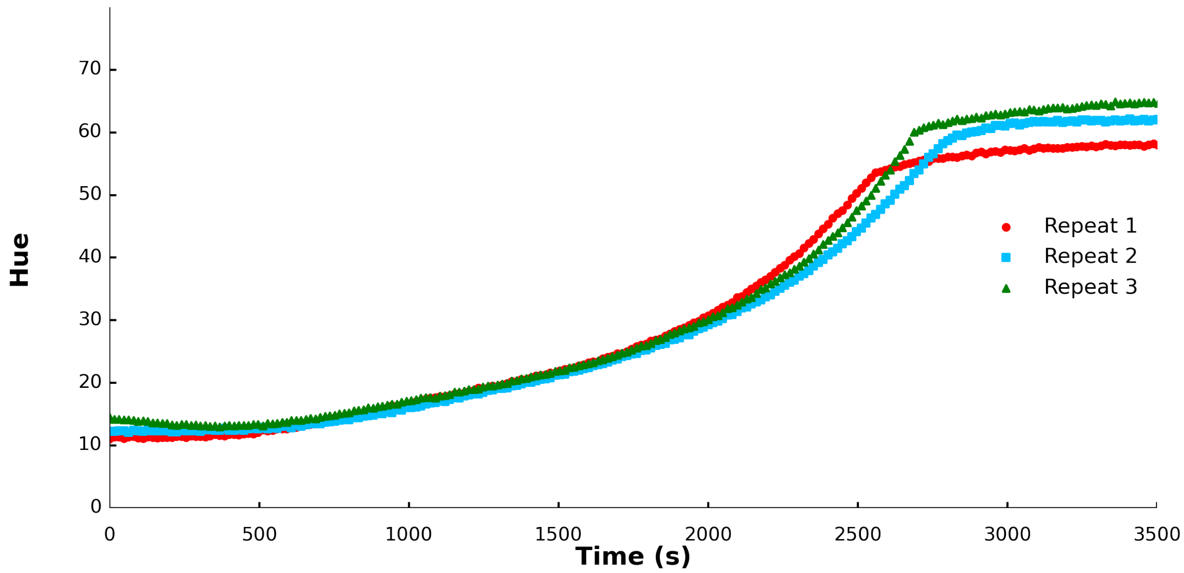

Figure S25: Three repeats of Kineticolor analysis of complex 1 when exposed to CO produced from SilaCO.

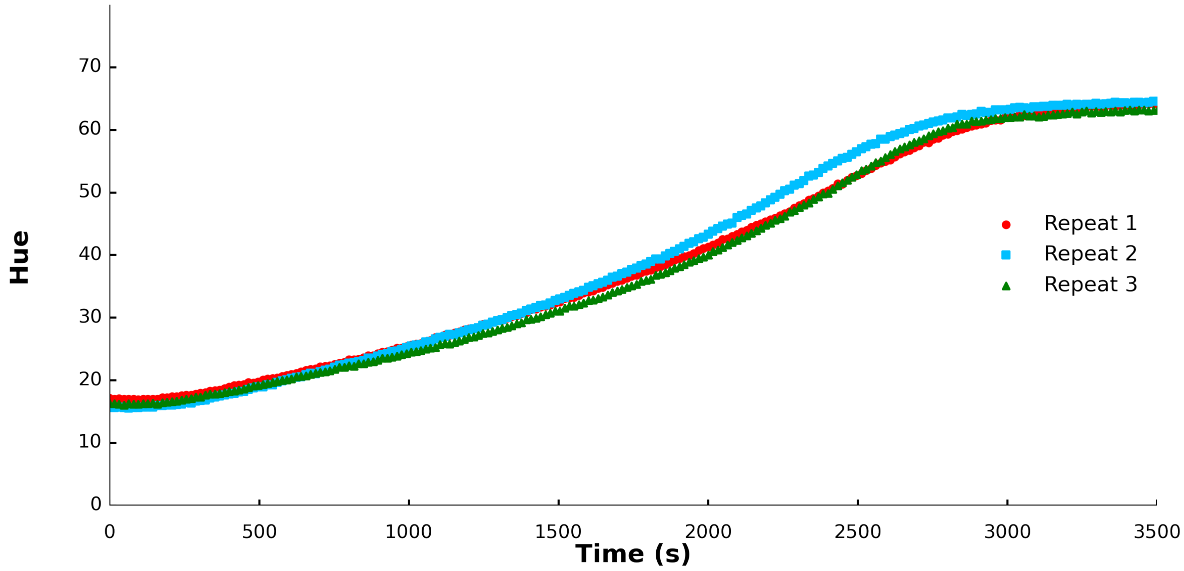

Figure S26: Three repeats of Kineticolor analysis of complex 1 when exposed to CO produced from $W(CO)_6$.

3.2.3. Weibull curve fitting data

The Weibull distribution was selected as it flexibly captures induction, growth, and plateau behavior without assuming a mechanistic rate law. The model was selected having produced the lowest error (RMSE, MAE) when fit to hue vs time data for each CO surrogate studied (Table S9).

All raw data and code pertaining to calculation of Weibull model errors versus Gompertz and other related growth functions is available in the machine-readable information made available on figshare.

Table S9: Statistical comparison of five growth functions applied to the dataset, where n = 27 independent measurements of ΔE. RSS, residual sum of squares; MAE, Mean absolute error; RMSE, Root mean square error; AIC, Akaike information criterion.

Growth Model	average RSS	average MAE	average RMSE	average AIC
Weibull	316.88	0.96	1.15	41.53
Rogers	563.59	1.23	1.50	138.58
Zwietering	563.59	1.23	1.50	138.58
Lay	563.59	1.23	1.50	138.58
Gompertz	2535.33	2.74	3.29	487.31

Table S10: Full details of the Weibull parameters for each analysis carried out. A-scale parameter, b – shape parameter, c – Plateau parameter and d – offset parameter.

Surrogate	File name	a	b	c	d
**Balloon**	benchmark	79.9	4.3	47.6	2.0
**$Mo(CO)_6$**	Color Analysis KD-009E-002	2088.2	2.9	55.1	2.6
	Color Analysis KD-009E-003	2133.6	2.8	51.2	1.7
	Color Analysis KD-009E-004	2205.3	2.8	50.8	1.5
**$W(CO)_6$**	Color Analysis KD-010E-001	48804.5	1.6	3775.7	-0.2
	Color Analysis KD-010E-003	3255.6	1.9	86.2	0.5
	Color Analysis KD-010E-004	15626.1	1.7	866.6	0.3
**$Cr(CO)_6$**	Color Analysis KD-011E-001	2157.6	2.5	48.7	1.6
	Color Analysis KD-011E-002	2054.0	3.0	51.0	1.6
	Color Analysis KD-011E-003	1880.8	3.2	51.3	2.3
**Formic acid**	Color Analysis KD-012E-002	1081.8	4.8	49.7	1.4
	Color Analysis KD-012E-003	1126.3	5.3	51.6	1.7
	Color Analysis KD-012E-004	1050.6	4.2	51.6	1.7
**$Mn(CO)_5Br$**	Color Analysis KD-013E-002	825.9	2.5	46.9	-1.9
	Color Analysis KD-013E-003	895.5	2.8	46.1	0.6
	Color Analysis KD-013E-004	1017.1	3.2	45.5	0.9
***N*-formylsaccharin**	Color Analysis KD-014E-001	2128.5	2.4	56.9	0.6
	Color Analysis KD-014E-002	2163.6	2.2	59.7	0.3
	Color Analysis KD-014E-003	2072.9	2.1	57.1	0.6
**SilaCO**	Color Analysis KD-015E-001	60627.2	2.7	184745.1	0.4
	Color Analysis KD-015E-002	2550.7	3.5	57.6	1.7
	Color Analysis KD-015E-003	3197.0	3.5	96.7	-0.3
**COgen**	Color Analysis KD-030E-011	1608.6	4.0	46.4	2.2
	Color Analysis KD-030E-012	3378.8	2.0	33.0	-0.4
	Color Analysis KD-030E-013	76466.1	2.3	60812.4	-1.3
**2,4,6 trichlorophenyl formate**	Color Analysis MG E003 006	2020.4	4.2	51.2	-1.5
	Color Analysis MG E003 007	1984.3	2.9	48.5	-1.9
	Color Analysis MG E003 008	1492.9	3.7	50.6	-0.1

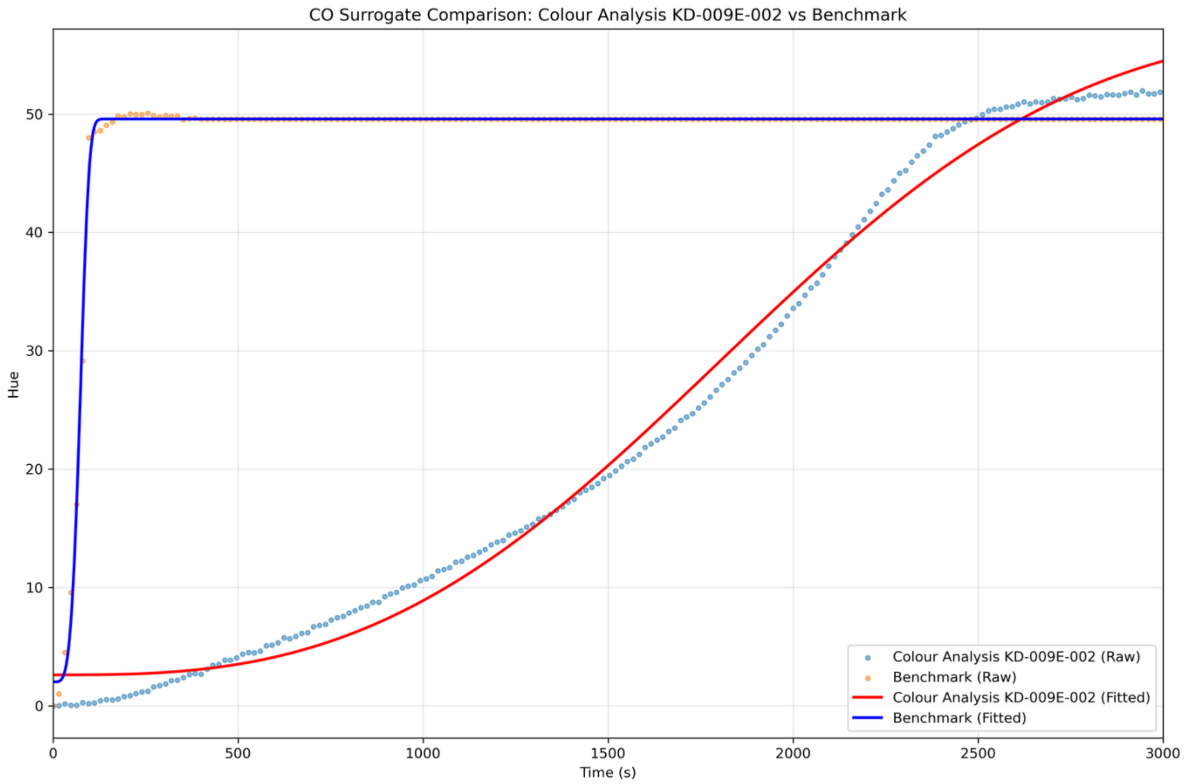

Figure S27: Weibull curves used to calculate surrogate score for KD-009E-002, $Mo(CO)_6$ surrogate.

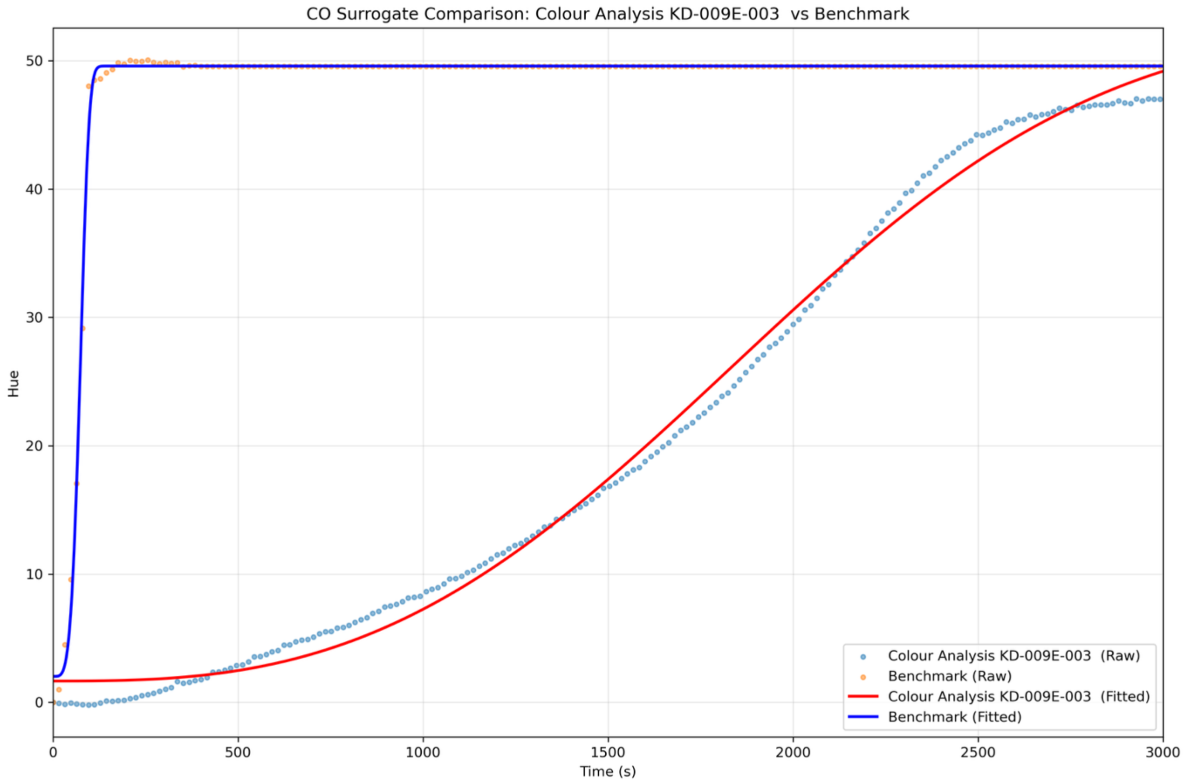

Figure S28: Weibull curves used to calculate surrogate score for KD-009E-003, $Mo(CO)_6$ surrogate.

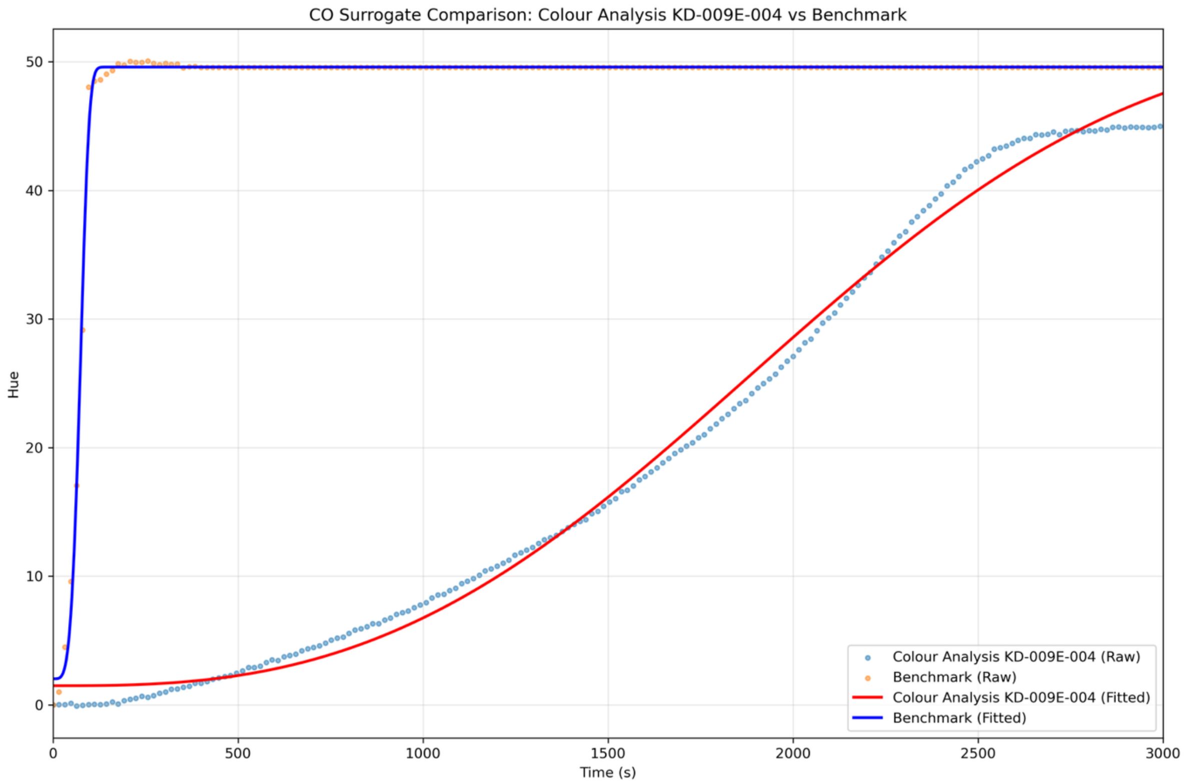

Figure S29: Weibull curves used to calculate surrogate score for KD-009E-004, $Mo(CO)_6$ surrogate.

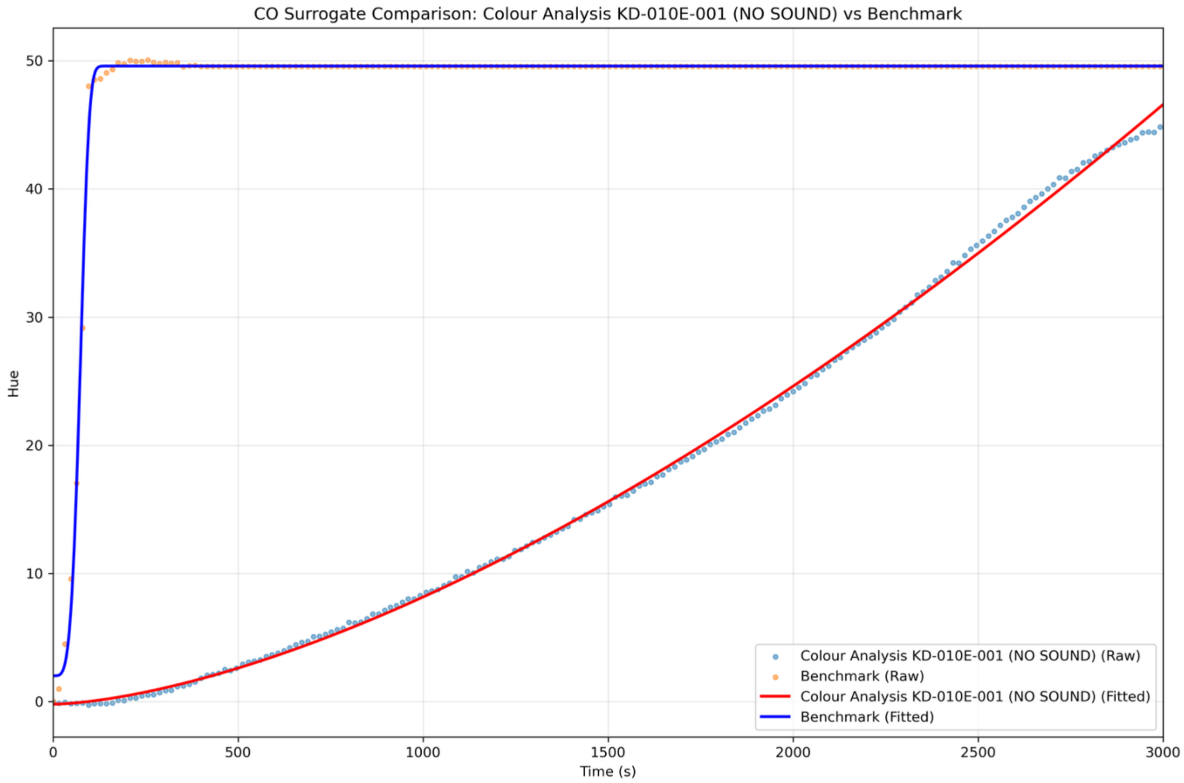

Figure S30: Weibull curves used to calculate surrogate score for KD-010E-001, $W(CO)_6$ surrogate.

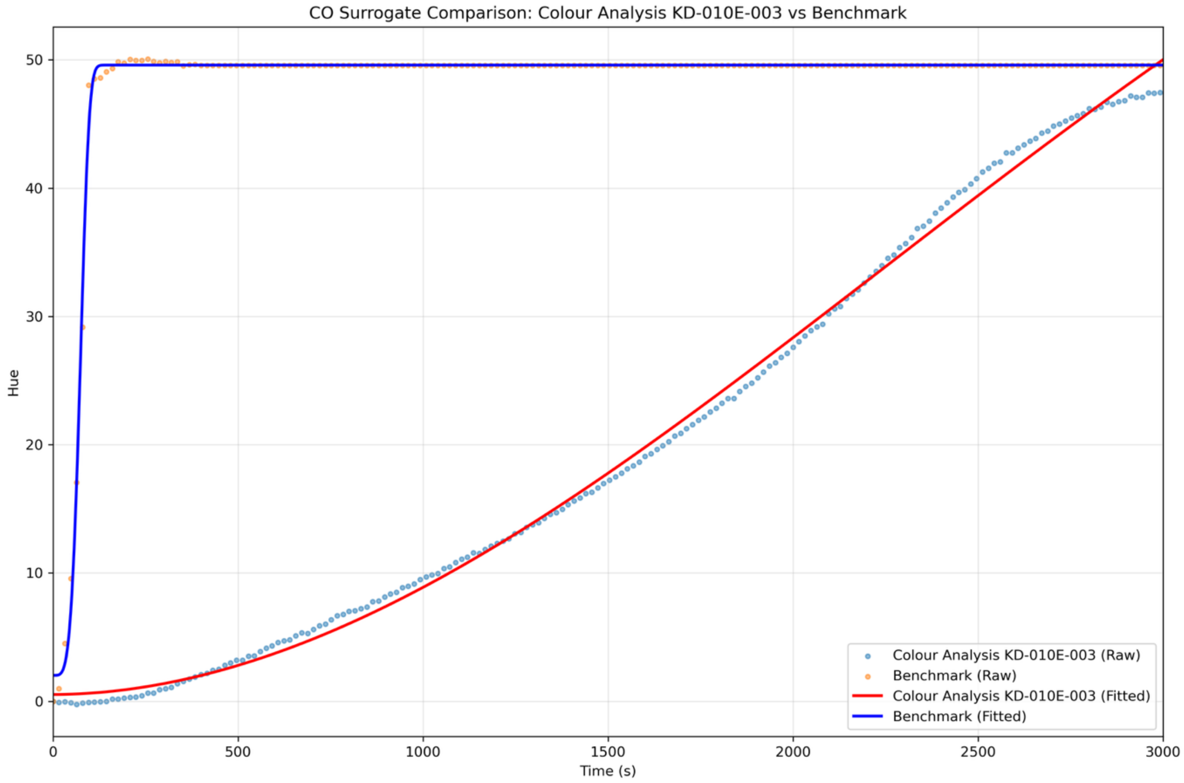

Figure S31: Weibull curves used to calculate surrogate score for KD-010E-003, $W(CO)_6$ surrogate.

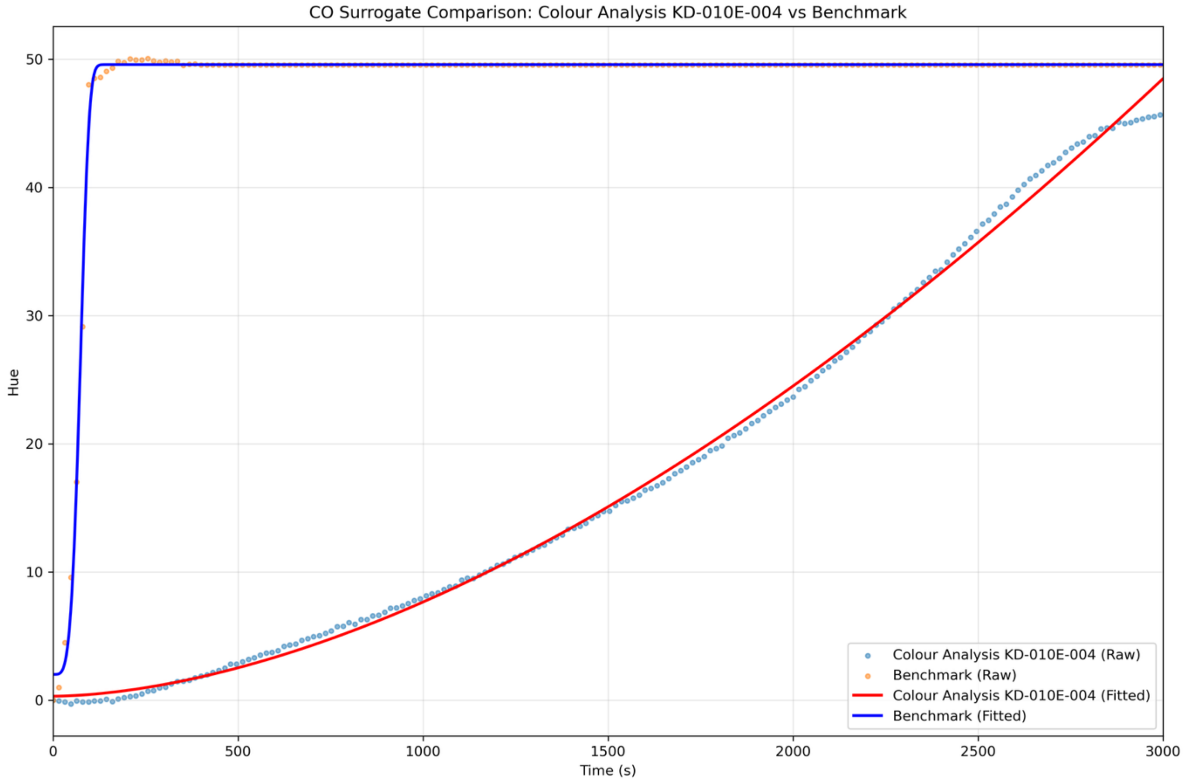

Figure S32: Weibull curves used to calculate surrogate score for KD-010E-004, $W(CO)_6$ surrogate.

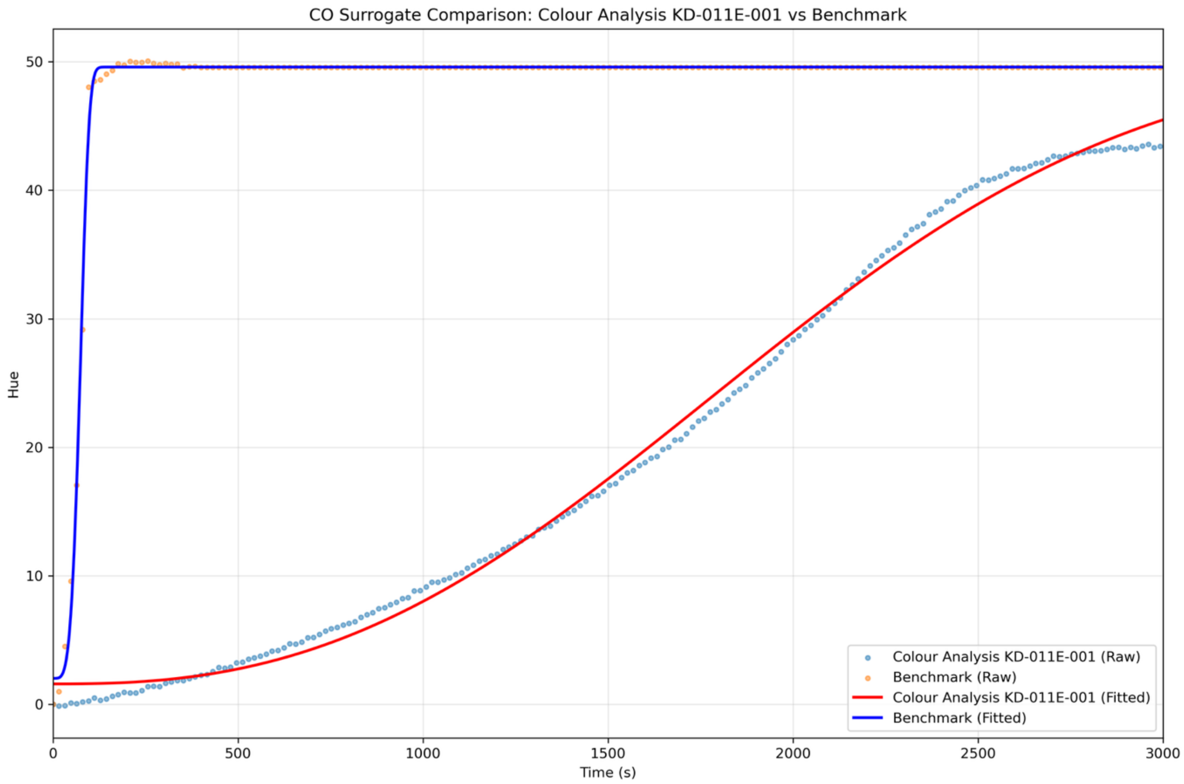

Figure S33: Weibull curves used to calculate surrogate score for KD-011E-001, $Cr(CO)_6$ surrogate.

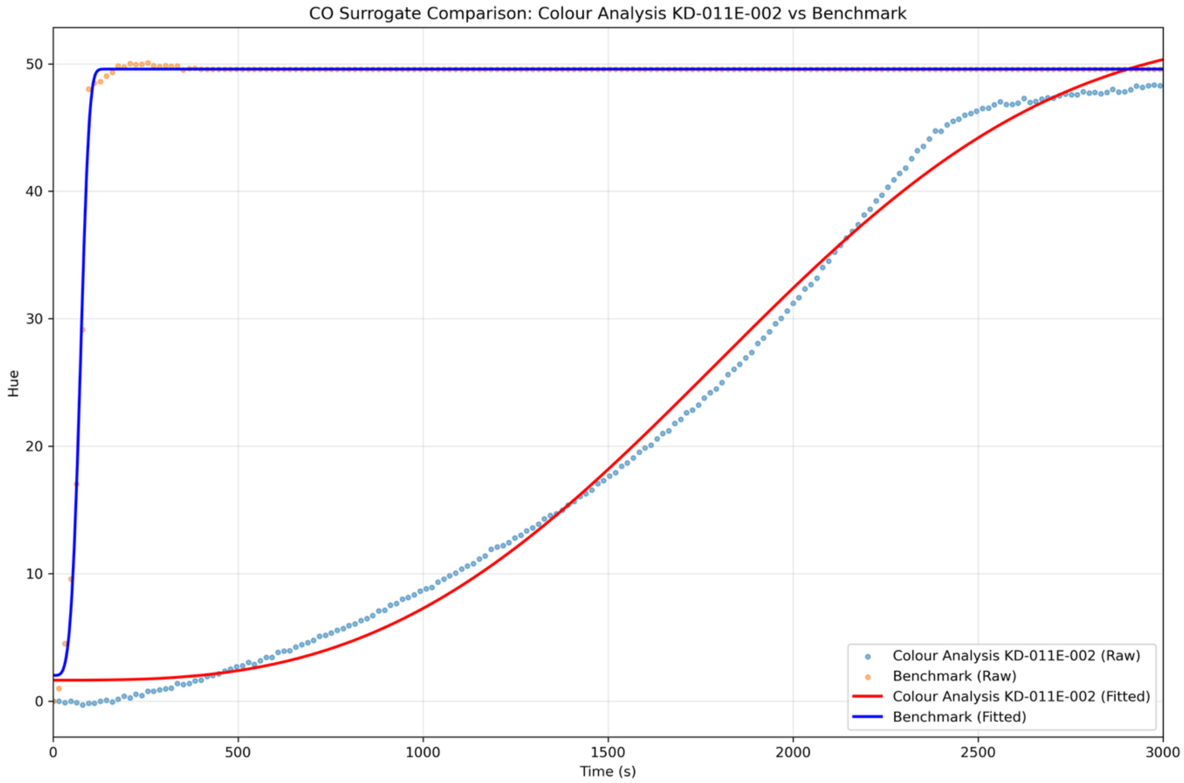

Figure S34: Weibull curves used to calculate surrogate score for KD-011E-002, $Cr(CO)_6$ surrogate.

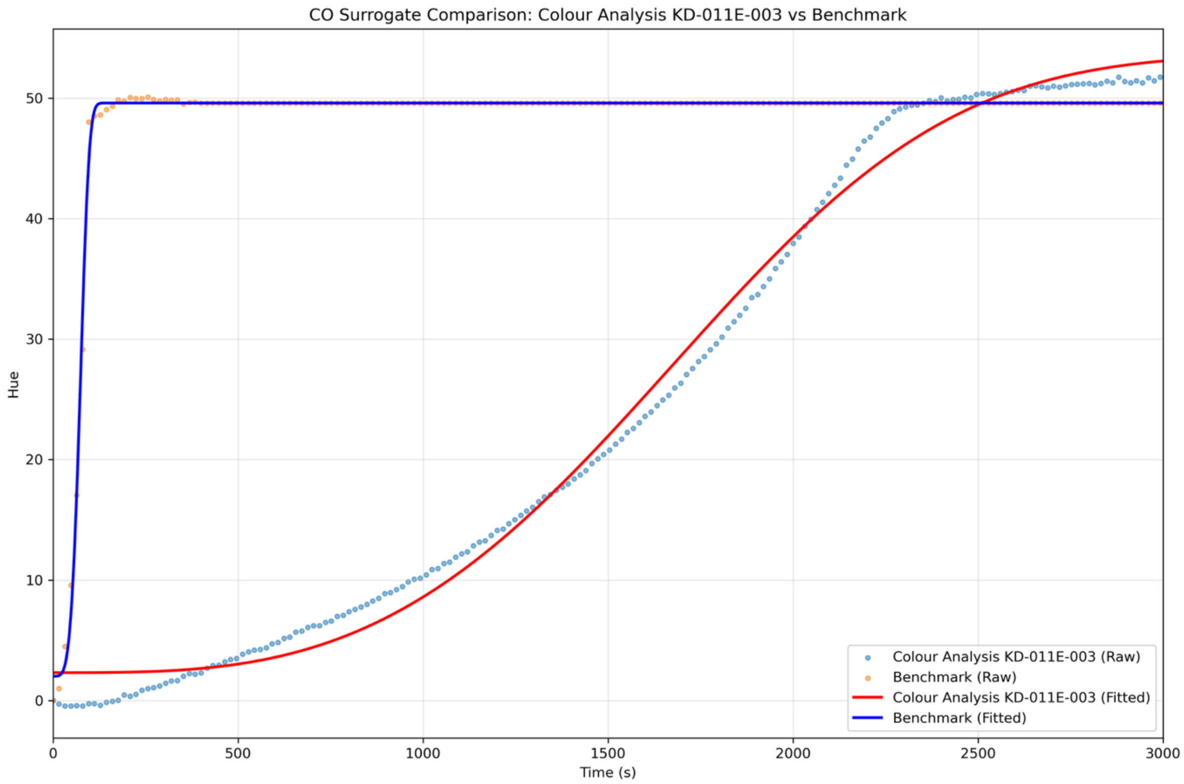

Figure S35: Weibull curves used to calculate surrogate score for KD-011E-003, $Cr(CO)_6$ surrogate.

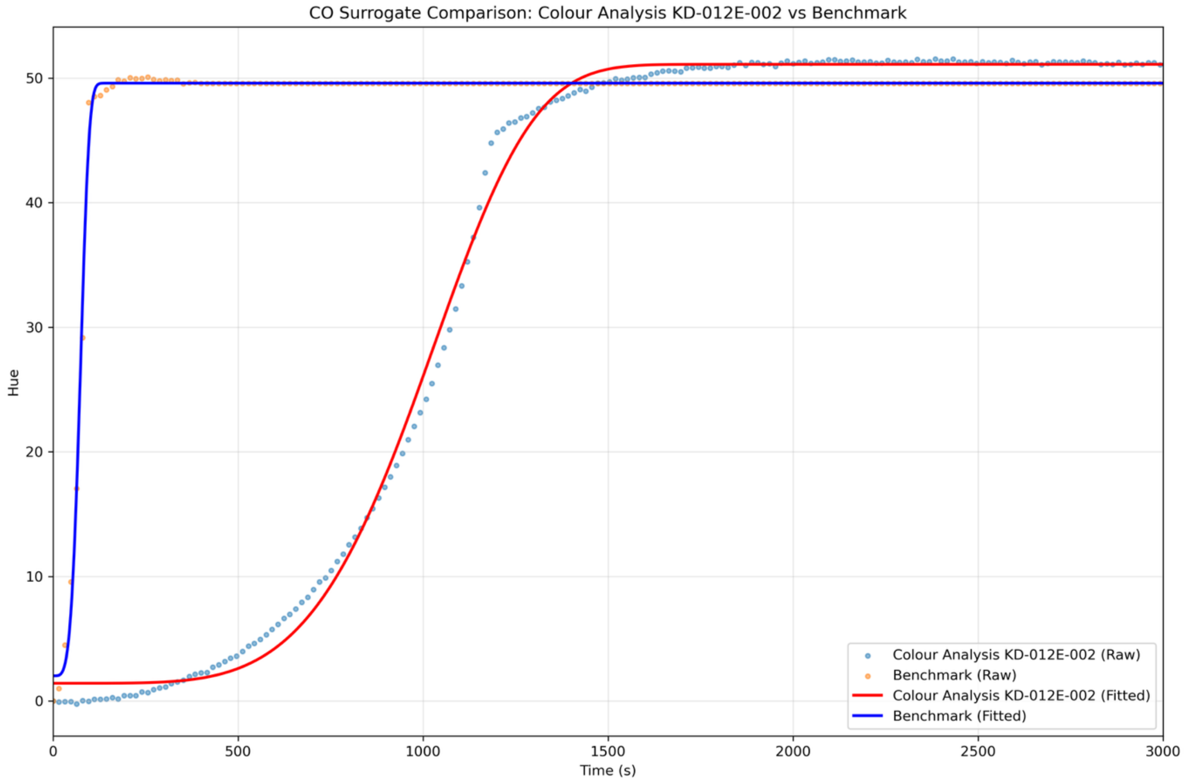

Figure S36: Weibull curves used to calculate surrogate score for KD-012E-002, formic acid surrogate.

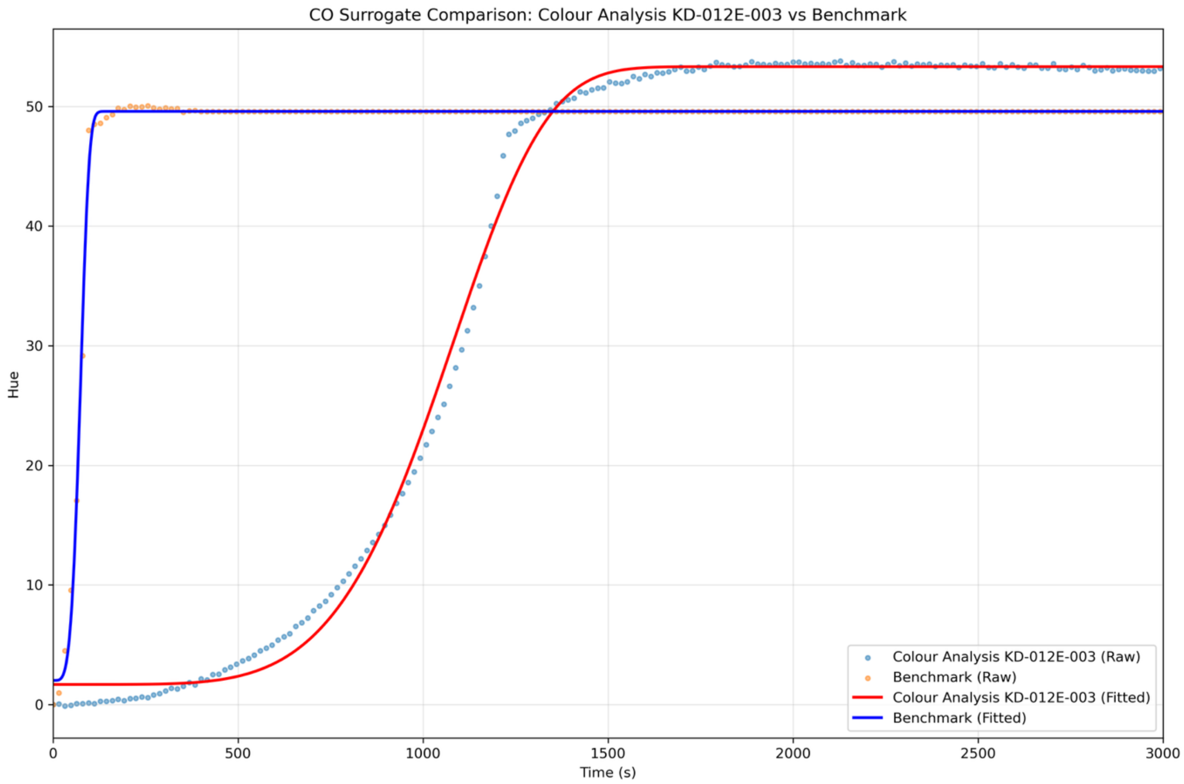

Figure S37: Weibull curves used to calculate surrogate score for KD-012E-003, formic acid surrogate.

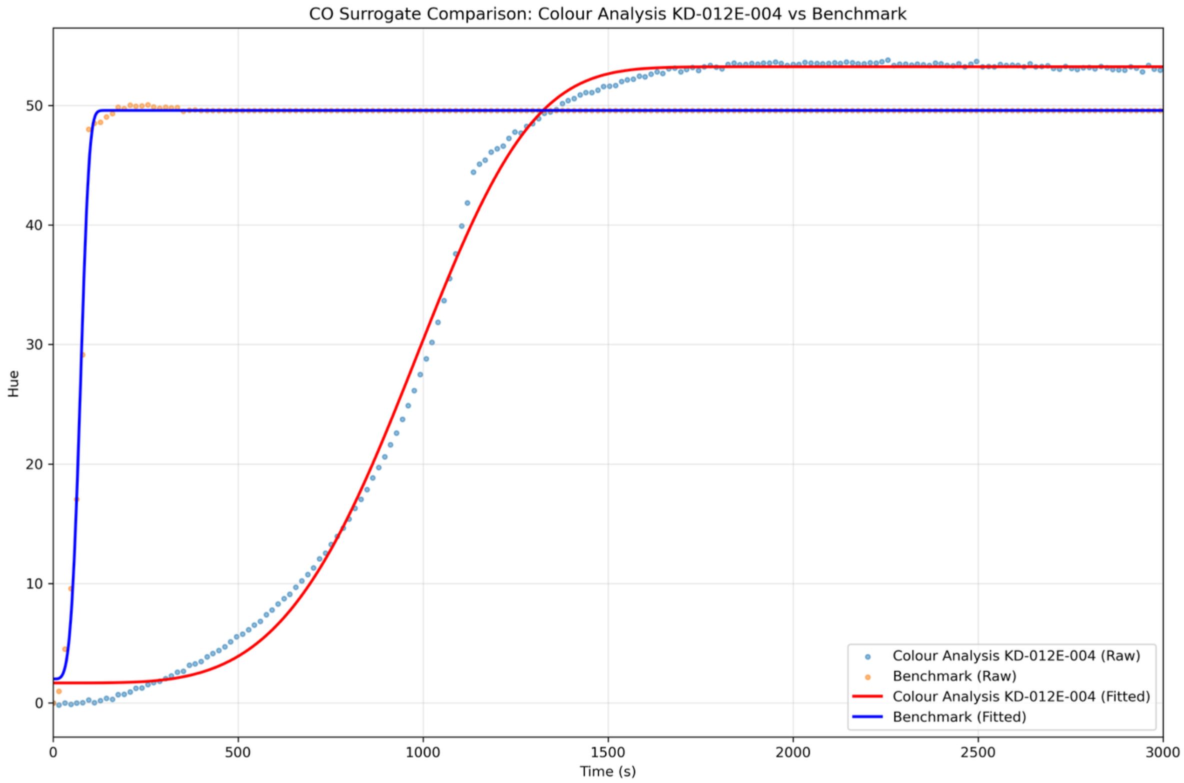

Figure S38: Weibull curves used to calculate surrogate score for KD-012E-004, formic acid surrogate.

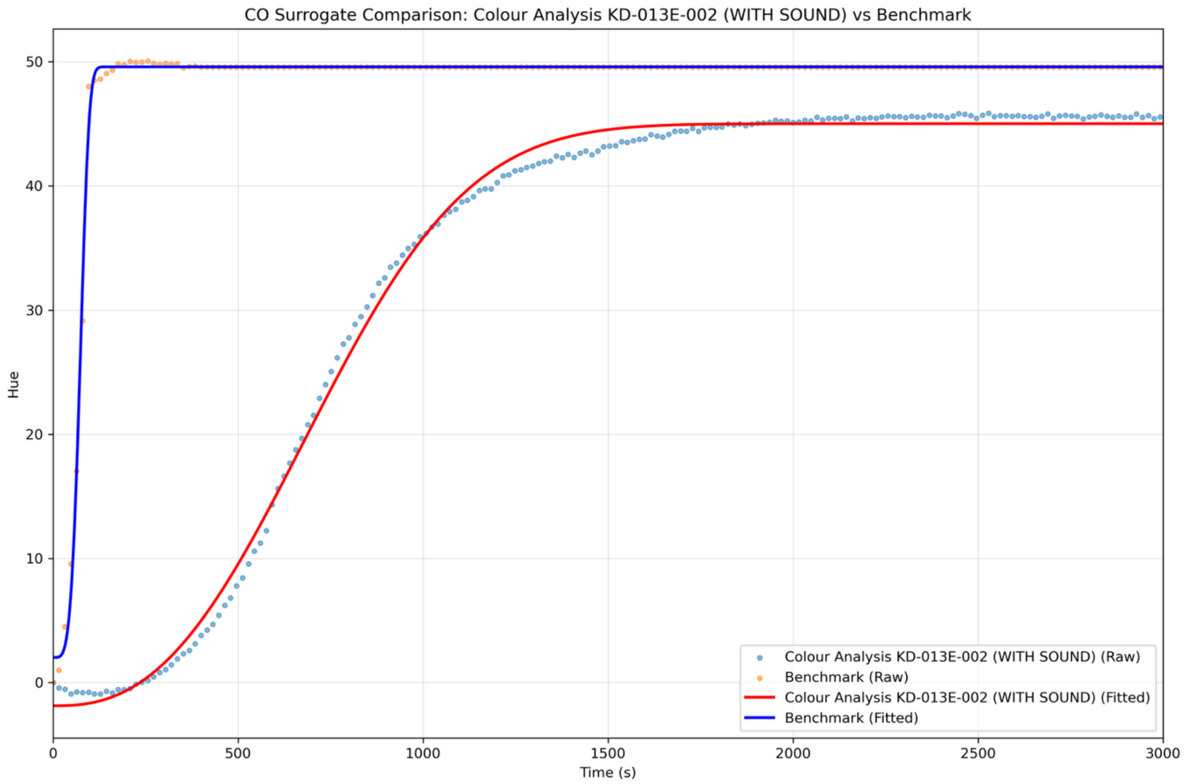

Figure S39: Weibull curves used to calculate surrogate score for KD-013E-002, $Mn(CO)_5Br$ surrogate.

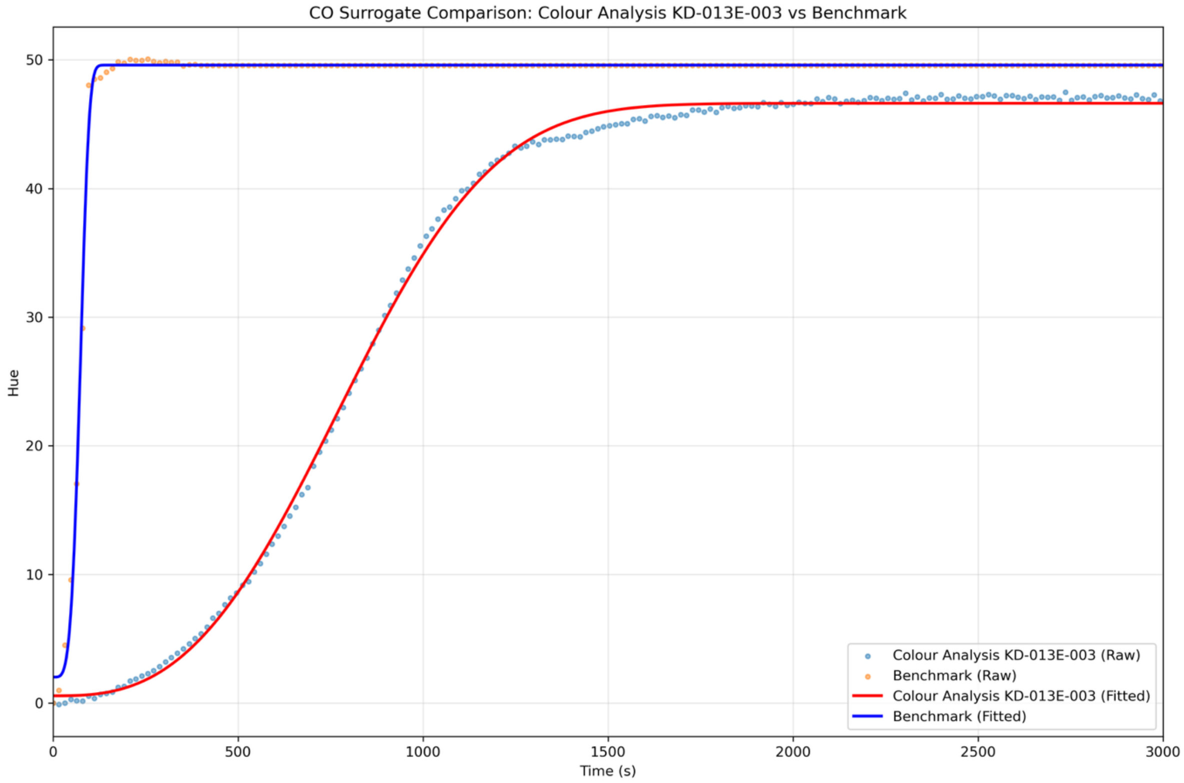

Figure S40: Weibull curves used to calculate surrogate score for KD-013E-003, $Mn(CO)_5Br$ surrogate.

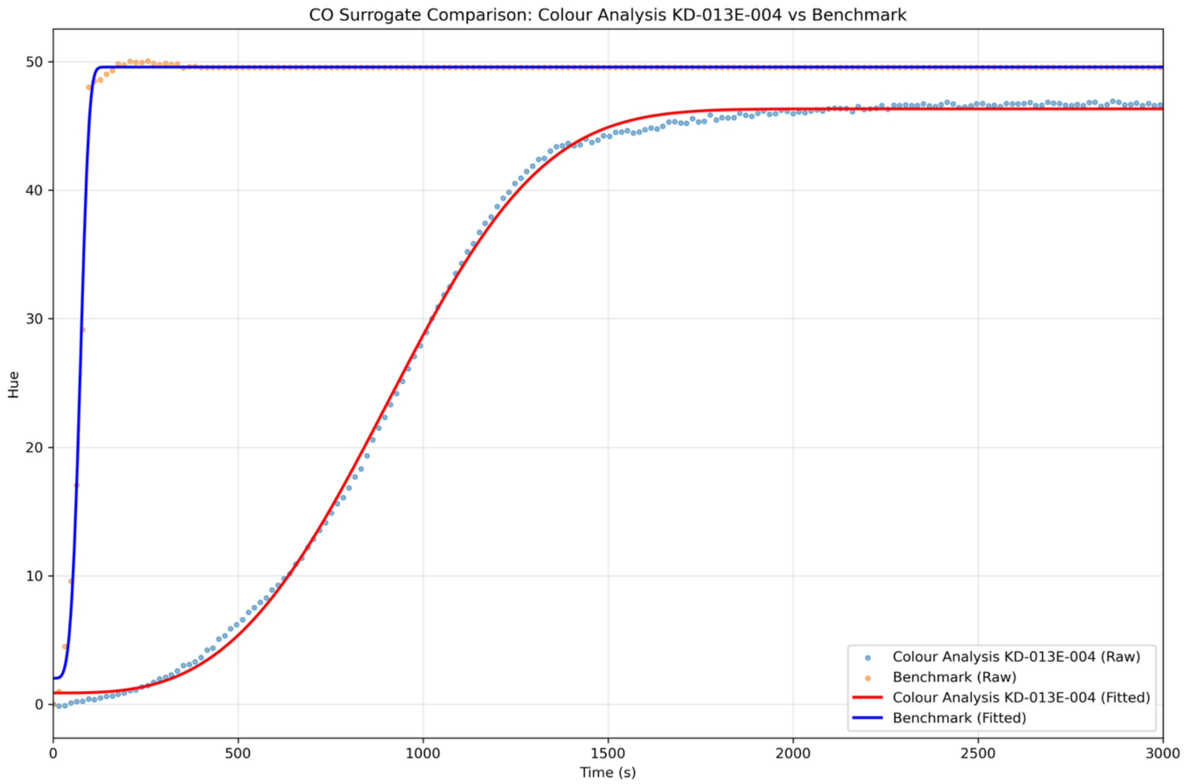

Figure S41: Weibull curves used to calculate surrogate score for KD-013E-004, $Mn(CO)_5Br$ surrogate.

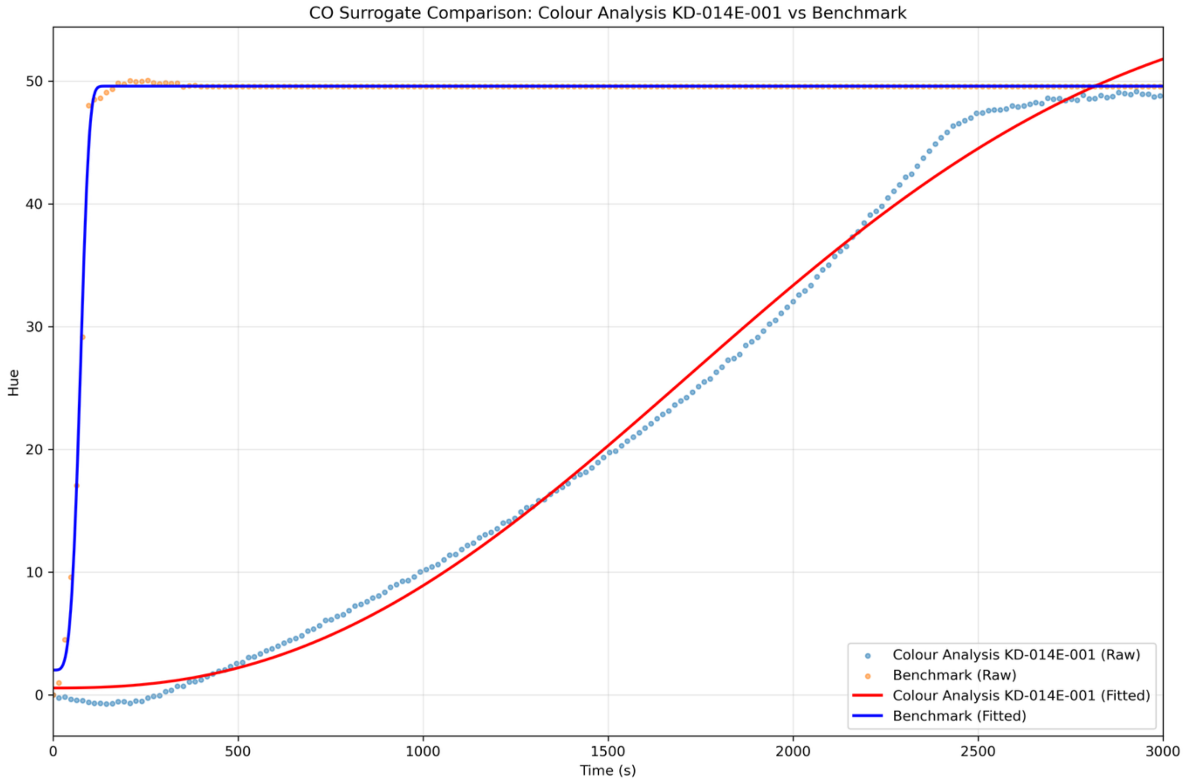

Figure S42: Weibull curves used to calculate surrogate score for KD-014E-001, N-formylsaccharin surrogate.

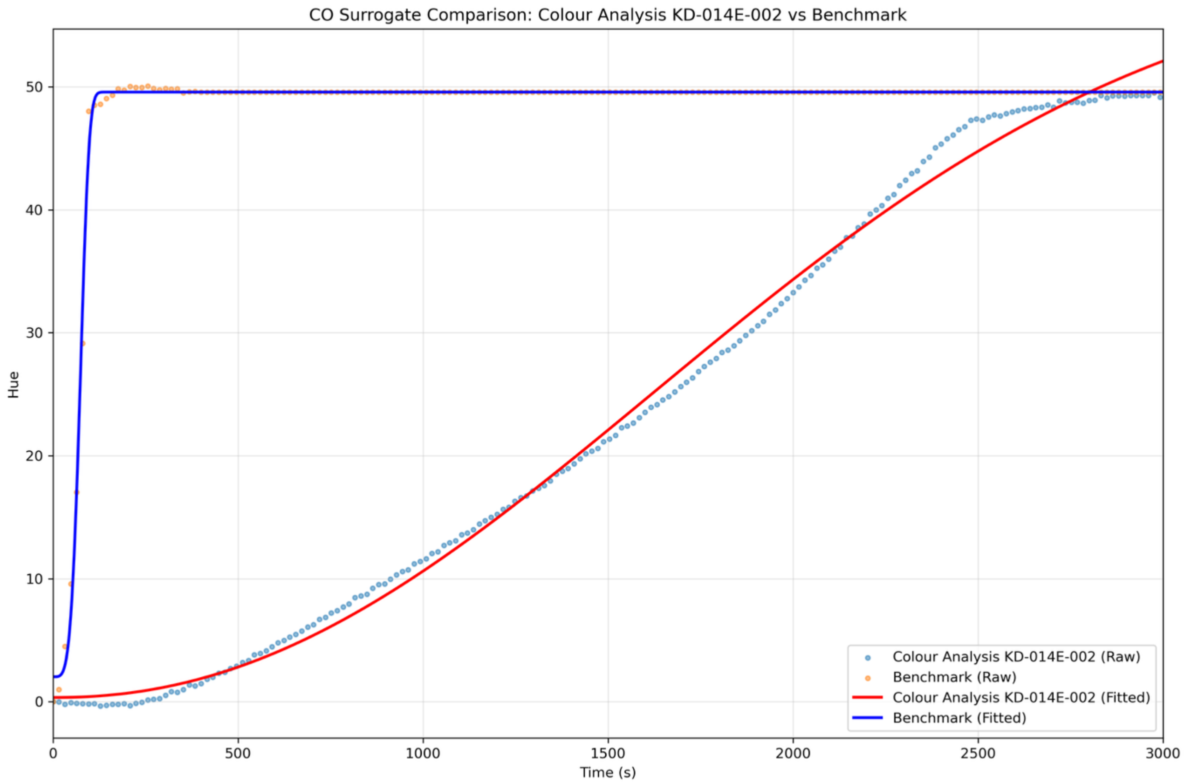

Figure S43: Weibull curves used to calculate surrogate score for KD-014E-002, N-formylsaccharin surrogate.

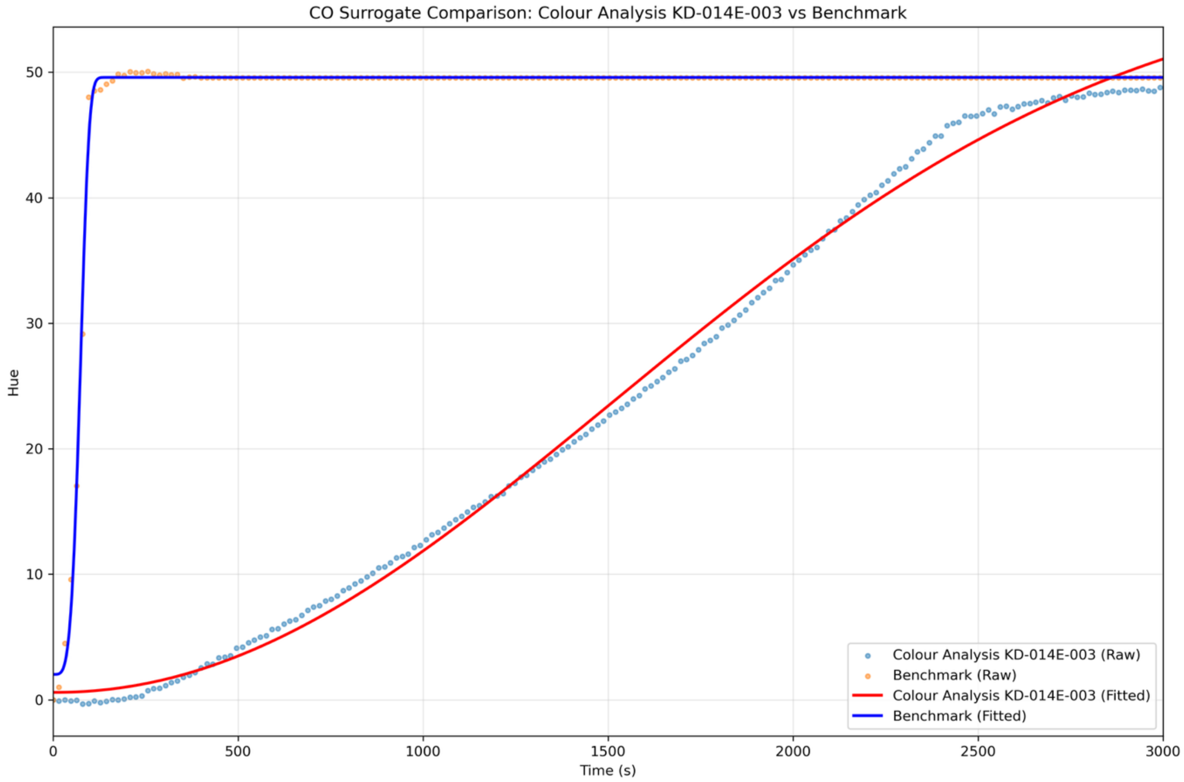

Figure S44: Weibull curves used to calculate surrogate score for KD-014E-003, N-formylsaccharin surrogate.

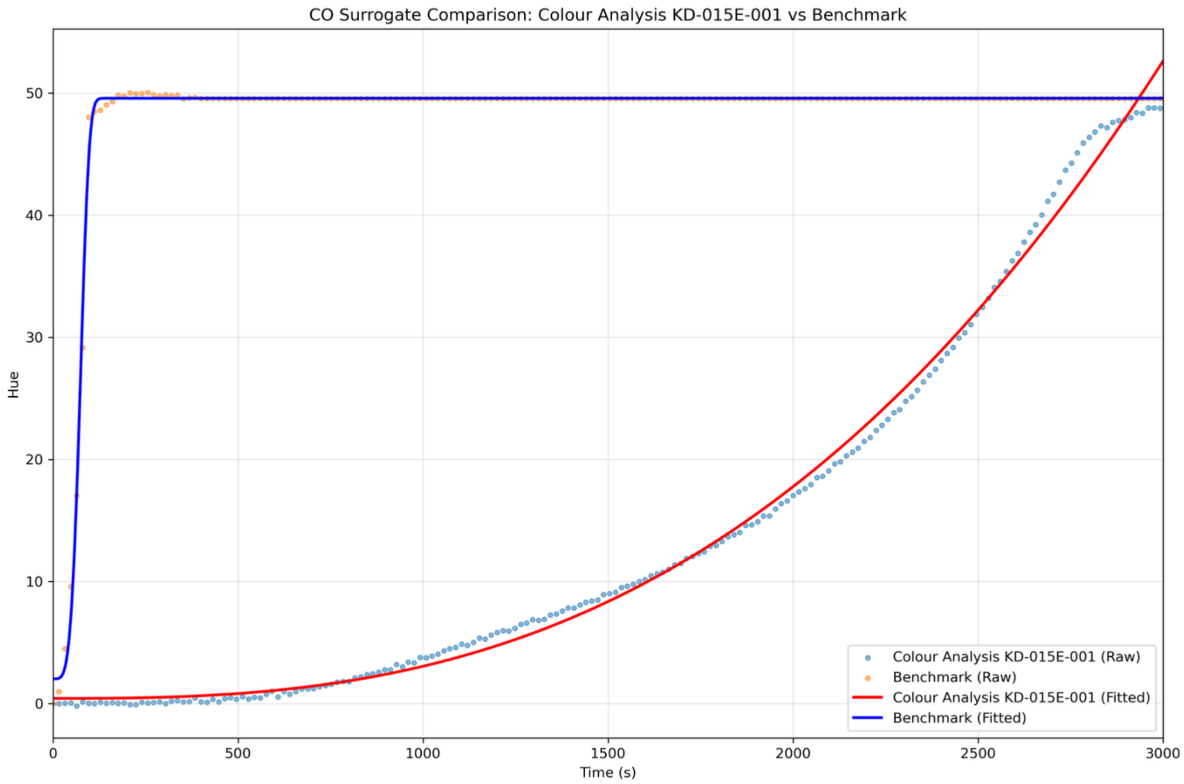

Figure S45: Weibull curves used to calculate surrogate score for KD-015E-001, SilaCO surrogate.

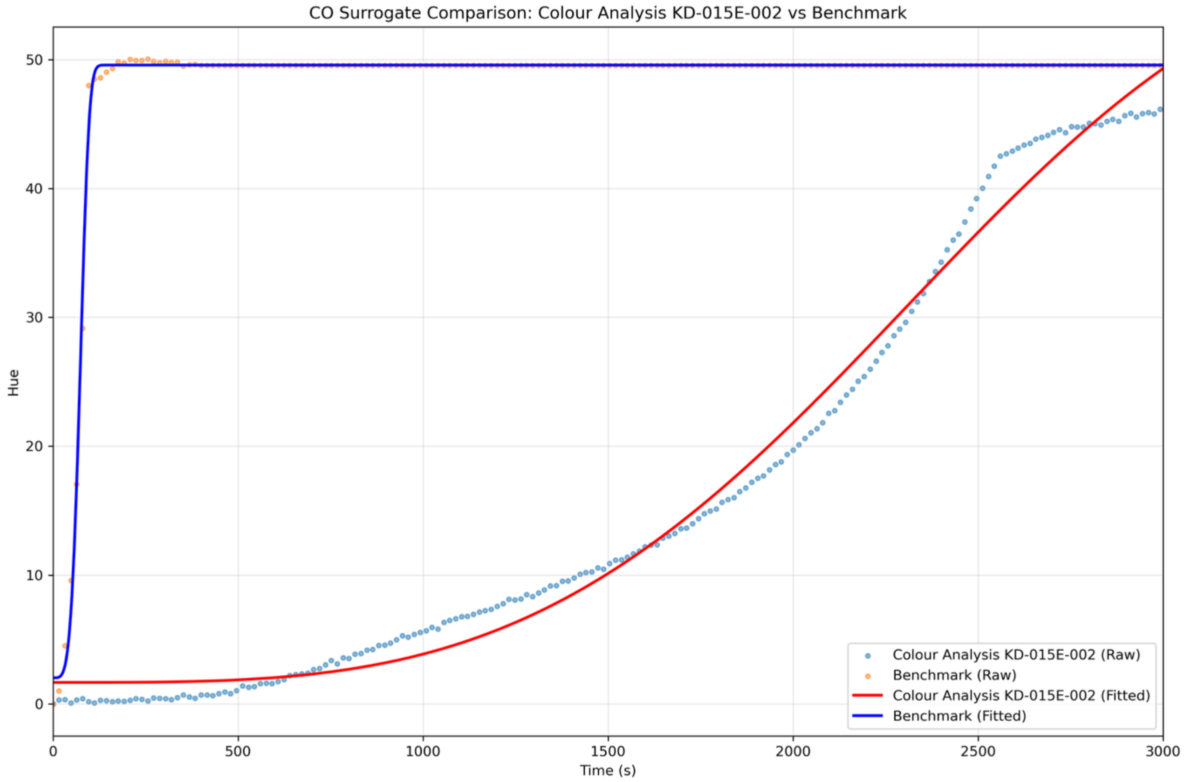

Figure S46: Weibull curves used to calculate surrogate score for KD-015E-002, SilaCO surrogate.

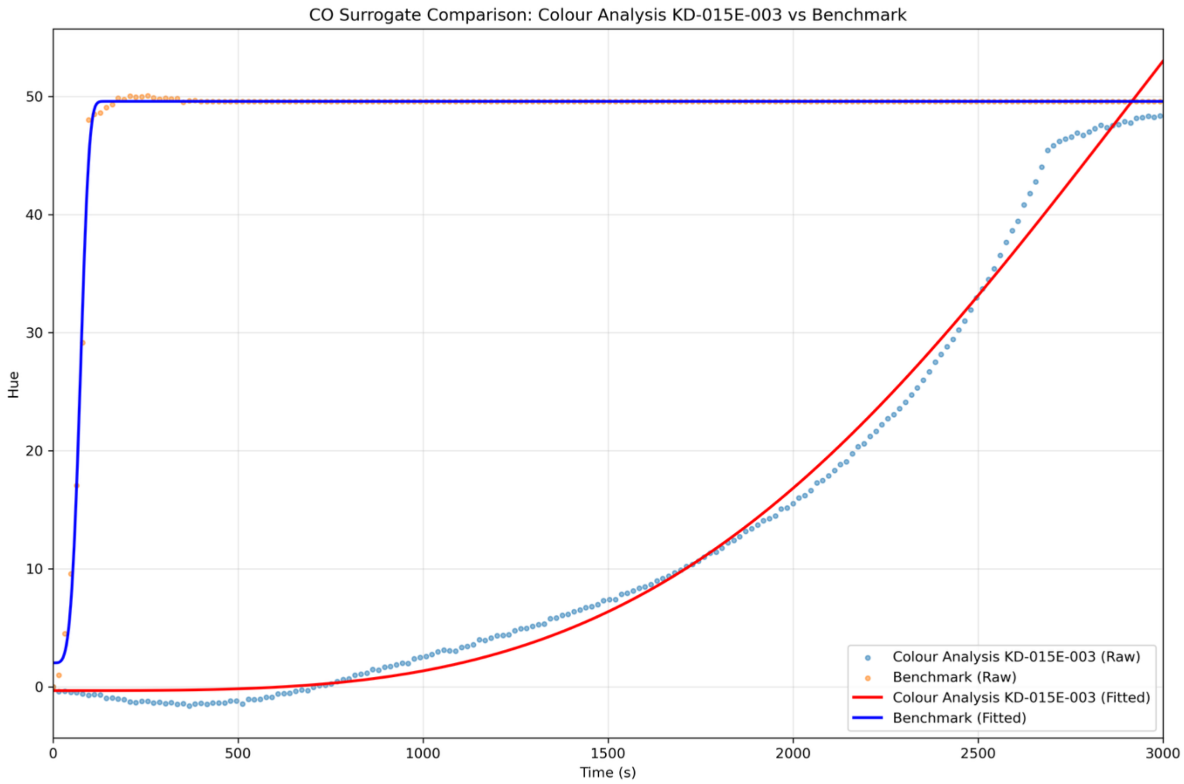

Figure S47: Weibull curves used to calculate surrogate score for KD-015E-003, SilaCO surrogate.

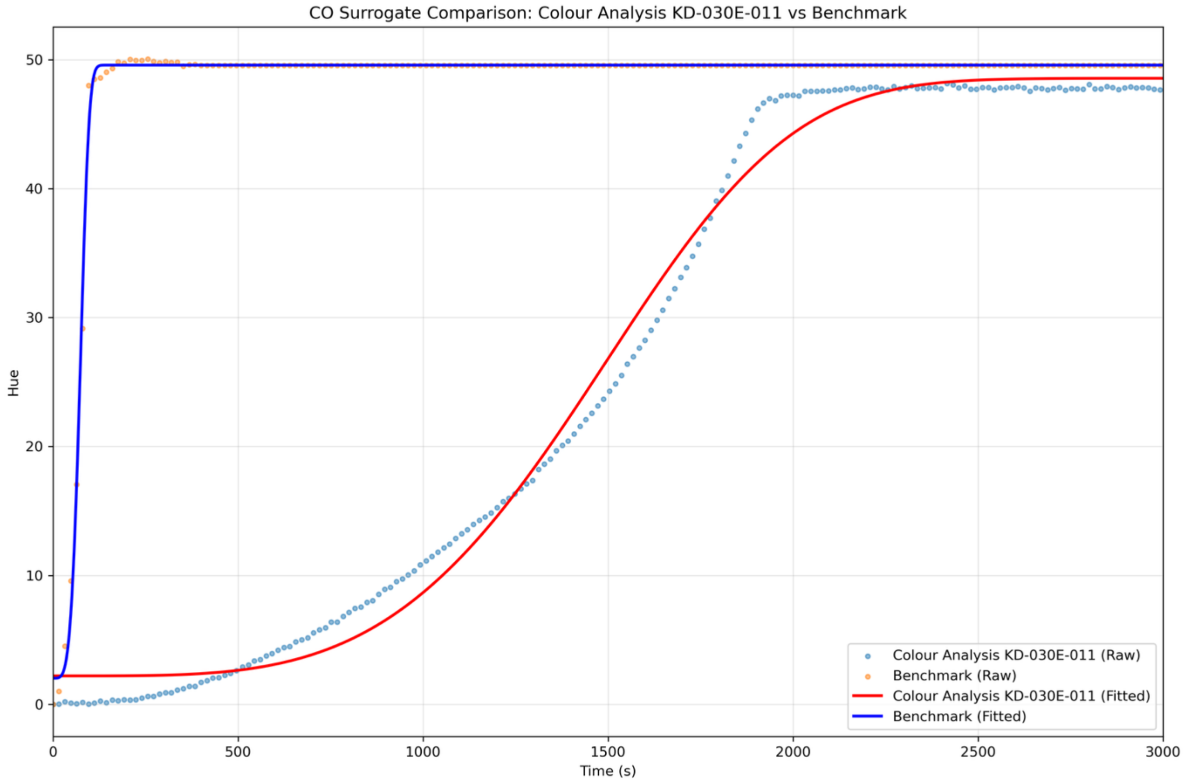

Figure S48: Weibull curves used to calculate surrogate score for KD-030E-011, COgen surrogate.

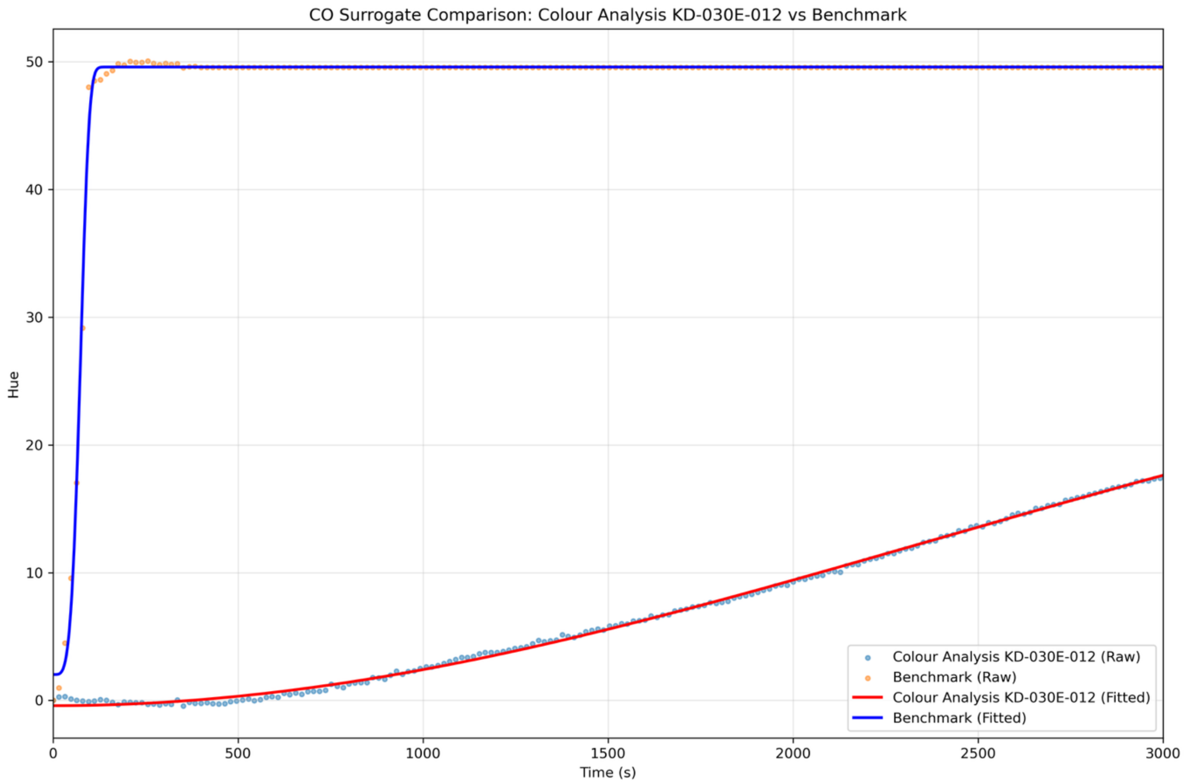

Figure S49: Weibull curves used to calculate surrogate score for KD-030E-012, COgen surrogate.

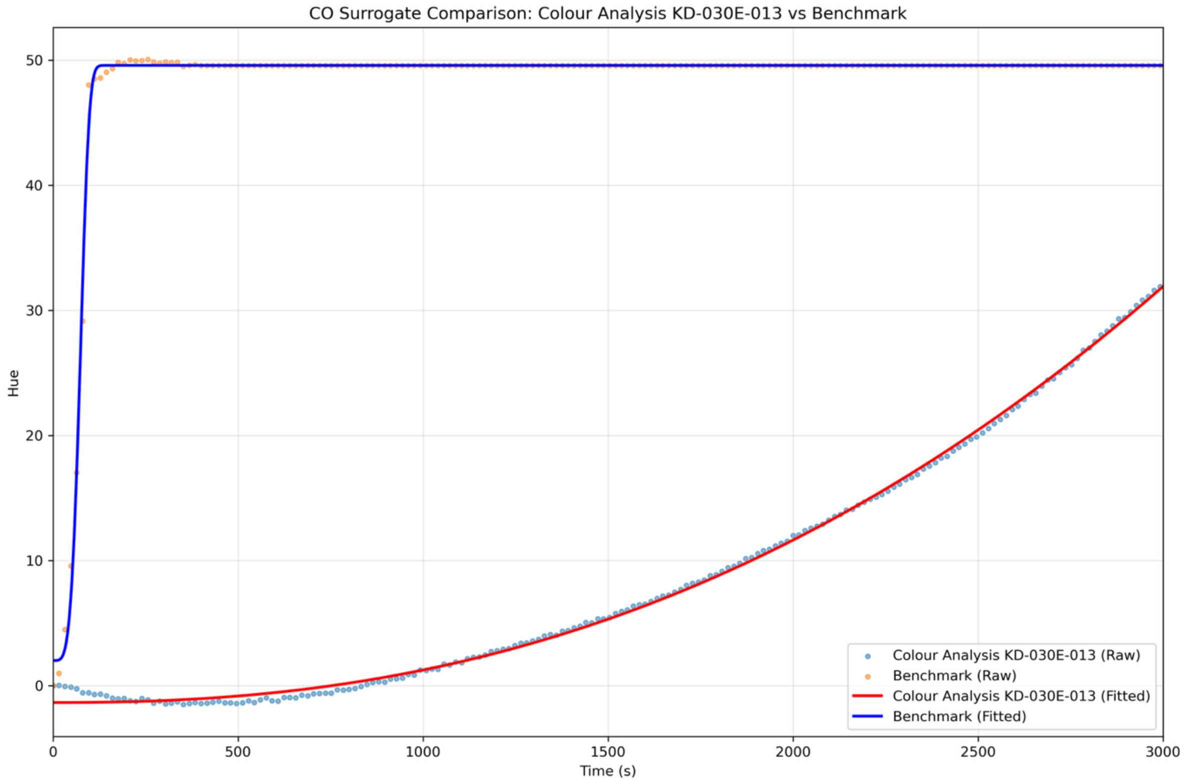

Figure S50: Weibull curves used to calculate surrogate score for KD-030E-013, COgen surrogate.

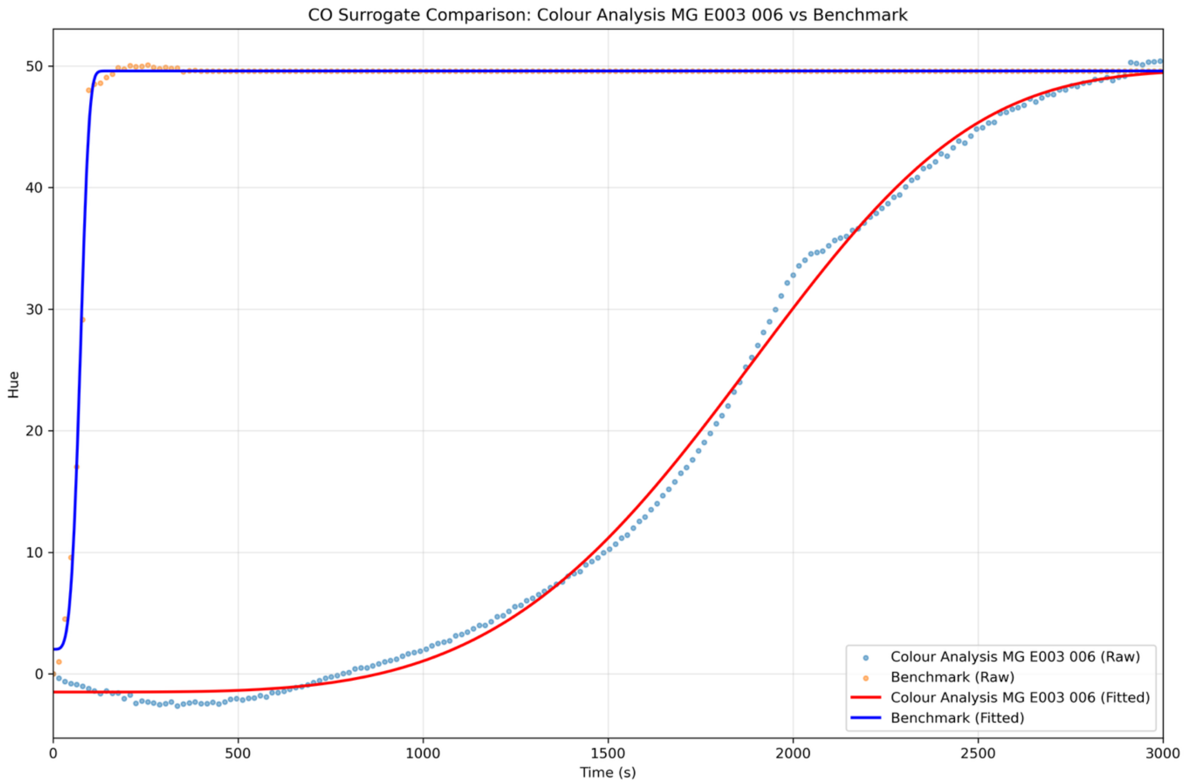

Figure S51: Weibull curves used to calculate surrogate score for MG E003 006, 2,4,6 trichlorophenyl formate surrogate.

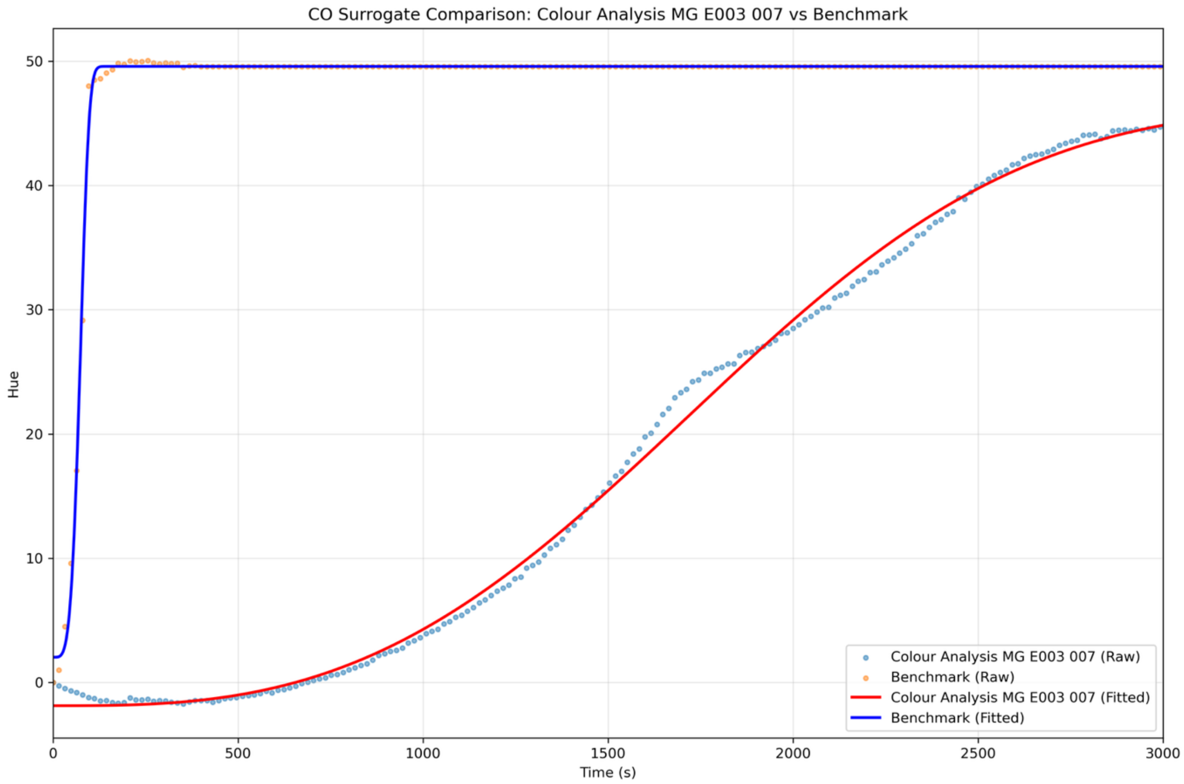

Figure S52: Weibull curves used to calculate surrogate score for MG E003 007, 2,4,6 trichlorophenyl formate surrogate.

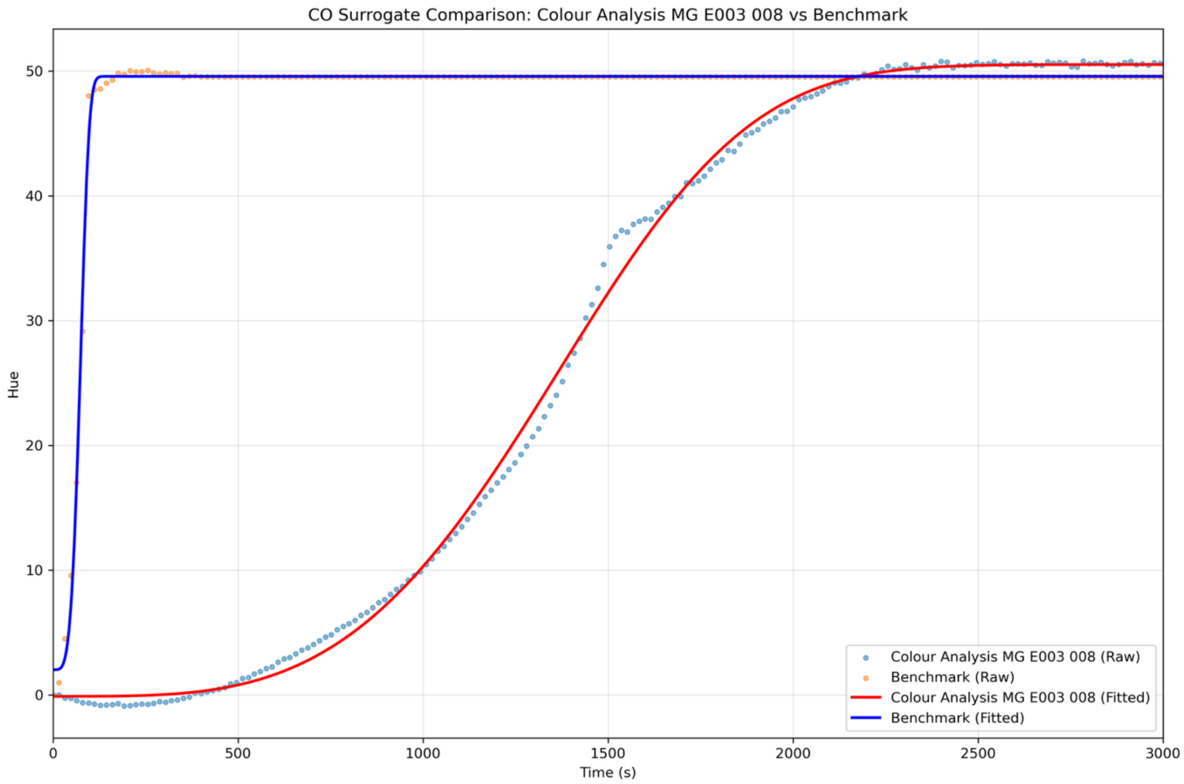

Figure S53: Weibull curves used to calculate surrogate score for MG E003 008, 2,4,6 trichlorophenyl formate surrogate.

4. Calibration of color and concentration

This calibration enables semi-quantitative estimation of sensor saturation but is not intended as a direct measure of absolute CO concentration.

4.1. Calibration details

See **section 1.10** for full experimental details.

Table S11: Kineticolor data used to create the video hue calibration. Hue was normalized by subtracting the hue value of vial 1 from all subsequent vials. i.e. for Vial 4, Normalized hue = 22.74-18.11 = 4.63

Vial	complex 1 (mmol)	complex 2 (mmol)	BTD (mmol)	Hue	Normalized hue
1	0.004	0	0	18.11	0.00
2	0.0036	0.0004	0.0004	19.63	1.52
3	0.0032	0.0008	0.0008	20.29	2.18
4	0.0028	0.0012	0.0012	22.74	4.63
5	0.0024	0.0016	0.0016	24.41	6.30
6	0.002	0.002	0.002	27.08	8.97
7	0.0016	0.0024	0.0024	29.72	11.61
8	0.0012	0.0028	0.0028	33.22	15.10
9	0.0008	0.0032	0.0032	39.06	20.94
10	0.0004	0.0036	0.0036	47.58	29.47
11	0	0.004	0.004	71.65	53.54

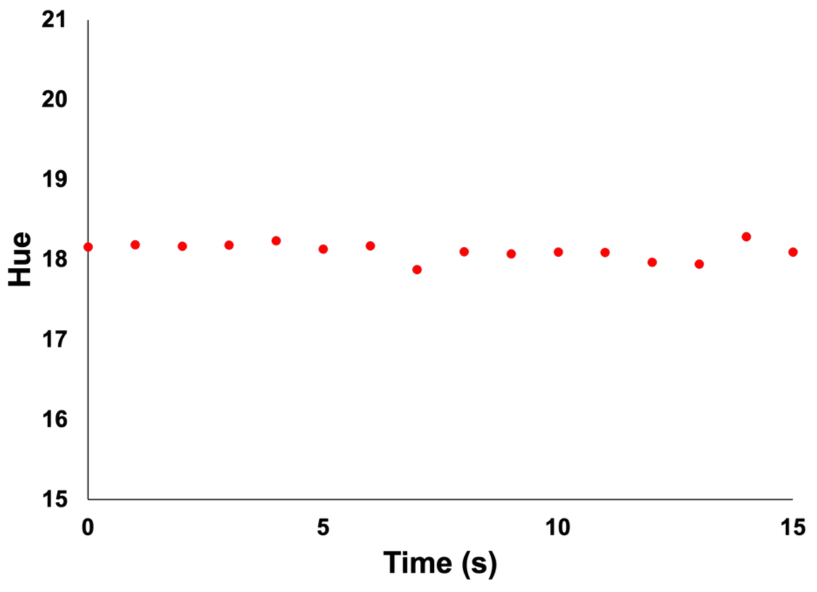

Figure S54: Kineticolor hue output from analysis of vial 1.

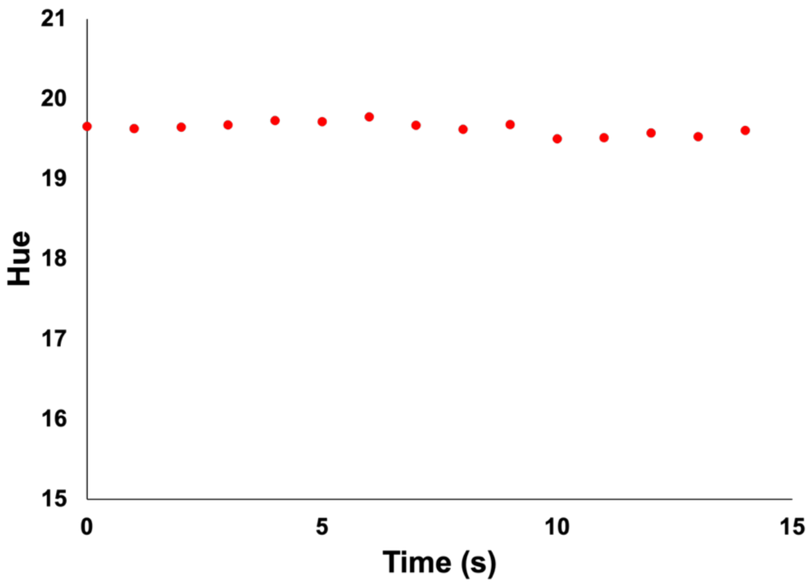

Figure S55: Kineticolor hue output from analysis of vial 2.

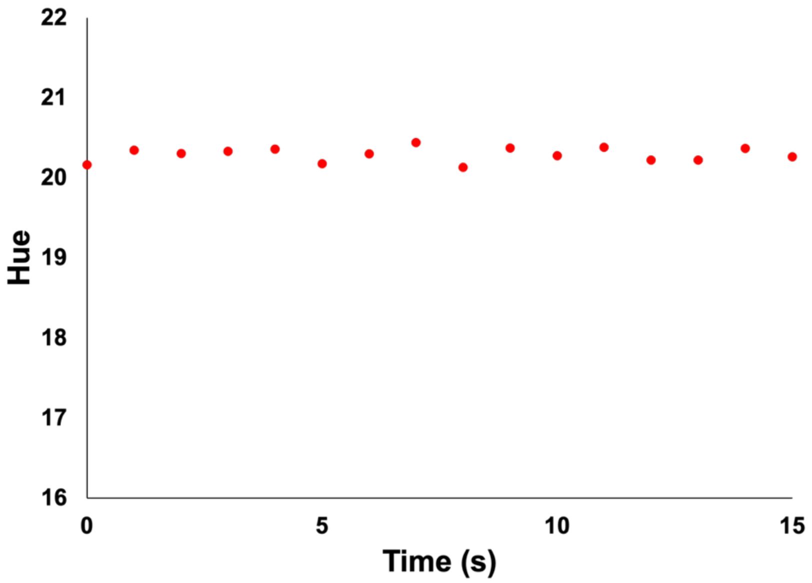

Figure S56:Kineticolor hue output from analysis of vial 3.

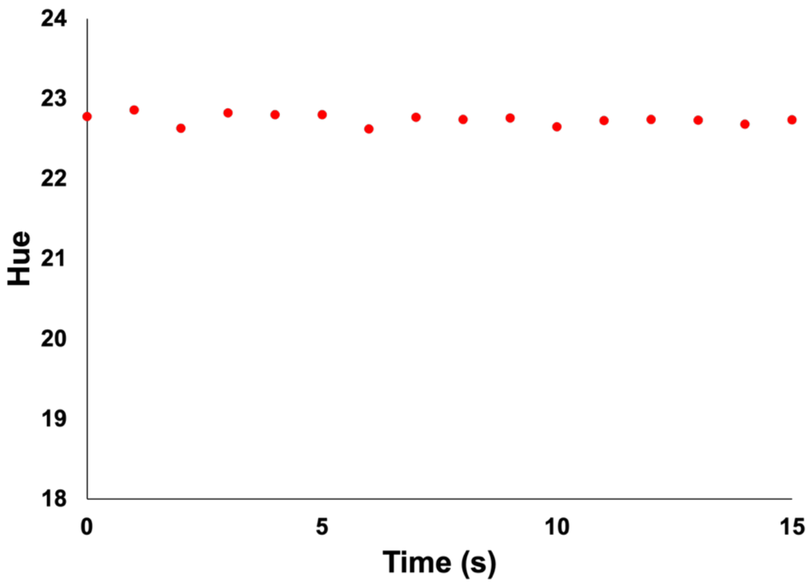

Figure S57: Kineticolor hue output from analysis of vial 4.

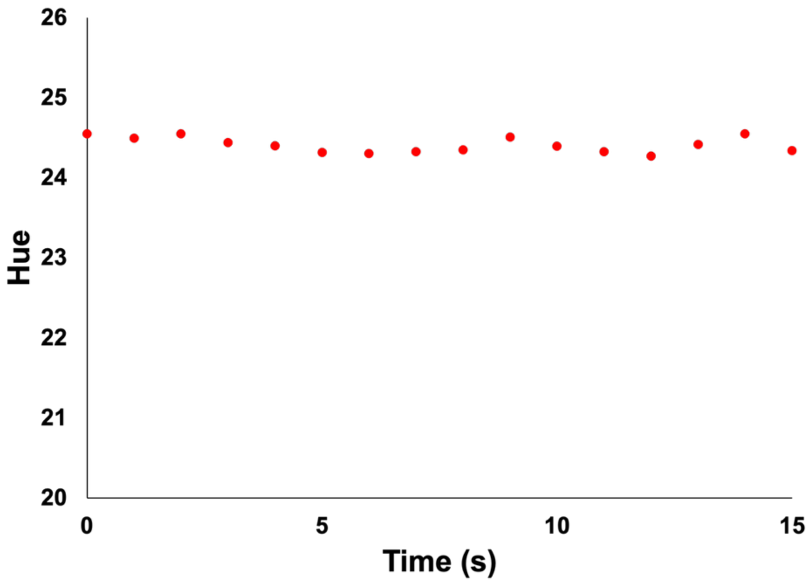

Figure S58: Kineticolor hue output from analysis of vial 5.

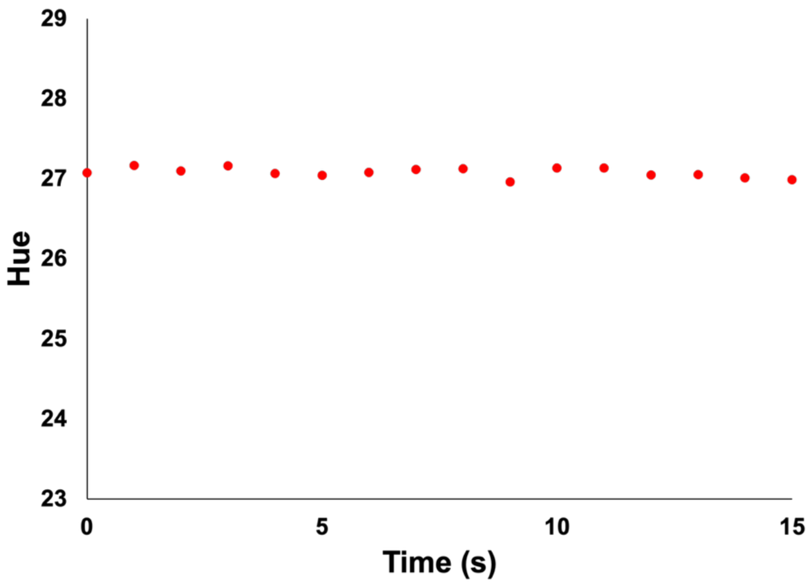

Figure S59: Kineticolor hue output from analysis of vial 6.

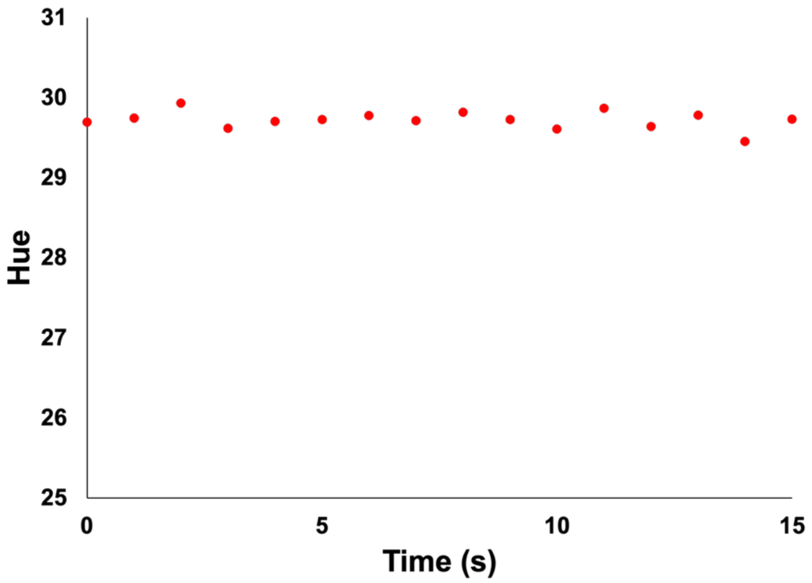

Figure S60:Kineticolor hue output from analysis of vial 7.

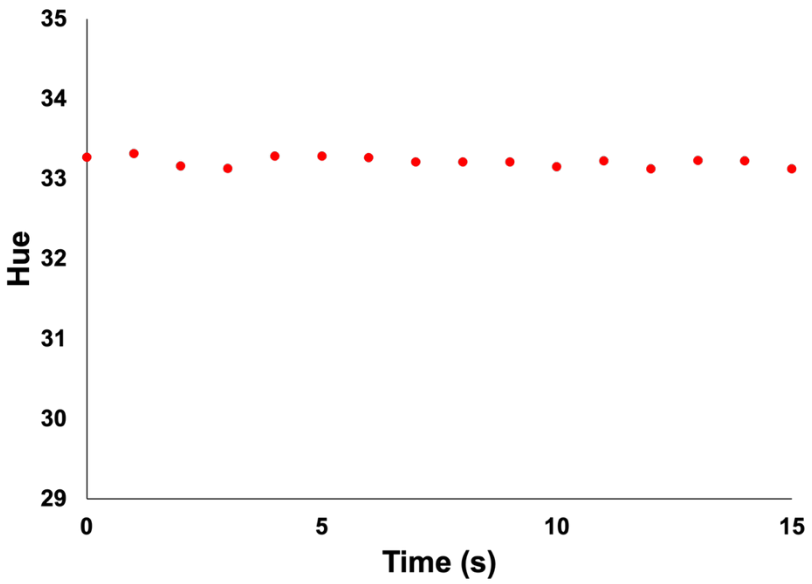

Figure S61: Kineticolor hue output from analysis of vial 8.

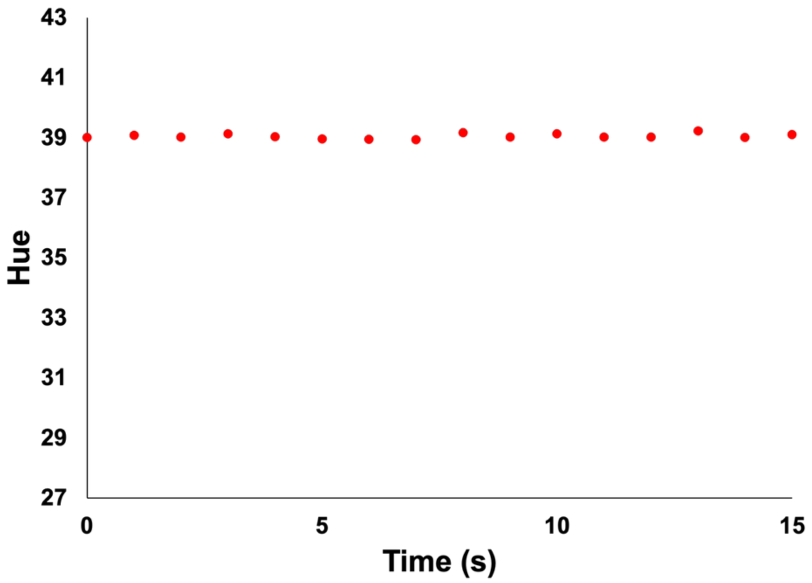

Figure S62: Kineticolor hue output from analysis of vial 9.

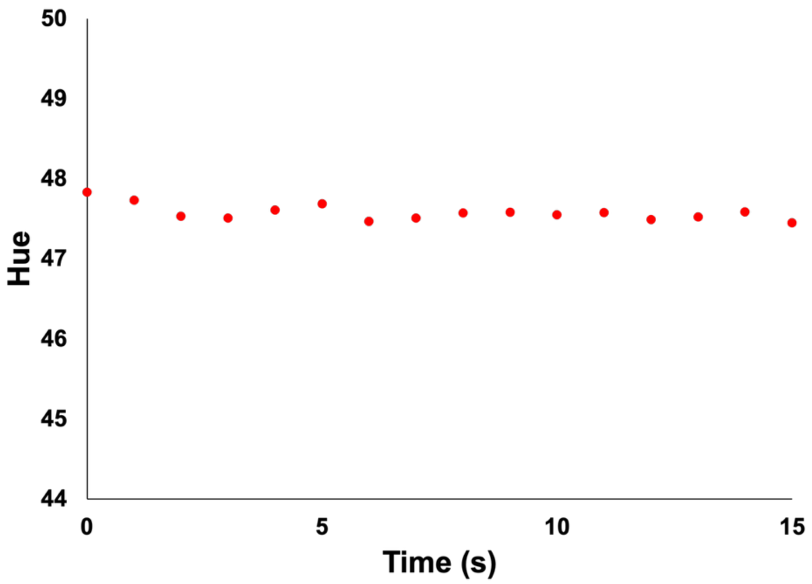

Figure S63:Kineticolor hue output from analysis of vial 10.

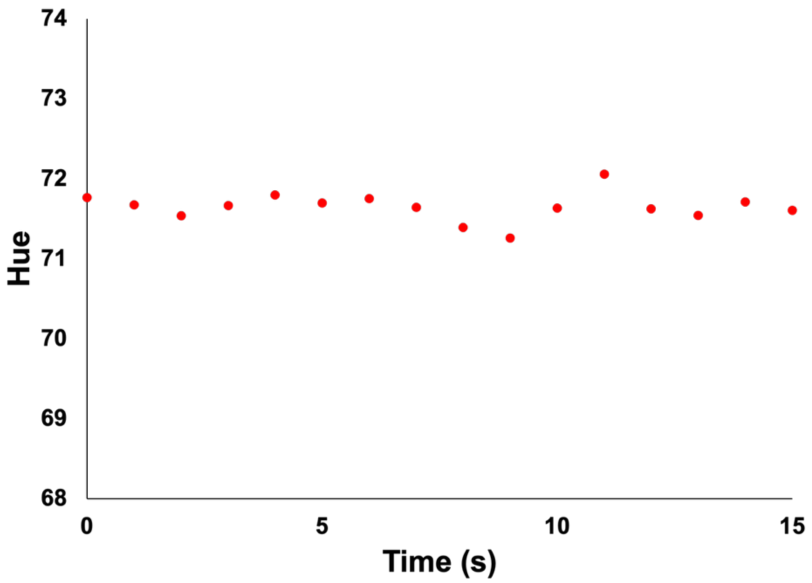

Figure S64: Kineticolor hue output from analysis of vial 11.

4.2. Normalized Kineticolor data for calibration studies

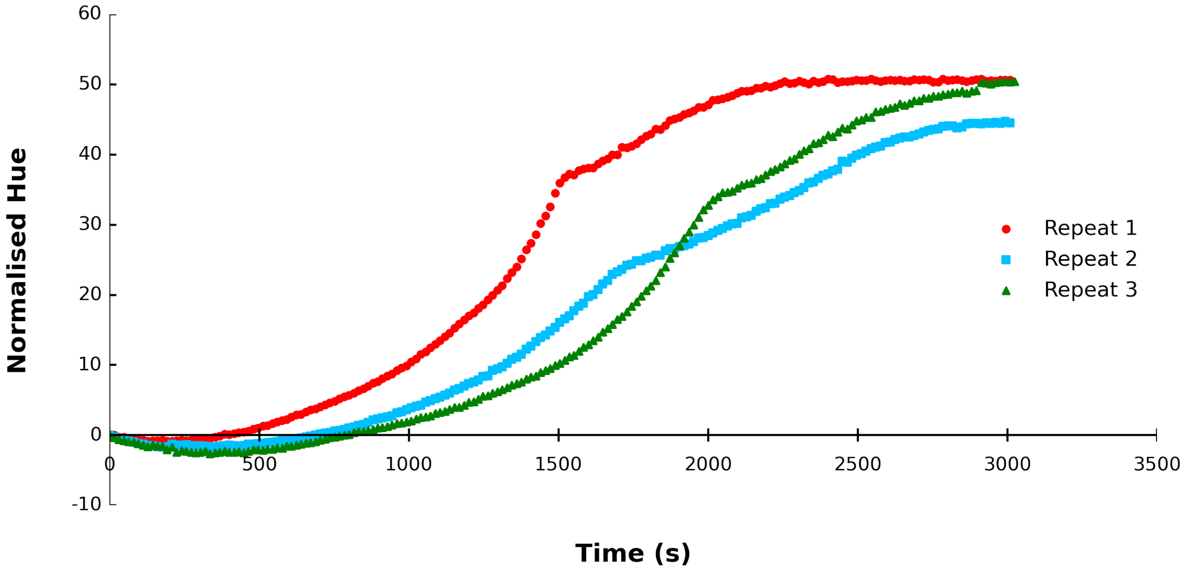

Figure S65: Normalized hue data of each repeat of 2,4,6 trichlorophenyl formate that was used in the color calibration study. Hue was normalized by subtracting the initial hue value from all subsequent values. Ensuring the data started at (0,0)

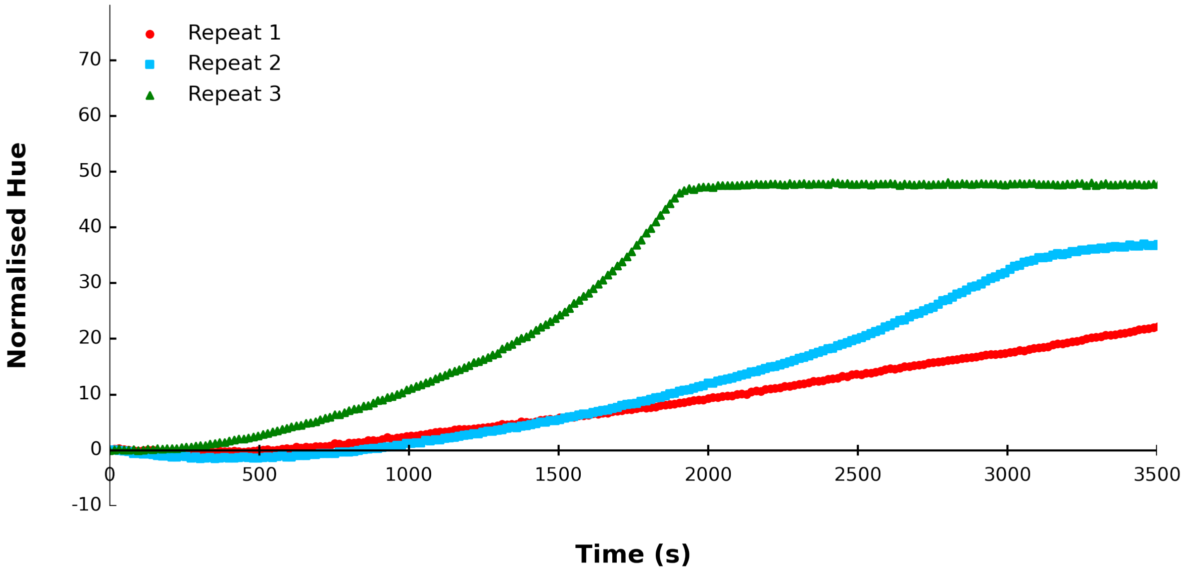

Figure S66: Normalized hue data of each repeat of COgen that was used in the color calibration study. Hue was normalized by subtracting the initial hue value from all subsequent values. Ensuring the data started at (0,0)

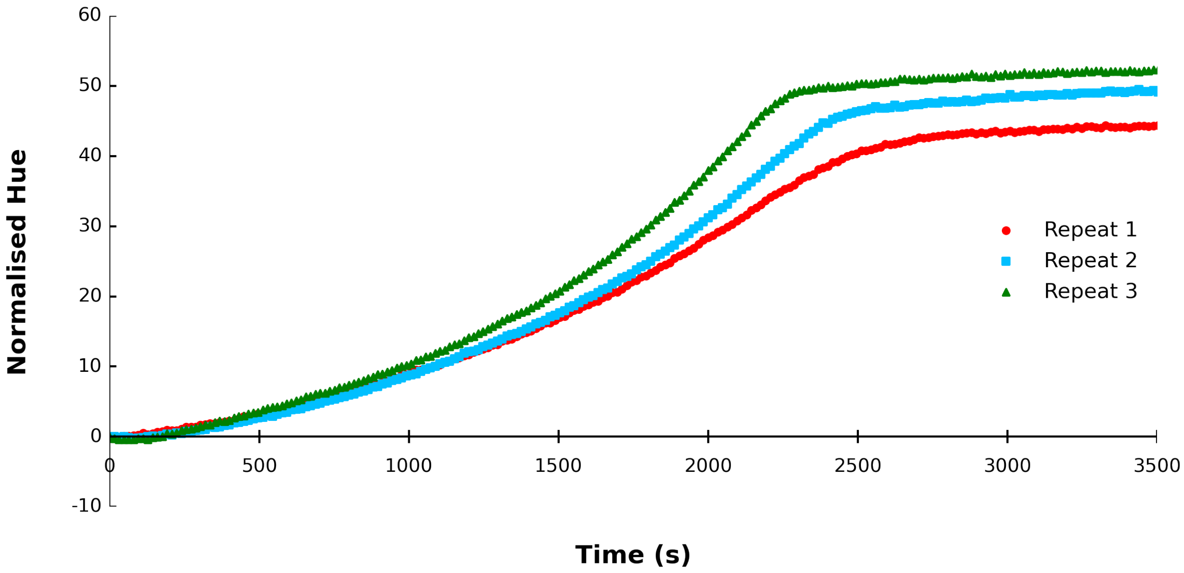

Figure S67: Normalized hue data of each repeat of $Cr(CO)_6$ that was used in the color calibration study. Hue was normalized by subtracting the initial hue value from all subsequent values. Ensuring the data started at (0,0)

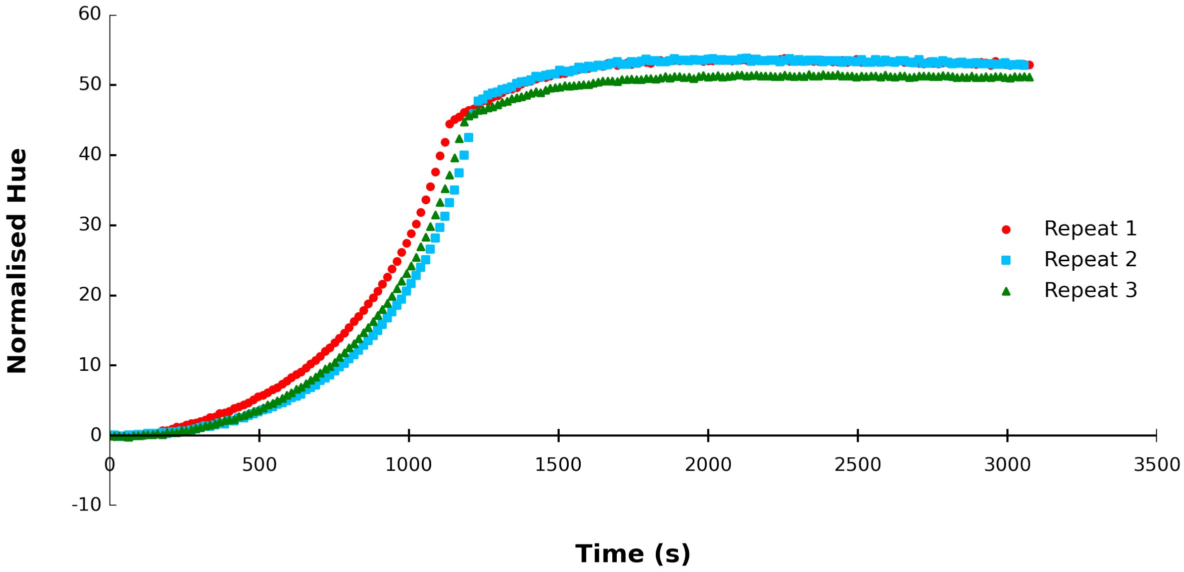

Figure S68: Normalized hue data of each repeat of formic acid that was used in the color calibration study. Hue was normalized by subtracting the initial hue value from all subsequent values. Ensuring the data started at (0,0)

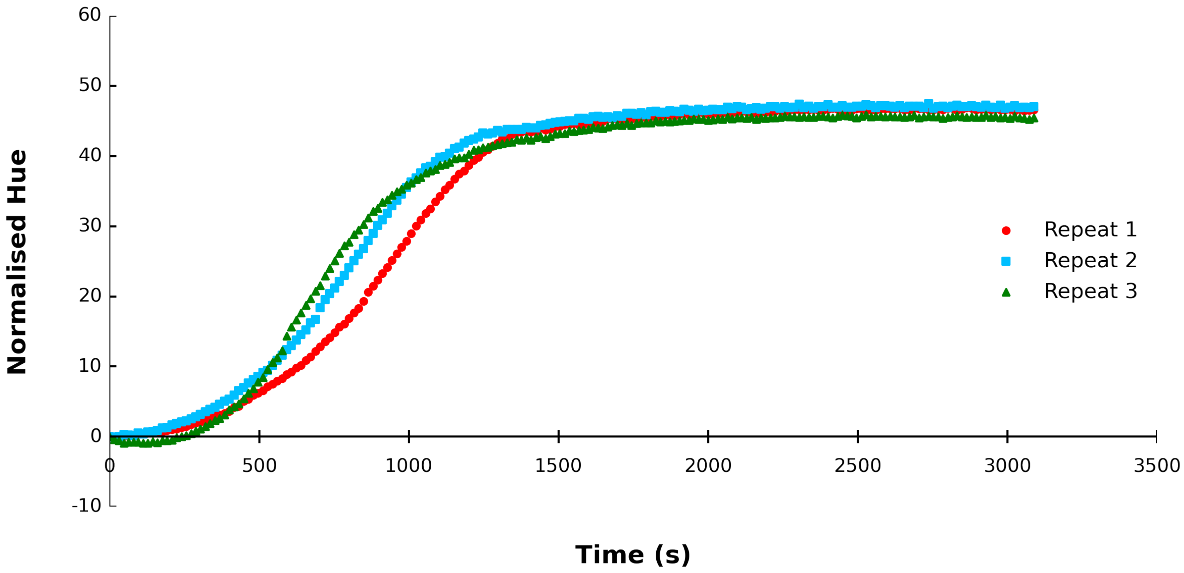

Figure S69: Normalized hue data of each repeat of $Mn(CO)_5Br$ that was used in the color calibration study. Hue was normalized by subtracting the initial hue value from all subsequent values. Ensuring the data started at (0,0)

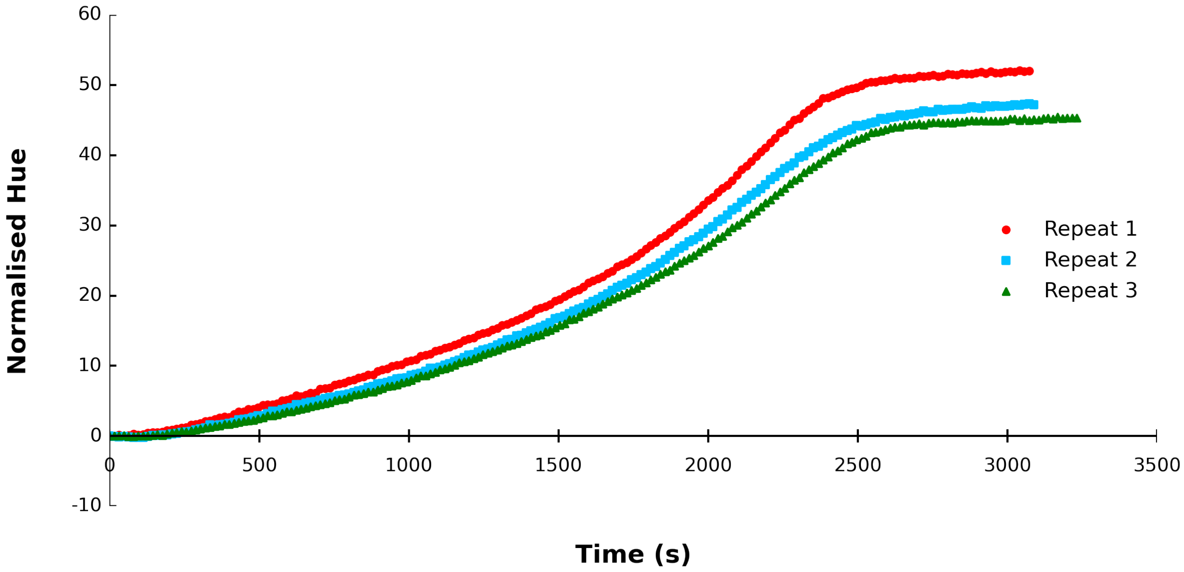

Figure S70: Normalized hue data of each repeat of $Mo(CO)_6$ that was used in the color calibration study. Hue was normalized by subtracting the initial hue value from all subsequent values. Ensuring the data started at (0,0)

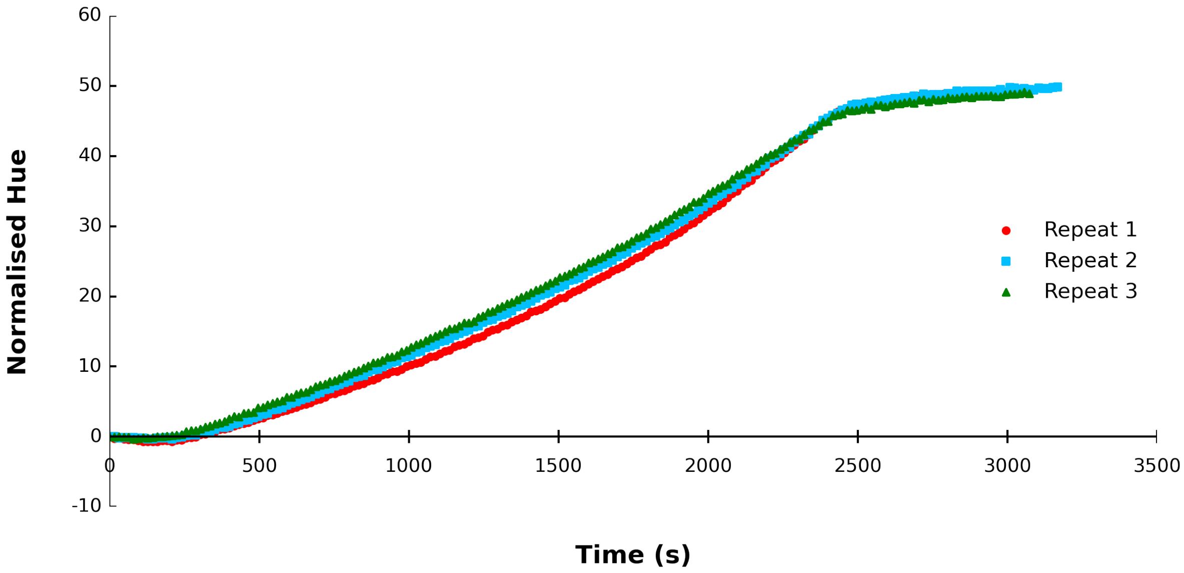

Figure S71: Normalized hue data of each repeat of N-formylsaccharin that was used in the color calibration study. Hue was normalized by subtracting the initial hue value from all subsequent values. Ensuring the data started at (0,0)

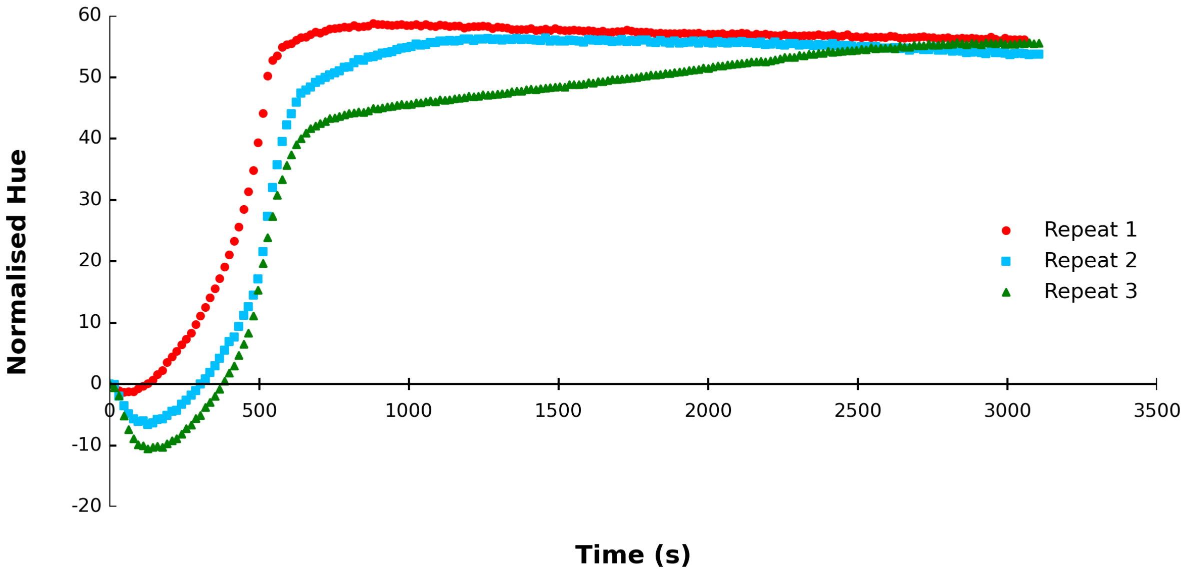

Figure S72: Normalized hue data of each repeat of oxalyl chloride that was used in the color calibration study. Hue was normalized by subtracting the initial hue value from all subsequent values. Ensuring the data started at (0,0). Diagnostic not scored.

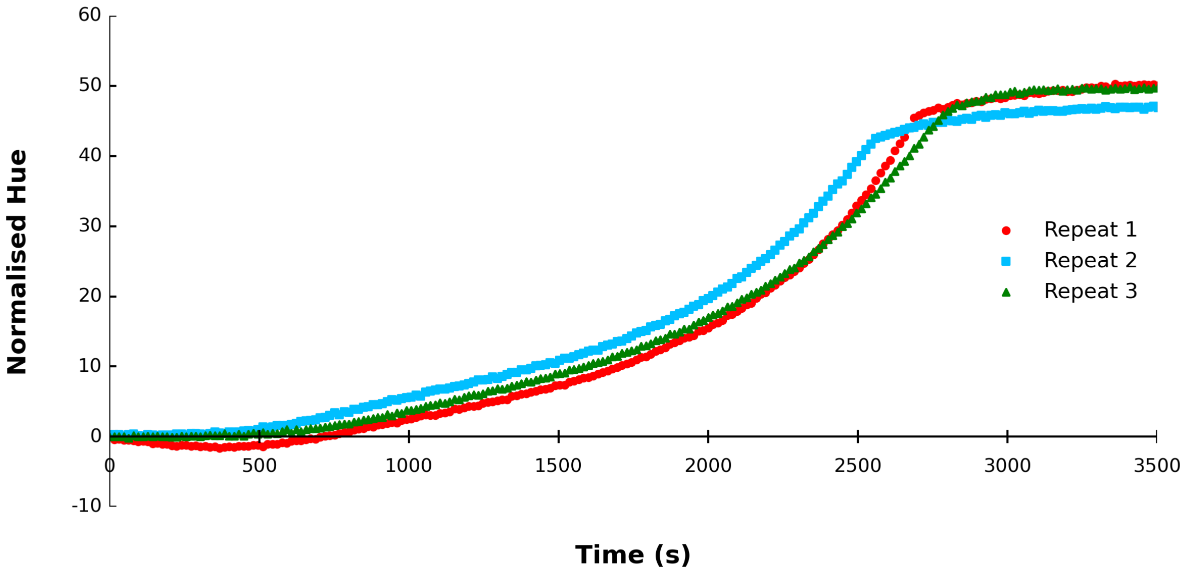

Figure S73: Normalized hue data of each repeat of SilaCO. Hue was normalized by subtracting the initial hue value from all subsequent values. Ensuring the data started at (0,0).

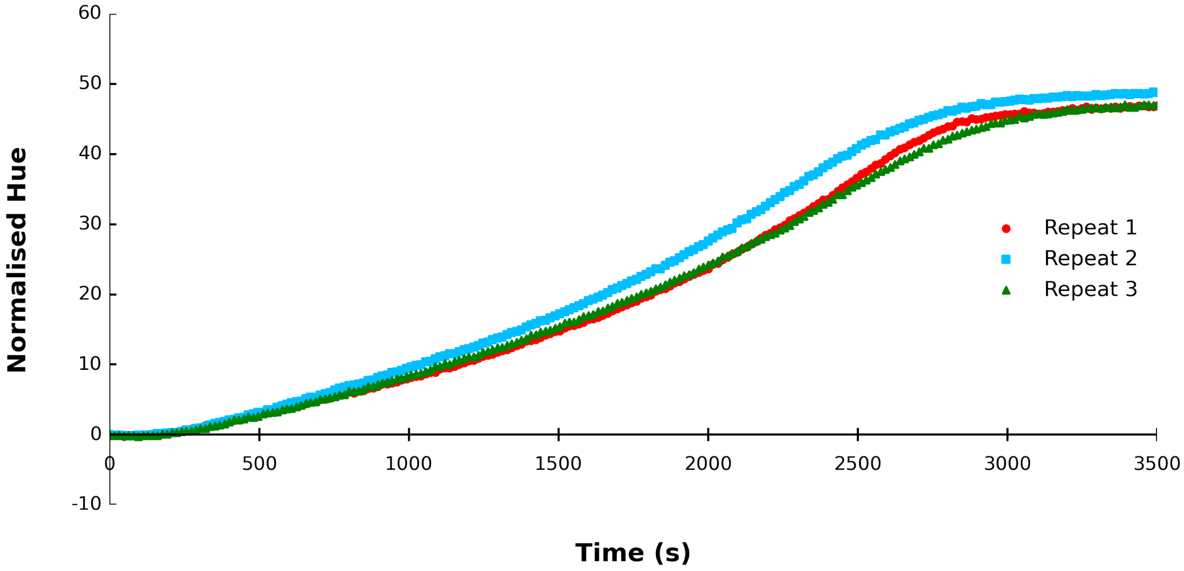

Figure S74: Normalized hue data of each repeat of $W(CO)_6$ that was used in the color calibration study. Hue was normalized by subtracting the initial hue value from all subsequent values. Ensuring the data started at (0,0).

5. Impact of reaction conditions on CO release

5.1. Surrogate Scores

Table S12 : Full details of calculated surrogate score of select surrogates under different reaction conditions. α is defined as the speed component and is a measure of how quickly the surrogate reaches its maximum rate of hue change compared to the benchmark. β is the plateau time component which measures how quickly the surrogate reaches its plateau compared to the benchmark. γ is the magnitude component and measures the total hue change of the surrogate compared to the benchmark.

Surrogate system	File Name	Score A	Std Dev	α	β	γ
800 RPM $Mo(CO)_6$	Color Analysis KD-017E-001	1.02	0.20	0.17	0.17	1.19
	Color Analysis KD-017E-002	0.93		0.14	0.15	1.25
	Color Analysis KD-017E-003	0.64		0.06	0.05	1.04
$Mo(CO)_6$ reduced base	Color Analysis KD-018E-001	0.39	0.03	0.00	0.00	5968.90
	Color Analysis KD-018E-002	0.34		0.00	0.00	8736.27
	Color Analysis KD-018E-003	0.33		0.00	0.00	8441.93
SilaCO with KF/18-C-6	Color Analysis KD-020E-001	0.60	0.12	0.05	0.06	1.03
	Color Analysis KD-020E-002	0.82		0.09	0.11	1.09
	Color Analysis KD-020E-003	0.80		0.10	0.12	1.05
COgen with $Pd_2(dba)_3$	Color Analysis KD-030E-004	0.53	0.07	0.05	0.04	1.03
	Color Analysis KD-030E-009	0.42		0.03	0.02	1.79
	Color Analysis KD-030E-010	0.41		0.03	0.01	2.68
2,4,6 trichlorophenyl formate in toluene	Color Analysis MG E015 001	0.71	0.01	0.11	0.12	0.92
	Color Analysis MG E015 002	0.70		0.09	0.08	1.02
	Color Analysis MG E015 003	0.69		0.08	0.08	1.07
2,4,6 trichlorophenyl formate in DMF	Color Analysis MG E015 004	1.15	0.25	inf	0.05	1.18
	Color Analysis MG E015 005	0.88		0.20	0.12	1.12
	Color Analysis MG E015 006	0.65		0.20	0.10	0.87
2,4,6 trichlorophenyl formate triggered with DBU	Color Analysis MG E015 007	0.4	0.2	0.1	0.0	1.0
	Color Analysis MG E015 009	0.7		0.1	0.0	1.1
	Color Analysis MG E015 010	0.6		0.1	0.0	1.2

5.2. Kineticolor data

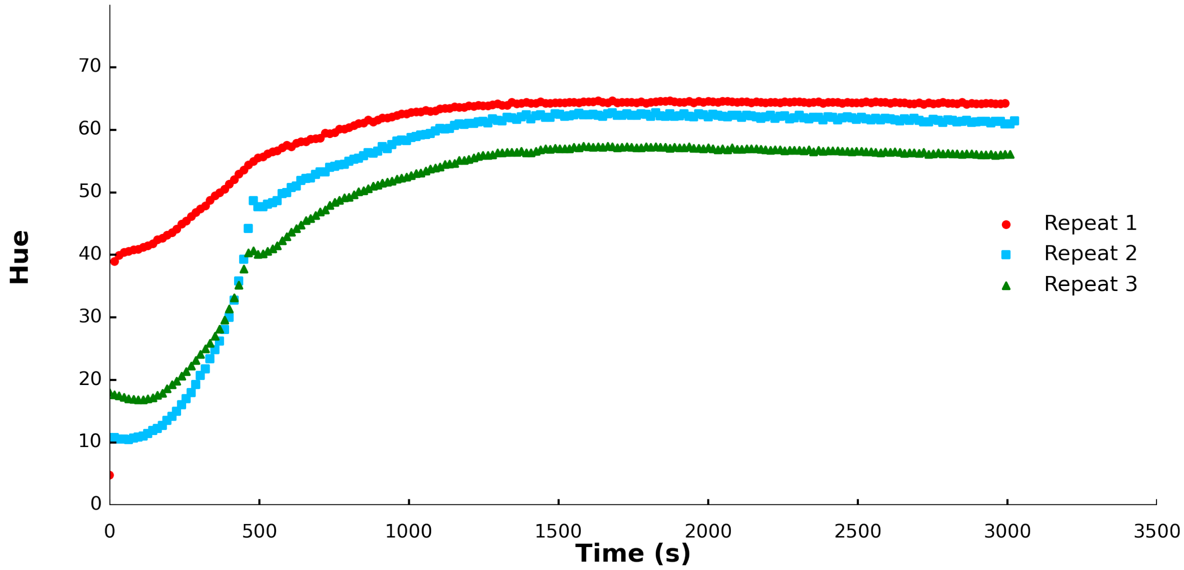

Figure S75: Three repeats of Kineticolor analysis of complex 1 when exposed to CO produced from 2,4,6 trichlorophenyl formate in DMF.

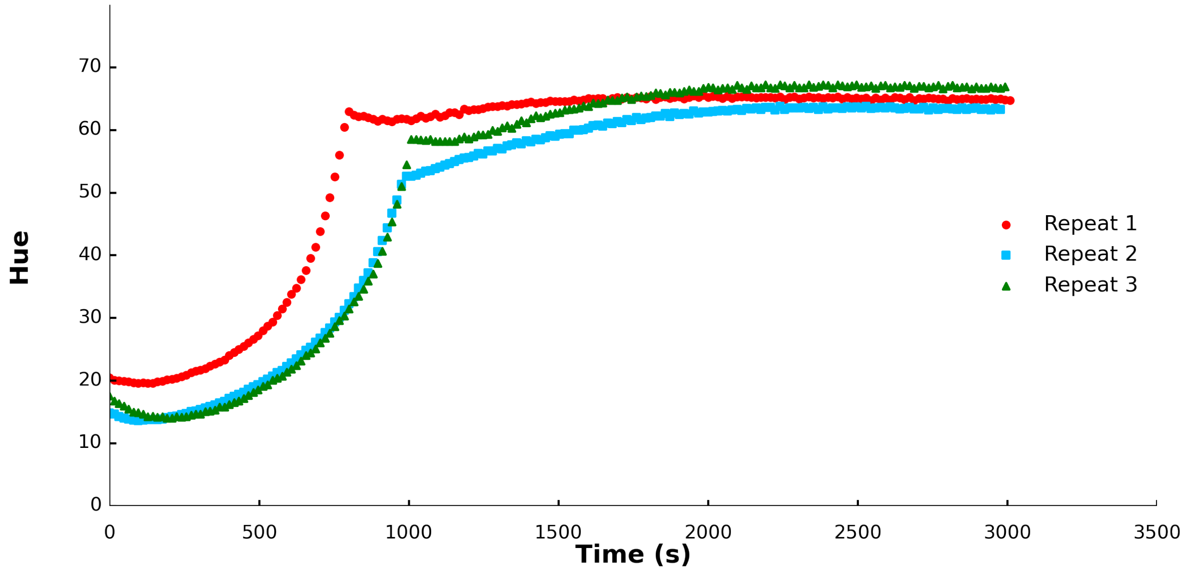

Figure S76: Three repeats of Kineticolor analysis of complex 1 when exposed to CO produced from 2,4,6 trichlorophenyl formate in toluene.

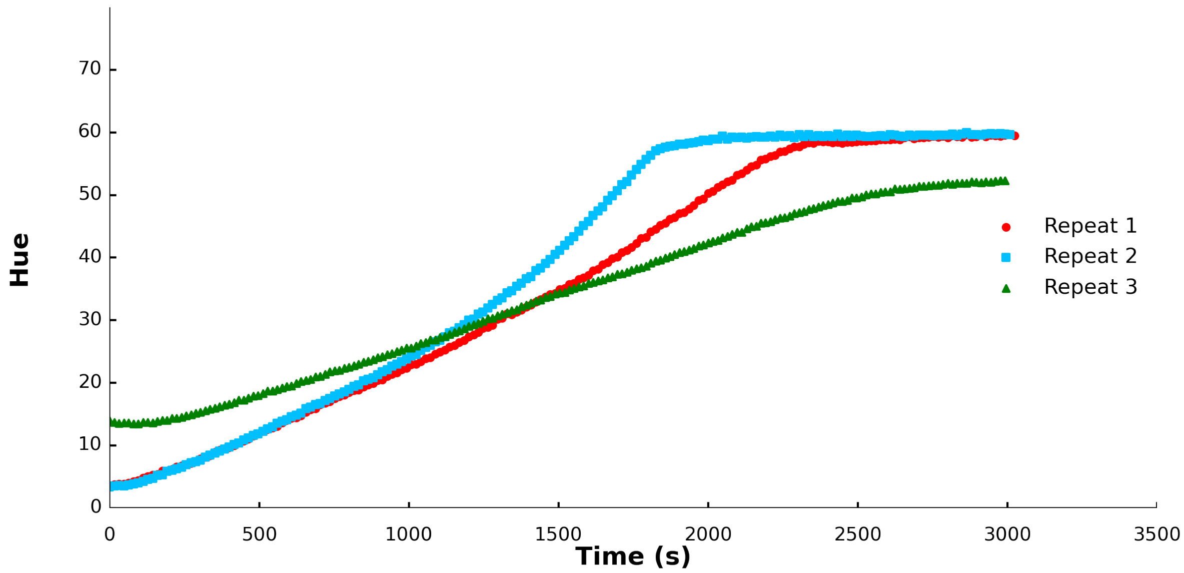

Figure S77: Three repeats of Kineticolor analysis of complex 1 when exposed to CO produced from 2,4,6 trichlorophenyl formate, CO release was triggered by DBU.

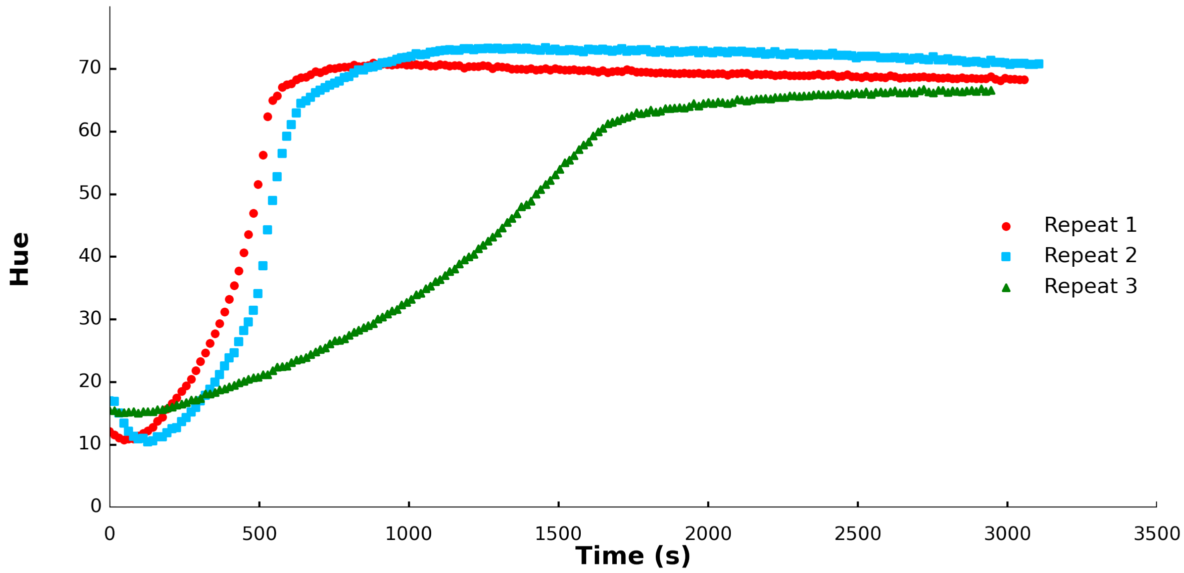

Figure S78: Three repeats of Kineticolor analysis of complex 1 when exposed to CO produced from $Mo(CO)_6$ stirred at 800 RPM.

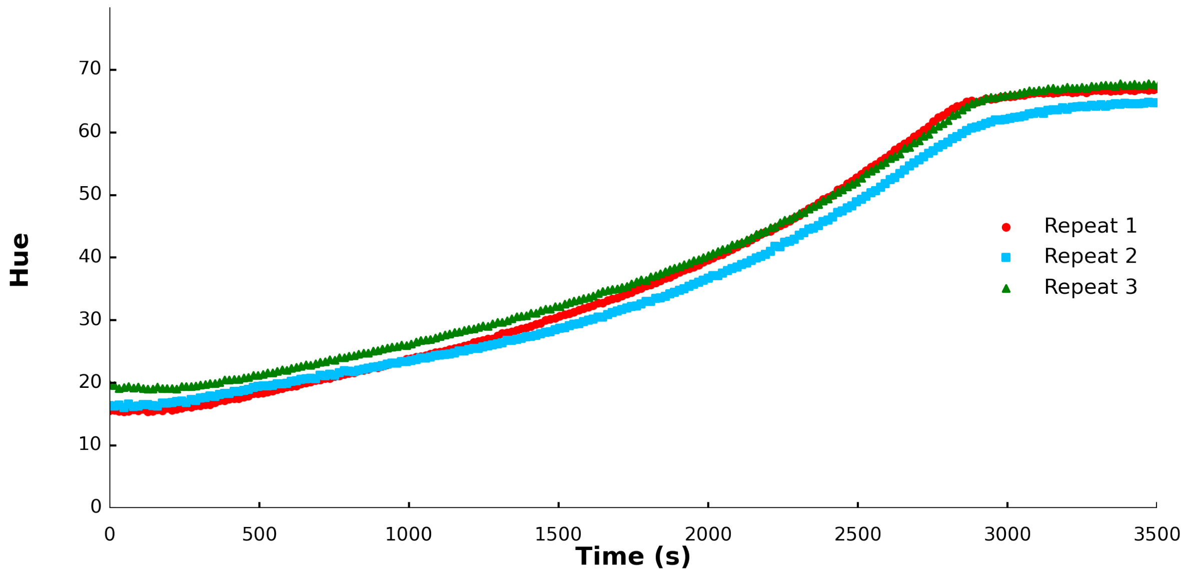

Figure S79: Three repeats of Kineticolor analysis of complex 1 when exposed to CO produced from $Mo(CO)_6$ triggered with a reduced volume of DBU (4.6 μL, 0.003 mmol).

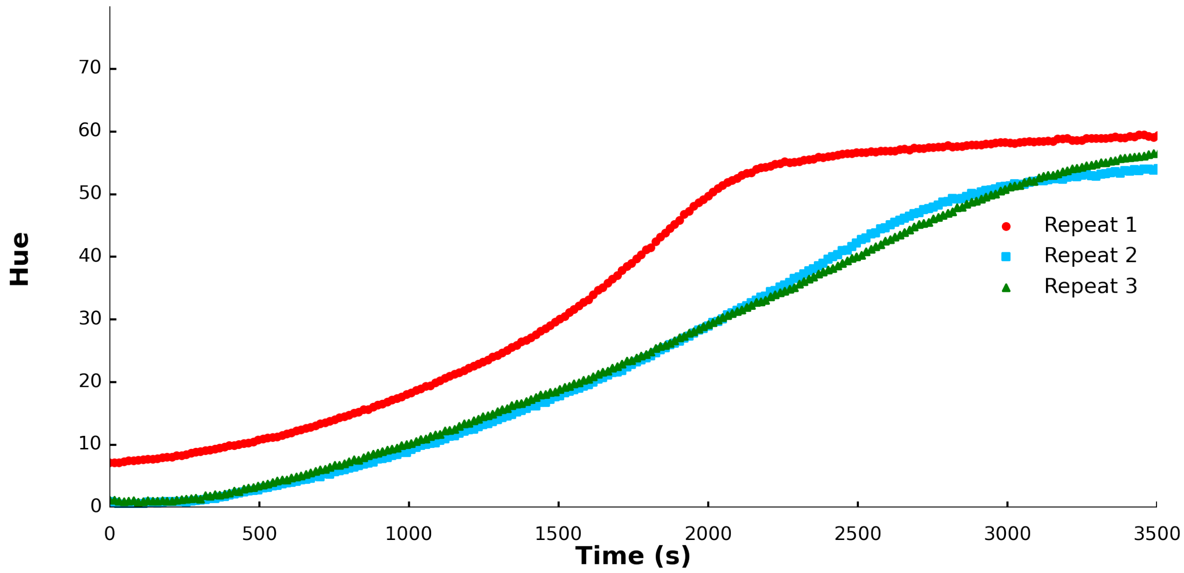

Figure S80: Three repeats of Kineticolor analysis of complex 1 when exposed to CO produced from COgen with $Pd_2(dba)_3$ as the source of Pd(0).

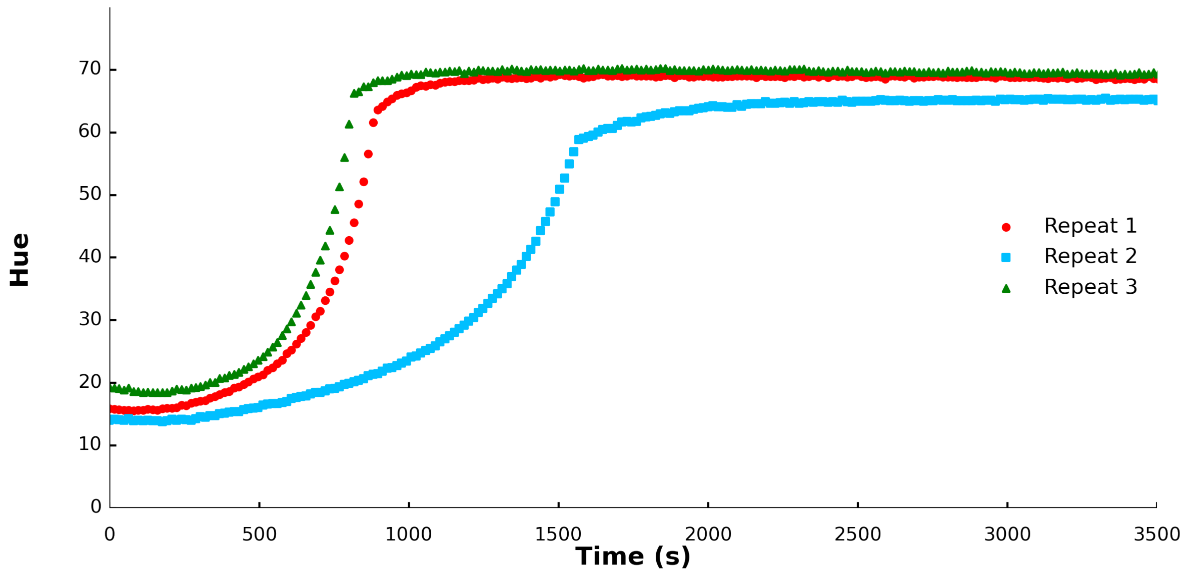

Figure S81: Three repeats of Kineticolor analysis of complex 1 when exposed to CO produced from SilaCO using the modified KF/18-C-6 trigger system.

As with Figures S18 and S19, the inter-replicate variability observed in Figures S78, S80, and S81 is attributed to the sensitivity of these particular surrogate systems to minor variations in reagent handling, stirring consistency, and COware sealing between independent experiments. These observations underscore the importance of reporting replicate data with appropriate error analysis, as performed throughout this study.

5.3. Weibull Data

Table S13: Full details of the Weibull parameters for each analysis carried out. A-scale parameter, b – shape parameter, c – Plateau parameter and d – offset parameter.

Surrogate	File Name	a	b	c	d
800 RPM $Mo(CO)_6$	Color Analysis KD-017E-001	472.47	4.40	56.39	2.0
	Color Analysis KD-017E-002	560.87	4.82	59.45	1.0
	Color Analysis KD-017E-003	1357.70	3.02	49.32	-4.3
$Mo(CO)_6$ reduced base	Color Analysis KD-018E-001	297514.52	1.87	283876.94	1.6
	Color Analysis KD-018E-002	237714.31	2.08	415491.04	0.8
	Color Analysis KD-018E-003	302284.42	1.96	401492.27	1.4
SilaCO with KF/18-C-6	Color Analysis KD-020E-001	1449.37	5.43	49.03	0.1
	Color Analysis KD-020E-002	827.19	6.59	51.69	1.8
	Color Analysis KD-020E-003	756.94	7.51	50.10	1.3
COgen with $Pd_2(dba)_3$	Color Analysis KD-030E-004	1751.57	3.30	49.18	0.4
	Color Analysis KD-030E-009	3077.02	2.08	85.09	2.3
	Color Analysis KD-030E-010	4463.09	1.71	127.67	-0.0
2,4,6 trichlorophenyl formate in toluene	Color Analysis MG E015 001	721.38	5.92	43.57	-0.6
	Color Analysis MG E015 002	957.07	3.32	48.66	0.5
	Color Analysis MG E015 003	967.18	4.10	51.08	-1.0
	Color Analysis MG E015 004	/	/	/	-2.9

2,4,6 trichlorophenyl formate in DMF	Color Analysis MG E015 005	507.69	2.14	53.09	6.4
	Color Analysis MG E015 006	577.12	1.85	41.49	-2.3
2,4,6 trichlorophenyl formate triggered by DBU	Color Analysis MG E015 007	2052.02	1.70	46.78	-3.0
	Color Analysis MG E015 009	1432.19	2.79	53.41	-0.4
	Color Analysis MG E015 010	1730.17	2.09	57.96	3.9

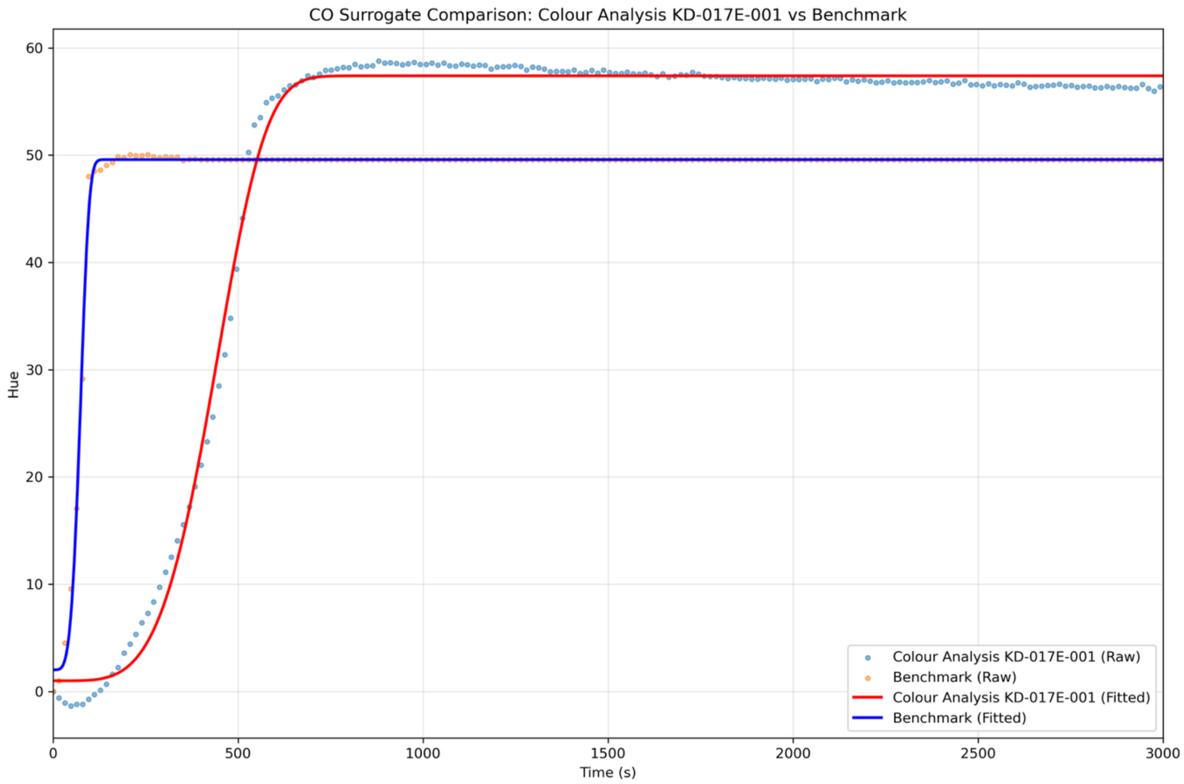

Figure S82: Weibull curves used to calculate surrogate score for KD-017E-001, $Mo(CO)_6$ surrogate at 800 RPM.

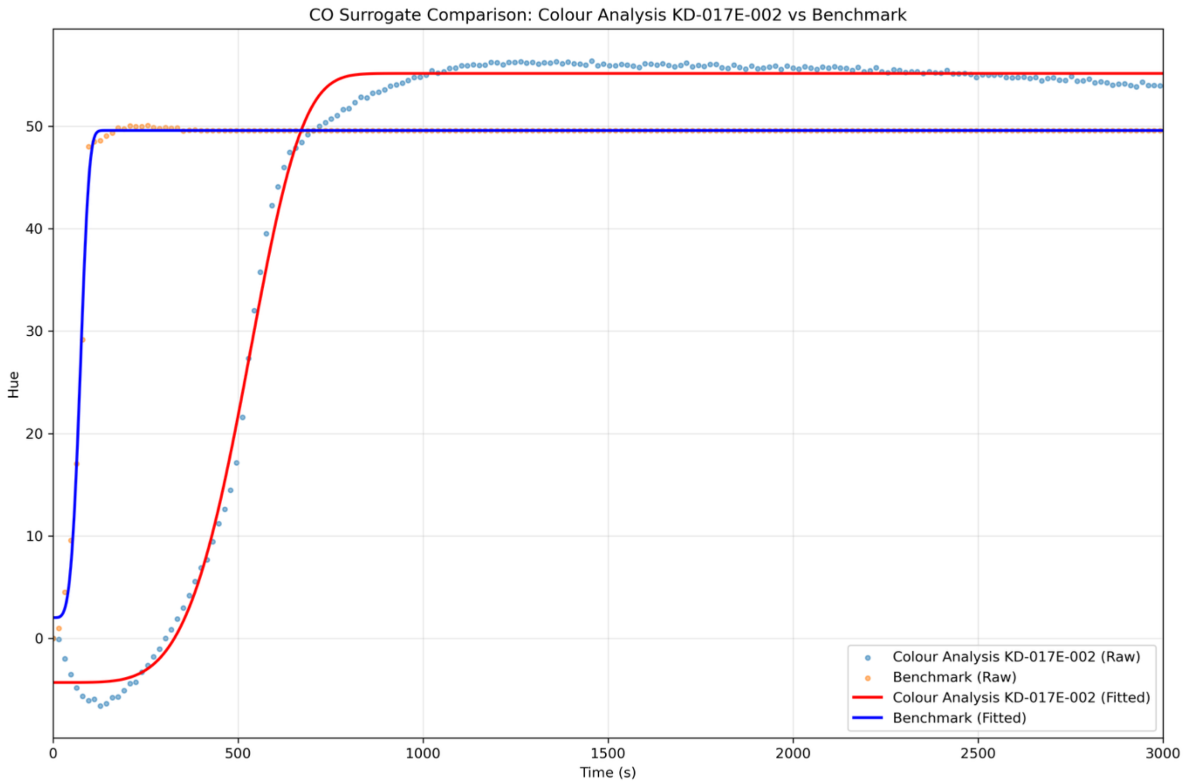

Figure S83: Weibull curves used to calculate surrogate score for KD-017E-002, $Mo(CO)_6$ surrogate at 800 RPM.

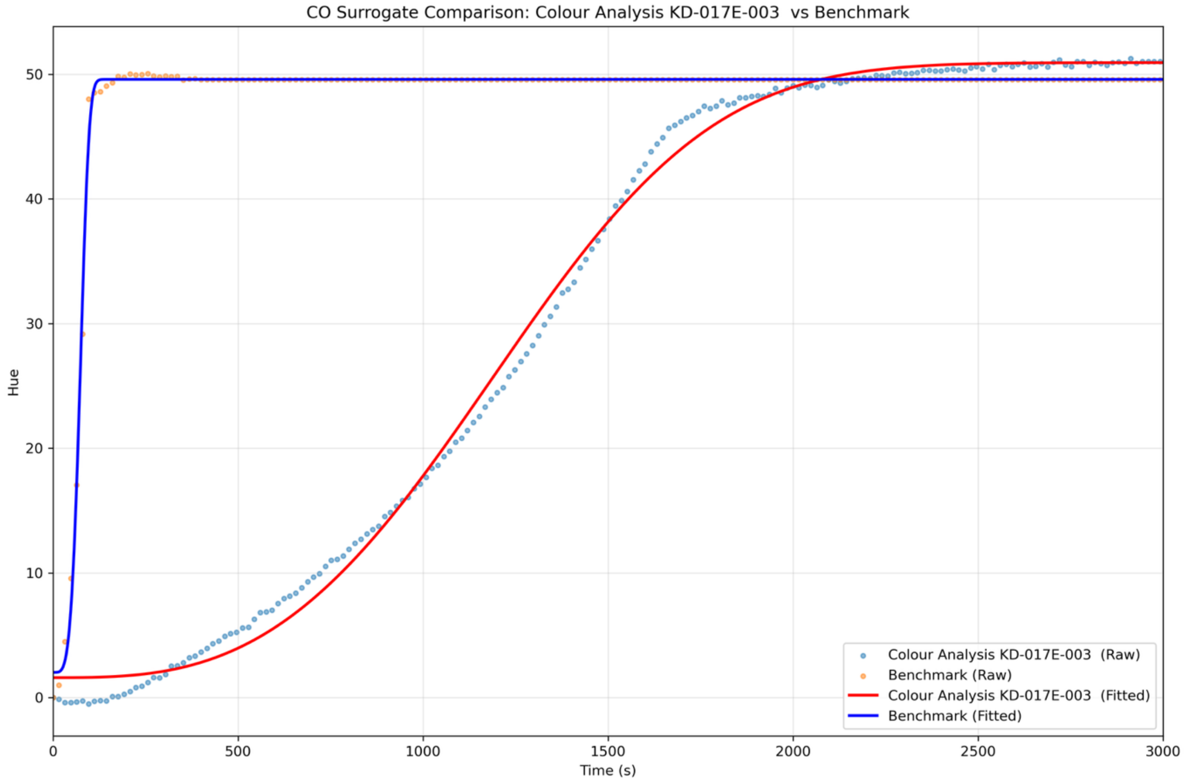

Figure S84: Weibull curves used to calculate surrogate score for KD-017E-001, $Mo(CO)_6$ surrogate at 800 RPM.

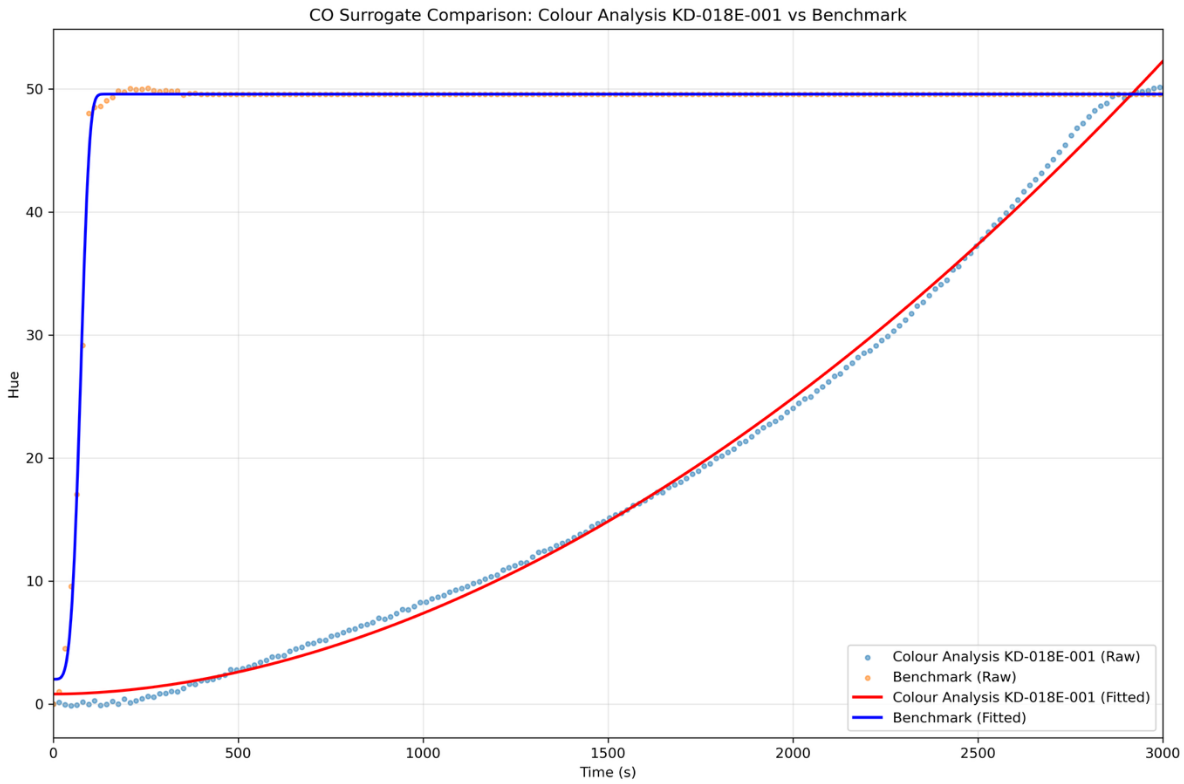

Figure S85: Weibull curves used to calculate surrogate score for KD-018E-001, $Mo(CO)_6$ surrogate with reduced base volume.

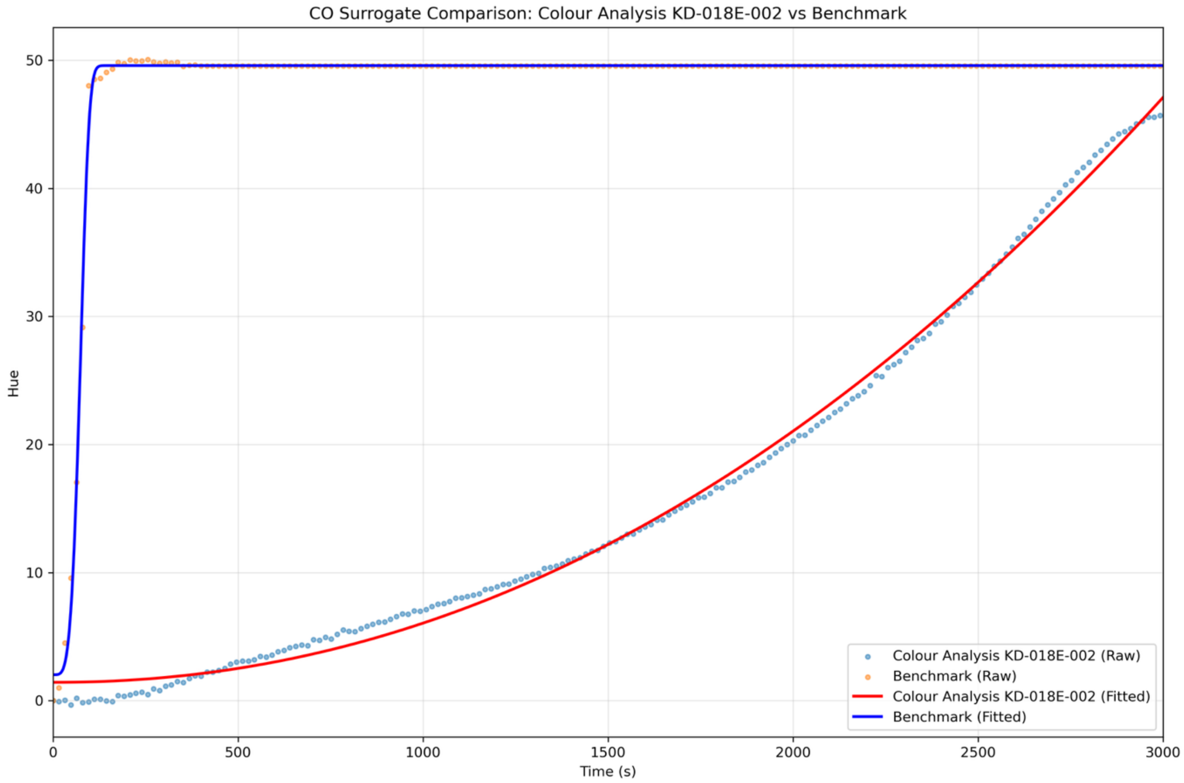

Figure S86: Weibull curves used to calculate surrogate score for KD-018E-002, $Mo(CO)_6$ surrogate with reduced base volume.

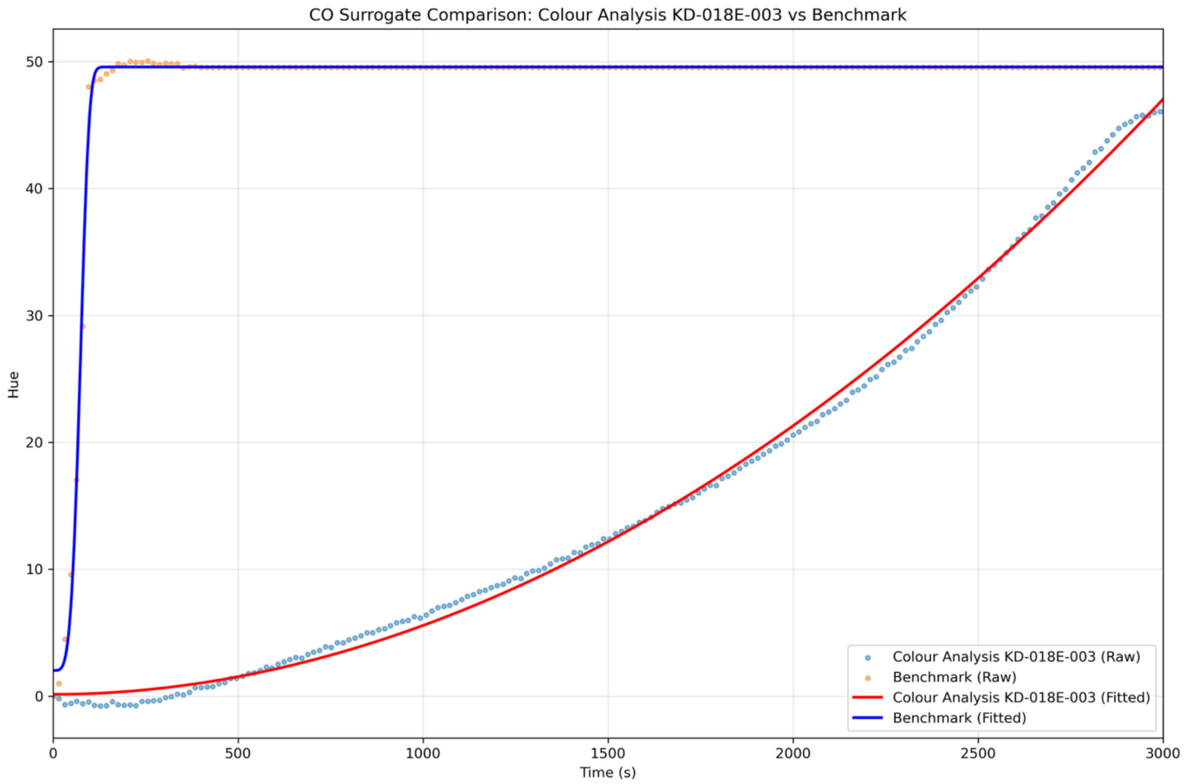

Figure S87: Weibull curves used to calculate surrogate score for KD-018E-003, $Mo(CO)_6$ surrogate with reduced base volume.

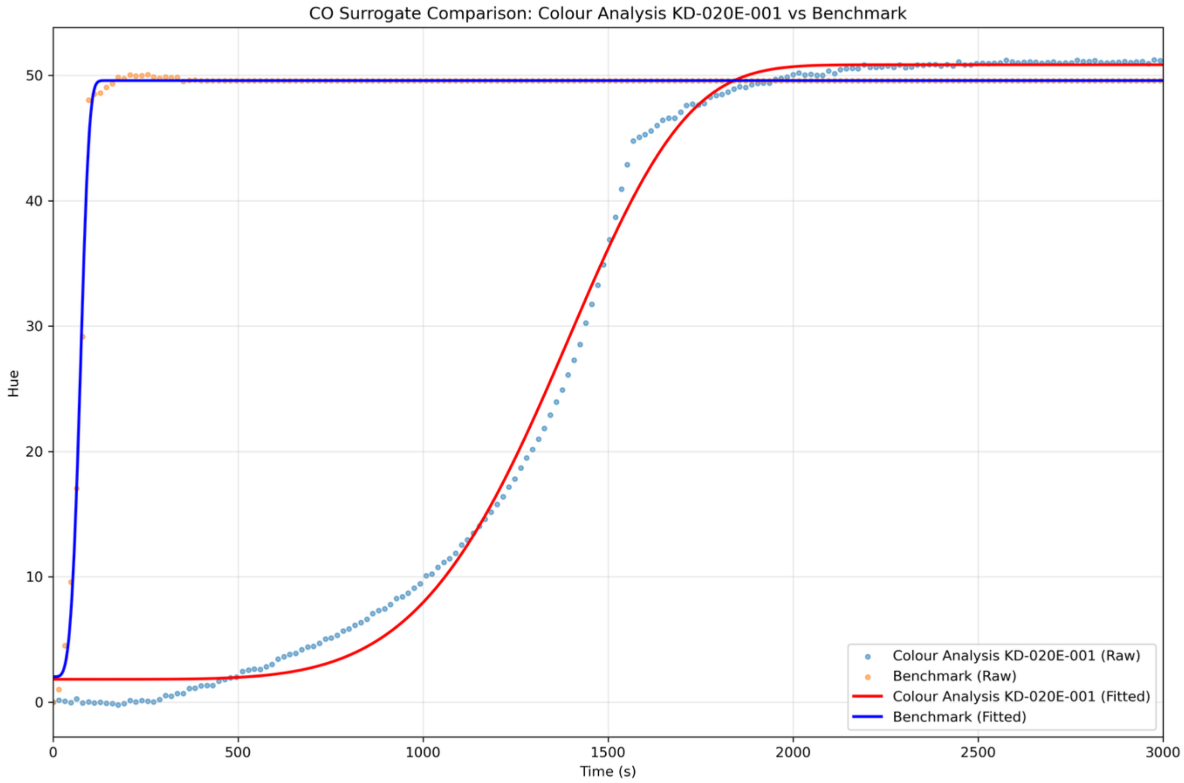

Figure S88: Weibull curves used to calculate surrogate score for KD-020E-001, SilaCO surrogate using modified KF/18-C-6 trigger system.

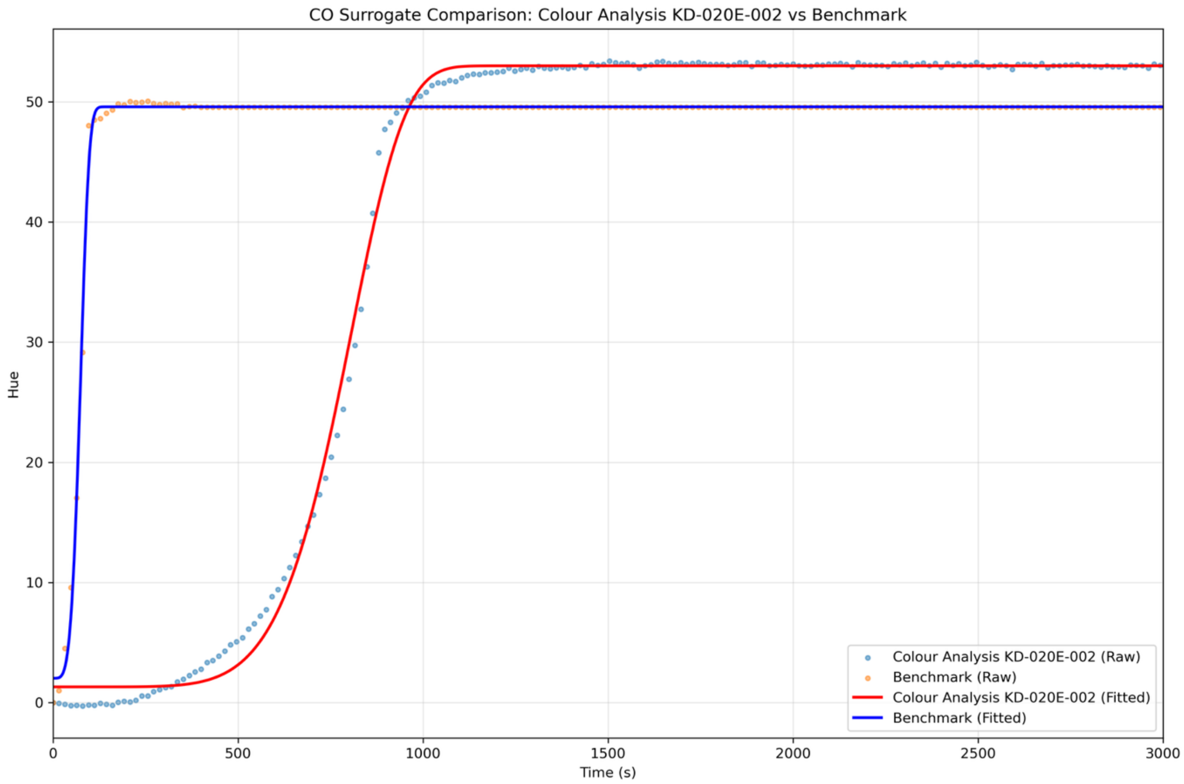

Figure S89: Weibull curves used to calculate surrogate score for KD-020E-002, SilaCO surrogate using modified KF/18-C-6 trigger system.

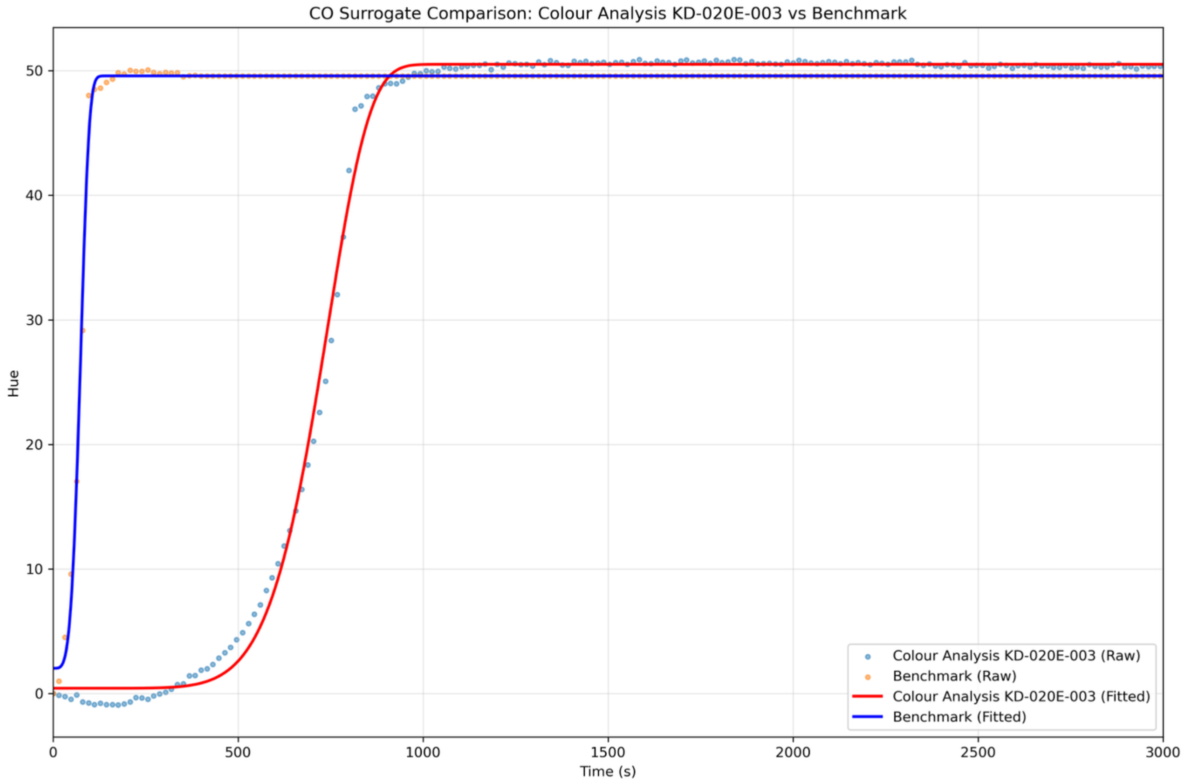

Figure S90: Weibull curves used to calculate surrogate score for KD-020E-003, SilaCO surrogate using modified KF/18-C-6 trigger system.

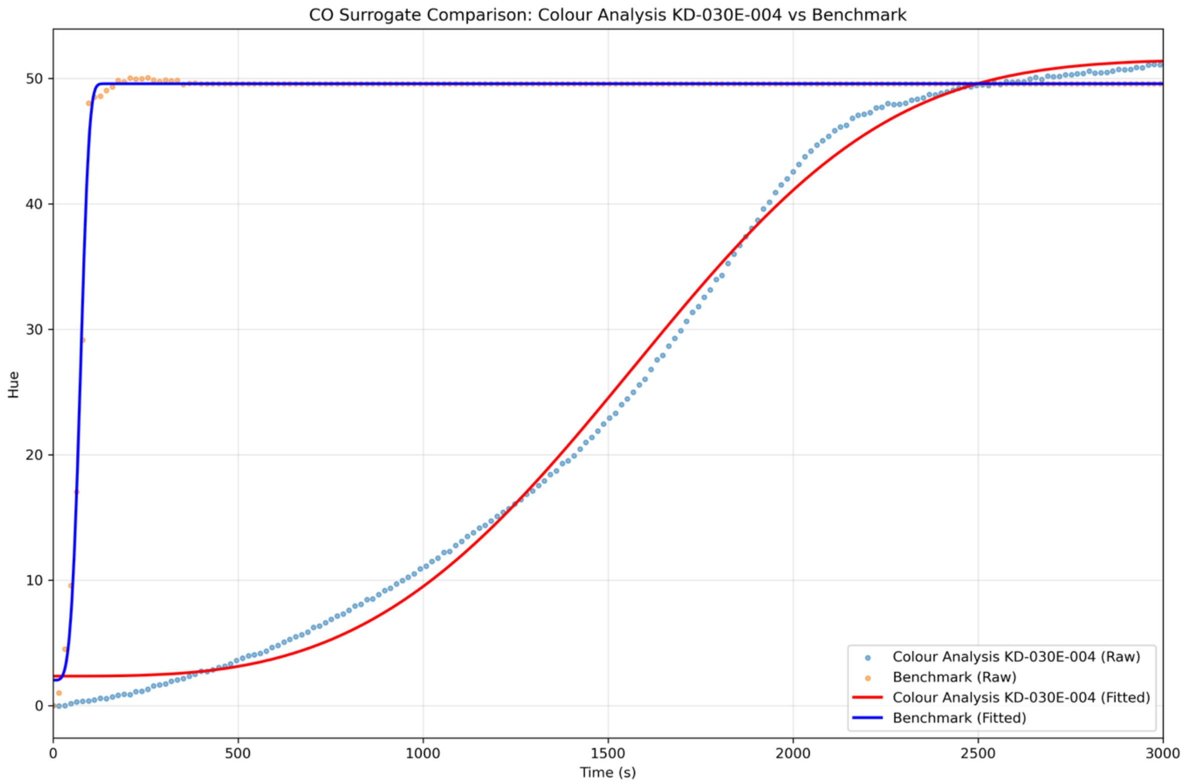

Figure S91: Weibull curves used to calculate surrogate score for KD-030E-004, COgen surrogate with $Pd_2(dba)_3$.

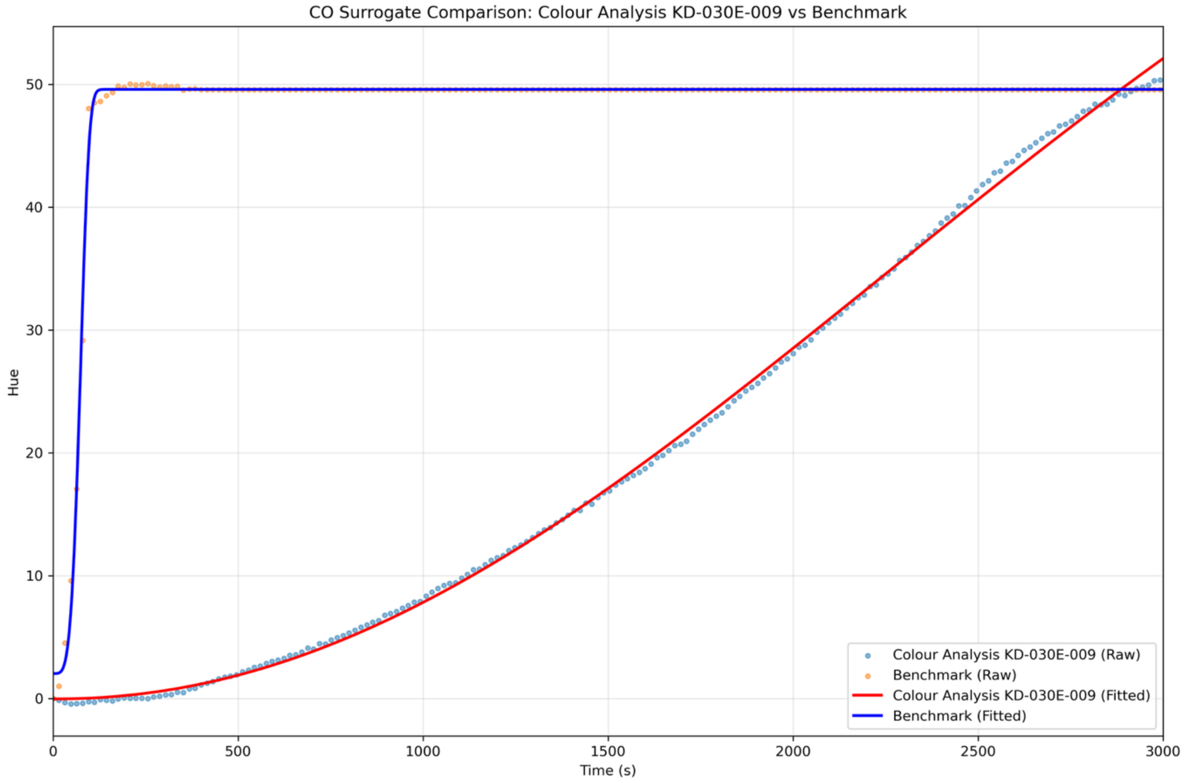

Figure S92: Weibull curves used to calculate surrogate score for KD-030E-009, COgen surrogate with $Pd_2(dba)_3$.

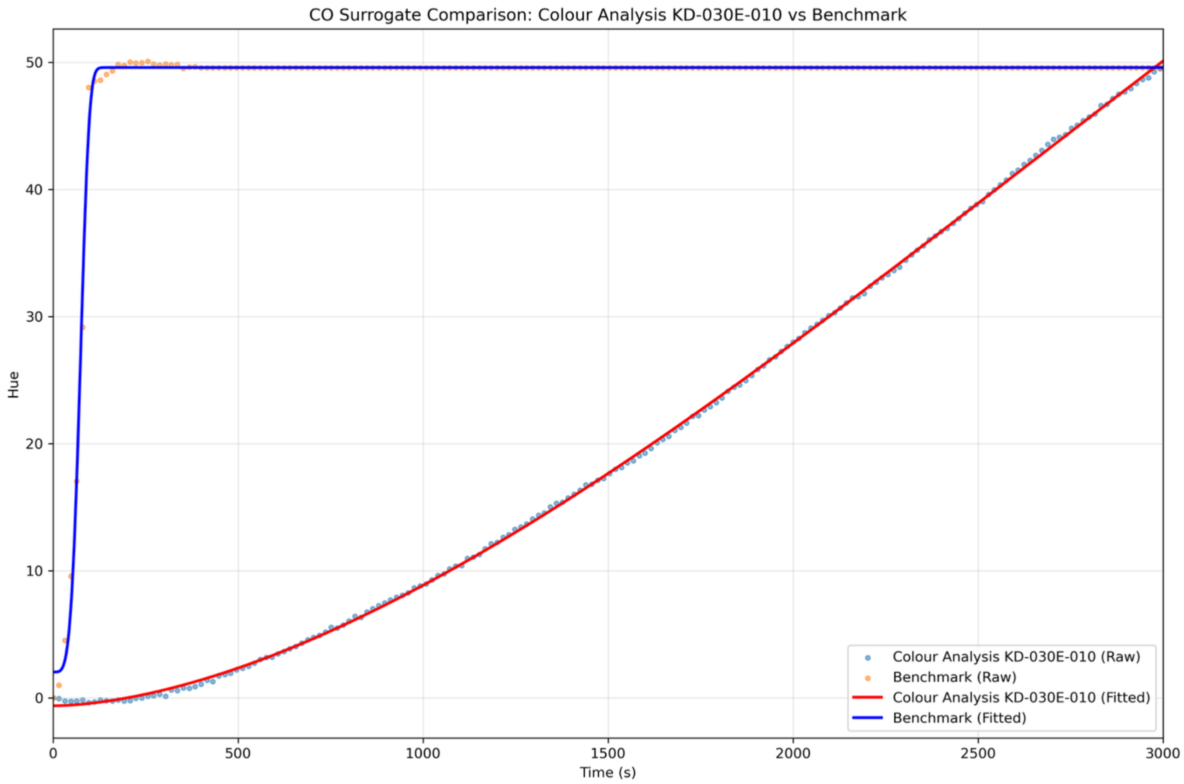

Figure S93: Weibull curves used to calculate surrogate score for KD-030E-004, COgen surrogate with $Pd_2(dba)_3$.

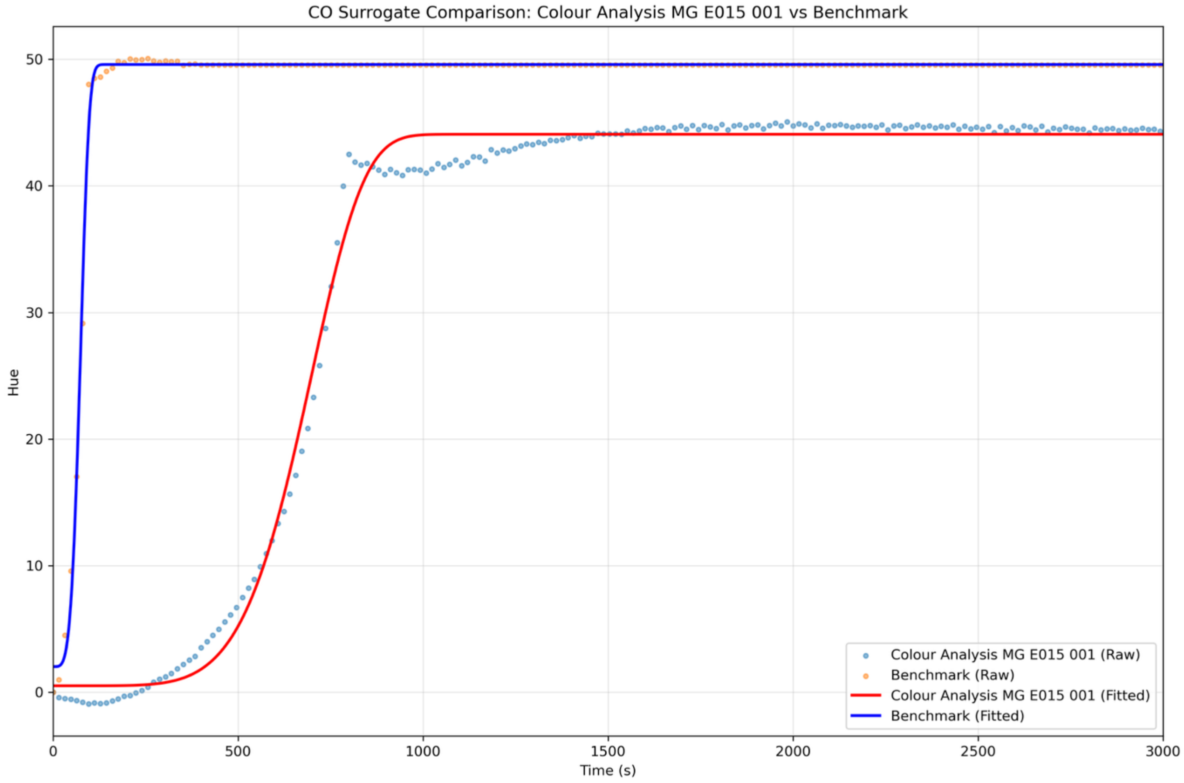

Figure S94: Weibull curves used to calculate surrogate score for MG E015 001, 2,4,6 trichlorophenyl formate surrogate in toluene.

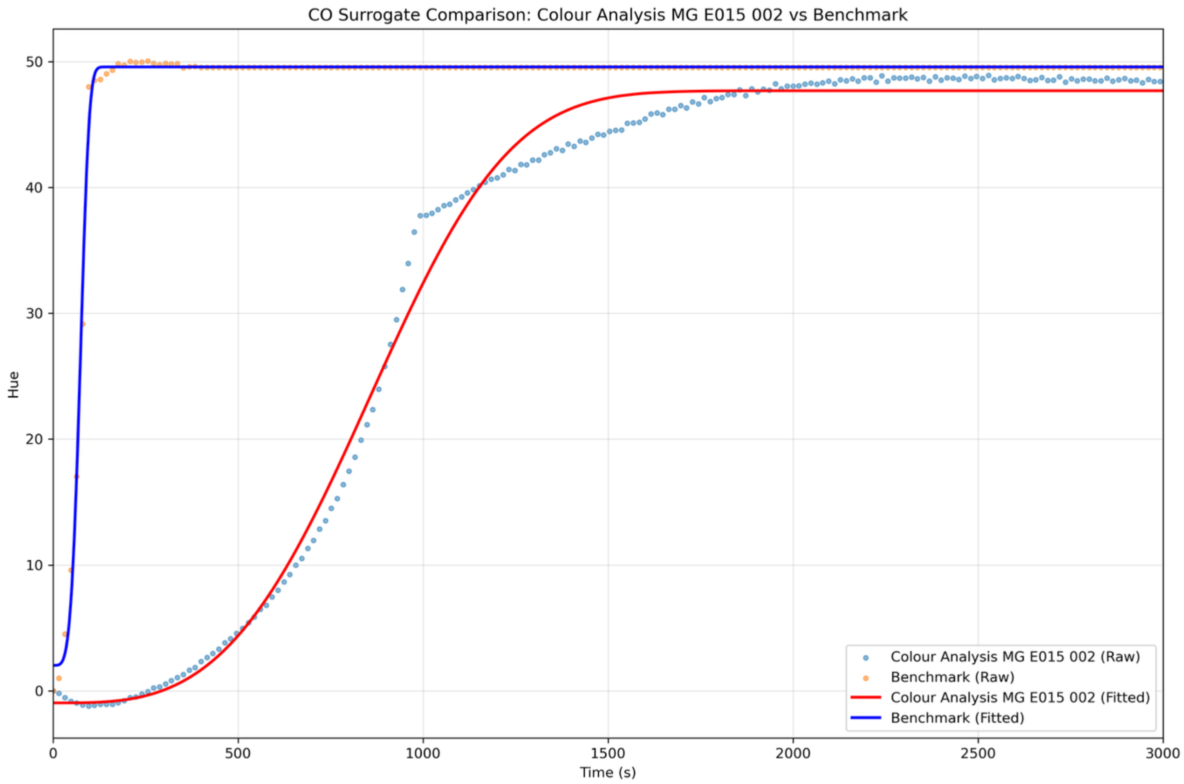

Figure S95: Weibull curves used to calculate surrogate score for MG E015 002, 2,4,6 trichlorophenyl formate surrogate in toluene.

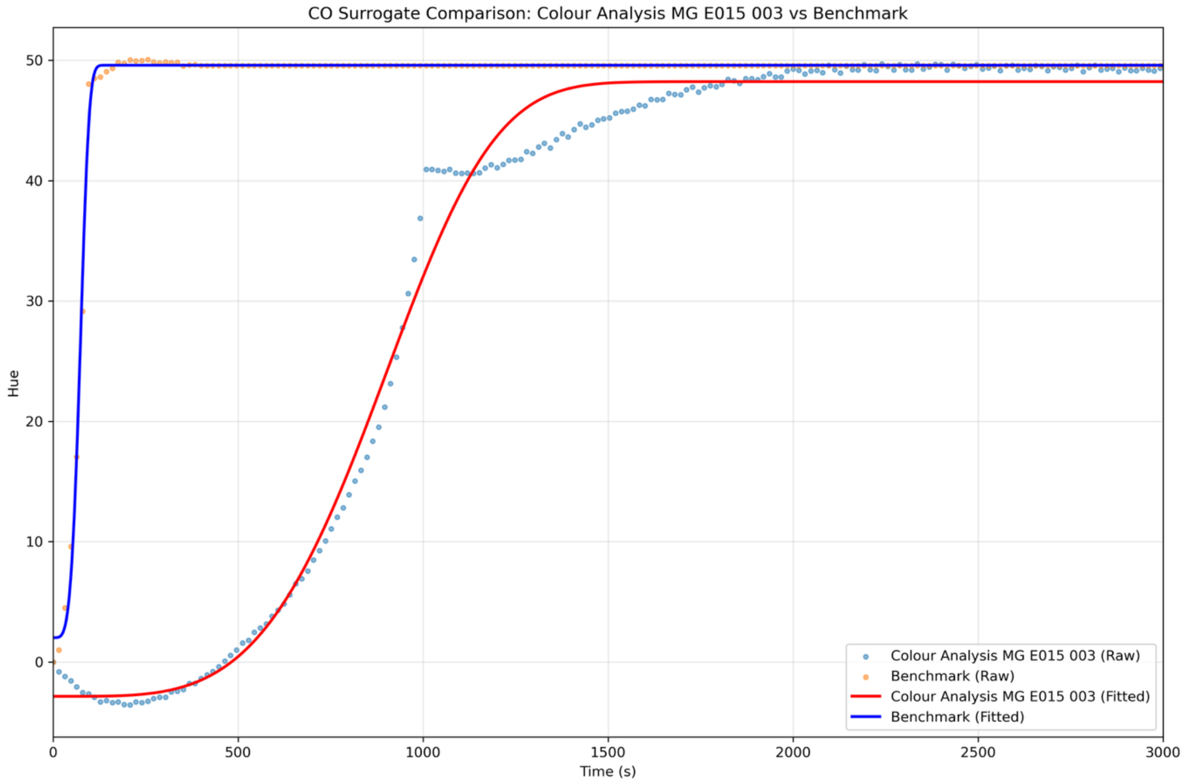

Figure S96: Weibull curves used to calculate surrogate score for MG E015 003, 2,4,6 trichlorophenyl formate surrogate in toluene.

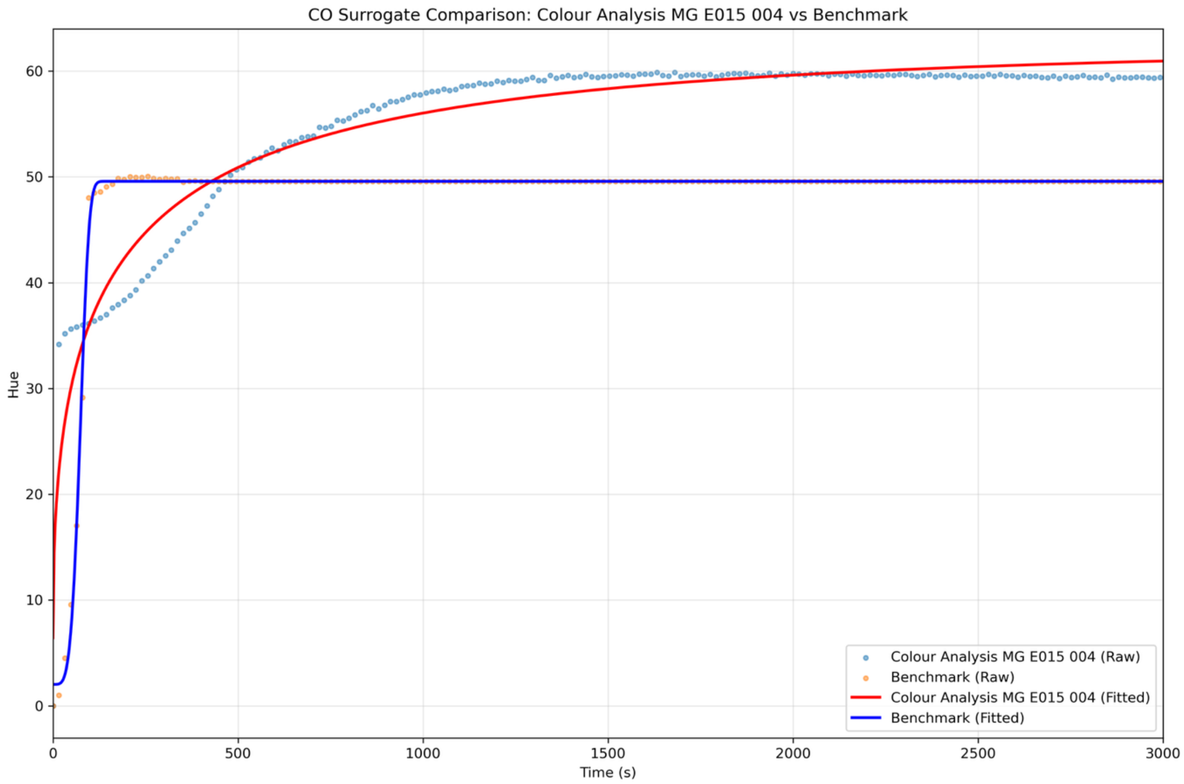

Figure S97: Weibull curves used to calculate surrogate score for MG E015 004, 2,4,6 trichlorophenyl formate surrogate in DMF.

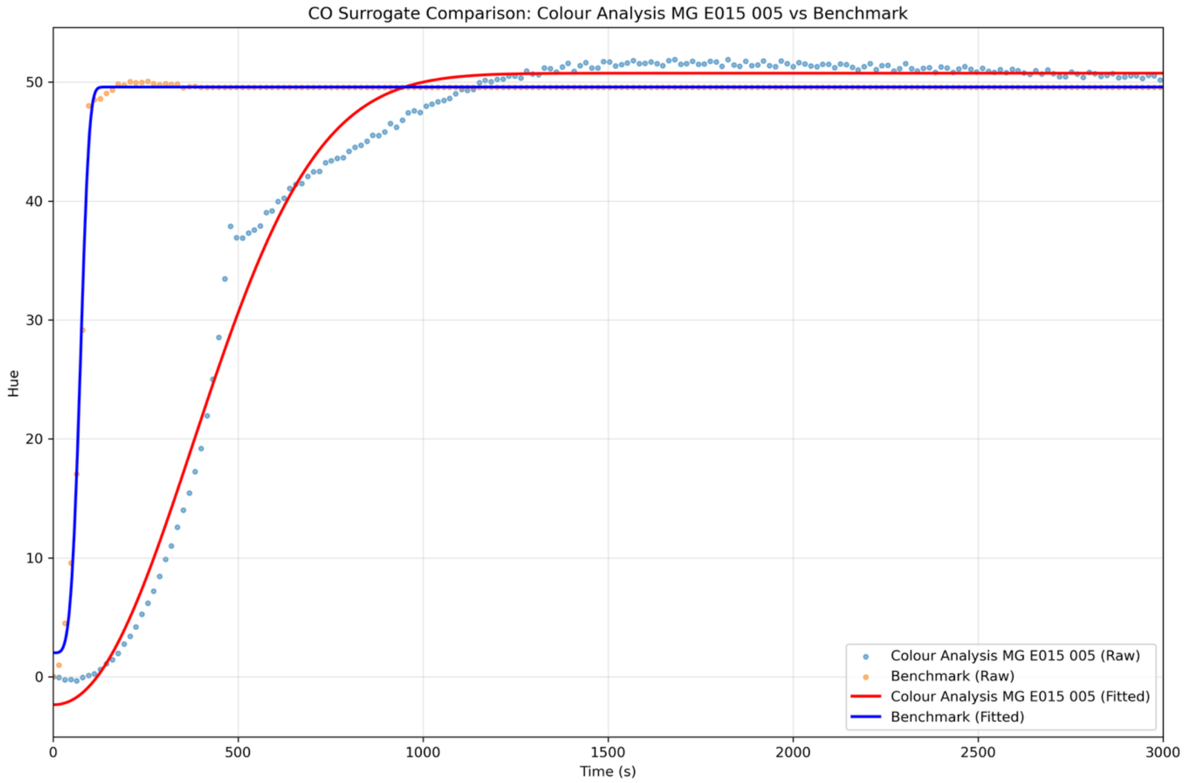

Figure S98: Weibull curves used to calculate surrogate score for MG E015 005, 2,4,6 trichlorophenyl formate surrogate in DMF.

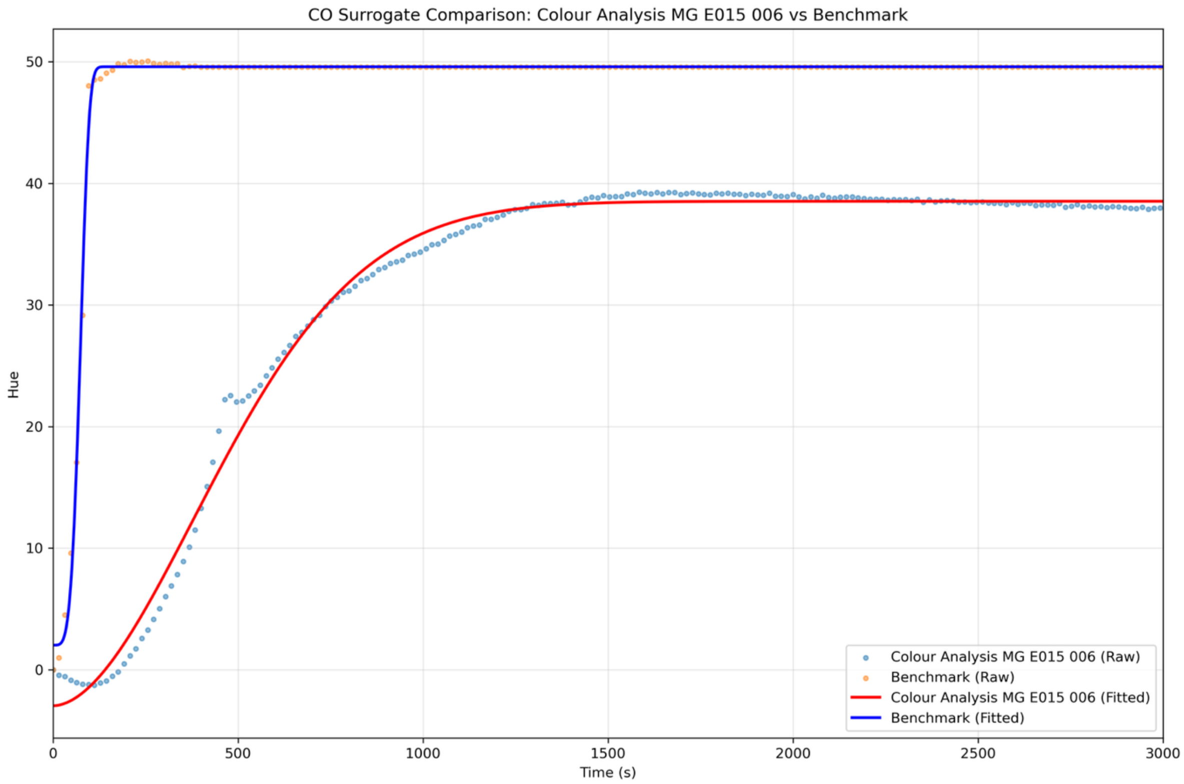

Figure S99: Weibull curves used to calculate surrogate score for MG E015 006, 2,4,6 trichlorophenyl formate surrogate in DMF.

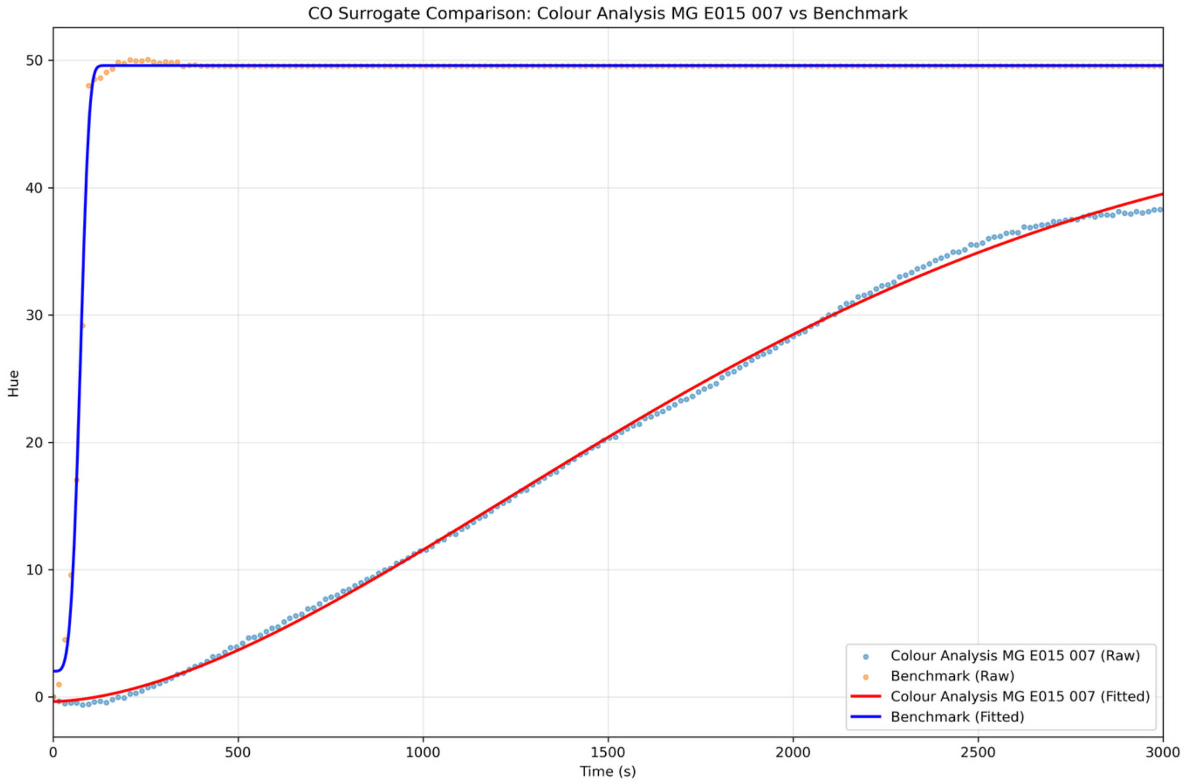

Figure S100: Weibull curves used to calculate surrogate score for MG E015 007, 2,4,6 trichlorophenyl formate surrogate with DBU trigger.

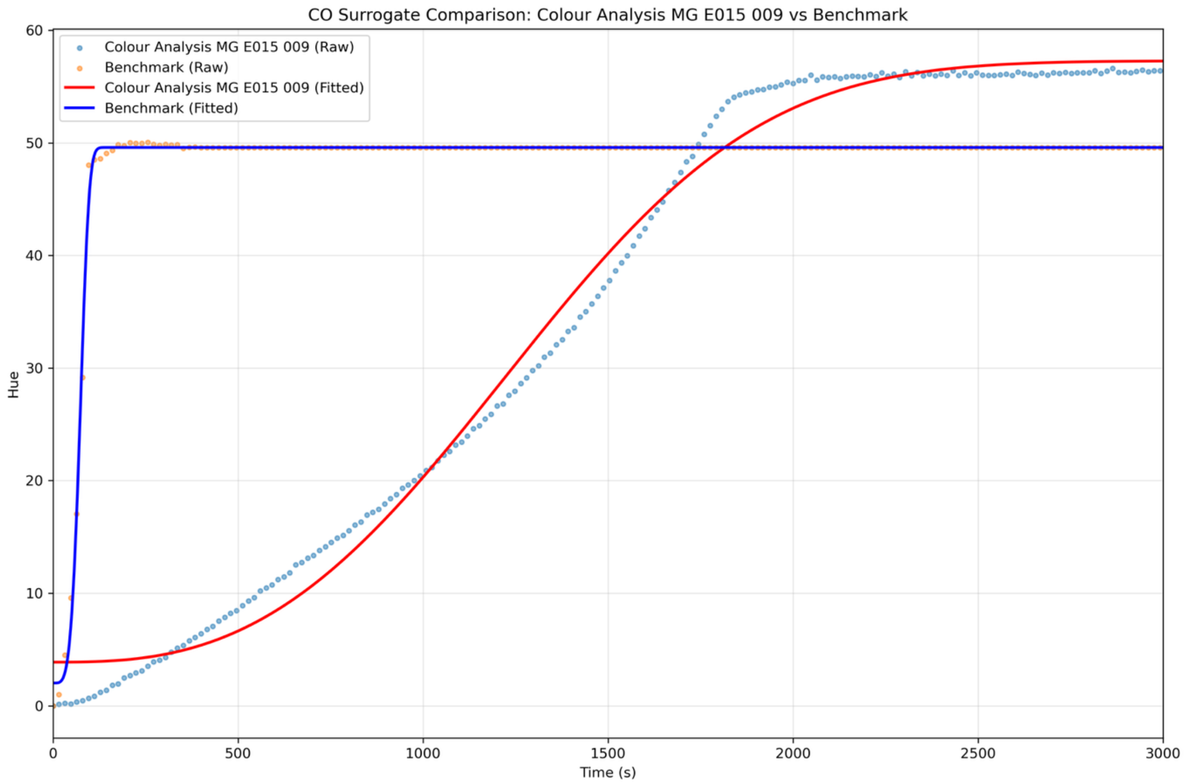

Figure S101: Weibull curves used to calculate surrogate score for MG E015 009, 2,4,6 trichlorophenyl formate surrogate with DBU trigger.

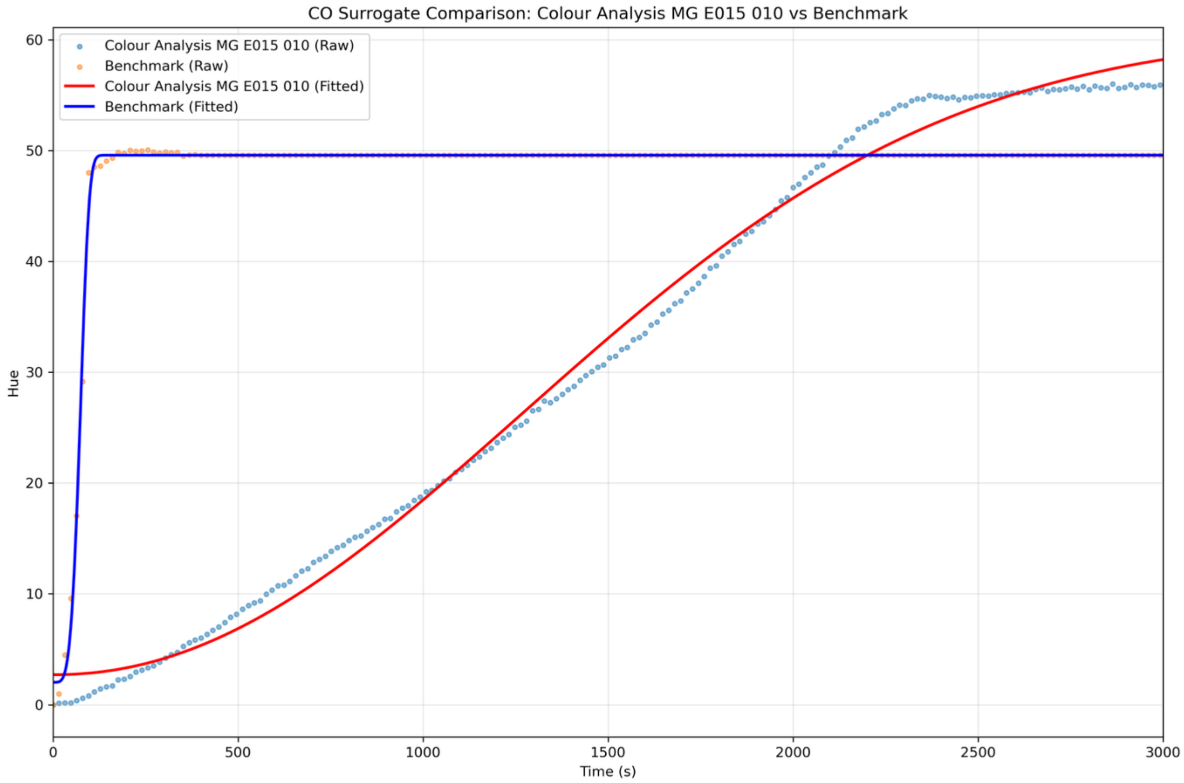

Figure S102: Weibull curves used to calculate surrogate score for MG E015 010, 2,4,6 trichlorophenyl formate surrogate with DBU trigger.

6. NMR

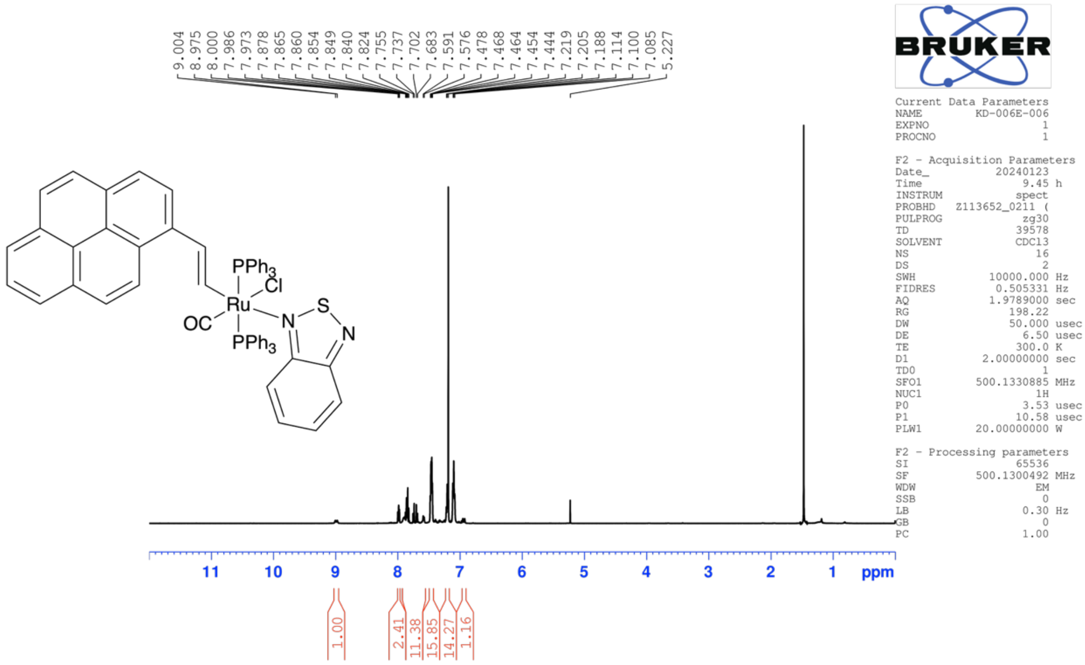

*Figure S103: ¹H NMR of complex **1**.*

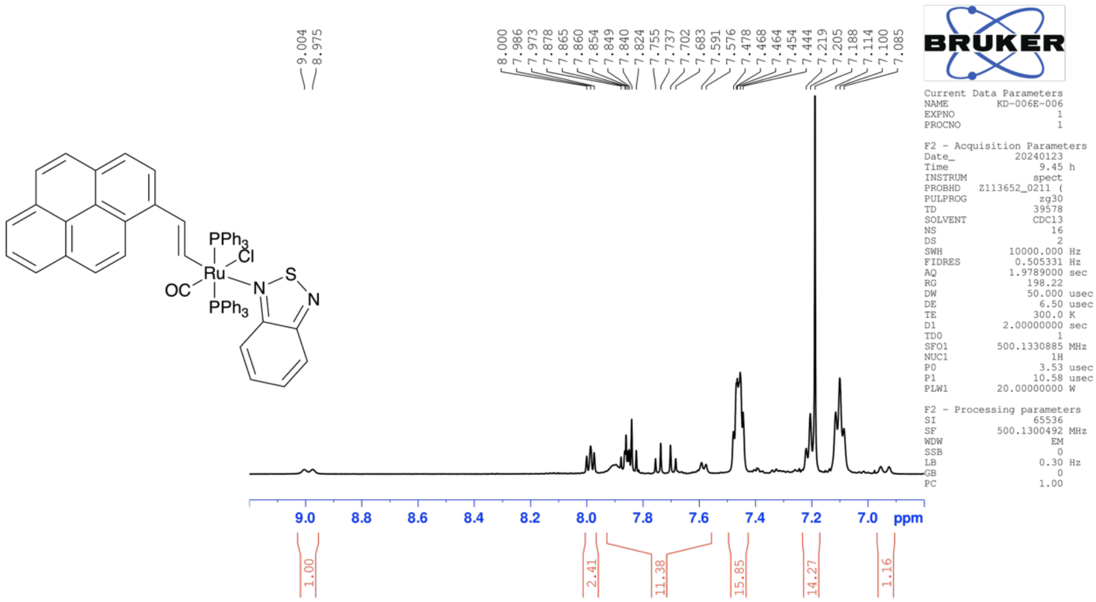

*Figure S104 : ¹H NMR of complex **1**. With focus on the aromatic region of the spectrum.*

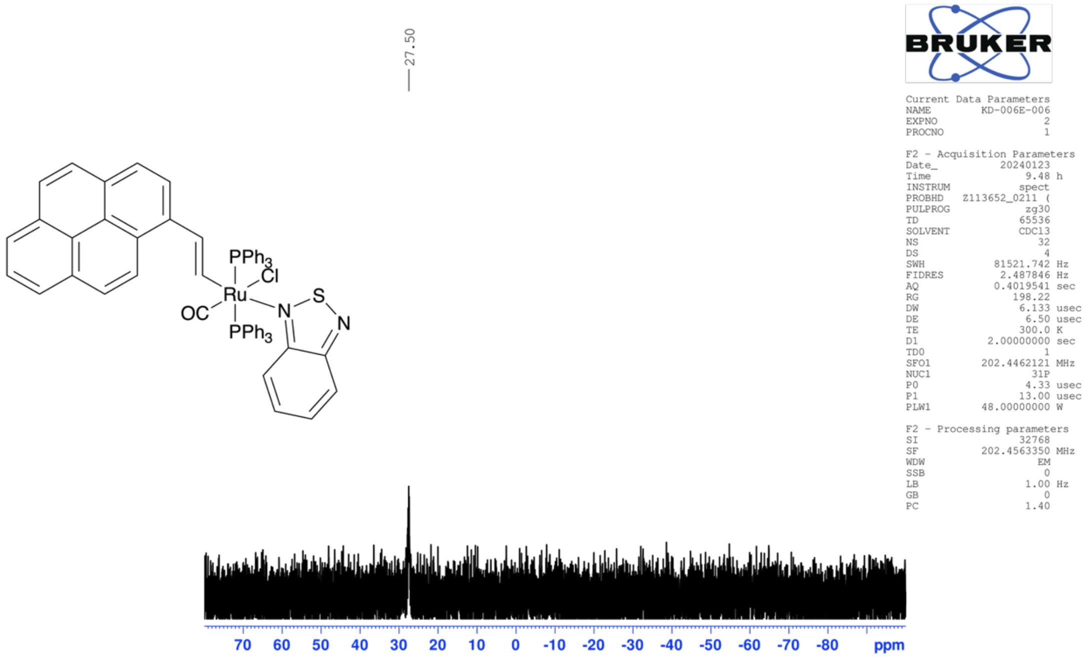

*Figure S105: ^{31}P NMR of complex **1**.*

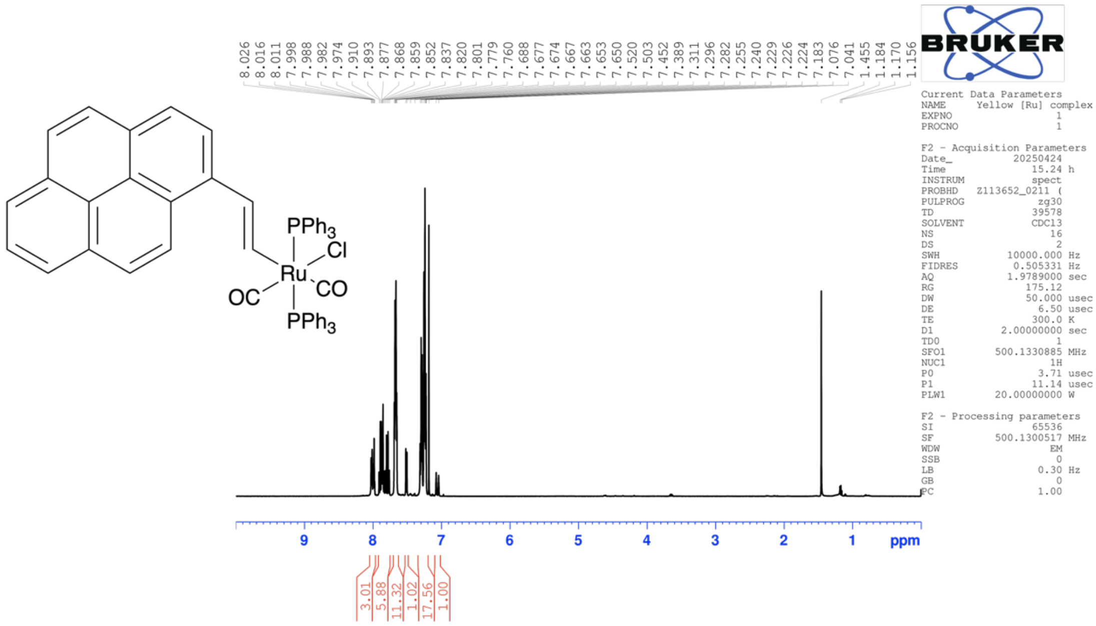

*Figure S106: ^{1}H NMR of complex **2**.*

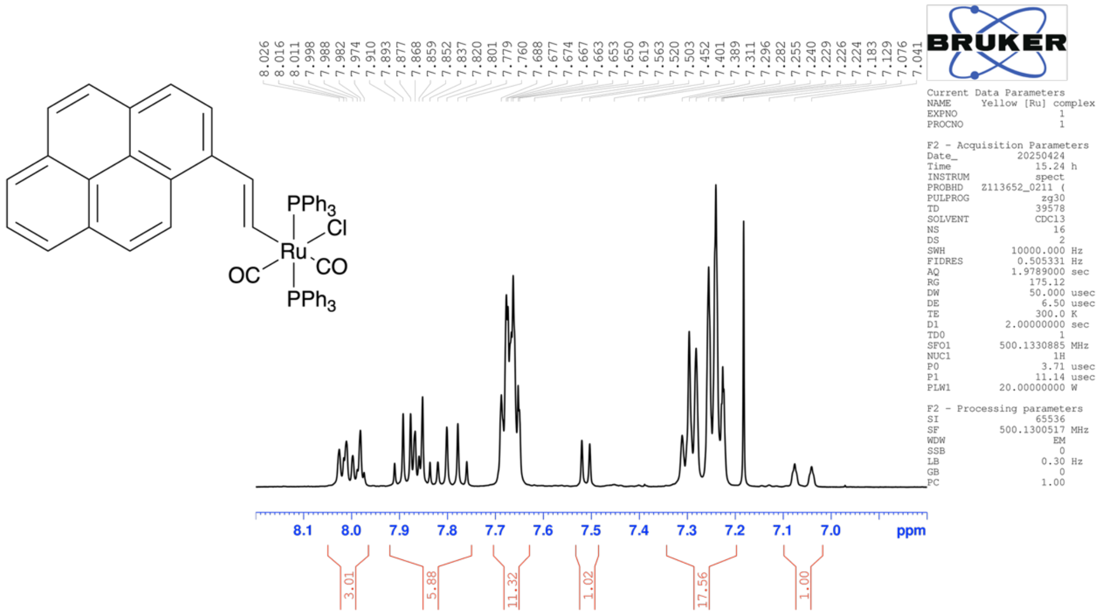

Figure S107: ^{1}H NMR of complex **2**.With focus on the aromatic region of the spectrum.

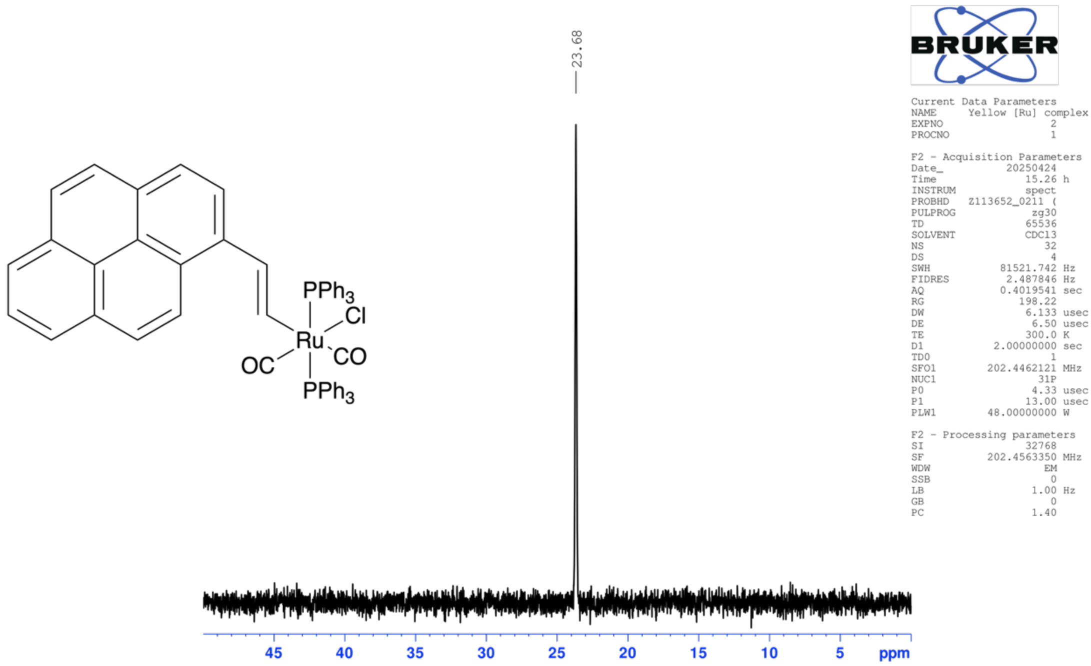

Figure S108: ^{31}P NMR of complex **2**.

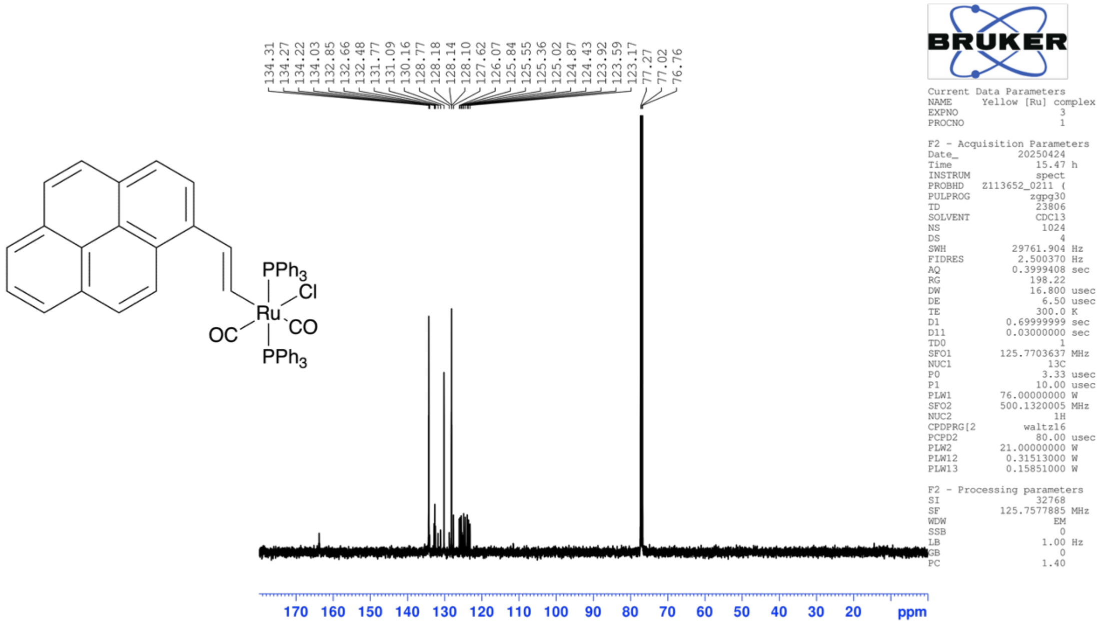

*Figure S109: ^{13}C NMR of complex **2**.*

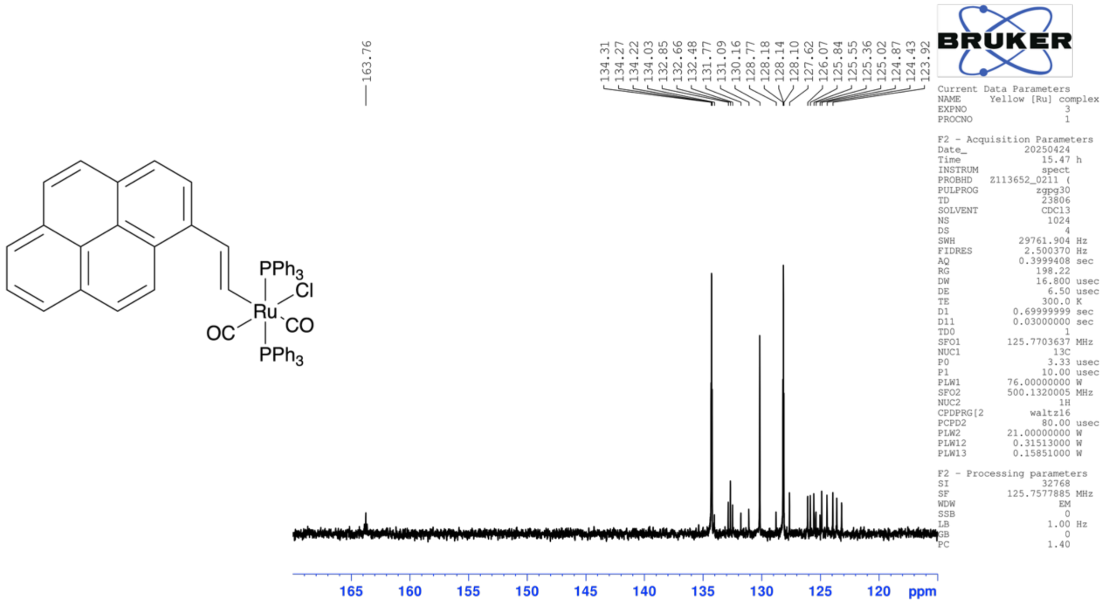

*Figure S110: ^{13}C NMR of complex **2**. Focus on the aromatic region of the spectrum.*

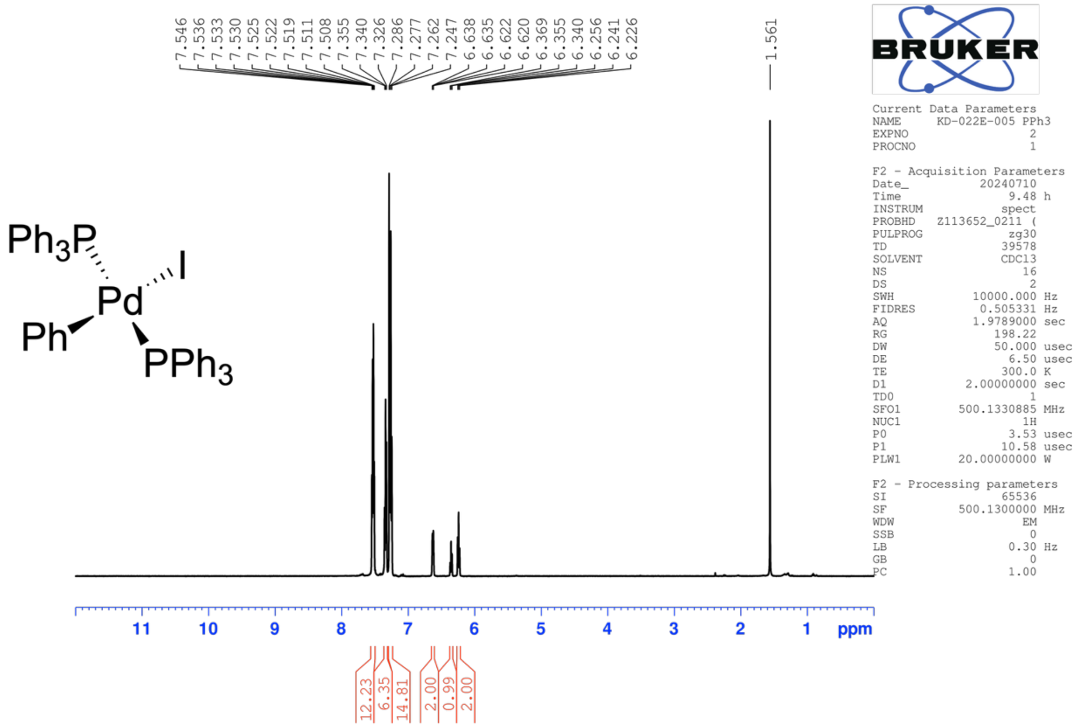

Figure S111: ^{1}H NMR of Ph(I)Pd(PPh$_3$)$_2$.

.

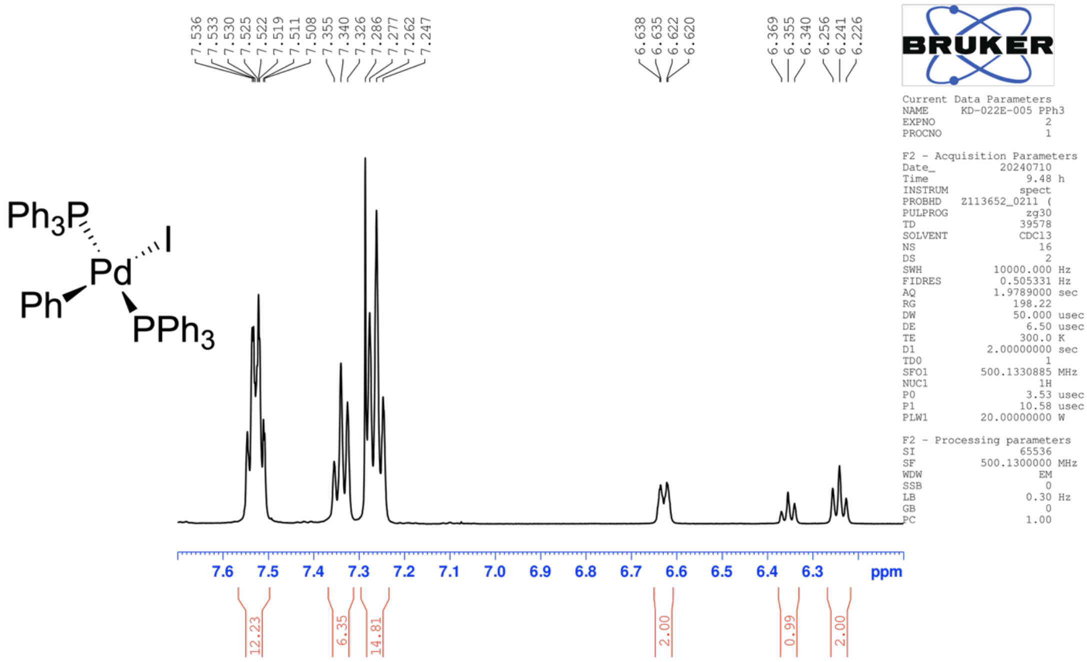

Figure S112: ^{1}H NMR of Ph(I)Pd(PPh$_3$)$_2$. Focus on the aromatic region of the spectrum.

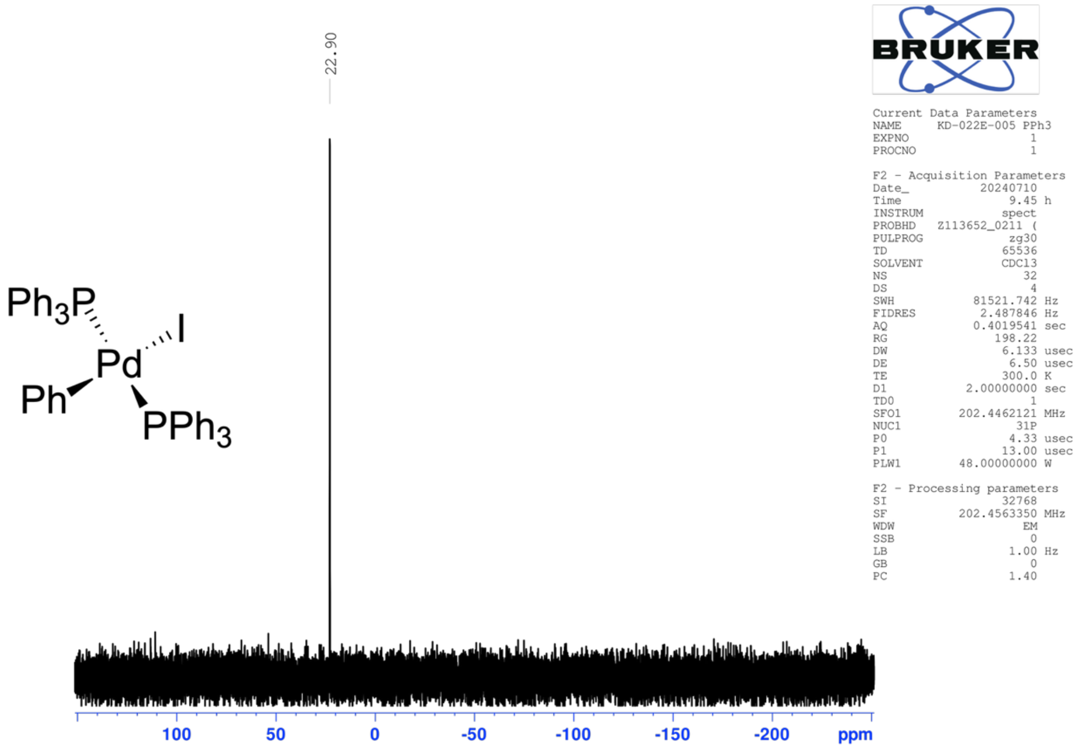

Figure S113: ^{31}P NMR of Ph(I)Pd(PPh$_3$)$_2$.

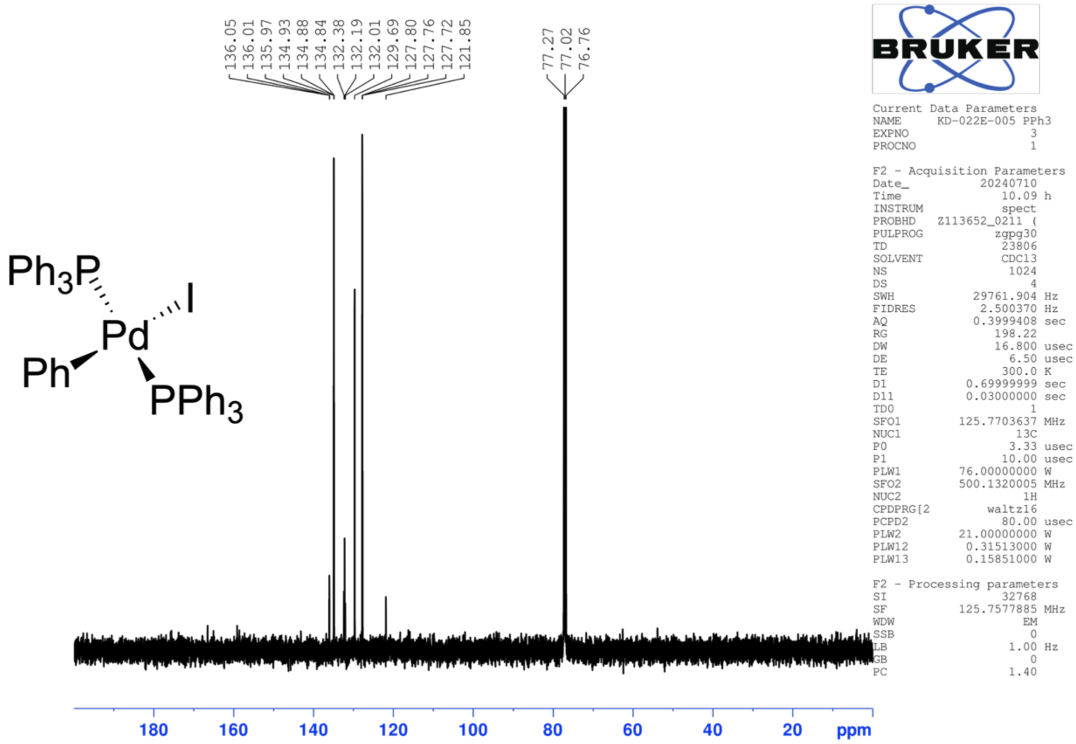

Figure S114: ^{13}C NMR of Ph(I)Pd(PPh$_3$)$_2$.

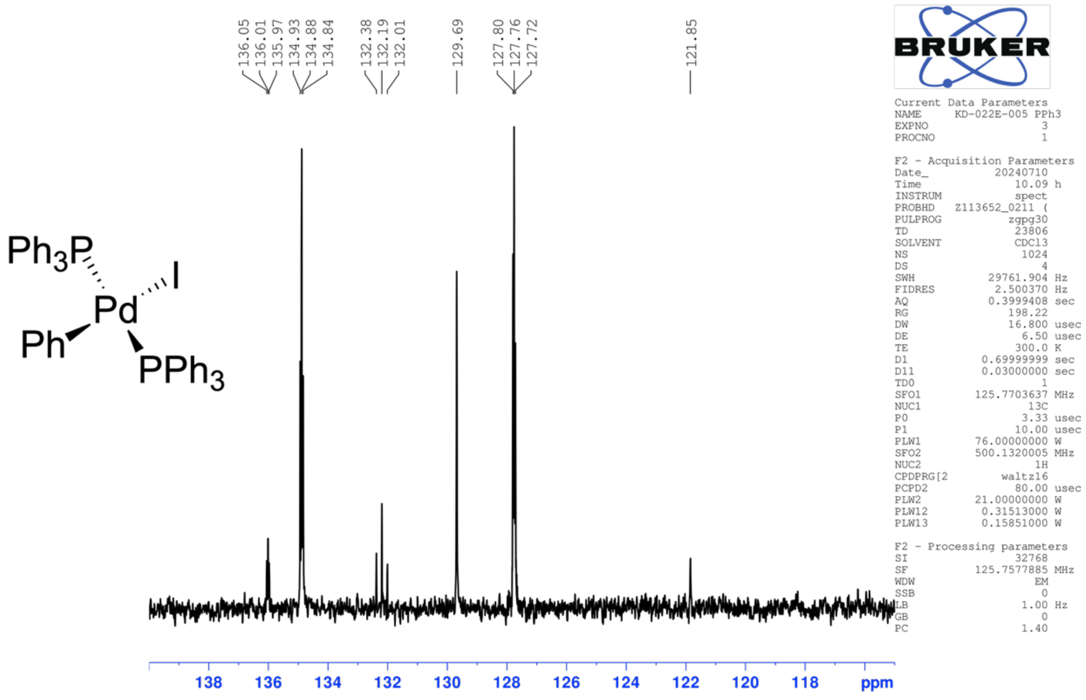

Figure S115: ^{13}C NMR of $Ph(I)Pd(PPh_3)_2$. Focus on the aromatic region of the spectrum.

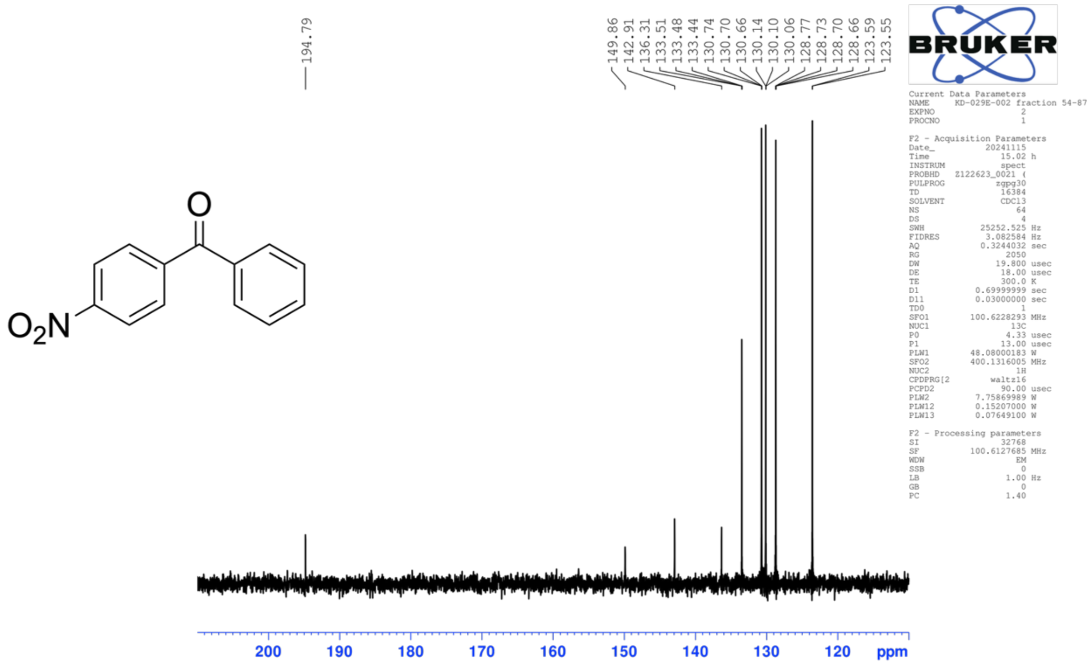

Figure S116: ^{13}C NMR of (4-Nitrophenyl)(phenyl)methanone.

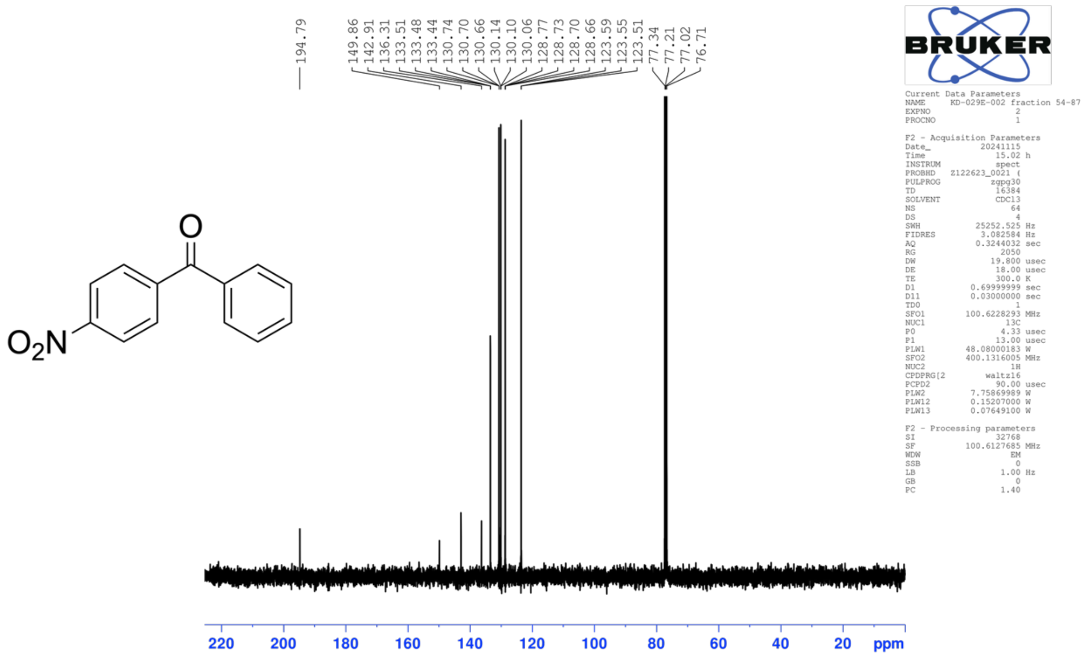

Figure S117: ^{13}C NMR of (4-Nitrophenyl)(phenyl)methanone. Focus on the aromatic region of the spectrum.

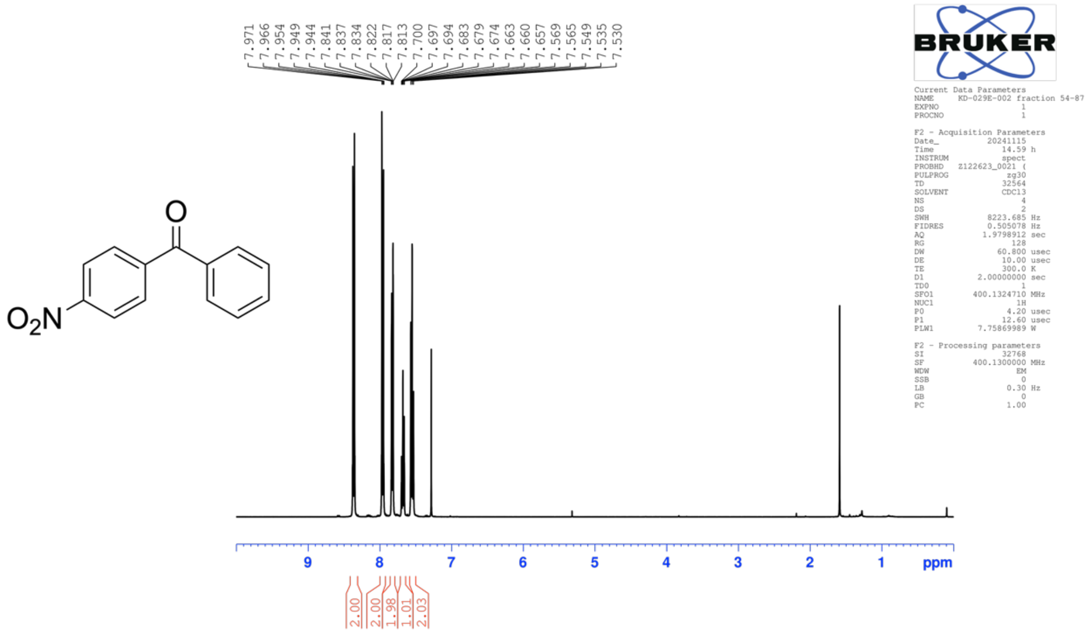

Figure S118: ^{1}H NMR of (4-Nitrophenyl)(phenyl)methanone.

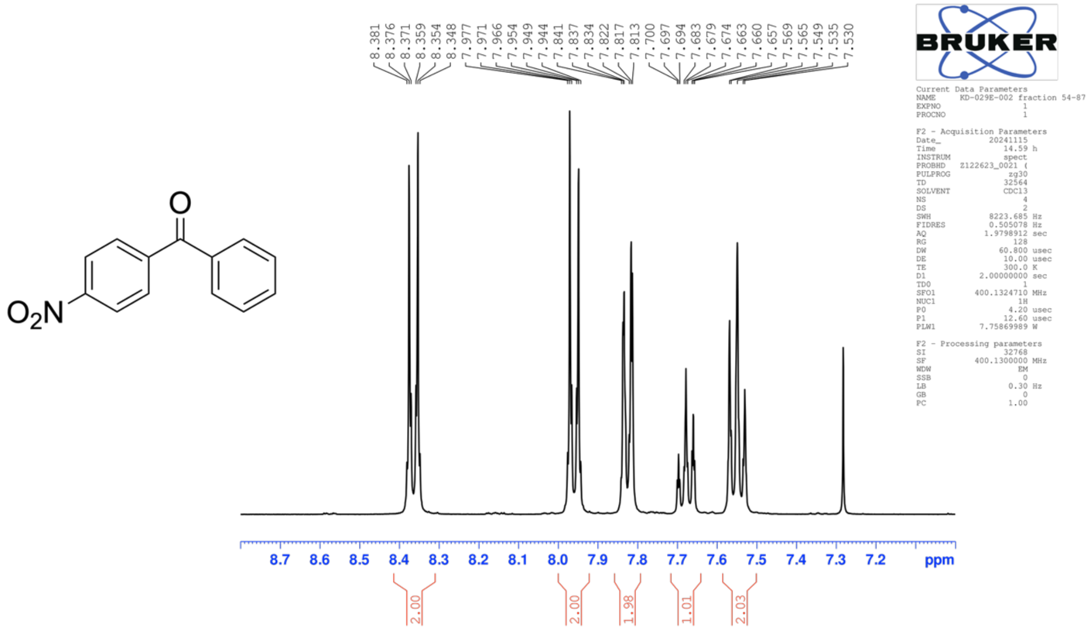

Figure S119: ^{1}H NMR of (4-Nitrophenyl)(phenyl)methanone. Focus on the aromatic region of the spectrum.

7. FTIR

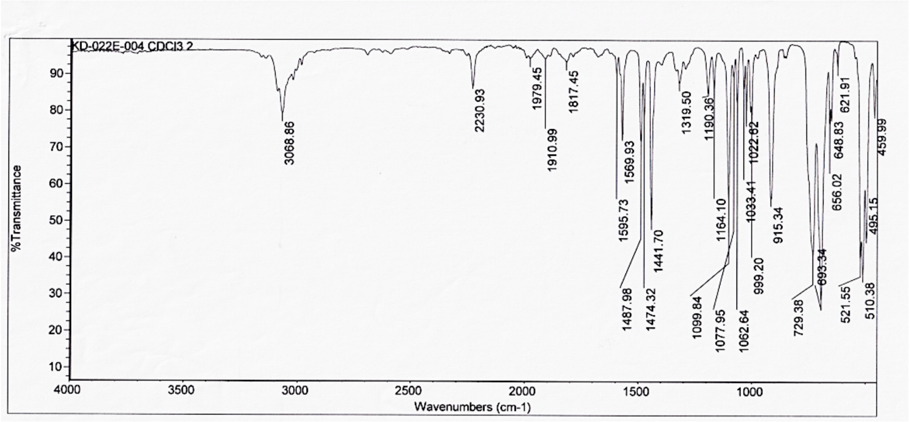

Figure S120: FTIR of $Ph(I)Pd(PPh_3)_2$ in $CDCl_3$

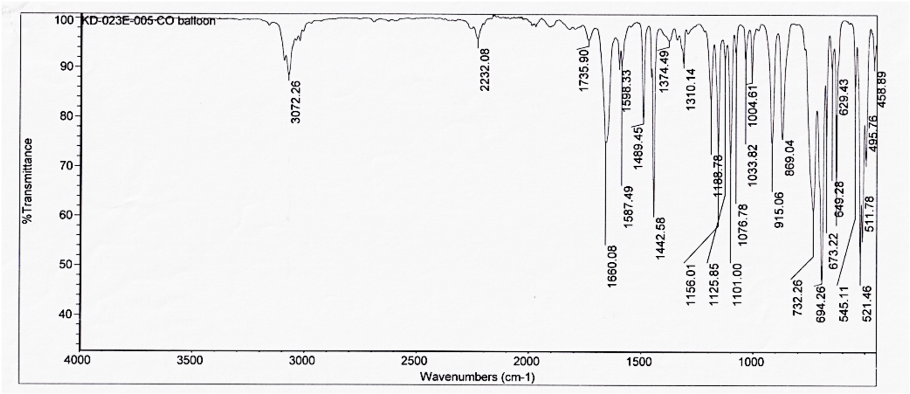

Figure S121: FTIR of $PhCO(I)Pd(PPh_3)_2$ in $CDCl_3$, CO from a balloon.

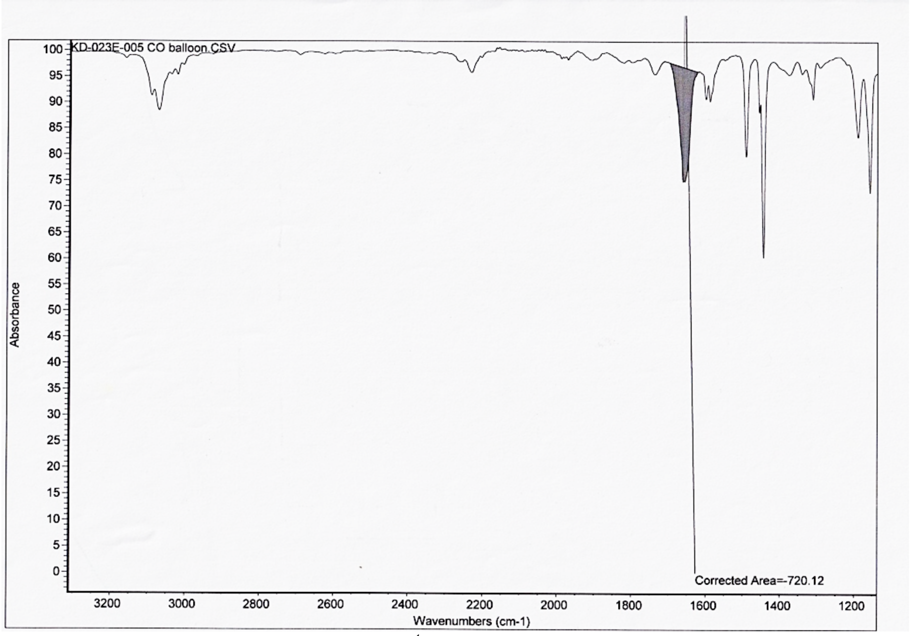

Figure S122: Corrected area of C=O peak (1660 cm^{-1}) obtained from FTIR analysis of $PhCO(I)Pd(PPh_3)_2$ production using a CO balloon.

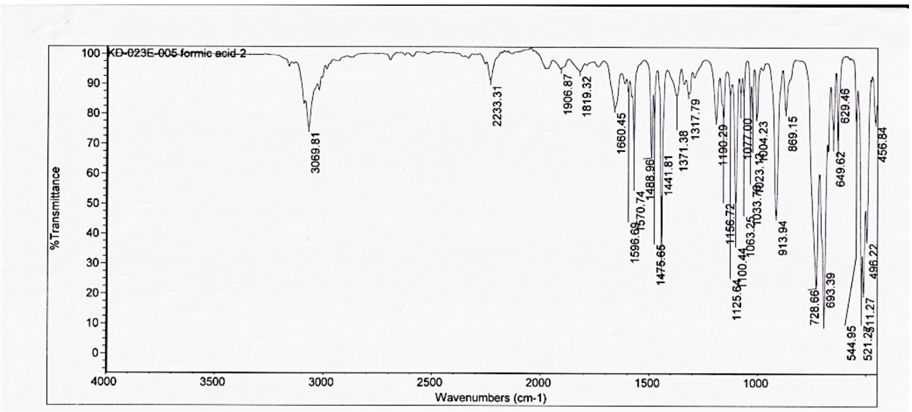

Figure S123: FTIR of PhCO(I)Pd(PPh$_3$)$_2$ in CDCl$_3$, CO from formic acid.

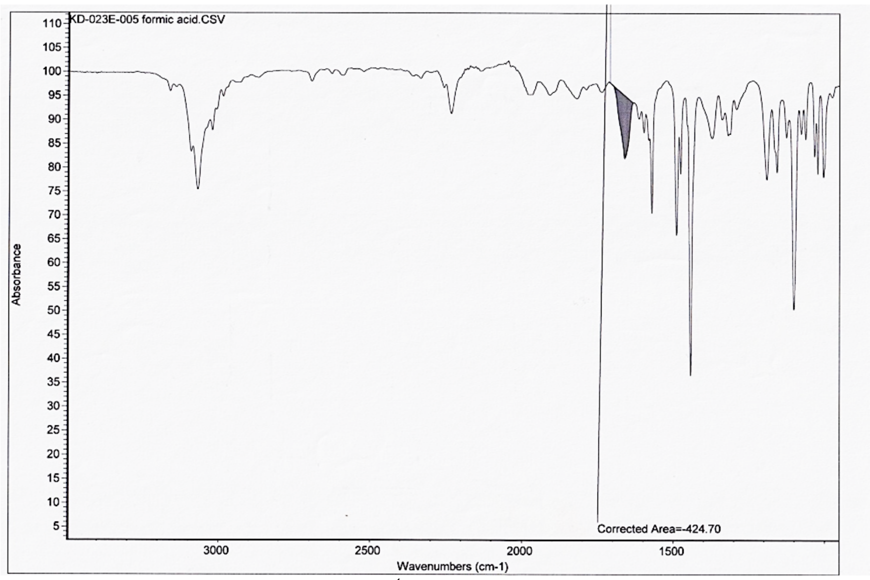

Figure S124: Corrected area of C=O peak (1660 cm^{-1}) obtained from FTIR analysis of PhCO(I)Pd(PPh$_3$)$_2$ production using formic acid.

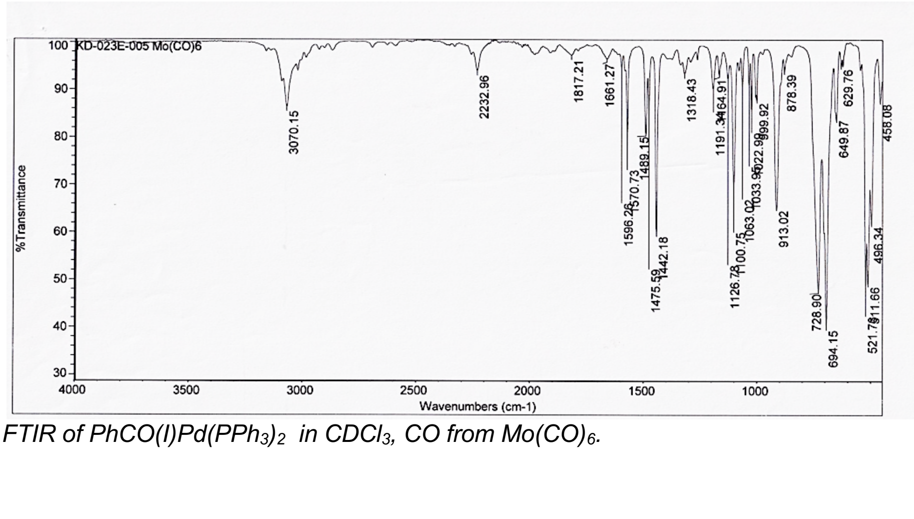

Figure S125: FTIR of PhCO(I)Pd(PPh$_3$)$_2$ in CDCl$_3$, CO from Mo(CO)$_6$.

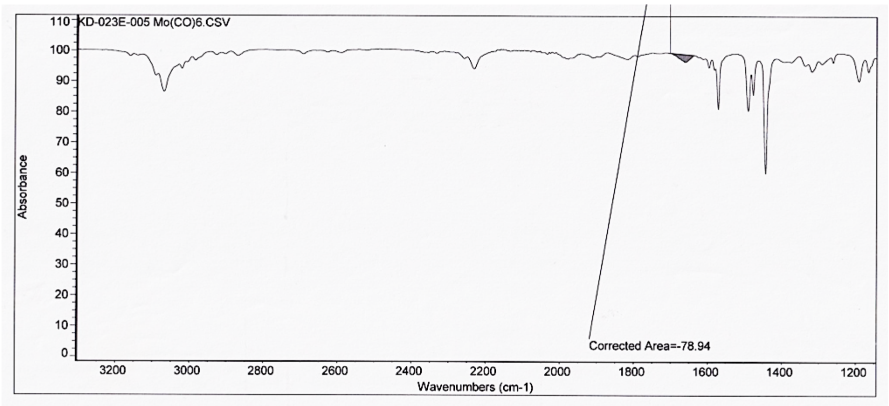

Figure S126: Corrected area of C=O peak (1660 cm^{-1}) obtained from FTIR analysis of $PhCO(I)Pd(PPh_3)_2$ production using $Mo(CO)_6$.

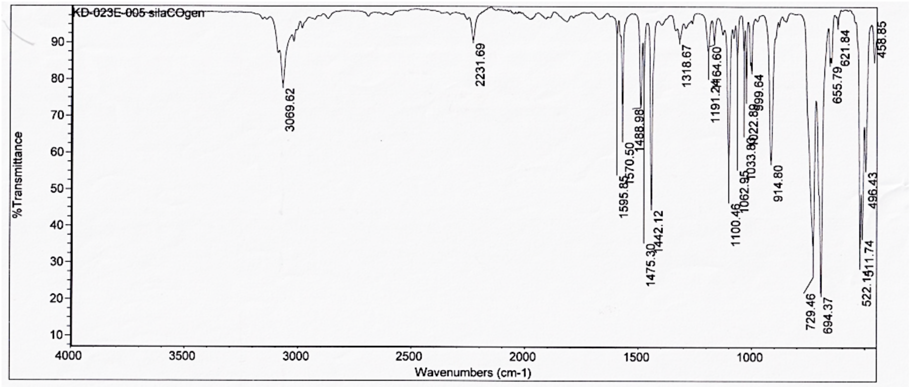

Figure S127: FTIR of $PhCO(I)Pd(PPh_3)_2$ in $CDCl_3$, CO from SilaCO.

8. UV-Vis

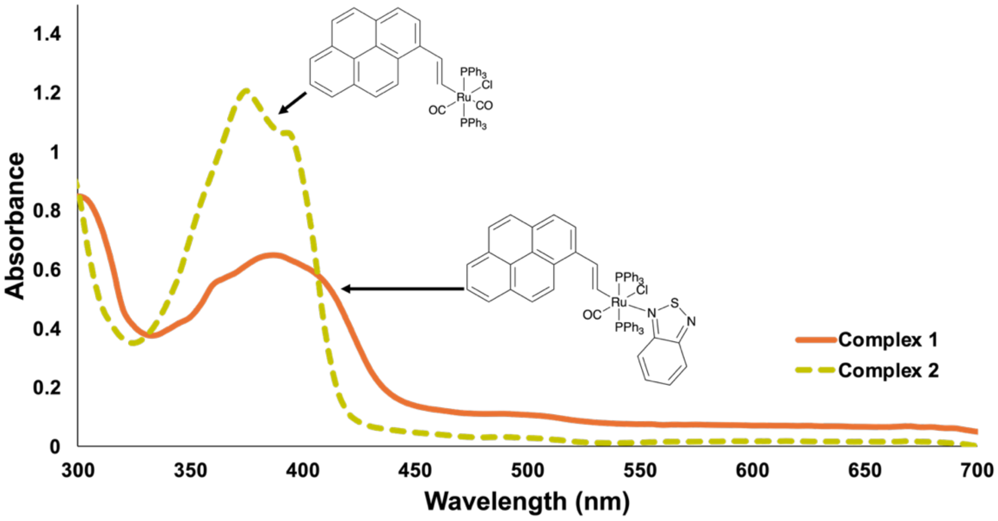

Figure S128: UV-Vis data of complex 1 (solid orange line) and complex 2 (dash yellow line). Samples were 0.027 mM in DCE.

9. HPLC

Acquisition method shown in Table S14. Each sample was prepared by taking 25μL of the crude reaction mixture from chamber **B,** 250 μL of 1mg/mL acetophenone in acetonitrile (internal standard) and adding it to 725 μL neat acetonitrile. The sample was analyzed using an Agilent 1220 Infinity II fitted with a Poroshell, HPH-C18 4 μm, 4.6 μm x 150 mm. UV detection was set at both 214 and 273 nm. Full HPLC methods are available in the supporting information zipped folder.

Table S14: HPLC acquisition. ***A:*** *acetonitrile with 0.1% TFA,* ***B:*** *water with 0.1% TFA. Flow rate was set to 1.8 mL/min, pressure 600 bar, injection volume was 5μL and column temperature was 30℃.*

**Time (min)**	**A (%)**	**B (%)**
Start Conditions	50	50
0.50	20	80
3.00	50	50
5.00	50	50

Table S15: HPLC results from analysis of crude reaction mixture from Chamber B of each repeat.

Experiment	Surrogate	HPLC yield (%)	Average conversion	Standard Dev	Product Detected (mmol)	Remaining 1-iodo-4-nitrobenzene (mg)	1-iodo-4-nitrobenzene consumed (mmol)
KD-029E-015	Formic acid	38	36	10	0.19	72	0.21
KD-029E-019	Formic acid	26			0.13	73	0.21
KD-029E-023	Formic acid	46			0.23	56	0.28
KD-029E-010	$Mo(CO)_6$	44	50	7	0.25	43	0.33
KD-029E-014	$Mo(CO)_6$	48			0.24	36	0.36
KD-029E-021	$Mo(CO)_6$	58			0.29	33	0.37
KD-029E-013	SilaCO	63	61	3	0.31	17	0.43
KD-029E-017	SilaCO	58			0.29	28	0.39
KD-029E-018	SilaCO	63			0.31	25	0.40
KD-029E-029	SilaCO + KF/18-C-6	32	32	14	0.16	6	0.47
KD-029E-030	SilaCO + KF/18-C-6	45			0.23	6	0.48
KD-029E-031	SilaCO + KF/18-C-6	18			0.09	9	0.46

Injection Report - By Sample

Kineticolor

**Sample name:**	KD-029E-029		
**Data file:**	2025-07-15 08-45-12+01-00-02.dx	**Operator:**	SYSTEM
**Instrument:**	1220 Infinity II HPLC	**Injection date:**	2025-07-15 08:46:41+01:00
**Inj. volume:**	5.000 µL	**Location:**	24
**Acq. method:**	KD 29E.amx	**Type:**	Sample
**Processing method:**	3D UV Quantitative_DefaultMethod_KD 29E_unknown.pmx		
**Manually modified:**	Manual Integration		

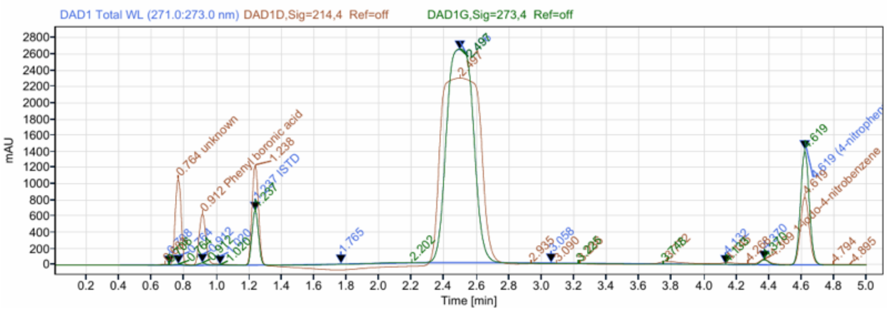

Sample Name	Name	RT (mins)	Area	Compound Amount (mg/mL)
KD-029E-029	unknown	0.764	2677.4827	
KD-029E-029	Phenyl boronic acid	0.912	1776.7988	0.108
KD-029E-029	ISTD	1.237	1861.5496	1.000
KD-029E-029	1-iodo-4-nitrobenzene	4.369	204.5227	0.054
KD-029E-029	(4-nitrophenyl)(phenyl)methone	4.619	5380.3615	0.299

Figure S129: HPLC trace of crude reaction mixture from KD-029E-029. SilaCO with modified KF/18-C-6 trigger system as CO source.

Injection Report - By Sample

**Sample name:**	KD-029E-030-2		
**Data file:**	2025-07-16 09-11-39+01-00-02.dx	**Operator:**	SYSTEM
**Instrument:**	1220 Infinity II HPLC	**Injection date:**	2025-07-16 09:13:11+01:00
**Inj. volume:**	5.000 µL	**Location:**	26
**Acq. method:**	KD 29E.amx	**Type:**	Sample
**Processing method:**	3D UV Quantitative_DefaultMethod_KD 29E_unknown.pmx		
**Manually modified:**	Manual Integration		

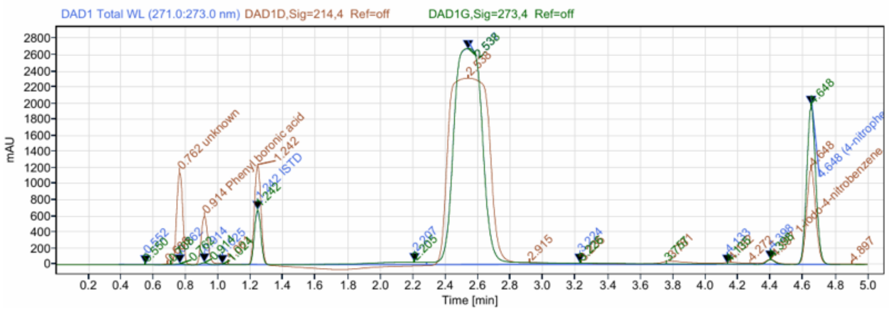

Sample Name	Name	RT (mins)	Area	Compound Amount (mg/mL)
KD-029E-030-2	unknown	0.762	2915.9519	
KD-029E-030-2	Phenyl boronic acid	0.914	1656.9735	0.100
KD-029E-030-2	ISTD	1.242	1866.1991	1.000
KD-029E-030-2	1-iodo-4-nitrobenzene	4.397	191.6184	0.051
KD-029E-030-2	(4-nitrophenyl)(phenyl)methone	4.648	7665.7764	0.428

Figure S130: HPLC trace of crude reaction mixture from KD-029E-030. SilaCO with modified KF/18-C-6 trigger system as CO source.

Injection Report - By Sample

Kineticolor

**Sample name:**			
**Data file:**	2025-07-17 12-28-16+01-00-02.dx	**Operator:**	SYSTEM
**Instrument:**	1220 Infinity II HPLC	**Injection date:**	2025-07-17 12:29:44+01:00
**Inj. volume:**	5.000 µL	**Location:**	28
**Acq. method:**	KD 29E.amx	**Type:**	Sample
**Processing method:**	3D UV Quantitative_DefaultMethod_KD 29E_unknown.pmx		
**Manually modified:**	Manual Integration		

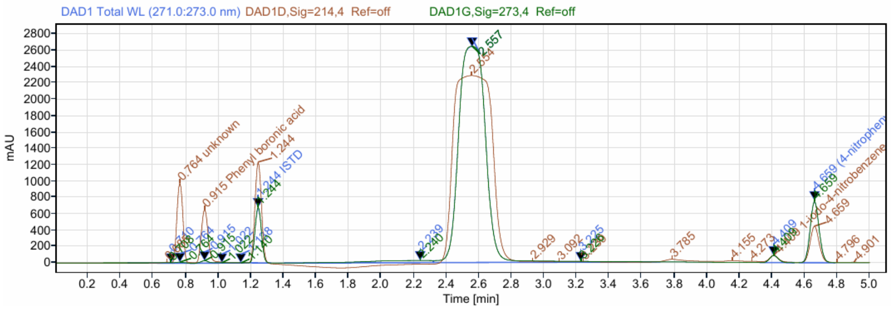

Sample Name	Name	RT (mins)	Area	Compound Amount (mg/mL)
	unknown	0.764	2438.8091	
	Phenyl boronic acid	0.915	1804.6595	0.109
	ISTD	1.244	1873.9612	1.000
	1-iodo-4-nitrobenzene	4.409	288.3611	0.070
	(4-nitrophenyl)(phenyl)methone	4.659	2899.2346	0.156

Figure S131:HPLC trace of crude reaction mixture from KD-029E-031. SilaCO with modified KF/18-C-6 trigger system as CO source.

Injection Report - By Sample

Kineticolor

**Sample name:**	29E-019		
**Data file:**	2025-02-14 07-36-29+00-00-02.dx	**Operator:**	SYSTEM
**Instrument:**	1220 Infinity II HPLC	**Injection date:**	2025-02-14 07:38:07+00:00
**Inj. volume:**	5.000 µL	**Location:**	25
**Acq. method:**	KD 29E.amx	**Type:**	Sample
**Processing method:**	3D UV Quantitative_DefaultMethod_KD 29E_unknown.pmx		
**Manually modified:**	Manual Integration		

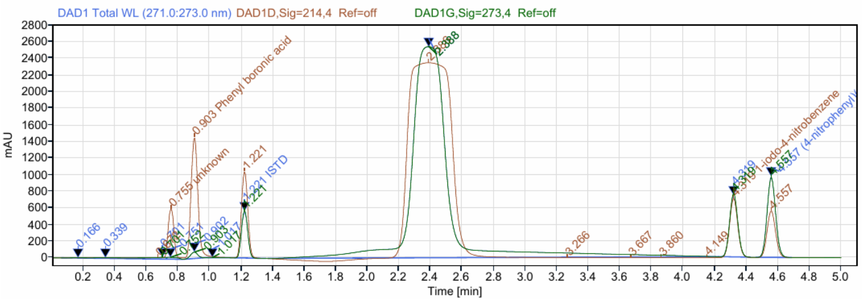

Sample Name	Name	RT (mins)	Area	Compound Amount
29E-019	unknown	0.755	1461.1050	
29E-019	Phenyl boronic acid	0.903	4251.2376	0.349
29E-019	ISTD	1.221	1566.5111	1.000
29E-019	1-iodo-4-nitrobenzene	4.319	2592.6151	0.609
29E-019	(4-nitrophenyl)(phenyl)methone	4.557	3713.3949	0.244

Figure S132: HPLC trace of crude reaction mixture from KD-029E-019. Formic acid as CO source.

Injection Report - By Sample

Kineticolor

**Sample name:**	29E-015		
**Data file:**	2025-01-29 07-50-54+00-00-02.dx	**Operator:**	SYSTEM
**Instrument:**	1220 Infinity II HPLC	**Injection date:**	2025-01-29 07:52:28+00:00
**Inj. volume:**	5.000 µL	**Location:**	15
**Acq. method:**	KD 29E.amx	**Type:**	Sample
**Processing method:**	3D UV Quantitative_DefaultMethod_KD 29E_unknown.pmx		
**Manually modified:**	Manual Integration		

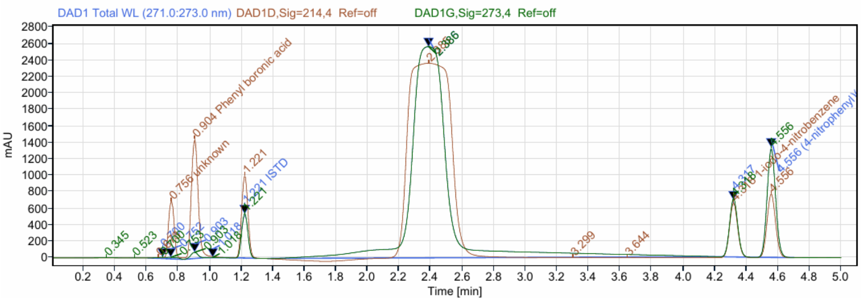

Sample Name	Name	RT (mins)	Area	Compound Amount
29E-015	unknown	0.756	1700.8720	
29E-015	Phenyl boronic acid	0.904	4164.0784	0.365
29E-015	ISTD	1.221	1481.9754	1.000
29E-015	1-iodo-4-nitrobenzene	4.318	2399.2773	0.596
29E-015	(4-nitrophenyl)(phenyl)methone	4.556	5092.2789	0.357

Figure S133: HPLC trace of crude reaction mixture from KD-029E-015. Formic acid as CO source.

Injection Report - By Sample

Kineticolor

**Sample name:**	KD-029E-010 25 micro		
**Data file:**	2025-01-10 09-23-24+00-00-03.dx	**Operator:**	SYSTEM
**Instrument:**	1220 Infinity II HPLC	**Injection date:**	2025-01-10 09:24:56+00:00
**Inj. volume:**	5.000 µL	**Location:**	12
**Acq. method:**	KD 29E.amx	**Type:**	Sample
**Processing method:**	3D UV Quantitative_DefaultMethod_KD 29E_unknown.pmx		
**Manually modified:**	Manual Integration		

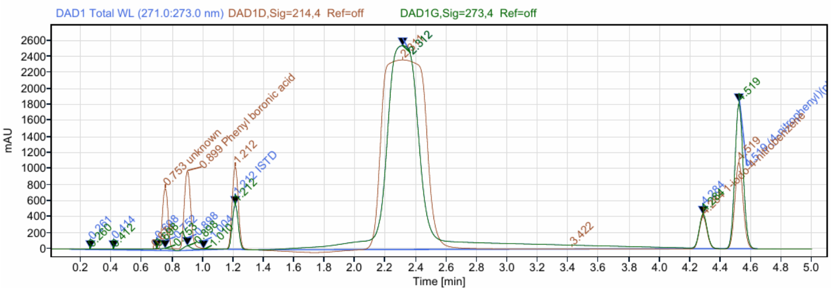

Sample Name	Name	RT (mins)	Area	Compound Amount
KD-029E-010 25 micro	unknown	0.753	1910.0888	
KD-029E-010 25 micro	Phenyl boronic acid	0.899	2749.7791	0.211
KD-029E-010 25 micro	ISTD	1.212	1534.9044	1.000
KD-029E-010 25 micro	1-iodo-4-nitrobenzene	4.284	1454.9736	0.355
KD-029E-010 25 micro	(4-nitrophenyl)(phenyl)methone	4.519	7046.6652	0.479

Figure S134: HPLC trace of crude reaction mixture from KD-029E-010. Formic acid as CO source.

Injection Report - By Sample

Kineticolor

**Sample name:**	KD-029E-014
**Data file:**	2025-01-28 07-43-08+00-00-02.dx
**Operator:**	SYSTEM
**Instrument:**	1220 Infinity II HPLC
**Injection date:**	2025-01-28 07:44:41+00:00
**Inj. volume:**	5.000 µL
**Location:**	14
**Acq. method:**	KD 29E.amx
**Type:**	Sample
**Processing method:**	3D UV Quantitative_DefaultMethod_KD 29E_unknown.pmx
**Manually modified:**	Manual Integration

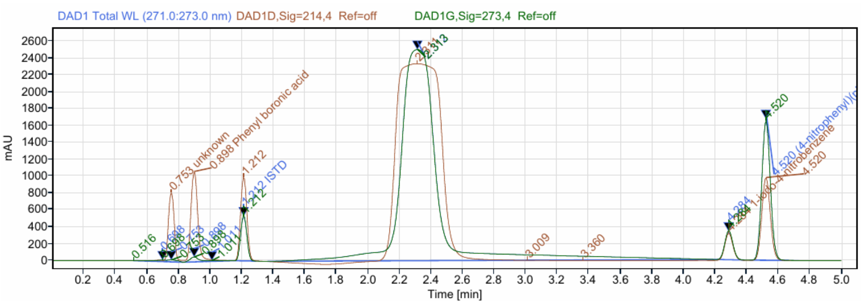

Sample Name	Name	RT (mins)	Area	Compound Amount
KD-029E-014	unknown	0.753	1986.7644	
KD-029E-014	Phenyl boronic acid	0.898	2982.1481	0.244
KD-029E-014	ISTD	1.212	1467.2249	1.000
KD-029E-014	1-iodo-4-nitrobenzene	4.284	1168.2908	0.300
KD-029E-014	(4-nitrophenyl)(phenyl)methone	4.520	6387.1697	0.454

Figure S135: HPLC trace of crude reaction mixture from KD-029E-014. $Mo(CO)_6$ as CO source.

Injection Report - By Sample

Kineticolor

**Sample name:**	29E-021		
**Data file:**	2025-02-20 09-28-45+00-00-02.dx	**Operator:**	SYSTEM
**Instrument:**	1220 Infinity II HPLC	**Injection date:**	2025-02-20 09:30:23+00:00
**Inj. volume:**	5.000 µL	**Location:**	61
**Acq. method:**	KD 29E.amx	**Type:**	Sample
**Processing method:**	3D UV Quantitative_DefaultMethod_KD 29E_unknown.pmx		
**Manually modified:**	Manual Integration		

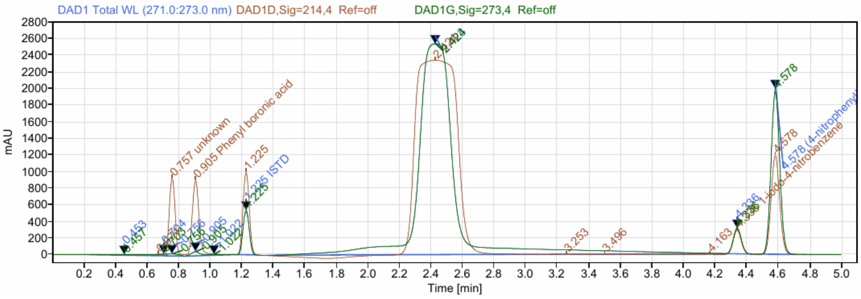

Sample Name	Name	RT (mins)	Area	Compound Amount
29E-021	unknown	0.757	2313.3333	
29E-021	Phenyl boronic acid	0.905	2487.3387	0.198
29E-021	ISTD	1.225	1473.8324	1.000
29E-021	1-iodo-4-nitrobenzene	4.336	1054.0615	0.271
29E-021	(4-nitrophenyl)(phenyl)methone	4.578	7721.9496	0.548

Figure S136:HPLC trace of crude reaction mixture from KD-029E-021. $Mo(CO)_6$ as CO source.

Injection Report - By Sample

Kineticolor

**Sample name:**	29E-023		
**Data file:**	2025-03-04 08-24-54+00-00-02.dx	**Operator:**	SYSTEM
**Instrument:**	1220 Infinity II HPLC	**Injection date:**	2025-03-04 08:26:30+00:00
**Inj. volume:**	5.000 µL	**Location:**	61
**Acq. method:**	KD 29E.amx	**Type:**	Sample
**Processing method:**	3D UV Quantitative_DefaultMethod_KD 29E_unknown.pmx		
**Manually modified:**	Manual Integration		

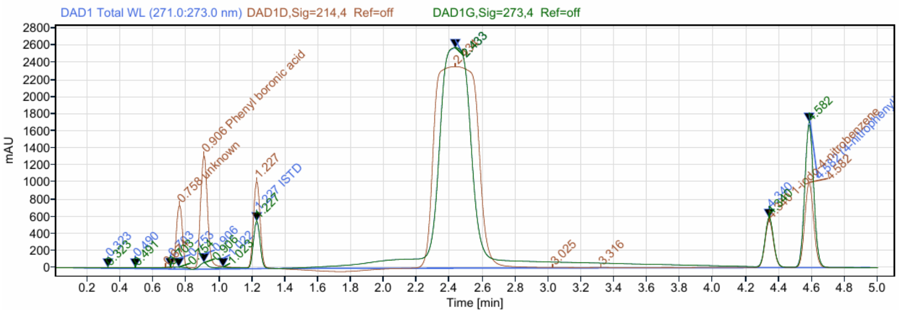

Sample Name	Name	RT (mins)	Area	Compound Amount (mg/mL)
29E-023	unknown	0.758	1823.7763	
29E-023	Phenyl boronic acid	0.906	3802.3565	0.302
29E-023	ISTD	1.227	1567.6015	1.000
29E-023	1-iodo-4-nitrobenzene	4.340	1968.9660	0.466
29E-023	(4-nitrophenyl)(phenyl)methone	4.582	6534.0359	0.434

Figure S137: HPLC trace of crude reaction mixture from KD-029E-023. $Mo(CO)_6$ as CO source.

Injection Report - By Sample **_Kineticolor_**

**Sample name:**	29E-018		
**Data file:**	2025-02-13 08-23-01+00-00-02.dx	**Operator:**	SYSTEM
**Instrument:**	1220 Infinity II HPLC	**Injection date:**	2025-02-13 08:24:37+00:00
**Inj. volume:**	5.000 µL	**Location:**	21
**Acq. method:**	KD 29E.amx	**Type:**	Sample
**Processing method:**	3D UV Quantitative_DefaultMethod_KD 29E_unknown.pmx		
**Manually modified:**	Manual Integration		

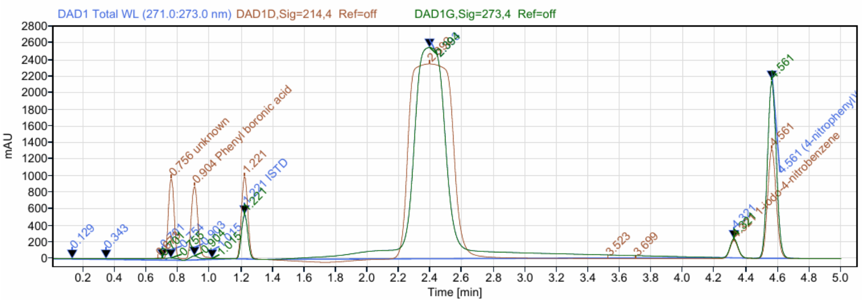

Sample Name	Name	RT (mins)	Area	Compound Amount
29E-018	unknown	0.756	2426.6546	
29E-018	Phenyl boronic acid	0.904	2479.5139	0.194
29E-018	ISTD	1.221	1492.8677	1.000
29E-018	1-iodo-4-nitrobenzene	4.321	791.1998	0.205
29E-018	(4-nitrophenyl)(phenyl)methone	4.561	8467.4766	0.594

Figure S138: HPLC trace of crude reaction mixture from KD-029E-018. SilaCO as CO source.

Injection Report - By Sample

Kineticolor

**Sample name:**	29E-017		
**Data file:**	2025-02-11 07-37-19+00-00-02.dx	**Operator:**	SYSTEM
**Instrument:**	1220 Infinity II HPLC	**Injection date:**	2025-02-11 07:38:51+00:00
**Inj. volume:**	5.000 µL	**Location:**	17
**Acq. method:**	KD 29E.amx	**Type:**	Sample
**Processing method:**	3D UV Quantitative_DefaultMethod_KD 29E_unknown.pmx		
**Manually modified:**	Manual Integration		

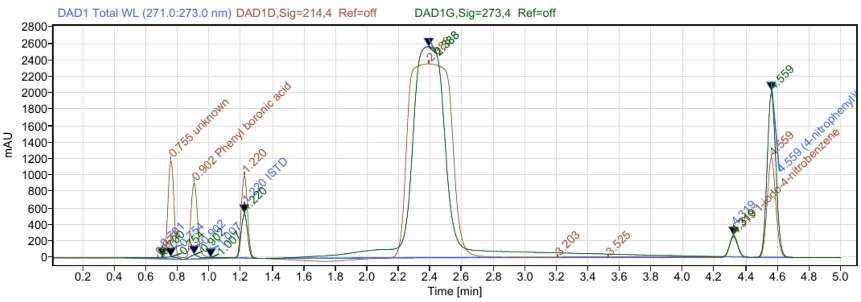

Sample Name	Name	RT (mins)	Area	Compound Amount
29E-017	unknown	0.755	3034.9979	
29E-017	Phenyl boronic acid	0.902	2589.7992	0.203
29E-017	ISTD	1.220	1498.5214	1.000
29E-017	1-iodo-4-nitrobenzene	4.319	915.7879	0.234
29E-017	(4-nitrophenyl)(phenyl)methone	4.559	7868.4174	0.549

Figure S139: HPLC trace of crude reaction mixture from KD-029E-017. SilaCO as CO source.

Injection Report - By Sample

Kineticolor

**Sample name:**	KD-029E-013		
**Data file:**	2025-01-21 07-54-20+00-00-02.dx	**Operator:**	SYSTEM
**Instrument:**	1220 Infinity II HPLC	**Injection date:**	2025-01-21 07:55:54+00:00
**Inj. volume:**	5.000 µL	**Location:**	9
**Acq. method:**	KD 29E.amx	**Type:**	Sample
**Processing method:**	3D UV Quantitative_DefaultMethod_KD 29E_unknown.pmx		
**Manually modified:**	Manual Integration		

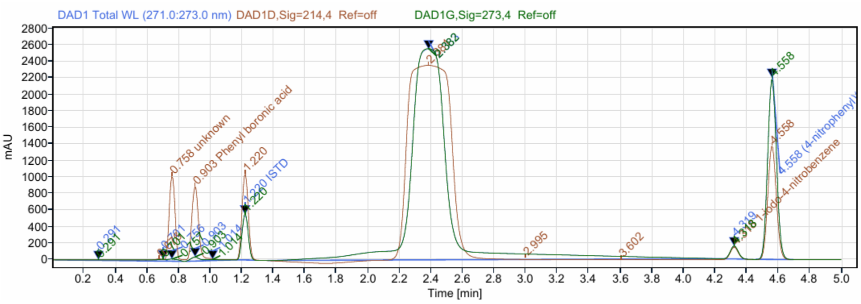

Sample Name	Name	RT (mins)	Area	Compound Amount
KD-029E-013	unknown	0.758	2556.7332	
KD-029E-013	Phenyl boronic acid	0.903	2477.8492	0.190
KD-029E-013	ISTD	1.220	1523.2945	1.000
KD-029E-013	1-iodo-4-nitrobenzene	4.318	547.0232	0.143
KD-029E-013	(4-nitrophenyl)(phenyl)methone	4.558	8643.8209	0.594

Figure S140: HPLC trace of crude reaction mixture from KD-029E-013. SilaCO as CO source.

10. References

[1] B. J. Wilson, J. N. Brantley, "Synthesis and Reactivity of Metallocarbene-Containing Polymers" *J. Am. Chem. Soc.* **2019**, *141*, 12453–12457.

[2] M. E. Moragues, A. Toscani, F. Sancenón, R. Martínez-Máñez, A. J. P. White, J. D. E. T. Wilton-Ely, "A chromo-fluorogenic synthetic 'canary' for CO detection based on a pyrenylvinyl ruthenium(II) complex" *J. Am. Chem. Soc.* **2014**, *136*, 11930–11933.

[3] A. Ahlburg, A. T. Lindhardt, R. H. Taaning, A. E. Modvig, T. Skrydstrup, "An air-tolerant approach to the carbonylative suzuki-miyaura coupling: Applications in isotope labeling" *Journal of Organic Chemistry* **2013**, *78*, 10310–10318.

[4] T. L. Andersen, S. D. Friis, H. Audrain, P. Nordeman, G. Antoni, T. Skrydstrup, "Efficient 11C-carbonylation of isolated aryl palladium complexes for PET: Application to challenging radiopharmaceutical synthesis" *J. Am. Chem. Soc.* **2015**, *137*, 1548–1555.

[5] D. Benito-Garagorri, M. Puchberger, K. Mereiter, K. Kirchner, "Stereospecific and reversible CO binding at iron pincer complexes" *Angewandte Chemie - International Edition* **2008**, *47*, 9142–9145.

[6] R. E. Winecker, *SOP-074-Determination of Carbon Monoxide by Palladium Chloride Determination of Carbon Monoxide by Use of Palladium Revision: Revision Date/Initials: Approving Authority Name Approving Authority Signature Approval Date SOP-074-Determination of Carbon Monoxide by Palladium Chloride*, **n.d.**

[7] H. J. Yun Chang Kristin Donnachie Timothy D McCabe Henry Barrington Felicity Carlysle-Davies Kristin Ceniccola-Campos Marc Reid, M. Reid, "Presumptive Tests for Xylazine-A Computer Vision Approach" **2025**, DOI 10.1002/ansa.70008.

[8] H. Barrington, A. Dickinson, J. McGuire, C. Yan, M. Reid, "Computer Vision for Kinetic Analysis of Lab- and Process-Scale Mixing Phenomena" *Org. Process Res. Dev.* **2022**, *26*, 3073–3088.

[9] C. Yan, C. Fyfe, L. Minty, H. Barrington, C. Jamieson, M. Reid, "Computer vision as a new paradigm for monitoring of solution and solid phase peptide synthesis" *Chem. Sci.* **2023**, *14*, 11872–11880.

[10] N. Bugeja, C. Oliver, N. McGrath, J. McGuire, C. Yan, F. Carlysle-Davies, M. Reid, "Teaching old presumptive tests new digital tricks with computer vision for forensic applications" *Digital Discovery* **2023**, *2*, 1143–1151.

[11] H. Barrington, T. J. D. McCabe, K. Donnachie, C. Fyfe, A. McFall, M. Gladkikh, J. McGuire, C. Yan, M. Reid, "Parallel and High Throughput Reaction Monitoring with Computer Vision" *Angewandte Chemie International Edition* **2024**, *64*, e202413395.

[12] C. Yan, M. Cowie, C. Howcutt, K. M. P. Wheelhouse, N. S. Hodnett, M. Kollie, M. Gildea, M. H. Goodfellow, M. Reid, "Computer vision for non-contact monitoring of catalyst degradation and product formation kinetics" *Chem. Sci.* **2023**, *14*, 5323–5331.
